# Supplementary material for: Proteoform Patterns in Hepatocellular Carcinoma Tissues: Aspects of Oncomarkers
Source: Proteomes. 2025 Jul 1;13(3):27. doi: 10.3390/proteomes13030027 (PMC12285994; doi:10.3390/proteomes13030027)
Supplement: Supplementary file 1 [file proteomes-13-00027-s001.zip › Supplementary Table S3.pdf]

Table S3: The selected proteins with different proteoform patterns in HCC and control	
**HCC**	**HCC control**
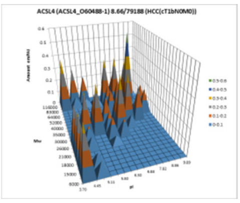	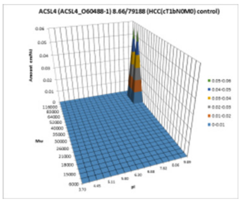
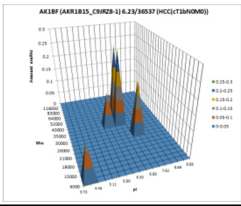	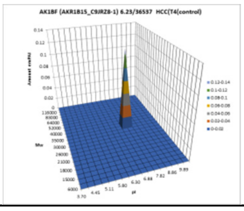
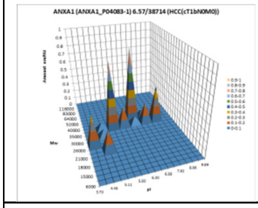	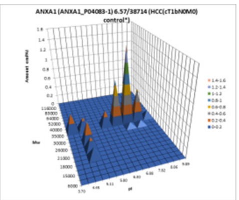
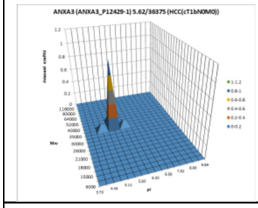	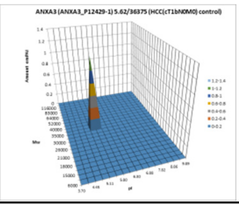
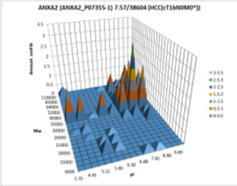	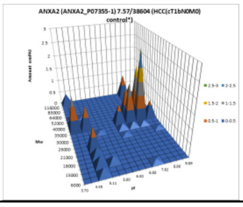
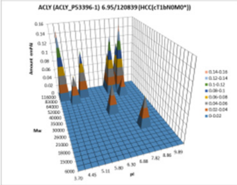	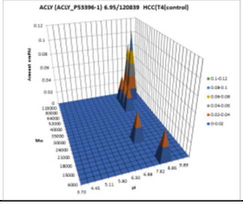
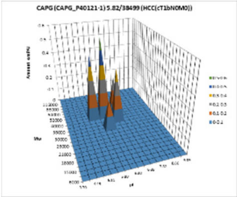	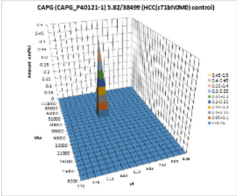

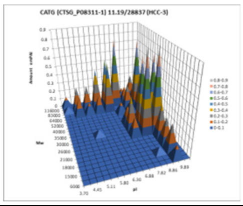
CATG (CTSG_P08311-1) 11.19/28837 (HCC-3)

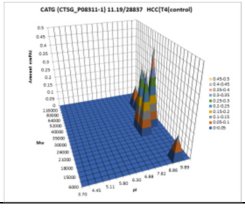
CATG (CTSG_P08311-1) 11.19/28837 HCC(T4(control)

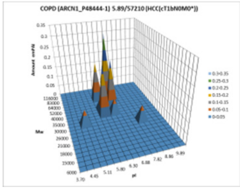
COPD (ARCN1_P48444-1) 5.89/57210 (HCC(cT1bN0M0*))

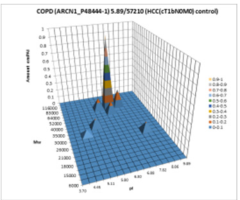
COPD (ARCN1_P48444-1) 5.89/57210 (HCC(cT1bN0M0) control)

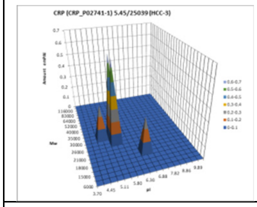
CRP (CRP_P02741-1) 5.45/25039 (HCC-3)

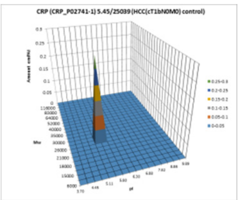
CRP (CRP_P02741-1) 5.45/25039 (HCC(cT1bN0M0) control)

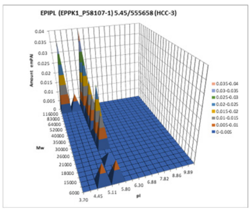
EPIPL (EPPK1_P58107-1) 5.45/555658 (HCC-3)

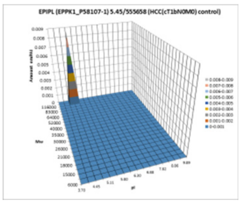
EPIPL (EPPK1_P58107-1) 5.45/555658 (HCC(cT1bN0M0) control)

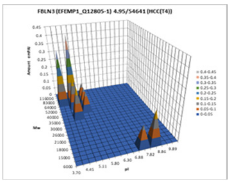
FBLN3 (EFEMP1_Q12805-1) 4.95/54641 (HCC(T4))

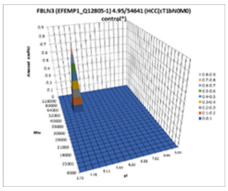
FBLN3 (EFEMP1_Q12805-1) 4.95/54641 (HCC(cT1bN0M0)
control(*)

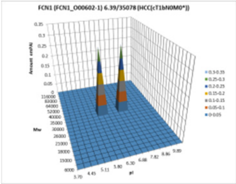
FCN1 (FCN1_O00602-1) 6.39/35078 (HCC(cT1bN0M0*))

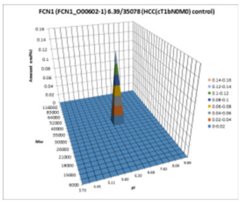
FCN1 (FCN1_O00602-1) 6.39/35078 (HCC(cT1bN0M0) control)

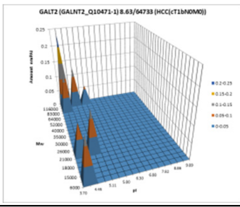
GALT2 (GALNT2_Q10471-1) 8.63/64733 (HCC(cT1bN0M0))

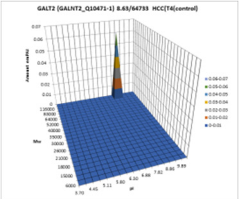
GALT2 (GALNT2_Q10471-1) 8.63/64733 HCC(T4(control)

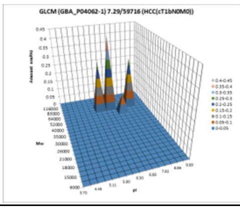
GLCM (GBA_P04062-1) 7.29/59716 (HCC(cT1bN0M0))

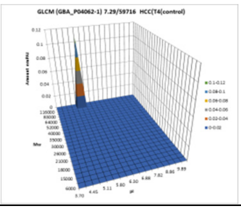
GLCM (GBA_P04062-1) 7.29/59716 HCC(T4(control)

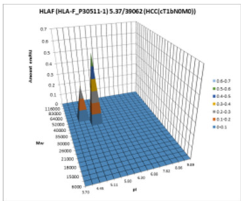HLAF (HLA-F_P30511-1) 5.37/39062 (HCC(cT1bN0M0))	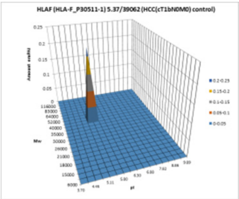HLAF (HLA-F_P30511-1) 5.37/39062 (HCC(cT1bN0M0) control)
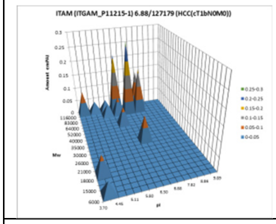ITAM (ITGAM_P11215-1) 6.88/127179 (HCC(cT1bN0M0))	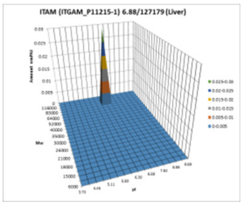ITAM (ITGAM_P11215-1) 6.88/127179 (Liver)
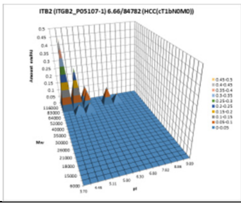ITB2 (ITGB2_P05107-1) 6.66/84782 (HCC(cT1bN0M0))	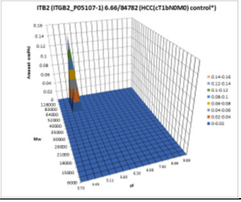ITB2 (ITGB2_P05107-1) 6.66/84782 (HCC(cT1bN0M0) control*)
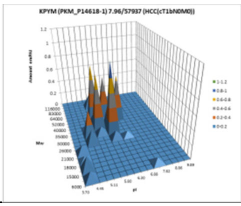KPYM (PKM_P14618-1) 7.96/57937 (HCC(cT1bN0M0))	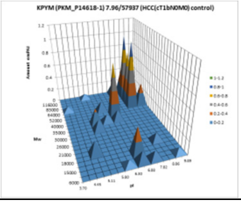KPYM (PKM_P14618-1) 7.96/57937 (HCC(cT1bN0M0) control)
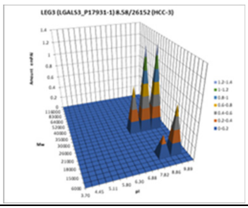LEG3 (LGALS3_P17931-1) 8.58/26152 (HCC-3)	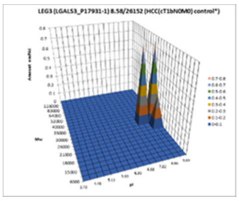LEG3 (LGALS3_P17931-1) 8.58/26152 (HCC(cT1bN0M0) control*)
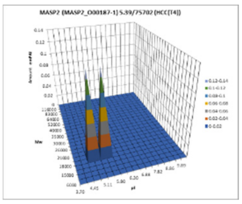MASP2 (MASP2_O00187-1) 5.39/75702 (HCC(T4))	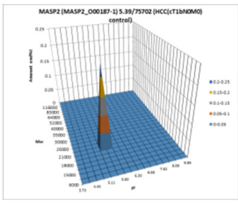MASP2 (MASP2_O00187-1) 5.39/75702 (HCC(cT1bN0M0) control)
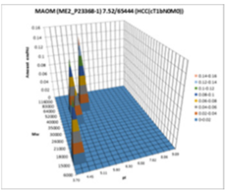MAOM (ME2_P23368-1) 7.52/65444 (HCC(cT1bN0M0))	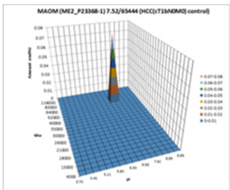MAOM (ME2_P23368-1) 7.52/65444 (HCC(cT1bN0M0) control)
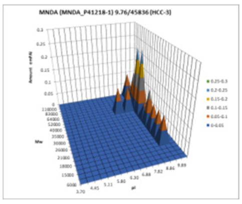MNDA (MNDA_P41218-1) 9.76/45836 (HCC-3)	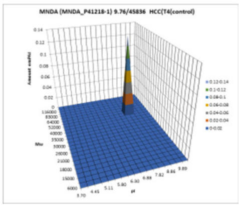MNDA (MNDA_P41218-1) 9.76/45836 HCC(T4(control)

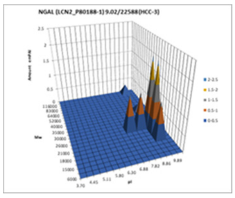NGAL (LCN2_P80188-1) 9.02/22588(HCC-3)	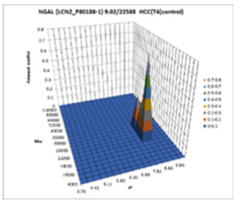NGAL (LCN2_P80188-1) 9.02/22588 HCC(T4(control)
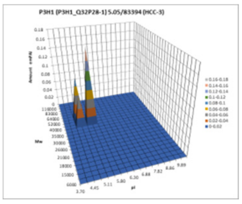P3H1 (P3H1_Q32P28-1) 5.05/83394 (HCC-3)	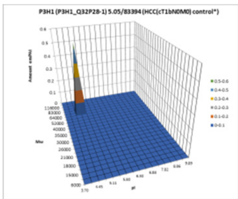P3H1 (P3H1_Q32P28-1) 5.05/83394 (HCC(cT1bN0M0) control*)
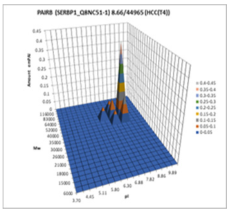PAIRB (SERBP1_Q8NC51-1) 8.66/44965 (HCC(T4))	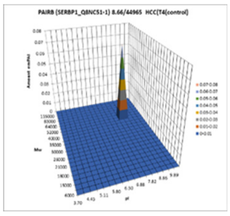PAIRB (SERBP1_Q8NC51-1) 8.66/44965 HCC(T4(control)
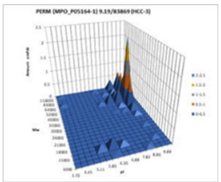PERM (MPO_P05164-1) 9.19/83869 (HCC-3)	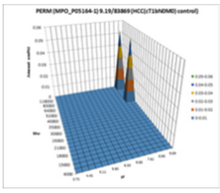PERM (MPO_P05164-1) 9.19/83869 (HCC(cT1bN0M0) control)
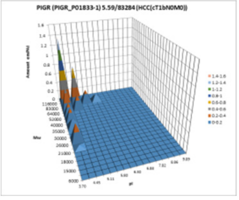PIGR (PIGR_P01833-1) 5.59/83284 (HCC(cT1bN0M0))	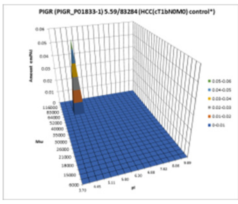PIGR (PIGR_P01833-1) 5.59/83284 (HCC(cT1bN0M0) control*)
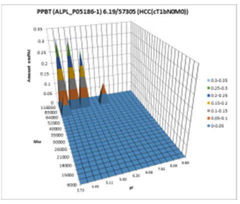PPBT (ALPL_P05186-1) 6.19/57305 (HCC(cT1bN0M0))	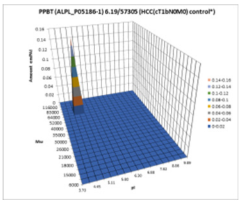PPBT (ALPL_P05186-1) 6.19/57305 (HCC(cT1bN0M0) control*)
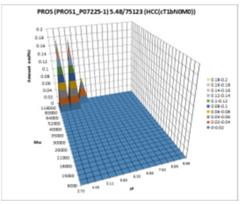PROS (PROS1_P07225-1) 5.48/75123 (HCC(cT1bN0M0))	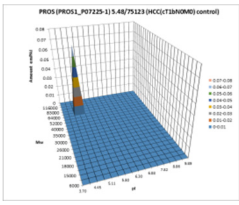PROS (PROS1_P07225-1) 5.48/75123 (HCC(cT1bN0M0) control)

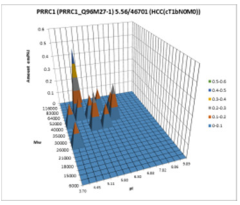
PRRC1 (PRRC1_Q96M27-1) 5.56/46701 (HCC(cT1bN0M0))

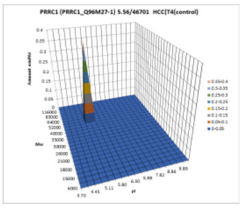
PRRC1 (PRRC1_Q96M27-1) 5.56/46701 HCC(T4(control)

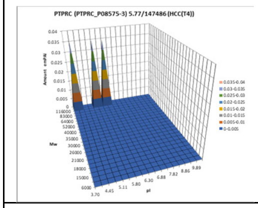
PTPRC (PTPRC_P08575-3) 5.77/147486 (HCC(T4))

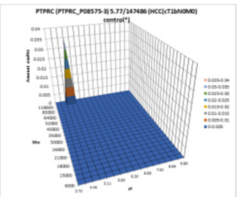
PTPRC (PTPRC_P08575-3) 5.77/147486 (HCC(cT1bN0M0) control*)

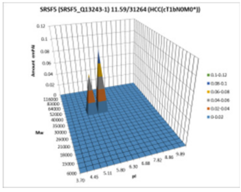
SRSF5 (SRSF5_Q13243-1) 11.59/31264 (HCC(cT1bN0M0*))

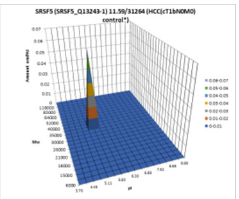
SRSF5 (SRSF5_Q13243-1) 11.59/31264 (HCC(cT1bN0M0) control*)

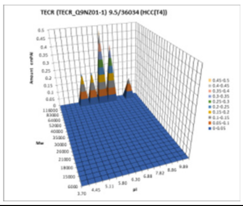
TECR (TECR_Q9NZ01-1) 9.5/36034 (HCC(T4))

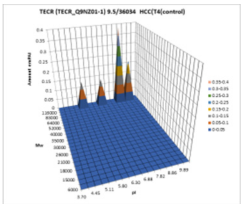
TECR (TECR_Q9NZ01-1) 9.5/36034 HCC(T4(control)

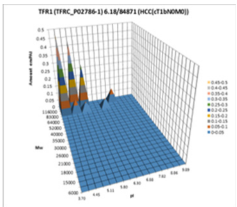
TFR1 (TFRC_P02786-1) 6.18/84871 (HCC(cT1bN0M0))

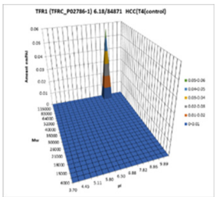
TFR1 (TFRC_P02786-1) 6.18/84871 HCC(T4(control)

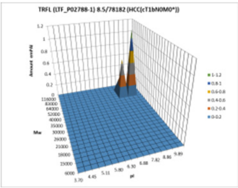
TRFL (LTF_P02788-1) 8.5/78182 (HCC(cT1bN0M0*))

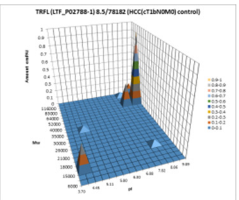
TRFL (LTF_P02788-1) 8.5/78182 (HCC(cT1bN0M0) control)

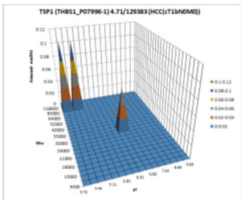
TSP1 (THBS1_P07996-1) 4.71/129383 (HCC(cT1bN0M0))

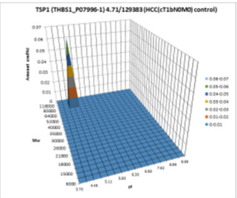
TSP1 (THBS1_P07996-1) 4.71/129383 (HCC(cT1bN0M0) control)

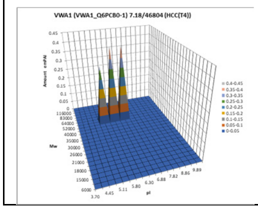
VWA1 (VWA1_Q6PCB0-1) 7.18/46804 (HCC(T4))

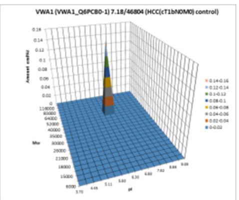
VWA1 (VWA1_Q6PCB0-1) 7.18/46804 (HCC(cT1bN0M0) control)
