# Supplementary material for: Proteoform Patterns in Hepatocellular Carcinoma Tissues: Aspects of Oncomarkers
Source: Proteomes. 2025 Jul 1;13(3):27. doi: 10.3390/proteomes13030027 (PMC12285994; doi:10.3390/proteomes13030027)
Supplement: Supplementary file 1 [file proteomes-13-00027-s001.zip › Supplementary Table S4.pdf]

Supplementary Table S4

A complete list of proteins identified by two methods – 2DE sectional and panoramic proteomic profiling (FASP).

Uniprot	Protein	Gene	Description	2DE (T) mPAI(%)	2DE (C) emPAI(%)	FC 2DE T/C	FASP (T) mPAI(%)	FASP (C) mPAI(%)	FC FASP T/C
P11171	41	EPB41	Protein 4.1	0.0023	0.0002	13.78	0.0060	0.0043	1.38
P31946	1433B	YWHAB	14-3-3 protein beta/alpha	0.1212	0.0880	1.38	0.1155	0.0838	1.38
P62258	1433E	YWHAE	14-3-3 protein epsilon	0.2028	0.2521	0.80	0.1520	0.1391	1.09
Q04917	1433F	YWHAH	14-3-3 protein eta	0.0522	0.0319	1.64	0.0370	0.0375	0.99
P61981	1433G	YWHAG	14-3-3 protein gamma	0.1280	0.0804	1.59	0.0928	0.0604	1.54
P31947	1433S	SFN	14-3-3 protein sigma	0.0056	-	+/-	-	-	-/-
P27348	1433T	YWHAQ	14-3-3 protein theta	0.0565	0.0413	1.37	0.0756	0.0713	1.06
P63104	1433Z	YWHAZ	14-3-3 protein zeta/delta	0.1920	0.0999	1.92	0.2123	0.1075	1.97
P30443	1A01	HLA-A	HLA class I histocompatibility antigen, A-1 alpha chain	0.0088	-	+/-	-	-	-/-
P01892	1A02	HLA-A	HLA class I histocompatibility antigen, A-2 alpha chain	0.0090	0.0012	7.50	-	-	-/-
P04439	1A03	HLA-A	HLA class I histocompatibility antigen, A-3 alpha chain	0.0469	0.0151	3.10	0.0909	0.0298	3.05
P18462	1A25	HLA-A	HLA class I histocompatibility antigen, A-25 alpha chain	0.0168	0.0029	5.79	-	-	-/-
P10314	1A32	HLA-A	HLA class I histocompatibility antigen, A-32 alpha chain	0.0368	0.0060	6.10	-	-	-/-
P01891	1A68	HLA-A	HLA class I histocompatibility antigen, A-68 alpha chain	0.0017	-	+/-	-	-	-/-

P01889	1B07	HLA-B	HLA class I histocompatibility antigen, B-7 alpha chain	0.0234	0.0069	3.41	0.0699	0.0241	2.90
P30486	1B48	HLA-B	HLA class I histocompatibility antigen, B-48 alpha chain	-	0.0012	-/+	-	-	-/-
P10319	1B58	HLA-B	HLA class I histocompatibility antigen, B-58 alpha chain	0.0016	-	+/-	-	-	-/-
P04222	1C03	HLA-C	HLA class I histocompatibility antigen, Cw-3 alpha chain	0.0072	-	+/-	-	-	-/-
P10321	1C07	HLA-C	HLA class I histocompatibility antigen, Cw-7 alpha chain	0.0141	0.0047	3.01	0.0689	0.0175	3.93
Q29960	1C16	HLA-C	HLA class I histocompatibility antigen, Cw-16 alpha chain	-	0.0010	-/+	-	-	-/-
Q15172	2A5A	PPP2R5A	Serine/threonine-protein phosphatase 2A 56 kDa regulatory subunit alpha isoform	0.0002	-	+/-	0.0079	-	+/-
Q14738	2A5D	PPP2R5D	Serine/threonine-protein phosphatase 2A 56 kDa regulatory subunit delta isoform	0.0001	-	+/-	-	-	-/-
Q16537	2A5E	PPP2R5E	Serine/threonine-protein phosphatase 2A 56 kDa regulatory subunit epsilon isoform	-	-	-/-	0.0046	-	+/-
Q13362	2A5G	PPP2R5C	Serine/threonine-protein phosphatase 2A 56 kDa regulatory subunit gamma isoform	-	-	-/-	-	0.0030	-/+
P30153	2AAA	PPP2R1A	Serine/threonine-protein phosphatase 2A 65 kDa regulatory subunit A alpha isoform	0.0202	0.0220	0.92	0.0397	0.0357	1.11
P30154	2AAB	PPP2R1B	Serine/threonine-protein phosphatase 2A 65 kDa regulatory subunit A beta isoform	0.0008	0.0026	0.31	-	-	-/-

P63151	2ABA	PPP2R2A	Serine/threonine-protein phosphatase 2A 55 kDa regulatory subunit B alpha isoform	0.0005	0.0017	0.31	0.0036	-	+/-
Q66LE6	2ABD	PPP2R2D	Serine/threonine-protein phosphatase 2A 55 kDa regulatory subunit B delta isoform	-	0.0005	-/+	-	-	-/-
P13761	2B17	HLA-DRB1	HLA class II histocompatibility antigen, DRB1-7 beta chain	0.0176	0.0085	2.06	-	-	-/-
Q9H2F3	3BHS7	HSD3B7	3 beta-hydroxysteroid dehydrogenase type 7	0.0019	-	+/-	-	0.0064	-/+
P46952	3HAO	HAAO	3-hydroxyanthranilate 3,4-dioxygenase	0.0413	0.1040	0.40	0.0379	0.1039	0.36
P31937	3HIDH	HIBADH	3-hydroxyisobutyrate dehydrogenase, mitochondrial	0.0470	0.1402	0.33	0.0382	0.1666	0.23
P29372	3MG	MPG	DNA-3-methyladenine glycosylase	0.0002	-	+/-	-	-	-/-
P08195	4F2	SLC3A2	4F2 cell-surface antigen heavy chain	0.0006	-	+/-	0.0054	0.0056	0.97
Q9H0P0	5NT3A	NT5C3A	Cytosolic 5'-nucleotidase 3A	0.0003	0.0007	0.48	-	-	-/-
Q969T7	5NT3B	NT5C3B	7-methylguanosine phosphate-specific 5'-nucleotidase	0.0005	0.0005	0.89	-	-	-/-
P49902	5NTC	NT5C2	Cytosolic purine 5'-nucleotidase	-	-	-/-	0.0030	-	+/-
P21589	5NTD	NT5E	5'-nucleotidase	0.0041	0.0026	1.59	0.0107	0.0071	1.51
P52209	6PGD	PGD	6-phosphogluconate dehydrogenase, decarboxylating	0.0335	0.0260	1.29	0.0810	0.0604	1.34
O95336	6PGL	PGLS	6-phosphogluconolactonase	0.0386	0.0526	0.73	0.0624	0.0892	0.70
P36639	8ODP	NUDT1	7,8-dihydro-8-oxoguanine triphosphatase	0.0010	0.0026	0.37	-	-	-/-

Q8IZ83	A16A1	ALDH16A1	Aldehyde dehydrogenase family 16 member A1	0.0014	0.0012	1.15	0.0108	0.0175	0.62
P02763	A1AG1	ORM1	Alpha-1-acid glycoprotein 1	0.1223	0.0548	2.23	0.0806	0.0480	1.68
P19652	A1AG2	ORM2	Alpha-1-acid glycoprotein 2	0.0357	0.0292	1.22	0.0302	0.0236	1.28
P01009	A1AT	SERPINA1	Alpha-1-antitrypsin	0.5061	0.2494	2.03	0.2803	0.1609	1.74
P04217	A1BG	A1BG	Alpha-1B-glycoprotein	0.0259	0.0145	1.79	0.0282	0.0238	1.19
Q9NQ94	A1CF	A1CF	APOBEC1 complementation factor	0.0149	0.0188	0.79	0.0163	0.0169	0.97
P08697	A2AP	SERPINF2	Alpha-2-antiplasmin	0.0132	0.0037	3.51	0.0021	-	+/-
P02750	A2GL	LRG1	Leucine-rich alpha-2-glycoprotein	0.0068	0.0057	1.18	0.0082	-	+/-
P01023	A2MG	A2M	Alpha-2-macroglobulin	0.1215	0.0496	2.45	0.0603	0.0631	0.96
P22760	AAAD	AADAC	Arylacetamide deacetylase	0.0270	0.0212	1.27	0.0533	0.0700	0.76
Q9NRG9	AAAS	AAAS	Aladin	-	-	-/-	0.0047	-	+/-
P01011	AACT	SERPINA3	Alpha-1-antichymotrypsin	0.1933	0.0548	3.53	0.1030	0.0120	8.58
Q8N5Z0	AADAT	AADAT	Kynurenine/alpha-aminoadipate aminotransferase, mitochondrial	0.0017	0.0187	0.09	-	0.0280	-/+
Q9Y478	AAKB1	PRKAB1	5'-AMP-activated protein kinase subunit beta-1	0.0022	0.0018	1.22	-	-	-/-
O43741	AAKB2	PRKAB2	5'-AMP-activated protein kinase subunit beta-2	0.0133	0.0246	0.54	0.0116	0.0168	0.69
P54619	AAKG1	PRKAG1	5'-AMP-activated protein kinase subunit gamma-1	0.0050	0.0118	0.42	0.0335	0.0296	1.13
Q9H7C9	AAMDC	AAMDC	Mth938 domain-containing protein	0.0027	0.0067	0.40	-	-	-/-

Q13131	AAPK1	PRKAA1	5'-AMP-activated protein kinase catalytic subunit alpha-1	0.0043	0.0025	1.69	0.0114	0.0171	0.67
P54646	AAPK2	PRKAA2	5'-AMP-activated protein kinase catalytic subunit alpha-2	0.0004	-	+/-	-	-	-/-
Q9BTE6	AASD1	AARSD1	Alanyl-tRNA editing protein Aarsd1	0.0008	0.0013	0.66	-	-	-/-
Q9UDR5	AASS	AASS	Alpha-aminoadipic semialdehyde synthase, mitochondrial	0.0018	0.0052	0.33	-	0.0316	-/+
P17174	AATC	GOT1	Aspartate aminotransferase, cytoplasmic	0.0792	0.1174	0.67	0.1548	0.1665	0.93
Q9NY61	AATF	AATF	Protein AATF	0.0001	-	+/-	-	-	-/-
P00505	AATM	GOT2	Aspartate aminotransferase, mitochondrial	0.0833	0.2188	0.38	0.0617	0.2461	0.25
Q7Z5R6	AB1IP	APBB1IP	Amyloid beta A4 precursor protein-binding family B member 1-interacting protein	0.0005	-	+/-	0.0040	-	+/-
Q8N139	ABCA6	ABCA6	ATP-binding cassette sub-family A member 6	0.0007	0.0002	2.98	0.0068	0.0050	1.36
O94911	ABCA8	ABCA8	ABC-type organic anion transporter ABCA8	-	0.0001	-/+	-	0.0024	-/+
Q9NP58	ABCB6	ABCB6	ATP-binding cassette sub-family B member 6, mitochondrial	0.0002	-	+/-	0.0025	-	+/-
O75027	ABCB7	ABCB7	ATP-binding cassette sub-family B member 7, mitochondrial	0.0006	-	+/-	0.0036	0.0024	1.47
Q9NRK6	ABCBA	ABCB10	ATP-binding cassette sub-family B member 10, mitochondrial	0.0002	-	+/-	0.0024	-	+/-
O95342	ABCBB	ABCB11	Bile salt export pump	0.0007	0.0005	1.35	0.0052	0.0093	0.56
P33897	ABCD1	ABCD1	ATP-binding cassette sub-family D member 1	0.0007	-	+/-	0.0118	-	+/-

P28288	ABCD3	ABCD3	ATP-binding cassette sub-family D member 3	0.0065	0.0024	2.68	0.0295	0.0206	1.43
P61221	ABCE1	ABCE1	ATP-binding cassette sub-family E member 1	0.0012	0.0005	2.33	0.0146	0.0069	2.10
Q8NE71	ABCF1	ABCF1	ATP-binding cassette sub-family F member 1	0.0012	-	+/-	0.0042	0.0036	1.17
Q9UG63	ABCF2	ABCF2	ATP-binding cassette sub-family F member 2	0.0004	-	+/-	0.0022	-	+/-
Q9NUQ8	ABCF3	ABCF3	ATP-binding cassette sub-family F member 3	0.0001	0.0001	0.61	0.0055	0.0022	2.51
Q8N2K0	ABD12	ABHD12	Lysophosphatidylserine lipase ABHD12	-	-	-/-	0.0060	-	+/-
Q6UXT9	ABH15	ABHD15	Protein ABHD15	-	-	-/-	-	0.0039	-/+
Q8WTS1	ABHD5	ABHD5	1-acylglycerol-3-phosphate O-acyltransferase ABHD5	-	0.0004	-/+	-	-	-/-
Q9BV23	ABHD6	ABHD6	Monoacylglycerol lipase ABHD6	-	-	-/-	-	0.0062	-/+
Q9NUJ1	ABHDA	ABHD10	Mycophenolic acid acyl-glucuronide esterase, mitochondrial	0.0112	0.0085	1.32	0.0120	0.0161	0.75
Q8NFV4	ABHDB	ABHD11	Protein ABHD11	0.0037	0.0042	0.89	-	-	-/-
Q96IU4	ABHEB	ABHD14B	Protein ABHD14B	0.1317	0.1481	0.89	0.0979	0.1879	0.52
O95870	ABHGA	ABHD16A	Phosphatidylserine lipase ABHD16A	0.0003	-	+/-	0.0086	-	+/-
Q8IZP0	ABI1	ABI1	Abl interactor 1	0.0011	0.0003	3.59	0.0045	-	+/-
Q9P2A4	ABI3	ABI3	ABI gene family member 3	-	0.0024	-/+	-	-	-/-
Q9NX38	ABITM	ABITRAM	Protein Abitram	0.0003	-	+/-	-	-	-/-
O14639	ABLM1	ABLIM1	Actin-binding LIM protein 1	0.0001	0.0001	0.75	-	-	-/-
O94929-2	ABLM3	ABLIM3	Isoform 2 of Actin-binding LIM protein 3	-	0.0002	-/+	0.0027	0.0043	0.63
Q9P1F3	ABRAL	ABRACL	Costars family protein ABRACL	0.0062	0.0027	2.28	-	-	-/-

Q15018	ABRX2	ABRAXAS2	BRISC complex subunit Abraxas 2	0.0002	0.0002	0.76	-	-	-/-
Q13085	ACACA	ACACA	Acetyl-CoA carboxylase 1	-	-	-/-	0.0019	0.0040	0.47
O00763	ACACB	ACACB	Acetyl-CoA carboxylase 2	0.0003	0.0005	0.59	-	0.0119	-/+
Q9UKU7	ACAD8	ACAD8	Isobutyryl-CoA dehydrogenase, mitochondrial	0.0015	0.0045	0.34	0.0044	0.0070	0.63
Q9H845	ACAD9	ACAD9	Acyl-CoA dehydrogenase family member 9, mitochondrial	0.0007	0.0008	0.85	0.0147	0.0113	1.30
P28330	ACADL	ACADL	Long-chain specific acyl-CoA dehydrogenase, mitochondrial	0.0019	0.0045	0.42	0.0058	0.0104	0.55
P11310	ACADM	ACADM	Medium-chain specific acyl-CoA dehydrogenase, mitochondrial	0.0430	0.0653	0.66	0.0315	0.0593	0.53
P16219	ACADS	ACADS	Short-chain specific acyl-CoA dehydrogenase, mitochondrial	0.0490	0.0785	0.62	0.0385	0.1106	0.35
P49748	ACADV	ACADVL	Very long-chain specific acyl-CoA dehydrogenase, mitochondrial	0.0625	0.0663	0.94	0.0835	0.1385	0.60
Q15057	ACAP2	ACAP2	Arf-GAP with coiled-coil, ANK repeat and PH domain-containing protein 2	-	-	-/-	0.0027	-	+/-
O00400	ACATN	SLC33A1	Acetyl-coenzyme A transporter 1	0.0002	-	+/-	0.0068	-	+/-
Q5T8D3-2	ACBD5	ACBD5	Isoform 2 of Acyl-CoA-binding domain-containing protein 5	0.0011	0.0021	0.52	0.0086	-	+/-
Q5T8D3-4	ACBD5	ACBD5	Isoform 4 of Acyl-CoA-binding domain-containing protein 5	0.0004	-	+/-	-	-	-/-

Q5T8D3	ACBD5	ACBD5	Acyl-CoA-binding domain-containing protein 5	0.0033	0.0006	5.97	0.0080	0.0034	2.35
Q9BR61	ACBD6	ACBD6	Acyl-CoA-binding domain-containing protein 6	0.0015	0.0013	1.15	-	-	-/-
P07108	ACBP	DBI	Acyl-CoA-binding protein	0.0128	0.0218	0.59	0.0036	0.0120	0.30
Q6JQN1	ACD10	ACAD10	Acyl-CoA dehydrogenase family member 10	0.0008	-	+/-	0.0042	0.0034	1.22
Q709F0	ACD11	ACAD11	Acyl-CoA dehydrogenase family member 11	0.0039	0.0053	0.73	0.0069	0.0172	0.40
P45954	ACDSB	ACADSB	Short/branched chain specific acyl-CoA dehydrogenase, mitochondrial	0.0806	0.1343	0.60	0.0709	0.1463	0.48
Q9UKV3	ACINU	ACIN1	Apoptotic chromatin condensation inducer in the nucleus	0.0000	-	+/-	0.0012	-	+/-
O96019	ACL6A	ACTL6A	Actin-like protein 6A	0.0032	0.0022	1.44	0.0103	-	+/-
P53396	ACLY	ACLY	ATP-citrate synthase	0.0196	0.0011	17.24	0.0224	0.0188	1.19
Q8TDX5	ACMSD	ACMSD	2-amino-3-carboxymuconate-6-semialdehyde decarboxylase	0.0010	0.0064	0.16	-	0.0110	-/+
Q8WYK0	ACO12	ACOT12	Acetyl-coenzyme A thioesterase	-	0.0002	-/+	-	0.0035	-/+
Q9NPJ3	ACO13	ACOT13	Acyl-coenzyme A thioesterase 13	0.0188	0.0820	0.23	0.0124	0.0326	0.38
P21399	ACOC	ACO1	Cytoplasmic aconitate hydratase	0.0308	0.0678	0.45	0.0423	0.0968	0.44
Q99798	ACON	ACO2	Aconitate hydratase, mitochondrial	0.0211	0.0295	0.71	0.0433	0.0641	0.68
Q86TX2	ACOT1	ACOT1	Acyl-coenzyme A thioesterase 1	0.0047	0.0114	0.42	-	-	-/-
P49753-2	ACOT2	ACOT2	Isoform 2 of Acyl-coenzyme A thioesterase 2, mitochondrial	-	0.0004	-/+	-	-	-/-

P49753	ACOT2	ACOT2	Acyl-coenzyme A thioesterase 2, mitochondrial	0.0152	0.0498	0.30	0.0247	0.0764	0.32
Q8N9L9	ACOT4	ACOT4	Peroxisomal succinyl-coenzyme A thioesterase	0.0004	-	+/-	0.0069	-	+/-
O14734	ACOT8	ACOT8	Acyl-coenzyme A thioesterase 8	0.0017	0.0015	1.14	0.0233	0.0068	3.41
Q9Y305	ACOT9	ACOT9	Acyl-coenzyme A thioesterase 9, mitochondrial	0.0033	0.0021	1.56	0.0073	0.0055	1.32
Q15067-2	ACOX1	ACOX1	Isoform 2 of Peroxisomal acyl-coenzyme A oxidase 1	0.0005	0.0031	0.17	0.0386	0.0427	0.90
Q15067	ACOX1	ACOX1	Peroxisomal acyl-coenzyme A oxidase 1	0.0130	0.0246	0.53	0.0359	0.0665	0.54
Q99424	ACOX2	ACOX2	Peroxisomal acyl-coenzyme A oxidase 2	0.0093	0.0129	0.72	0.0385	0.0652	0.59
O15254	ACOX3	ACOX3	Peroxisomal acyl-coenzyme A oxidase 3	0.0015	0.0006	2.76	0.0288	0.0120	2.40
P13798	ACPH	APEH	Acylamino-acid-releasing enzyme	0.0118	0.0170	0.70	0.0342	0.0521	0.66
O14561	ACPM	NDUFAB1	Acyl carrier protein, mitochondrial	0.0009	0.0029	0.30	0.0031	0.0031	0.98
Q08AH3	ACS2A	ACSM2A	Acyl-coenzyme A synthetase ACSM2A, mitochondrial	0.0289	0.0679	0.43	0.0727	0.1852	0.39
Q68CK6	ACS2B	ACSM2B	Acyl-coenzyme A synthetase ACSM2B, mitochondrial	0.0329	0.0771	0.43	0.0786	0.1622	0.48
Q9NR19	ACSA	ACSS2	Acetyl-coenzyme A synthetase, cytoplasmic	0.0001	0.0009	0.13	0.0055	0.0158	0.35
Q96CM8	ACSF2	ACSF2	Medium-chain acyl-CoA ligase ACSF2, mitochondrial	0.0023	0.0052	0.44	0.0110	0.0318	0.35
Q4G176	ACSF3	ACSF3	Malonate--CoA ligase ACSF3, mitochondrial	0.0013	0.0007	1.92	0.0118	0.0122	0.97

P33121	ACSL1	ACSL1	Long-chain-fatty-acid--CoA ligase 1	0.1033	0.1850	0.56	0.0745	0.1876	0.40
P33121-3	ACSL1	ACSL1	Isoform 3 of Long-chain-fatty-acid--CoA ligase 1	-	-	-/-	-	0.1188	-/+
O95573	ACSL3	ACSL3	Long-chain-fatty-acid--CoA ligase 3	0.0033	0.0002	19.87	0.0209	0.0095	2.21
O60488	ACSL4	ACSL4	Long-chain-fatty-acid--CoA ligase 4	0.0990	0.0003	375.38	0.1909	-	+/-
Q9ULC5	ACSL5	ACSL5	Long-chain-fatty-acid--CoA ligase 5	0.0090	0.0213	0.42	0.0266	0.0823	0.32
Q9UKU0	ACSL6	ACSL6	Long-chain-fatty-acid--CoA ligase 6	0.0007	-	+/-	-	-	-/-
Q53FZ2	ACSM3	ACSM3	Acyl-coenzyme A synthetase ACSM3, mitochondrial	0.0046	0.0132	0.35	0.0006	0.0363	0.02
Q6NUN0	ACSM5	ACSM5	Acyl-coenzyme A synthetase ACSM5, mitochondrial	0.0055	0.0116	0.47	0.0080	0.0394	0.20
Q9H6R3	ACSS3	ACSS3	Acyl-CoA synthetase short-chain family member 3, mitochondrial	0.0038	0.0050	0.75	0.0107	0.0405	0.26
P62736	ACTA	ACTA2	Actin, aortic smooth muscle	0.7702	0.4109	1.87	0.4297	0.2295	1.87
P60709	ACTB	ACTB	Actin, cytoplasmic 1	2.3875	1.1846	2.02	1.3574	0.6655	2.04
Q562R1	ACTBL	ACTBL2	Beta-actin-like protein 2	0.2982	0.0665	4.49	0.0863	0.0702	1.23
Q9BYX7	ACTBM	POTEKP	Putative beta-actin-like protein 3	0.0011	0.0012	0.92	-	-	-/-
P68032	ACTC	ACTC1	Actin, alpha cardiac muscle 1	0.1271	-	+/-	0.4406	0.2255	1.95
P63261	ACTG	ACTG1	Actin, cytoplasmic 2	0.3683	0.4231	0.87	1.4272	0.7367	1.94
P63267	ACTH	ACTG2	Actin, gamma-enteric smooth muscle	-	0.0113	-/+	-	-	-/-
P12814	ACTN1	ACTN1	Alpha-actinin-1	0.1110	0.0611	1.82	0.1298	0.0873	1.49

P35609	ACTN2	ACTN2	Alpha-actinin-2	0.0008	-	+/-	-	-	-/-
Q08043	ACTN3	ACTN3	Alpha-actinin-3	0.0001	-	+/-	-	-	-/-
O43707-3	ACTN4	ACTN4	Isoform 3 of Alpha-actinin-4	-	0.0003	-/+	-	-	-/-
O43707	ACTN4	ACTN4	Alpha-actinin-4	0.1924	0.1315	1.46	0.1637	0.1926	0.85
P68133	ACTS	ACTA1	Actin, alpha skeletal muscle	0.0811	0.0345	2.35	-	-	-/-
P42025	ACTY	ACTR1B	Beta-centractin	0.0032	0.0069	0.47	0.0113	0.0173	0.66
P61163	ACTZ	ACTR1A	Alpha-centractin	0.0059	0.0089	0.66	0.0221	0.0198	1.12
Q03154	ACY1	ACY1	Aminoacylase-1	0.0706	0.1154	0.61	0.0648	0.1135	0.57
P45381	ACY2	ASPA	Aspartoacylase	0.0006	0.0025	0.22	-	-	-/-
Q96HD9	ACY3	ACY3	N-acyl-aromatic-L-amino acid amidohydrolase (carboxylate-forming)	0.0051	0.0119	0.43	-	0.0203	-/+
P07311	ACYP1	ACYP1	Acylphosphatase-1	-	0.0013	-/+	-	-	-/-
P14621	ACYP2	ACYP2	Acylphosphatase-2	0.0007	0.0064	0.12	-	-	-/-
P00813	ADA	ADA	Adenosine deaminase	0.0048	0.0014	3.40	-	-	-/-
O14672	ADA10	ADAM10	Disintegrin and metalloproteinase domain-containing protein 10	-	0.0003	-/+	0.0033	-	+/-
Q9NZK5	ADA2	ADA2	Adenosine deaminase 2	0.0002	-	+/-	0.0033	-	+/-
O00116	ADAS	AGPS	Alkyldihydroxyacetonephosphate synthase, peroxisomal	0.0013	-	+/-	0.0178	0.0040	4.46
Q08828	ADCY1	ADCY1	Adenylate cyclase type 1	0.0001	-	+/-	-	-	-/-
P35611	ADDA	ADD1	Alpha-adducin	0.0022	0.0012	1.85	0.0096	0.0047	2.04

P35612	ADDB	ADD2	Beta-adducin	-	0.0001	-/+	-	-	-/-
Q9UEY8	ADDG	ADD3	Gamma-adducin	0.0002	0.0001	1.57	0.0035	-	+/-
P07327	ADH1A	ADH1A	Alcohol dehydrogenase 1A	0.2068	0.6545	0.32	0.1675	0.7128	0.23
P00325	ADH1B	ADH1B	Alcohol dehydrogenase 1B	0.2852	0.7872	0.36	0.1975	1.0268	0.19
P00326	ADH1G	ADH1C	Alcohol dehydrogenase 1C	0.1869	0.4577	0.41	0.1861	0.5095	0.37
P08319	ADH4	ADH4	Alcohol dehydrogenase 4	0.2225	0.5873	0.38	0.2210	0.4989	0.44
P28332	ADH6	ADH6	Alcohol dehydrogenase 6	0.0406	0.0935	0.43	0.0384	0.2043	0.19
P11766	ADHX	ADH5	Alcohol dehydrogenase class-3	0.0312	0.0504	0.62	0.0595	0.0760	0.78
Q15848	ADIPO	ADIPOQ	Adiponectin	0.0006	-	+/-	-	-	-/-
P55263	ADK	ADK	Adenosine kinase	0.0100	0.0287	0.35	0.0213	0.0374	0.57
Q9BRR6	ADPGK	ADPGK	ADP-dependent glucokinase	0.0005	0.0003	1.72	0.0084	-	+/-
Q9NRN7	ADPPT	AASDHPPT	L-aminoadipate-semialdehyde dehydrogenase-phosphopantetheinyl transferase	0.0012	0.0023	0.53	-	-	-/-
Q16186	ADRM1	ADRM1	Proteasomal ubiquitin receptor ADRM1	0.0027	0.0037	0.74	0.0143	-	+/-
P22570	ADRO	FDXR	NADPH:adrenodoxin oxidoreductase, mitochondrial	0.0147	0.0321	0.46	0.0099	0.0362	0.27
P12235	ADT1	SLC25A4	ADP/ATP translocase 1	0.0220	0.0151	1.46	0.0809	0.0701	1.15
P05141	ADT2	SLC25A5	ADP/ATP translocase 2	0.0817	0.0640	1.28	0.1047	0.0966	1.08
P12236	ADT3	SLC25A6	ADP/ATP translocase 3	0.0106	0.0041	2.59	0.0839	0.0595	1.41
P10109	ADX	FDX1	Adrenodoxin, mitochondrial	0.0098	0.0179	0.55	-	-	-/-

Q8IUX7	AEBP1	AEBP1	Adipocyte enhancer-binding protein 1	0.0002	-	+/-	0.0050	-	+/-
P55196	AFAD	AFDN	Afadin	-	0.0001	-/+	0.0008	0.0016	0.48
P55196-1	AFAD	AFDN	Isoform 2 of Afadin	-	-	-/-	0.0024	0.0012	1.96
P43652	AFAM	AFM	Afamin	0.0014	0.0013	1.12	0.0026	0.0026	0.97
Q8N556	AFAP1	AFAP1	Actin filament-associated protein 1	-	0.0001	-/+	-	-	-/-
Q9Y4W6	AFG32	AFG3L2	AFG3-like protein 2	0.0034	0.0014	2.41	0.0055	0.0077	0.71
P06280	AGAL	GLA	Alpha-galactosidase A	0.0012	-	+/-	0.0051	-	+/-
P52594	AGFG1	AGFG1	Arf-GAP domain and FG repeat-containing protein 1	0.0010	-	+/-	-	-	-/-
O95081	AGFG2	AGFG2	Arf-GAP domain and FG repeat-containing protein 2	0.0009	0.0007	1.20	-	-	-/-
Q53H12	AGK	AGK	Acylglycerol kinase, mitochondrial	0.0035	0.0006	6.29	0.0082	0.0062	1.32
O95394	AGM1	PGM3	Phosphoacetylglucosamine mutase	0.0033	0.0038	0.88	0.0054	0.0193	0.28
Q9UL18	AGO1	AGO1	Protein argonaute-1	-	-	-/-	0.0039	-	+/-
Q9UKV8	AGO2	AGO2	Protein argonaute-2	0.0003	-	+/-	0.0038	-	+/-
Q9HCK5	AGO4	AGO4	Protein argonaute-4	-	-	-/-	0.0020	-	+/-
O00468-3	AGRIN	AGRN	Isoform 3 of Agrin	0.0002	-	+/-	0.0066	-	+/-
O00468	AGRIN	AGRN	Agrin	0.0042	0.0002	17.76	0.0003	0.0016	0.19
O95490	AGRL2	ADGRL2	Adhesion G protein-coupled receptor L2	0.0001	-	+/-	-	-	-/-
Q9HBW9	AGRL4	ADGRL4	Adhesion G protein-coupled receptor L4	0.0009	-	+/-	-	-	-/-

Q9BYV1	AGT2	AGXT2	Alanine--glyoxylate aminotransferase 2, mitochondrial	0.0024	0.0074	0.32	0.0086	0.0456	0.19
Q09666	AHNK	AHNAK	Neuroblast differentiation-associated protein AHNAK	0.0041	0.0012	3.31	0.0059	0.0050	1.18
Q8IVF2	AHNK2	AHNAK2	Protein AHNAK2	-	0.0000	-/+	-	-	-/-
O95433	AHSA1	AHSA1	Activator of 90 kDa heat shock protein ATPase homolog 1	0.0076	0.0051	1.48	0.0073	0.0049	1.49
Q96BJ3	AIDA	AIDA	Axin interactor, dorsalization-associated protein	0.0011	0.0022	0.50	0.0047	-	+/-
O95831	AIFM1	AIFM1	Apoptosis-inducing factor 1, mitochondrial	0.0585	0.0471	1.24	0.0324	0.0445	0.73
Q12904	AIMP1	AIMP1	Aminoacyl tRNA synthase complex-interacting multifunctional protein 1	0.0065	0.0064	1.02	0.0140	-	+/-
Q13155	AIMP2	AIMP2	Aminoacyl tRNA synthase complex-interacting multifunctional protein 2	0.0042	0.0074	0.57	0.0138	0.0103	1.34
O00170	AIP	AIP	AH receptor-interacting protein	0.0074	0.0044	1.68	0.0122	0.0047	2.63
P14550	AK1A1	AKR1A1	Aldo-keto reductase family 1 member A1	0.0904	0.1730	0.52	0.1248	0.1891	0.66
O60218	AK1BA	AKR1B10	Aldo-keto reductase family 1 member B10	0.3162	0.0014	232.26	0.4175	0.0052	80.78
C9JRZ8	AK1BF	AKR1B15	Aldo-keto reductase family 1 member B15	0.0220	0.0003	81.90	0.0788	-	+/-
C9JRZ8-2	AK1BF	AKR1B15	Isoform 2 of Aldo-keto reductase family 1 member B15	0.0007	0.0006	1.26	-	-	-/-
Q04828	AK1C1	AKR1C1	Aldo-keto reductase family 1 member C1	0.0795	0.0993	0.80	0.3258	0.2889	1.13
P52895-2	AK1C2	AKR1C2	Isoform 2 of Aldo-keto reductase family 1 member C2	0.0011	-	+/-	-	-	-/-

P52895	AK1C2	AKR1C2	Aldo-keto reductase family 1 member C2	0.1167	0.1561	0.75	0.2180	0.1672	1.30
P42330	AK1C3	AKR1C3	Aldo-keto reductase family 1 member C3	0.1640	0.1063	1.54	0.2052	0.1379	1.49
P17516	AK1C4	AKR1C4	Aldo-keto reductase family 1 member C4	0.1991	0.2687	0.74	0.3691	0.4948	0.75
P51857-3	AK1D1	AKR1D1	Isoform 3 of Aldo-keto reductase family 1 member D1	0.0043	0.0017	2.52	-	-	-/-
P51857	AK1D1	AKR1D1	Aldo-keto reductase family 1 member D1	0.0305	0.0612	0.50	0.0478	0.0534	0.89
O43572	AKA10	AKAP10	A-kinase anchor protein 10, mitochondrial	-	-	-/-	0.0028	-	+/-
Q02952	AKA12	AKAP12	A-kinase anchor protein 12	0.0001	-	+/-	-	-	-/-
Q92667	AKAP1	AKAP1	A-kinase anchor protein 1, mitochondrial	0.0003	-	+/-	-	-	-/-
Q9Y2D5	AKAP2	AKAP2	A-kinase anchor protein 2	0.0003	0.0009	0.35	-	0.0021	-/+
Q9Y2D5-4	AKAP2	AKAP2	Isoform 3 of A-kinase anchor protein 2	0.0006	0.0006	1.07	-	-	-/-
Q5JQC9	AKAP4	AKAP4	A-kinase anchor protein 4	0.0001	-	+/-	-	-	-/-
Q99996	AKAP9	AKAP9	A-kinase anchor protein 9	0.0000	-	+/-	-	-	-/-
Q9ULX6	AKP8L	AKAP8L	A-kinase anchor protein 8-like	-	0.0004	-/+	-	-	-/-
P31749	AKT1	AKT1	RAC-alpha serine/threonine-protein kinase	0.0004	0.0006	0.78	-	-	-/-
P31751	AKT2	AKT2	RAC-beta serine/threonine-protein kinase	-	0.0003	-/+	-	-	-/-
P00352	AL1A1	ALDH1A1	Retinal dehydrogenase 1	0.3266	0.2907	1.12	0.2944	0.3430	0.86
O94788	AL1A2	ALDH1A2	Retinal dehydrogenase 2	0.0003	-	+/-	-	-	-/-
P47895	AL1A3	ALDH1A3	Aldehyde dehydrogenase family 1 member A3	-	-	-/-	0.0089	0.0061	1.46
P30837	AL1B1	ALDH1B1	Aldehyde dehydrogenase X, mitochondrial	0.0509	0.0724	0.70	0.0406	0.0907	0.45

O75891-2	AL1L1	ALDH1L1	Isoform 2 of Cytosolic 10-formyltetrahydrofolate dehydrogenase	0.0002	-	+/-	-	-	-/-
O75891	AL1L1	ALDH1L1	Cytosolic 10-formyltetrahydrofolate dehydrogenase	0.0934	0.2094	0.45	0.0642	0.1480	0.43
Q3SY69	AL1L2	ALDH1L2	Mitochondrial 10-formyltetrahydrofolate dehydrogenase	0.0002	-	+/-	-	-	-/-
P30838	AL3A1	ALDH3A1	Aldehyde dehydrogenase, dimeric NADP-preferring	0.0011	0.0004	2.42	0.0091	-	+/-
P51648-2	AL3A2	ALDH3A2	Isoform 2 of Aldehyde dehydrogenase family 3 member A2	0.0003	-	+/-	-	-	-/-
P51648	AL3A2	ALDH3A2	Aldehyde dehydrogenase family 3 member A2	0.0533	0.0385	1.39	0.0565	0.0612	0.92
P30038	AL4A1	ALDH4A1	Delta-1-pyrroline-5-carboxylate dehydrogenase, mitochondrial	0.1055	0.1340	0.79	0.0797	0.1213	0.66
P20292	AL5AP	ALOX5AP	Arachidonate 5-lipoxygenase-activating protein	0.0025	-	+/-	-	-	-/-
P49419	AL7A1	ALDH7A1	Alpha-aminoadipic semialdehyde dehydrogenase	0.0407	0.0697	0.58	0.0874	0.1388	0.63
Q9H2A2-2	AL8A1	ALDH8A1	Isoform 2 of 2-aminomuconic semialdehyde dehydrogenase	-	0.0003	-/+	-	-	-/-
Q9H2A2	AL8A1	ALDH8A1	2-aminomuconic semialdehyde dehydrogenase	0.0196	0.0562	0.35	0.0377	0.1309	0.29
P49189	AL9A1	ALDH9A1	4-trimethylaminobutyraldehyde dehydrogenase	0.0579	0.0934	0.62	0.1095	0.1990	0.55

P24298	ALAT1	GPT	Alanine aminotransferase 1	0.0070	0.0257	0.27	0.0148	0.0883	0.17
P02768-3	ALBU	ALB	Isoform 3 of Serum albumin	-	0.0301	-/+	-	-	-/-
P02768	ALBU	ALB	Serum albumin	1.2344	0.7298	1.69	0.4802	0.4860	0.99
P05091	ALDH2	ALDH2	Aldehyde dehydrogenase, mitochondrial	0.3203	0.3642	0.88	0.1331	0.3114	0.43
P04075	ALDOA	ALDOA	Fructose-bisphosphate aldolase A	0.0862	0.0374	2.31	0.1161	0.0777	1.49
P05062	ALDOB	ALDOB	Fructose-bisphosphate aldolase B	0.3190	0.6793	0.47	0.2225	0.5642	0.39
P09972	ALDOC	ALDOC	Fructose-bisphosphate aldolase C	0.0108	0.0315	0.34	0.0103	0.0451	0.23
P15121	ALDR	AKR1B1	Aldo-keto reductase family 1 member B1	0.0154	0.0080	1.92	0.0547	0.0223	2.46
Q9BT22	ALG1	ALG1	Chitobiosyldiphosphodolichol beta-mannosyltransferase	-	-	-/-	0.0046	-	+/-
Q9NP73	ALG13	ALG13	Putative bifunctional UDP-N-acetylglucosamine transferase and deubiquitinase ALG13	0.0015	0.0010	1.55	-	-	-/-
Q9H553	ALG2	ALG2	Alpha-1,3/1,6-mannosyltransferase ALG2	0.0001	-	+/-	0.0038	0.0061	0.63
Q9Y673	ALG5	ALG5	Dolichyl-phosphate beta-glucosyltransferase	-	0.0002	-/+	0.0227	0.0144	1.57
Q9NXW9	ALKB4	ALKBH4	Alpha-ketoglutarate-dependent dioxygenase alkB homolog 4	0.0001	0.0005	0.32	-	-	-/-
Q9BT30	ALKB7	ALKBH7	Alpha-ketoglutarate-dependent dioxygenase alkB homolog 7, mitochondrial	0.0006	0.0026	0.24	-	-	-/-
Q6ZNB7	ALKMO	AGMO	Alkylglycerol monooxygenase	-	0.0003	-/+	0.0103	-	+/-
Q8TCU4	ALMS1	ALMS1	Alstrom syndrome protein 1	0.0000	-	+/-	-	-	-/-

P35858	ALS	IGFALS	Insulin-like growth factor-binding protein complex acid labile subunit	-	-	-/-	0.0036	0.0057	0.63
Q9UHK6	AMACR	AMACR	Alpha-methylacyl-CoA racemase	0.0022	0.0120	0.18	-	0.0321	-/+
P02760	AMBP	AMBP	Protein AMBP	0.0241	0.0083	2.91	0.0310	0.0304	1.02
Q8N7J2	AMER2	AMER2	APC membrane recruitment protein 2	0.0002	-	+/-	-	-	-/-
Q9H4A4	AMPB	RNPEP	Aminopeptidase B	0.0069	0.0108	0.64	0.0449	0.0488	0.92
Q01433	AMPD2	AMPD2	AMP deaminase 2	0.0001	0.0003	0.24	0.0027	0.0048	0.57
Q01432	AMPD3	AMPD3	AMP deaminase 3	0.0009	-	+/-	-	-	-/-
Q07075	AMPE	ENPEP	Glutamyl aminopeptidase	0.0005	-	+/-	0.0043	0.0022	1.98
P28838	AMPL	LAP3	Cytosol aminopeptidase	0.0575	0.0528	1.09	0.0707	0.0998	0.71
P15144	AMPN	ANPEP	Aminopeptidase N	0.0216	0.0084	2.56	0.0278	0.0309	0.90
P30533	AMRP	LRPAP1	Alpha-2-macroglobulin receptor-associated protein	0.0009	0.0004	2.10	-	-	-/-
P04745	AMY1	AMY1A	Alpha-amylase 1	-	0.0030	-/+	-	-	-/-
P0DTE7	AMY1B	AMY1B	Alpha-amylase 1B	0.0033	-	+/-	-	-	-/-
P04746	AMYP	AMY2A	Pancreatic alpha-amylase	0.0002	-	+/-	-	-	-/-
Q8IZ07	AN13A	ANKRD13A	Ankyrin repeat domain-containing protein 13A	-	0.0006	-/+	-	-	-/-
P39687	AN32A	ANP32A	Acidic leucine-rich nuclear phosphoprotein 32 family member A	0.0071	0.0084	0.85	0.0189	0.0019	9.94
Q92688	AN32B	ANP32B	Acidic leucine-rich nuclear phosphoprotein 32 family member B	0.0044	0.0069	0.64	0.0112	0.0115	0.97

Q9BTT0	AN32E	ANP32E	Acidic leucine-rich nuclear phosphoprotein 32 family member E	0.0059	0.0078	0.75	0.0259	-	+/-
P54802	ANAG	NAGLU	Alpha-N-acetylglucosaminidase	0.0007	-	+/-	0.0263	0.0070	3.78
Q96K21	ANCHR	ZFYVE19	Abscission/NoCut checkpoint regulator	0.0001	-	+/-	-	-	-/-
Q9P2R3	ANFY1	ANKFY1	Rabankyrin-5	0.0004	0.0003	1.19	0.0018	0.0028	0.64
P03950	ANGI	ANG	Angiogenin	-	-	-/-	0.0249	0.0113	2.20
Q8NI99	ANGL6	ANGPTL6	Angiopoietin-related protein 6	0.0001	0.0005	0.24	-	0.0175	-/+
P01019	ANGT	AGT	Angiotensinogen	0.0186	0.0133	1.40	0.0295	0.0287	1.03
P16157	ANK1	ANK1	Ankyrin-1	0.0021	0.0010	2.15	0.0013	0.0013	0.96
Q12955	ANK3	ANK3	Ankyrin-3	0.0003	0.0000	16.50	-	-	-/-
Q99873	ANM1	PRMT1	Protein arginine N-methyltransferase 1	0.0006	0.0007	0.88	0.0204	-	+/-
O14744	ANM5	PRMT5	Protein arginine N-methyltransferase 5	0.0003	0.0002	1.88	-	0.0032	-/+
Q4KMQ2	ANO6	ANO6	Anoctamin-6	0.0007	-	+/-	0.0048	-	+/-
Q6UB99	ANR11	ANKRD11	Ankyrin repeat domain-containing protein 11	0.0000	-	+/-	-	-	-/-
O15084	ANR28	ANKRD28	Serine/threonine-protein phosphatase 6 regulatory ankyrin repeat subunit A	-	0.0001	-/+	-	-	-/-
Q8N7Z5	ANR31	ANKRD31	Ankyrin repeat domain-containing protein 31	-	-	-/-	0.0008	-	+/-
Q8N8A2	ANR44	ANKRD44	Serine/threonine-protein phosphatase 6 regulatory ankyrin repeat subunit B	0.0001	-	+/-	-	-	-/-
P01008	ANT3	SERPINC1	Antithrombin-III	0.0827	0.0311	2.66	0.0653	0.0486	1.34
P50995	ANX11	ANXA11	Annexin A11	0.0171	0.0046	3.73	0.0472	0.0259	1.82

P27216	ANX13	ANXA13	Annexin A13	0.0038	0.0043	0.87	-	0.0040	-/+
P27216-2	ANX13	ANXA13	Isoform B of Annexin A13	-	0.0018	-/+	-	-	-/-
P04083	ANXA1	ANXA1	Annexin A1	0.0858	0.0529	1.62	0.0870	0.0388	2.24
P07355	ANXA2	ANXA2	Annexin A2	0.2631	0.1419	1.85	0.1560	0.1024	1.52
P12429	ANXA3	ANXA3	Annexin A3	0.0448	0.0060	7.50	0.0356	0.0067	5.33
P09525	ANXA4	ANXA4	Annexin A4	0.1803	0.1034	1.74	0.1132	0.0948	1.19
P08758	ANXA5	ANXA5	Annexin A5	0.2486	0.2027	1.23	0.1052	0.1308	0.80
P08133	ANXA6	ANXA6	Annexin A6	0.1456	0.1571	0.93	0.0863	0.1253	0.69
P20073	ANXA7	ANXA7	Annexin A7	0.0337	0.0176	1.92	0.0446	0.0368	1.21
O76027	ANXA9	ANXA9	Annexin A9	0.0045	0.0090	0.50	-	-	-/-
Q16853	AOC3	AOC3	Membrane primary amine oxidase	0.0048	0.0034	1.40	0.0153	0.0297	0.51
P21397	AOFA	MAOA	Amine oxidase [flavin-containing] A	0.0195	0.0237	0.82	0.0346	0.0570	0.61
P27338	AOFB	MAOB	Amine oxidase [flavin-containing] B	0.0392	0.0432	0.91	0.0548	0.1280	0.43
Q06278	AOXA	AOX1	Aldehyde oxidase	0.0310	0.0428	0.72	0.0581	0.1035	0.56
O60641	AP180	SNAP91	Clathrin coat assembly protein AP180	0.0001	-	+/-	-	-	-/-
Q10567-3	AP1B1	AP1B1	Isoform C of AP-1 complex subunit beta-1	0.0002	-	+/-	0.0324	0.0245	1.33
Q10567	AP1B1	AP1B1	AP-1 complex subunit beta-1	0.0075	0.0074	1.01	0.0376	0.0294	1.28
O43747	AP1G1	AP1G1	AP-1 complex subunit gamma-1	0.0006	0.0006	1.04	0.0085	0.0036	2.39
Q9BXS5	AP1M1	AP1M1	AP-1 complex subunit mu-1	0.0026	0.0011	2.41	0.0222	0.0150	1.48
P61966	AP1S1	AP1S1	AP-1 complex subunit sigma-1A	-	-	-/-	0.0133	-	+/-

O95782	AP2A1	AP2A1	AP-2 complex subunit alpha-1	0.0015	0.0006	2.33	0.0193	0.0164	1.17
O94973	AP2A2	AP2A2	AP-2 complex subunit alpha-2	0.0028	0.0022	1.24	0.0290	0.0299	0.97
O94973-2	AP2A2	AP2A2	Isoform 2 of AP-2 complex subunit alpha-2	-	-	-/-	-	0.0167	-/+
P63010	AP2B1	AP2B1	AP-2 complex subunit beta	0.0056	0.0071	0.79	0.0450	0.0370	1.22
Q96CW1	AP2M1	AP2M1	AP-2 complex subunit mu	0.0050	0.0029	1.71	0.0194	0.0179	1.08
P53680	AP2S1	AP2S1	AP-2 complex subunit sigma	0.0016	0.0008	1.97	0.0116	-	+/-
O00203	AP3B1	AP3B1	AP-3 complex subunit beta-1	0.0007	0.0002	3.28	0.0036	0.0030	1.22
O14617	AP3D1	AP3D1	AP-3 complex subunit delta-1	-	-	-/-	0.0074	-	+/-
Q9Y2T2	AP3M1	AP3M1	AP-3 complex subunit mu-1	0.0002	-	+/-	0.0226	0.0076	2.99
Q92572	AP3S1	AP3S1	AP-3 complex subunit sigma-1	0.0004	0.0010	0.41	-	-	-/-
P50583	AP4A	NUDT2	Bis (5'-nucleosyl)-tetraphosphatase [asymmetrical]	0.0040	0.0067	0.59	-	-	-/-
Q9UM13	APC10	ANAPC10	Anaphase-promoting complex subunit 10	0.0003	0.0005	0.67	-	-	-/-
P27695	APEX1	APEX1	DNA-(apurinic or apyrimidinic site) endonuclease	0.0123	0.0061	2.01	0.0202	0.0148	1.36
Q9BZZ5-2	API5	API5	Isoform 2 of Apoptosis inhibitor 5	0.0010	-	+/-	-	-	-/-
Q9BZZ5-1	API5	API5	Isoform 1 of Apoptosis inhibitor 5	0.0001	-	+/-	-	-	-/-
Q9BZZ5	API5	API5	Apoptosis inhibitor 5	0.0007	0.0005	1.31	0.0056	0.0058	0.97
Q9HDC9	APMAP	APMAP	Adipocyte plasma membrane-associated protein	0.1682	0.0582	2.89	0.1301	0.0734	1.77
P08519	APOA	LPA	Apolipoprotein(a)	0.0000	-	+/-	-	0.0007	-/+

P02647	APOA1	APOA1	Apolipoprotein A-I	0.2718	0.2030	1.34	0.1037	0.0963	1.08
P02652	APOA2	APOA2	Apolipoprotein A-II	0.5311	2.3805	0.22	0.0133	0.0045	2.94
P06727	APOA4	APOA4	Apolipoprotein A-IV	0.0440	0.0895	0.49	0.0108	-	+/-
P04114	APOB	APOB	Apolipoprotein B-100	0.0104	0.0051	2.05	0.0185	0.0163	1.14
P02654	APOC1	APOC1	Apolipoprotein C-I	0.0043	0.0036	1.18	0.0038	-	+/-
P02655	APOC2	APOC2	Apolipoprotein C-II	0.0778	0.1047	0.74	-	-	-/-
P02656	APOC3	APOC3	Apolipoprotein C-III	0.0426	0.0305	1.40	0.0111	-	+/-
P05090	APOD	APOD	Apolipoprotein D	0.0584	0.0611	0.96	0.0403	0.0816	0.49
P02649	APOE	APOE	Apolipoprotein E	0.0395	0.0188	2.10	0.0427	0.0344	1.24
P02749	APOH	APOH	Beta-2-glycoprotein 1	0.0350	0.0147	2.39	0.0666	0.0442	1.51
O14791	APOL1	APOL1	Apolipoprotein L1	0.0013	0.0007	1.76	-	-	-/-
Q9BQE5	APOL2	APOL2	Apolipoprotein L2	0.0107	0.0033	3.21	0.0011	-	+/-
O95236	APOL3	APOL3	Apolipoprotein L3	0.0006	0.0012	0.53	-	-	-/-
O95445	APOM	APOM	Apolipoprotein M	0.0027	0.0029	0.95	-	-	-/-
P07741	APT	APRT	Adenine phosphoribosyltransferase	0.0591	0.0601	0.98	0.0654	0.0868	0.75
O60306	AQR	AQR	RNA helicase aquarius	-	-	-/-	0.0018	-	+/-
P10398	ARAF	ARAF	Serine/threonine-protein kinase A-Raf	-	0.0001	-/+	0.0041	0.0028	1.47
Q96P48	ARAP1	ARAP1	Arf-GAP with Rho-GAP domain, ANK repeat and PH domain-containing protein 1	-	-	-/-	0.0029	-	+/-
Q8WZ64	ARAP2	ARAP2	Arf-GAP with Rho-GAP domain, ANK repeat and PH domain-containing protein 2	0.0001	-	+/-	-	-	-/-

Q92747	ARC1A	ARPC1A	Actin-related protein 2/3 complex subunit 1A	0.0018	0.0019	0.93	-	0.0170	-/+
O15143	ARC1B	ARPC1B	Actin-related protein 2/3 complex subunit 1B	0.0179	0.0072	2.48	0.0512	0.0321	1.60
Q8IWT0	ARCH	ZBTB8OS	Protein archease	0.0004	-	+/-	-	-	-/-
P61204	ARF3	ARF3	ADP-ribosylation factor 3	0.1009	0.0703	1.43	0.1904	0.1315	1.45
P18085	ARF4	ARF4	ADP-ribosylation factor 4	0.0725	0.0505	1.44	0.1665	0.1185	1.40
P84085	ARF5	ARF5	ADP-ribosylation factor 5	0.0089	-	+/-	0.1188	0.0892	1.33
P62330	ARF6	ARF6	ADP-ribosylation factor 6	0.0116	0.0276	0.42	0.0310	0.0321	0.97
Q8N6T3	ARFG1	ARFGAP1	ADP-ribosylation factor GTPase-activating protein 1	0.0006	0.0012	0.47	-	-	-/-
Q8N6H7	ARFG2	ARFGAP2	ADP-ribosylation factor GTPase-activating protein 2	0.0007	-	+/-	0.0131	0.0063	2.08
Q9NP61	ARFG3	ARFGAP3	ADP-ribosylation factor GTPase-activating protein 3	-	-	-/-	0.0080	-	+/-
P53367	ARFP1	ARFIP1	Arfaptin-1	0.0006	0.0016	0.39	0.0088	-	+/-
P53365	ARFP2	ARFIP2	Arfaptin-2	0.0005	-	+/-	-	-	-/-
Q13795	ARFRP	ARFRP1	ADP-ribosylation factor-related protein 1	-	0.0004	-/+	-	-	-/-
Q9HCE6	ARGAL	ARHGEF10L	Rho guanine nucleotide exchange factor 10-like protein	0.0001	0.0001	0.67	0.0055	0.0040	1.38
P05089	ARGI1	ARG1	Arginase-1	0.1379	0.2646	0.52	0.0966	0.1619	0.60
Q9NWB6	ARGL1	ARGLU1	Arginine and glutamate-rich protein 1	0.0001	-	+/-	-	-	-/-
Q5SW96	ARH	LDLRAP1	Low density lipoprotein receptor adapter protein 1	0.0005	-	+/-	-	-	-/-

Q92888	ARHG1	ARHGEF1	Rho guanine nucleotide exchange factor 1	0.0001	-	+/-	0.0044	0.0018	2.50
Q15052	ARHG6	ARHGEF6	Rho guanine nucleotide exchange factor 6	-	-	-/-	0.0022	-	+/-
Q14155	ARHG7	ARHGEF7	Rho guanine nucleotide exchange factor 7	0.0001	0.0002	0.61	0.0050	-	+/-
Q14155-1	ARHG7	ARHGEF7	Isoform 1 of Rho guanine nucleotide exchange factor 7	0.0005	-	+/-	-	-	-/-
Q9NZN5	ARHGC	ARHGEF12	Rho guanine nucleotide exchange factor 12	-	-	-/-	0.0011	-	+/-
Q96DR7	ARHGQ	ARHGEF26	Rho guanine nucleotide exchange factor 26	0.0010	-	+/-	-	-	-/-
Q9NX46	ARHL2	ADPRHL2	ADP-ribose glycohydrolase ARH3	0.0075	0.0138	0.55	-	0.0066	-/+
O95376	ARI2	ARIH2	E3 ubiquitin-protein ligase ARIH2	0.0030	0.0003	10.29	-	-	-/-
O43488	ARK72	AKR7A2	Aflatoxin B1 aldehyde reductase member 2	0.0316	0.0334	0.94	0.0265	0.0535	0.49
O95154	ARK73	AKR7A3	Aflatoxin B1 aldehyde reductase member 3	0.0950	0.1624	0.59	0.0976	0.2129	0.46
P40616	ARL1	ARL1	ADP-ribosylation factor-like protein 1	0.0048	0.0037	1.31	0.0460	0.0161	2.87
P36404	ARL2	ARL2	ADP-ribosylation factor-like protein 2	0.0050	0.0037	1.34	-	-	-/-
P36405	ARL3	ARL3	ADP-ribosylation factor-like protein 3	0.0029	0.0066	0.44	0.0093	-	+/-
Q96BM9	ARL8A	ARL8A	ADP-ribosylation factor-like protein 8A	0.0114	0.0039	2.91	0.0178	0.0083	2.13
Q9NVJ2	ARL8B	ARL8B	ADP-ribosylation factor-like protein 8B	0.0035	0.0033	1.04	0.0245	0.0125	1.95
P04424	ARLY	ASL	Argininosuccinate lyase	0.0310	0.0504	0.61	0.0513	0.0931	0.55
Q8N2F6	ARM10	ARMC10	Armadillo repeat-containing protein 10	0.0002	0.0005	0.39	-	-	-/-
Q9NVT9	ARMC1	ARMC1	Armadillo repeat-containing protein 1	0.0023	0.0036	0.64	-	-	-/-
Q6NXE6	ARMC6	ARMC6	Armadillo repeat-containing protein 6	0.0016	0.0006	2.70	-	-	-/-

Q5T2E6	ARMD3	ARMH3	Armadillo-like helical domain-containing protein 3	-	-	-/-	0.0028	-	+/-
Q9H993	ARMT1	ARMT1	Damage-control phosphatase ARMT1	0.0002	0.0005	0.33	0.0046	0.0074	0.63
Q9UH62	ARMX3	ARMCX3	Armadillo repeat-containing X-linked protein 3	0.0001	-	+/-	0.0058	-	+/-
P61160	ARP2	ACTR2	Actin-related protein 2	0.0175	0.0078	2.24	0.0877	0.0462	1.90
P61158	ARP3	ACTR3	Actin-related protein 3	0.0573	0.0312	1.84	0.1012	0.0775	1.31
Q9BPX5	ARP5L	ARPC5L	Actin-related protein 2/3 complex subunit 5-like protein	0.0142	0.0076	1.87	0.0098	0.0101	0.97
O15144	ARPC2	ARPC2	Actin-related protein 2/3 complex subunit 2	0.0689	0.0341	2.02	0.0638	0.0252	2.54
O15145	ARPC3	ARPC3	Actin-related protein 2/3 complex subunit 3	0.0206	0.0277	0.74	0.0291	0.0213	1.37
P59998	ARPC4	ARPC4	Actin-related protein 2/3 complex subunit 4	0.0502	0.0561	0.89	0.0293	0.0224	1.31
O15511	ARPC5	ARPC5	Actin-related protein 2/3 complex subunit 5	0.0308	0.0154	2.01	0.0460	0.0161	2.87
Q7Z6K5	ARPIN	ARPIN	Arpin	0.0021	0.0004	5.61	-	-	-/-
P49407	ARRB1	ARRB1	Beta-arrestin-1	-	0.0004	-/+	-	-	-/-
P15289	ARSA	ARSA	Arylsulfatase A	0.0067	0.0039	1.69	0.0354	0.0154	2.29
P15848	ARSB	ARSB	Arylsulfatase B	0.0003	-	+/-	-	-	-/-
P51690	ARSE	ARSE	Arylsulfatase E	0.0005	0.0008	0.56	0.0043	0.0168	0.26
Q6UWY0	ARSK	ARSK	Arylsulfatase K	0.0001	-	+/-	-	-	-/-
P18440	ARY1	NAT1	Arylamine N-acetyltransferase 1	-	0.0003	-/+	-	-	-/-
P11245	ARY2	NAT2	Arylamine N-acetyltransferase 2	-	0.0008	-/+	-	0.0161	-/+

Q9HBK9	AS3MT	AS3MT	Arsenite methyltransferase	0.0020	0.0041	0.48	0.0071	0.0074	0.97
Q13510	ASAH1	ASAH1	Acid ceramidase	0.0312	0.0201	1.55	0.0685	0.0321	2.13
Q96DX5	ASB9	ASB9	Ankyrin repeat and SOCS box protein 9	0.0010	0.0030	0.34	-	-	-/-
Q9ULZ3	ASC	PYCARD	Apoptosis-associated speck-like protein containing a CARD	0.0083	0.0087	0.95	0.0147	-	+/-
Q8N9N2	ASCC1	ASCC1	Activating signal cointegrator 1 complex subunit 1	-	0.0004	-/+	-	-	-/-
Q8N3C0	ASCC3	ASCC3	Activating signal cointegrator 1 complex subunit 3	0.0003	-	+/-	-	-	-/-
P07306	ASGR1	ASGR1	Asialoglycoprotein receptor 1	0.0223	0.0113	1.98	0.0711	0.0573	1.24
P07307	ASGR2	ASGR2	Asialoglycoprotein receptor 2	0.0042	0.0009	4.70	0.0254	0.0161	1.58
O95671	ASML	ASMTL	Probable bifunctional dTTP/UTP pyrophosphatase/methyltransferase protein	0.0010	0.0022	0.44	0.0059	0.0112	0.52
O43681	ASNA	ASNA1	ATPase ASNA1	0.0135	0.0074	1.84	0.0215	0.0099	2.17
Q9BZE9	ASPC1	ASPSCR1	Tether containing UBX domain for GLUT4	0.0001	0.0008	0.10	-	-	-/-
A6ND91	ASPD	ASPDH	Putative L-aspartate dehydrogenase	0.0125	0.0380	0.33	0.0088	0.0979	0.09
P20933	ASPG	AGA	N (4)-(beta-N-acetylglucosaminyl)-L-asparaginase	0.0035	0.0023	1.51	-	-	-/-
Q12797	ASPH	ASPH	Aspartyl/asparaginyl beta-hydroxylase	0.0110	0.0007	15.85	0.0330	0.0131	2.53
Q8IZT6	ASPM	ASPM	Abnormal spindle-like microcephaly-associated protein	0.0000	-	+/-	-	-	-/-
Q9BXN1	ASPN	ASPN	Asporin	0.0297	0.0076	3.94	0.0265	0.0112	2.37

P00966	ASSY	ASS1	Argininosuccinate synthase	0.0928	0.2067	0.45	0.1146	0.1937	0.59
Q8NB49	AT11C	ATP11C	Phospholipid-transporting ATPase IG	0.0001	-	+/-	-	0.0016	-/+
Q9HD20	AT131	ATP13A1	Endoplasmic reticulum transmembrane helix translocase	0.0002	0.0002	1.37	0.0108	-	+/-
P05023	AT1A1	ATP1A1	Sodium/potassium-transporting ATPase subunit alpha-1	0.0246	0.0048	5.16	0.0600	0.0396	1.51
P13637	AT1A3	ATP1A3	Sodium/potassium-transporting ATPase subunit alpha-3	0.0004	-	+/-	-	-	-/-
P05026	AT1B1	ATP1B1	Sodium/potassium-transporting ATPase subunit beta-1	0.0121	0.0063	1.91	0.0232	0.0140	1.66
O14983	AT2A1	ATP2A1	Sarcoplasmic/endoplasmic reticulum calcium ATPase 1	0.0011	0.0002	6.80	-	-	-/-
P16615	AT2A2	ATP2A2	Sarcoplasmic/endoplasmic reticulum calcium ATPase 2	0.0141	0.0021	6.71	0.0559	0.0263	2.12
Q93084	AT2A3	ATP2A3	Sarcoplasmic/endoplasmic reticulum calcium ATPase 3	0.0001	-	+/-	0.0109	-	+/-
P20020	AT2B1	ATP2B1	Plasma membrane calcium-transporting ATPase 1	0.0004	-	+/-	0.0036	0.0014	2.48
Q01814	AT2B2	ATP2B2	Plasma membrane calcium-transporting ATPase 2	0.0001	-	+/-	-	-	-/-
P23634	AT2B4	ATP2B4	Plasma membrane calcium-transporting ATPase 4	0.0006	-	+/-	0.0043	0.0022	1.98
P98194	AT2C1	ATP2C1	Calcium-transporting ATPase type 2C member 1	-	-	-/-	0.0032	-	+/-

Q8IUZ5	AT2L2	PHYKPL	5-phosphohydroxy-L-lysine phospho-lyase	0.0006	0.0064	0.09	-	0.0188	-/+
P24539	AT5F1	ATP5PB	ATP synthase F (0) complex subunit B1, mitochondrial	0.0511	0.0747	0.68	0.0810	0.1207	0.67
Q8NBU5	ATAD1	ATAD1	Outer mitochondrial transmembrane helix translocase	-	-	-/-	0.0068	0.0096	0.71
Q96QE3	ATAD5	ATAD5	ATPase family AAA domain-containing protein 5	0.0003	-	+/-	0.0009	0.0006	1.46
Q9NVI7	ATD3A	ATAD3A	ATPase family AAA domain-containing protein 3A	0.0041	0.0058	0.70	0.0031	0.0067	0.47
Q9NVI7-2	ATD3A	ATAD3A	Isoform 2 of ATPase family AAA domain-containing protein 3A	-	-	-/-	-	0.0045	-/+
Q5T9A4	ATD3B	ATAD3B	ATPase family AAA domain-containing protein 3B	0.0019	0.0022	0.87	-	-	-/-
Q9NT62	ATG3	ATG3	Ubiquitin-like-conjugating enzyme ATG3	0.0015	0.0008	1.94	0.0179	0.0103	1.74
O95352	ATG7	ATG7	Ubiquitin-like modifier-activating enzyme ATG7	-	-	-/-	0.0076	0.0030	2.55
Q9BSB4	ATGA1	ATG101	Autophagy-related protein 101	0.0004	0.0006	0.69	-	-	-/-
Q8NHH9	ATLA2	ATL2	Atlastin-2	-	-	-/-	0.0034	-	+/-
Q6DD88	ATLA3	ATL3	Atlastin-3	0.0149	0.0017	8.82	0.0354	0.0143	2.48
Q13315	ATM	ATM	Serine-protein kinase ATM	-	-	-/-	0.0005	-	+/-
O00244	ATOX1	ATOX1	Copper transport protein ATOX1	0.0041	0.0056	0.73	-	-	-/-
P56381	ATP5E	ATP5F1E	ATP synthase subunit epsilon, mitochondrial	-	0.0010	-/+	-	-	-/-

O75947	ATP5H	ATP5PD	ATP synthase subunit d, mitochondrial	0.1429	0.1367	1.05	0.0774	0.0872	0.89
P56385	ATP5I	ATP5ME	ATP synthase subunit e, mitochondrial	0.0093	0.0381	0.24	0.0344	0.0374	0.92
P18859	ATP5J	ATP5PF	ATP synthase-coupling factor 6, mitochondrial	-	0.0018	-/+	-	-	-/-
O75964	ATP5L	ATP5MG	ATP synthase subunit g, mitochondrial	0.0544	0.1160	0.47	0.0807	0.0959	0.84
Q99766	ATP5S	DMAC2L	ATP synthase subunit s, mitochondrial	0.0007	0.0060	0.11	-	-	-/-
P03928	ATP8	MT-ATP8	ATP synthase protein 8	0.0024	-	+/-	-	-	-/-
P25705	ATPA	ATP5F1A	ATP synthase subunit alpha, mitochondrial	0.3022	0.2809	1.08	0.0790	0.1142	0.69
P06576	ATPB	ATP5F1B	ATP synthase subunit beta, mitochondrial	0.7838	0.4850	1.62	0.2540	0.2880	0.88
P30049	ATPD	ATP5F1D	ATP synthase subunit delta, mitochondrial	0.0160	0.0220	0.73	0.0165	0.0284	0.58
Q5TC12	ATPF1	ATPAF1	ATP synthase mitochondrial F1 complex assembly factor 1	0.0010	0.0022	0.43	-	-	-/-
Q8N5M1	ATPF2	ATPAF2	ATP synthase mitochondrial F1 complex assembly factor 2	0.0016	0.0051	0.31	-	0.0093	-/+
P36542	ATPG	ATP5F1C	ATP synthase subunit gamma, mitochondrial	0.0562	0.0567	0.99	0.0216	0.0332	0.65
P56134-2	ATPK	ATP5MF	Isoform 2 of ATP synthase subunit f, mitochondrial	0.0029	-	+/-	-	-	-/-
P56134	ATPK	ATP5MF	ATP synthase subunit f, mitochondrial	0.0197	0.0238	0.83	0.0338	0.0338	1.00
Q96IX5	ATPMD	ATP5MD	ATP synthase membrane subunit DAPIT, mitochondrial	0.0251	0.0315	0.80	0.0460	0.0793	0.58
P48047	ATPO	ATP5PO	ATP synthase subunit O, mitochondrial	0.0326	0.1515	0.22	0.0870	0.0897	0.97
P17735	ATTY	TAT	Tyrosine aminotransferase	0.0001	0.0021	0.06	0.0073	0.0199	0.37

Q9UBB4	ATX10	ATXN10	Ataxin-10	-	-	-/-	0.0081	-	+/-
Q99700	ATX2	ATXN2	Ataxin-2	0.0001	0.0001	0.77	-	-	-/-
Q8WWM7	ATX2L	ATXN2L	Ataxin-2-like protein	0.0001	-	+/-	-	-	-/-
Q13825	AUHM	AUH	Methylglutaconyl-CoA hydratase, mitochondrial	0.0024	0.0044	0.55	-	-	-/-
Q07817	B2CL1	BCL2L1	Bcl-2-like protein 1	0.0011	-	+/-	-	-	-/-
Q9BXK5	B2L13	BCL2L13	Bcl-2-like protein 13	0.0160	0.0039	4.05	0.0114	0.0103	1.11
Q5TBC7	B2L15	BCL2L15	Bcl-2-like protein 15	0.0018	-	+/-	-	-	-/-
P61769	B2MG	B2M	Beta-2-microglobulin	0.0226	0.0060	3.77	0.1006	-	+/-
P02730	B3AT	SLC4A1	Band 3 anion transport protein	0.0218	0.0069	3.16	0.0261	0.0277	0.94
P15291	B4GT1	B4GALT1	Beta-1,4-galactosyltransferase 1	0.0002	-	+/-	0.0116	-	+/-
Q14032	BAAT	BAAT	Bile acid-CoA:amino acid N-acyltransferase	0.0263	0.0481	0.55	0.0336	0.0719	0.47
Q9NWV8	BABA1	BABAM1	BRISC and BRCA1-A complex member 1	0.0016	0.0032	0.49	-	-	-/-
Q9NXR7	BABA2	BABAM2	BRISC and BRCA1-A complex member 2	0.0010	-	+/-	0.0090	0.0060	1.50
O00154	BACH	ACOT7	Cytosolic acyl coenzyme A thioester hydrolase	-	0.0002	-/+	0.0038	-	+/-
O75531	BAF	BANF1	Barrier-to-autointegration factor	0.0194	0.0175	1.11	0.0155	0.0161	0.97
O95816	BAG2	BAG2	BAG family molecular chaperone regulator 2	0.0183	0.0035	5.27	0.0064	-	+/-
P46379	BAG6	BAG6	Large proline-rich protein BAG6	0.0005	-	+/-	0.0028	0.0045	0.63
Q9UQB8	BAIP2	BAIAP2	Brain-specific angiogenesis inhibitor 1-associated protein 2	0.0005	-	+/-	-	-	-/-

Q8IXM2	BAP18	BAP18	Chromatin complexes subunit BAP18	0.0022	0.0019	1.13	-	-	-/-
Q9UHQ4	BAP29	BCAP29	B-cell receptor-associated protein 29	0.0002	-	+/-	-	-	-/-
P51572	BAP31	BCAP31	B-cell receptor-associated protein 31	0.0166	0.0004	38.92	0.0525	0.0085	6.20
P35613	BASI	BSG	Basigin	0.0061	0.0041	1.46	0.0186	0.0193	0.97
P80723	BASP1	BASP1	Brain acid soluble protein 1	-	-	-/-	-	0.0016	-/+
Q07812	BAX	BAX	Apoptosis regulator BAX	0.0005	-	+/-	0.0219	0.0101	2.17
A8MTZ0	BBIP1	BBIP1	BBSome-interacting protein 1	-	0.0009	-/+	-	-	-/-
P50895	BCAM	BCAM	Basal cell adhesion molecule	0.0044	-	+/-	0.0071	0.0035	2.02
Q6ZUJ8	BCAP	PIK3AP1	Phosphoinositide 3-kinase adapter protein 1	0.0037	0.0004	9.69	0.0075	0.0078	0.97
O15382	BCAT2	BCAT2	Branched-chain-amino-acid aminotransferase, mitochondrial	0.0013	-	+/-	0.0075	-	+/-
Q9P287	BCCIP	BCCIP	BRCA2 and CDKN1A-interacting protein	-	0.0006	-/+	-	-	-/-
Q9BYV7	BCDO2	BCO2	Beta,beta-carotene 9',10'-oxygenase	0.0014	0.0014	1.00	-	0.0080	-/+
O14874	BCKD	BCKDK	[3-methyl-2-oxobutanoate dehydrogenase [lipoamide]] kinase, mitochondrial	0.0022	0.0067	0.34	0.0103	0.0184	0.56
Q9Y276	BCS1	BCS1L	Mitochondrial chaperone BCS1	0.0036	0.0021	1.73	0.0095	0.0127	0.75
Q8NFC6	BD1L1	BOD1L1	Biorientation of chromosomes in cell division protein 1-like 1	-	-	-/-	-	0.0005	-/+
Q02338	BDH	BDH1	D-beta-hydroxybutyrate dehydrogenase, mitochondrial	0.0765	0.1343	0.57	0.0801	0.1468	0.55
Q9BUT1	BDH2	BDH2	3-hydroxybutyrate dehydrogenase type 2	0.0121	0.0197	0.61	0.0107	0.0201	0.53

Q14457	BECN1	BECN1	Beclin-1	-	0.0009	-/+	-	-	-/-
O15155	BET1	BET1	BET1 homolog	-	0.0010	-/+	-	-	-/-
P16278	BGAL	GLB1	Beta-galactosidase	0.0117	0.0052	2.24	0.0348	0.0214	1.62
Q15582	BGH3	TGFBI	Transforming growth factor-beta-induced protein ig-h3	0.0514	0.0094	5.48	0.0524	0.0327	1.60
P08236	BGLR	GUSB	Beta-glucuronidase	0.0104	0.0045	2.28	0.0375	0.0181	2.08
Q93088	BHMT1	BHMT	Betaine--homocysteine S-methyltransferase 1	0.0678	0.2499	0.27	0.0496	0.2362	0.21
Q9H2M3	BHMT2	BHMT2	S-methylmethionine--homocysteine S-methyltransferase BHMT2	0.0250	0.0551	0.45	0.0222	0.1481	0.15
P55061	BI1	TMBIM6	Bax inhibitor 1	0.0010	-	+/-	-	-	-/-
Q9UHR4	BI2L1	BAIAP2L1	Brain-specific angiogenesis inhibitor 1-associated protein 2-like protein 1	0.0008	-	+/-	0.0029	-	+/-
P55957	BID	BID	BH3-interacting domain death agonist	0.0093	0.0063	1.47	0.0160	-	+/-
P53004	BIEA	BLVRA	Biliverdin reductase A	0.0100	0.0050	2.00	0.0109	0.0053	2.06
Q9Y6D5	BIG2	ARFGEF2	Brefeldin A-inhibited guanine nucleotide-exchange protein 2	-	-	-/-	0.0025	-	+/-
O00499	BIN1	BIN1	Myc box-dependent-interacting protein 1	0.0005	0.0041	0.13	-	-	-/-
Q9UBW5	BIN2	BIN2	Bridging integrator 2	0.0002	0.0002	0.77	0.0029	-	+/-
P11021	BIP	HSPA5	Endoplasmic reticulum chaperone BiP	0.3712	0.1609	2.31	0.1790	0.1129	1.59
P78537	BL1S1	BLOC1S1	Biogenesis of lysosome-related organelles complex 1 subunit 1	-	0.0005	-/+	-	-	-/-

Q6QNY1	BL1S2	BLOC1S2	Biogenesis of lysosome-related organelles complex 1 subunit 2	0.0004	0.0011	0.32	-	-	-/-
Q8TDH9	BL1S5	BLOC1S5	Biogenesis of lysosome-related organelles complex 1 subunit 5	0.0002	-	+/-	-	-	-/-
P51451	BLK	BLK	Tyrosine-protein kinase Blk	-	0.0002	-/+	-	-	-/-
Q13867	BLMH	BLMH	Bleomycin hydrolase	-	0.0049	-/+	0.0079	0.0039	2.02
P30043	BLVRB	BLVRB	Flavin reductase (NADPH)	0.1510	0.2212	0.68	0.1234	0.1461	0.84
O75936	BODG	BBOX1	Gamma-butyrobetaine dioxygenase	0.0012	0.0062	0.20	-	0.0170	-/+
Q9H3K6	BOLA2	BOLA2	BolA-like protein 2	0.0077	0.0095	0.81	-	-	-/-
Q53S33	BOLA3	BOLA3	BolA-like protein 3	-	0.0006	-/+	-	-	-/-
Q969J3	BORC5	BORCS5	BLOC-1-related complex subunit 5	0.0004	-	+/-	-	-	-/-
Q96B45	BORC7	BORCS7	BLOC-1-related complex subunit 7	0.0040	-	+/-	-	-	-/-
Q96FH0	BORC8	BORCS8	BLOC-1-related complex subunit 8	0.0004	-	+/-	-	-	-/-
Q86WA6	BPHL	BPHL	Valacyclovir hydrolase	0.0123	0.0227	0.54	0.0324	0.0270	1.20
P17213	BPI	BPI	Bactericidal permeability-increasing protein	0.0263	0.0010	27.70	0.0497	-	+/-
Q9NP55	BPIA1	BPIFA1	BPI fold-containing family A member 1	0.0060	0.0010	6.27	-	-	-/-
Q8TDL5	BPIB1	BPIFB1	BPI fold-containing family B member 1	0.0017	0.0003	5.38	-	-	-/-
O95861	BPNT1	BPNT1	3' (2'),5'-bisphosphate nucleotidase 1	0.0154	0.0187	0.83	0.0439	0.0232	1.90
P46736	BRCC3	BRCC3	Lys-63-specific deubiquitinase BRCC36	0.0011	0.0017	0.63	-	-	-/-
Q5VTR2	BRE1A	RNF20	E3 ubiquitin-protein ligase BRE1A	0.0001	-	+/-	-	-	-/-
Q8WUW1	BRK1	BRK1	Protein BRICK1	-	0.0031	-/+	-	-	-/-

Q5VW32	BROX	BROX	BRO1 domain-containing protein BROX	0.0009	0.0005	1.94	0.0086	-	+/-
Q96G97	BSCL2	BSCL2	Seipin	-	-	-/-	0.0069	-	+/-
Q9NW68	BSDC1	BSDC1	BSD domain-containing protein 1	0.0019	0.0009	2.00	-	-	-/-
Q10588	BST1	BST1	ADP-ribosyl cyclase/cyclic ADP-ribose hydrolase 2	0.0002	-	+/-	0.0082	-	+/-
Q10589	BST2	BST2	Bone marrow stromal antigen 2	0.0004	-	+/-	-	-	-/-
Q13410	BT1A1	BTN1A1	Butyrophilin subfamily 1 member A1	-	0.0002	-/+	-	-	-/-
O00478	BT3A3	BTN3A3	Butyrophilin subfamily 3 member A3	0.0007	0.0005	1.50	-	-	-/-
Q96K17	BT3L4	BTF3L4	Transcription factor BTF3 homolog 4	-	0.0008	-/+	-	-	-/-
P43251	BTD	BTD	Biotinidase	0.0004	-	+/-	-	-	-/-
P20290	BTF3	BTF3	Transcription factor BTF3	0.0043	0.0076	0.57	-	-	-/-
O43684	BUB3	BUB3	Mitotic checkpoint protein BUB3	0.0076	0.0077	1.00	0.0090	-	+/-
P41223	BUD31	BUD31	Protein BUD31 homolog	0.0004	0.0003	1.04	-	-	-/-
Q9UBR1	BUP1	UPB1	Beta-ureidopropionase	0.0292	0.0624	0.47	0.0064	0.1211	0.05
Q7L1Q6	BZW1	BZW1	Basic leucine zipper and W2 domain-containing protein 1	0.0029	0.0006	5.00	0.0088	0.0052	1.68
Q9Y6E2	BZW2	BZW2	Basic leucine zipper and W2 domain-containing protein 2	-	-	-/-	0.0031	-	+/-
Q99622	C10	C12orf57	Protein C10	0.0010	0.0109	0.09	-	-	-/-
Q86VB7	C163A	CD163	Scavenger receptor cysteine-rich type 1 protein M130	0.0022	0.0015	1.52	0.0150	0.0114	1.32

P02745	C1QA	C1QA	Complement C1q subcomponent subunit A	0.0017	0.0004	4.08	-	-	-/-
P02746	C1QB	C1QB	Complement C1q subcomponent subunit B	0.0120	0.0078	1.53	0.0294	0.0241	1.22
Q07021	C1QBP	C1QBP	Complement component 1 Q subcomponent-binding protein, mitochondrial	0.0323	0.0166	1.95	0.0389	0.0495	0.79
P02747	C1QC	C1QC	Complement C1q subcomponent subunit C	0.0156	0.0047	3.30	0.0258	0.0170	1.52
P00736	C1R	C1R	Complement C1r subcomponent	0.0034	0.0011	3.08	0.0190	0.0128	1.48
Q9NZP8	C1RL	C1RL	Complement C1r subcomponent-like protein	0.0002	-	+/-	-	-	-/-
P09871	C1S	C1S	Complement C1s subcomponent	0.0187	0.0084	2.21	0.0126	0.0049	2.59
P11586	C1TC	MTHFD1	C-1-tetrahydrofolate synthase, cytoplasmic	0.0663	0.1320	0.50	0.0473	0.1454	0.33
Q6UB35	C1TM	MTHFD1L	Monofunctional C1-tetrahydrofolate synthase, mitochondrial	0.0002	-	+/-	-	-	-/-
Q6P1N0	C2D1A	CC2D1A	Coiled-coil and C2 domain-containing protein 1A	0.0001	-	+/-	0.0023	0.0016	1.46
Q6DHV5	C2D2B	CC2D2B	Protein CC2D2B	-	-	-/-	-	0.0017	-/+
P04003	C4BPA	C4BPA	C4b-binding protein alpha chain	0.0375	0.0067	5.59	0.0826	0.0177	4.67
P20851	C4BPB	C4BPB	C4b-binding protein beta chain	0.0108	0.0005	21.56	-	-	-/-
Q99643	C560	SDHC	Succinate dehydrogenase cytochrome b560 subunit, mitochondrial	0.0005	0.0089	0.06	0.0671	0.0560	1.20
Q9BV19	CA050	C1orf50	Uncharacterized protein C1orf50	-	0.0011	-/+	-	-	-/-
Q9H425	CA198	C1orf198	Uncharacterized protein C1orf198	0.0002	-	+/-	-	-	-/-
P54289	CA2D1	CACNA2D1	Voltage-dependent calcium channel subunit alpha-2/delta-1	0.0007	-	+/-	0.0017	-	+/-

Q9Y376	CAB39	CAB39	Calcium-binding protein 39	0.0006	0.0013	0.44	0.0042	-	+/-
Q9BRK5	CAB45	SDF4	45 kDa calcium-binding protein	0.0053	0.0042	1.27	-	-	-/-
Q8TDN4	CABL1	CABLES1	CDK5 and ABL1 enzyme substrate 1	0.0012	-	+/-	-	-	-/-
Q9P1Z2	CACO1	CALCOCO1	Calcium-binding and coiled-coil domain-containing protein 1	-	0.0002	-/+	-	-	-/-
Q13137	CACO2	CALCOCO2	Calcium-binding and coiled-coil domain-containing protein 2	0.0009	0.0006	1.65	-	-	-/-
P43155	CACP	CRAT	Carnitine O-acetyltransferase	0.0083	0.0089	0.93	0.0210	0.0344	0.61
P55290	CAD13	CDH13	Cadherin-13	0.0006	-	+/-	-	-	-/-
P12830	CADH1	CDH1	Cadherin-1	0.0031	0.0027	1.17	0.0028	0.0060	0.47
P19022	CADH2	CDH2	Cadherin-2	0.0024	0.0024	1.00	-	0.0027	-/+
P33151	CADH5	CDH5	Cadherin-5	0.0002	-	+/-	0.0025	0.0026	0.97
Q5T440	CAF17	IBA57	Putative transferase CAF17, mitochondrial	0.0051	0.0059	0.86	0.0169	0.0080	2.12
P00915	CAH1	CA1	Carbonic anhydrase 1	0.2559	0.1792	1.43	0.1756	0.2777	0.63
P00918	CAH2	CA2	Carbonic anhydrase 2	0.0947	0.1518	0.62	0.0736	0.2136	0.34
P07451	CAH3	CA3	Carbonic anhydrase 3	0.0005	0.0008	0.69	-	-	-/-
P35218	CAH5A	CA5A	Carbonic anhydrase 5A, mitochondrial	0.0009	0.0043	0.21	-	-	-/-
P22676	CALB2	CALB2	Calretinin	0.0002	0.0006	0.41	-	-	-/-
Q05682-4	CALD1	CALD1	Isoform 4 of Caldesmon	0.0051	-	+/-	-	-	-/-
Q05682-3	CALD1	CALD1	Isoform 3 of Caldesmon	0.0011	-	+/-	0.0088	0.0043	2.03
Q05682	CALD1	CALD1	Caldesmon	0.0043	0.0003	17.00	0.0009	0.0008	1.08

Q96GE6	CALL4	CALML4	Calmodulin-like protein 4	0.0002	-	+/-	-	-	-/-
Q9NZT1	CALL5	CALML5	Calmodulin-like protein 5	0.0031	0.0224	0.14	-	-	-/-
P0DP23	CALM1	CALM1	Calmodulin-1	0.0261	0.1219	0.21	0.2033	0.0782	2.60
P27797	CALR	CALR	Calreticulin	0.1731	0.1108	1.56	0.1275	0.0980	1.30
O43852	CALU	CALU	Calumenin	0.0296	0.0134	2.21	0.0078	-	+/-
O43852-5	CALU	CALU	Isoform 5 of Calumenin	0.0085	0.0053	1.60	-	-	-/-
O43852-2	CALU	CALU	Isoform 2 of Calumenin	0.0115	0.0024	4.87	-	-	-/-
P27824	CALX	CANX	Calnexin	0.0842	0.0380	2.21	0.0879	0.0576	1.53
P49913	CAMP	CAMP	Cathelicidin antimicrobial peptide	0.0016	0.0005	3.36	0.0254	-	+/-
P07384	CAN1	CAPN1	Calpain-1 catalytic subunit	0.0053	0.0045	1.18	0.0413	0.0350	1.18
P17655	CAN2	CAPN2	Calpain-2 catalytic subunit	0.0004	0.0003	1.07	0.0212	0.0151	1.40
O15484	CAN5	CAPN5	Calpain-5	-	-	-/-	0.0024	0.0038	0.64
P63098	CANB1	PPP3R1	Calcineurin subunit B type 1	0.0048	0.0083	0.58	-	-	-/-
Q86VP6-2	CAND1	CAND1	Isoform 2 of Cullin-associated NEDD8-dissociated protein 1	0.0003	-	+/-	-	-	-/-
Q86VP6	CAND1	CAND1	Cullin-associated NEDD8-dissociated protein 1	0.0034	0.0033	1.03	0.0182	0.0259	0.70
Q01518	CAP1	CAP1	Adenylyl cyclase-associated protein 1	0.0584	0.0240	2.44	0.0721	0.0471	1.53
P40123	CAP2	CAP2	Adenylyl cyclase-associated protein 2	0.0013	-	+/-	0.0040	-	+/-
P20160	CAP7	AZU1	Azurocidin	0.0014	0.0007	1.99	0.0414	-	+/-
P40121	CAPG	CAPG	Macrophage-capping protein	0.0341	0.0011	31.22	0.0322	-	+/-

Q14444	CAPR1	CAPRIN1	Caprin-1	0.0033	0.0030	1.11	0.0050	-	+/-
P47756-2	CAPZB	CAPZB	Isoform 2 of F-actin-capping protein subunit beta	0.0579	0.0375	1.54	0.0788	0.0551	1.43
P47756	CAPZB	CAPZB	F-actin-capping protein subunit beta	0.0454	0.0177	2.56	0.0093	0.0560	0.17
Q5EG05	CAR16	CARD16	Caspase recruitment domain-containing protein 16	-	0.0021	-/+	-	-	-/-
Q96LW7-2	CAR19	CARD19	Isoform 2 of Caspase recruitment domain-containing protein 19	0.0004	0.0020	0.21	-	-	-/-
Q9NXV6	CARF	CDKN2AIP	CDKN2A-interacting protein	0.0004	-	+/-	-	-	-/-
Q6F5E8	CARL2	CARMIL2	Capping protein, Arp2/3 and myosin-I linker protein 2	0.0000	-	+/-	-	-	-/-
Q86X55	CARM1	CARM1	Histone-arginine methyltransferase CARM1	-	-	-/-	0.0046	-	+/-
P29466	CASP1	CASP1	Caspase-1	0.0023	0.0025	0.91	0.0065	-	+/-
P42574	CASP3	CASP3	Caspase-3	0.0011	0.0008	1.27	0.0054	-	+/-
P55212	CASP6	CASP6	Caspase-6	0.0006	0.0014	0.46	-	-	-/-
P55210	CASP7	CASP7	Caspase-7	0.0003	-	+/-	-	-	-/-
Q14790	CASP8	CASP8	Caspase-8	-	-	-/-	0.0033	-	+/-
P31944	CASPE	CASP14	Caspase-14	0.0010	0.0107	0.09	-	-	-/-
P04040	CATA	CAT	Catalase	0.1265	0.1868	0.68	0.1234	0.2186	0.56
P07858	CATB	CTSB	Cathepsin B	0.1431	0.1258	1.14	0.1556	0.1215	1.28
P53634	CATC	CTSC	Dipeptidyl peptidase 1	0.0089	0.0042	2.14	0.0383	0.0049	7.82

P07339	CATD	CTSD	Cathepsin D	0.2723	0.2115	1.29	0.2325	0.1946	1.19
Q9UBX1	CATF	CTSF	Cathepsin F	0.0003	0.0006	0.55	-	-	-/-
P08311	CATG	CTSG	Cathepsin G	0.0687	0.0048	14.28	0.0653	0.0148	4.43
P09668	CATH	CTSH	Pro-cathepsin H	0.0076	0.0036	2.15	0.0100	-	+/-
P07711	CATL1	CTSL	Cathepsin L1	0.0015	0.0007	2.11	-	-	-/-
P25774	CATS	CTSS	Cathepsin S	0.0109	0.0026	4.16	0.0383	0.0076	5.07
Q9UBR2	CATZ	CTSZ	Cathepsin Z	0.0260	0.0119	2.18	0.0371	0.0263	1.41
Q03135	CAV1	CAV1	Caveolin-1	0.0012	0.0031	0.39	0.0124	0.0128	0.97
Q6NZI2	CAVN1	CAVIN1	Caveolae-associated protein 1	0.0187	0.0162	1.15	0.0160	0.0142	1.12
O95810	CAVN2	CAVIN2	Caveolae-associated protein 2	0.0002	0.0041	0.06	-	-	-/-
Q969G5	CAVN3	CAVIN3	Caveolae-associated protein 3	0.0022	0.0012	1.87	-	-	-/-
Q5BKX8	CAVN4	CAVIN4	Caveolae-associated protein 4	0.0002	0.0003	0.65	-	0.0028	-/+
Q13938	CAYP1	CAPS	Calcyphosin	0.0004	0.0011	0.41	-	-	-/-
P52907	CAZA1	CAPZA1	F-actin-capping protein subunit alpha-1	0.0835	0.0264	3.16	0.0573	0.0509	1.13
P47755	CAZA2	CAPZA2	F-actin-capping protein subunit alpha-2	0.0451	0.0269	1.68	0.0500	0.0346	1.44
Q68DN1	CB016	C2orf16	Uncharacterized protein C2orf16	-	-	-/-	0.0007	-	+/-
A6NCS6	CB072	C2orf72	Uncharacterized protein C2orf72	0.0011	0.0023	0.48	0.0124	0.0093	1.34
Q3KRA6	CB076	C2orf76	UPF0538 protein C2orf76	0.0008	0.0007	1.11	-	-	-/-
Q9H9S4	CB39L	CAB39L	Calcium-binding protein 39-like	-	-	-/-	0.0039	-	+/-
P08185	CBG	SERPINA6	Corticosteroid-binding globulin	0.0015	-	+/-	0.0116	-	+/-

P15088	CBPA3	CPA3	Mast cell carboxypeptidase A	0.0002	0.0026	0.09	0.0032	0.0051	0.63
Q96IY4	CBPB2	CPB2	Carboxypeptidase B2	0.0011	-	+/-	0.0071	-	+/-
O75976	CBPD	CPD	Carboxypeptidase D	0.0027	-	+/-	0.0039	-	+/-
P14384	CBPM	CPM	Carboxypeptidase M	0.0009	-	+/-	-	-	-/-
P15169	CBPN	CPN1	Carboxypeptidase N catalytic chain	0.0010	-	+/-	-	-	-/-
Q9Y646	CBPQ	CPQ	Carboxypeptidase Q	0.0050	0.0028	1.81	0.0151	0.0047	3.24
P16152	CBR1	CBR1	Carbonyl reductase [NADPH] 1	0.1359	0.2341	0.58	0.1153	0.1575	0.73
O75828	CBR3	CBR3	Carbonyl reductase [NADPH] 3	0.0029	0.0049	0.58	0.0137	-	+/-
Q8N4T8	CBR4	CBR4	Carbonyl reductase family member 4	0.0098	0.0186	0.52	0.0143	0.0293	0.49
P35520	CBS	CBS	Cystathionine beta-synthase	0.0024	0.0032	0.74	0.0101	0.0314	0.32
P0DN79	CBSL	CBSL	Cystathionine beta-synthase-like protein	0.0034	0.0095	0.36	-	0.0038	-/+
P83916	CBX1	CBX1	Chromobox protein homolog 1	0.0100	0.0099	1.01	-	-	-/-
Q13185	CBX3	CBX3	Chromobox protein homolog 3	0.0242	0.0120	2.01	0.0093	0.0062	1.50
P45973	CBX5	CBX5	Chromobox protein homolog 5	0.0007	0.0014	0.50	-	-	-/-
Q9HC52	CBX8	CBX8	Chromobox protein homolog 8	0.0001	-	+/-	-	-	-/-
Q8IYK2	CC105	CCDC105	Coiled-coil domain-containing protein 105	-	0.0001	-/+	-	-	-/-
Q96NT0	CC115	CCDC115	Coiled-coil domain-containing protein 115	0.0007	-	+/-	-	-	-/-
Q96CT7	CC124	CCDC124	Coiled-coil domain-containing protein 124	0.0006	0.0003	2.01	0.0038	-	+/-
Q9H6E4	CC134	CCDC134	Coiled-coil domain-containing protein 134	0.0005	0.0003	1.50	-	-	-/-

Q9GZT6	CC90B	CCDC90B	Coiled-coil domain-containing protein 90B, mitochondrial	0.0004	0.0029	0.12	-	-	-/-
Q8IX12	CCAR1	CCAR1	Cell division cycle and apoptosis regulator protein 1	0.0001	-	+/-	0.0010	-	+/-
Q8N163	CCAR2	CCAR2	Cell cycle and apoptosis regulator protein 2	0.0009	0.0006	1.44	0.0051	0.0053	0.97
O60826	CCD22	CCDC22	Coiled-coil domain-containing protein 22	0.0005	0.0009	0.61	0.0047	0.0048	0.97
Q86WR0	CCD25	CCDC25	Coiled-coil domain-containing protein 25	0.0008	0.0038	0.22	-	-	-/-
Q4G0X9	CCD40	CCDC40	Coiled-coil domain-containing protein 40	0.0000	-	+/-	-	-	-/-
Q96MW1	CCD43	CCDC43	Coiled-coil domain-containing protein 43	0.0001	0.0004	0.41	-	-	-/-
Q96A33	CCD47	CCDC47	PAT complex subunit CCDC47	0.0008	0.0010	0.81	0.0105	0.0065	1.61
Q96ER9	CCD51	CCDC51	Coiled-coil domain-containing protein 51	0.0017	0.0021	0.82	-	-	-/-
Q4VC31	CCD58	CCDC58	Coiled-coil domain-containing protein 58	0.0015	0.0097	0.16	-	-	-/-
Q76M96	CCD80	CCDC80	Coiled-coil domain-containing protein 80	-	-	-/-	0.0013	-	+/-
Q567U6	CCD93	CCDC93	Coiled-coil domain-containing protein 93	0.0001	-	+/-	-	-	-/-
P53701	CCHL	HCCS	Cytochrome c-type heme lyase	0.0040	0.0013	3.15	-	-	-/-
Q9BSQ5	CCM2	CCM2	Cerebral cavernous malformations 2 protein	0.0010	-	+/-	-	-	-/-
Q9ULG6	CCPG1	CCPG1	Cell cycle progression protein 1	-	-	-/-	0.0017	-	+/-
O14618	CCS	CCS	Copper chaperone for superoxide dismutase	0.0143	0.0119	1.21	-	0.0144	-/+
P86790	CCZ1B	CCZ1B	Vacuolar fusion protein CCZ1 homolog B	-	-	-/-	0.0034	-	+/-
P21127	CD11B	CDK11B	Cyclin-dependent kinase 11B	-	-	-/-	0.0013	-	+/-
P08571	CD14	CD14	Monocyte differentiation antigen CD14	0.0104	0.0031	3.39	0.0265	0.0158	1.68

Q13740	CD166	ALCAM	CD166 antigen	0.0044	0.0007	6.40	0.0031	-	+/-
Q9Y5K6	CD2AP	CD2AP	CD2-associated protein	0.0007	0.0005	1.35	-	-	-/-
Q8IX05	CD302	CD302	CD302 antigen	-	-	-/-	0.0175	-	+/-
P16671	CD36	CD36	Platelet glycoprotein 4	0.0001	-	+/-	-	0.0068	-/+
Q7L3B6	CD37L	CDC37L1	Hsp90 co-chaperone Cdc37-like 1	-	0.0004	-/+	-	-	-/-
Q08722	CD47	CD47	Leukocyte surface antigen CD47	0.0024	-	+/-	-	-	-/-
P13987	CD59	CD59	CD59 glycoprotein	0.0091	0.0045	2.02	0.0521	0.0410	1.27
O43866	CD5L	CD5L	CD5 antigen-like	0.0016	0.0021	0.79	-	-	-/-
P08962	CD63	CD63	CD63 antigen	0.0023	-	+/-	0.0103	-	+/-
P60033	CD81	CD81	CD81 antigen	0.0110	0.0021	5.18	0.0506	0.0618	0.82
P27701	CD82	CD82	CD82 antigen	-	-	-/-	0.0109	0.0113	0.97
P21926	CD9	CD9	CD9 antigen	-	-	-/-	0.0147	0.0152	0.97
P48960	CD97	CD97	CD97 antigen	0.0011	0.0006	1.87	-	-	-/-
Q16543	CDC37	CDC37	Hsp90 co-chaperone Cdc37	0.0016	0.0022	0.72	0.0173	0.0045	3.81
P60953	CDC42	CDC42	Cell division control protein 42 homolog	0.0291	0.0259	1.13	0.0532	0.0579	0.92
Q99459	CDC5L	CDC5L	Cell division cycle 5-like protein	-	0.0002	-/+	-	-	-/-
Q6P1J9	CDC73	CDC73	Parafibromin	0.0008	0.0002	4.49	-	-	-/-
P32320	CDD	CDA	Cytidine deaminase	0.0053	0.0058	0.92	-	-	-/-
Q9HBB8	CDHR5	CDHR5	Cadherin-related family member 5	0.0004	-	+/-	-	-	-/-

O14735	CDIPT	CDIPT	CDP-diacylglycerol--inositol 3-phosphatidyltransferase	0.0005	0.0008	0.67	0.0254	-	+/-
P24941	CDK2	CDK2	Cyclin-dependent kinase 2	-	0.0009	-/+	0.0056	-	+/-
Q00535	CDK5	CDK5	Cyclin-dependent-like kinase 5	0.0009	0.0015	0.61	0.0054	-	+/-
P42773	CDN2C	CDKN2C	Cyclin-dependent kinase 4 inhibitor C	0.0008	-	+/-	-	-	-/-
Q49AH0	CDNF	CDNF	Cerebral dopamine neurotrophic factor	-	0.0016	-/+	-	-	-/-
Q16878	CDO1	CDO1	Cysteine dioxygenase type 1	0.0008	0.0019	0.42	-	-	-/-
O95674	CDS2	CDS2	Phosphatidate cytidylyltransferase 2	0.0002	0.0003	0.67	0.0054	-	+/-
Q15517	CDSN	CDSN	Corneodesmosin	0.0033	0.0196	0.17	-	-	-/-
Q9UKY7	CDV3	CDV3	Protein CDV3 homolog	0.0007	-	+/-	-	-	-/-
O94986	CE152	CEP152	Centrosomal protein of 152 kDa	0.0000	-	+/-	-	-	-/-
P13688	CEAM1	CEACAM1	Carcinoembryonic antigen-related cell adhesion molecule 1	0.0017	-	+/-	0.0175	-	+/-
P40199	CEAM6	CEACAM6	Carcinoembryonic antigen-related cell adhesion molecule 6	0.0022	-	+/-	-	-	-/-
P31997	CEAM8	CEACAM8	Carcinoembryonic antigen-related cell adhesion molecule 8	0.0016	-	+/-	-	-	-/-
Q92879	CELF1	CELF1	CUGBP Elav-like family member 1	0.0001	0.0010	0.14	0.0082	0.0055	1.50
O95319	CELF2	CELF2	CUGBP Elav-like family member 2	-	0.0004	-/+	0.0073	-	+/-
Q02224	CENPE	CENPE	Centromere-associated protein E	-	0.0000	-/+	-	-	-/-
P49454	CENPF	CENPF	Centromere protein F	-	-	-/-	0.0006	0.0009	0.72

Q7Z7K6	CENPV	CENPV	Centromere protein V	0.0014	0.0014	0.94	-	0.0049	-/+
Q9C0F1	CEP44	CEP44	Centrosomal protein of 44 kDa	-	0.0005	-/+	-	-	-/-
Q9Y6K0	CEPT1	CEPT1	Choline/ethanolaminephosphotransferase 1	-	-	-/-	-	0.0071	-/+
Q96G23	CERS2	CERS2	Ceramide synthase 2	0.0038	0.0009	4.28	0.0217	0.0104	2.09
Q9Y5P4	CERT	CERT	Ceramide transfer protein	-	0.0016	-/+	-	-	-/-
P00450	CERU	CP	Ceruloplasmin	0.0344	0.0110	3.14	0.0439	0.0278	1.58
P41208	CETN2	CETN2	Centrin-2	0.0006	0.0012	0.46	-	-	-/-
O15182	CETN3	CETN3	Centrin-3	0.0009	-	+/-	-	-	-/-
Q7Z4R8	CF120	C6orf120	UPF0669 protein C6orf120	0.0022	0.0021	1.02	-	-	-/-
Q9Y6A4	CFA20	CFAP20	Cilia- and flagella-associated protein 20	0.0009	0.0013	0.66	0.0066	-	+/-
P00751	CFAB	CFB	Complement factor B	0.0279	0.0041	6.81	0.0273	0.0111	2.46
P00746	CFAD	CFD	Complement factor D	0.0035	0.0022	1.59	-	-	-/-
P08603	CFAH	CFH	Complement factor H	0.0262	0.0049	5.38	0.0794	0.0266	2.98
P05156	CFAI	CFI	Complement factor I	0.0041	0.0013	3.22	0.0056	0.0043	1.29
Q9BPX7	CG025	C7orf25	UPF0415 protein C7orf25	0.0001	-	+/-	-	-	-/-
Q9UFW8	CGBP1	CGGBP1	CGG triplet repeat-binding protein 1	0.0008	-	+/-	-	-	-/-
P32929	CGL	CTH	Cystathionine gamma-lyase	0.0208	0.0697	0.30	0.0492	0.1241	0.40
Q0VF96	CGNL1	CGNL1	Cingulin-like protein 1	0.0008	0.0002	3.79	-	0.0010	-/+
Q6P1X6	CH082	C8orf82	UPF0598 protein C8orf82	0.0088	0.0155	0.56	0.0209	0.0259	0.81
P61604	CH10	HSPE1	10 kDa heat shock protein, mitochondrial	0.0556	0.0771	0.72	0.0382	0.0353	1.08

P36222	CH3L1	CHI3L1	Chitinase-3-like protein 1	0.0016	-	+/-	0.0226	-	+/-
P10809	CH60	HSPD1	60 kDa heat shock protein, mitochondrial	0.3615	0.2595	1.39	0.1085	0.1552	0.70
Q9BSY4	CHCH5	CHCHD5	Coiled-coil-helix-coiled-coil-helix domain-containing protein 5	0.0006	-	+/-	-	-	-/-
Q14839	CHD4	CHD4	Chromodomain-helicase-DNA-binding protein 4	0.0001	-	+/-	0.0017	-	+/-
Q8TD26	CHD6	CHD6	Chromodomain-helicase-DNA-binding protein 6	0.0000	-	+/-	-	-	-/-
Q8NE62	CHDH	CHDH	Choline dehydrogenase, mitochondrial	0.0167	0.0103	1.62	0.0449	0.0432	1.04
Q8IWX8	CHERP	CHERP	Calcium homeostasis endoplasmic reticulum protein	0.0001	-	+/-	-	-	-/-
Q9BWS9	CHID1	CHID1	Chitinase domain-containing protein 1	0.0005	0.0018	0.28	0.0159	0.0138	1.16
P52757	CHIO	CHN2	Beta-chimaerin	0.0003	-	+/-	-	-	-/-
Q9UNE7	CHIP	STUB1	E3 ubiquitin-protein ligase CHIP	0.0012	0.0016	0.72	-	-	-/-
Q13231	CHIT1	CHIT1	Chitotriosidase-1	0.0007	-	+/-	0.0093	-	+/-
P06276	CHLE	BCHE	Cholinesterase	0.0003	-	+/-	-	-	-/-
Q9HD42	CHM1A	CHMP1A	Charged multivesicular body protein 1a	0.0008	-	+/-	-	-	-/-
Q7LBR1	CHM1B	CHMP1B	Charged multivesicular body protein 1b	0.0009	0.0011	0.81	-	-	-/-
O43633	CHM2A	CHMP2A	Charged multivesicular body protein 2a	0.0006	0.0014	0.43	-	-	-/-
Q9UQN3	CHM2B	CHMP2B	Charged multivesicular body protein 2b	0.0008	-	+/-	-	-	-/-
Q9BY43	CHM4A	CHMP4A	Charged multivesicular body protein 4a	0.0002	0.0004	0.41	-	-	-/-

Q9Y3E7	CHMP3	CHMP3	Charged multivesicular body protein 3	0.0009	-	+/-	-	-	-/-
Q9NZZ3	CHMP5	CHMP5	Charged multivesicular body protein 5	0.0002	0.0004	0.41	-	-	-/-
Q8WUX9	CHMP7	CHMP7	Charged multivesicular body protein 7	0.0004	0.0002	2.40	-	-	-/-
Q99653	CHP1	CHP1	Calcineurin B homologous protein 1	0.0224	0.0148	1.51	-	-	-/-
Q96BS2	CHP3	TESC	Calcineurin B homologous protein 3	0.0018	-	+/-	-	-	-/-
Q9NRG0	CHRC1	CHRAC1	Chromatin accessibility complex protein 1	-	0.0008	-/+	-	-	-/-
Q9UHD1	CHRD1	CHORDC1	Cysteine and histidine-rich domain-containing protein 1	0.0007	0.0002	2.93	-	-	-/-
Q9Y2V2	CHSP1	CARHSP1	Calcium-regulated heat-stable protein 1	0.0108	0.0235	0.46	-	-	-/-
Q8WUH1	CHUR	CHURC1	Protein Churchill	0.0004	0.0016	0.28	-	-	-/-
Q9H5X1	CIA2A	CIAO2A	Cytosolic iron-sulfur assembly component 2A	0.0015	0.0056	0.26	0.0266	-	+/-
Q9Y3D0	CIA2B	CIAO2B	Cytosolic iron-sulfur assembly component 2B	0.0028	0.0080	0.36	-	-	-/-
Q9Y375	CIA30	NDUFAF1	Complex I intermediate-associated protein 30, mitochondrial	0.0044	0.0033	1.34	-	-	-/-
O76071	CIAO1	CIAO1	Probable cytosolic iron-sulfur protein assembly protein CIAO1	0.0030	0.0060	0.50	0.0112	0.0074	1.51
Q99828	CIB1	CIB1	Calcium and integrin-binding protein 1	0.0018	0.0026	0.69	-	-	-/-
Q9P2M7	CING	CGN	Cingulin	0.0006	-	+/-	-	-	-/-
Q14011	CIRBP	CIRBP	Cold-inducible RNA-binding protein	0.0047	0.0101	0.46	0.0155	0.0074	2.11
Q9NZ45	CISD1	CISD1	CDGSH iron-sulfur domain-containing protein 1	0.0155	0.0307	0.50	0.0213	0.0275	0.77

Q8N5K1	CISD2	CISD2	CDGSH iron-sulfur domain-containing protein 2	0.0072	0.0177	0.41	0.0175	0.0174	1.01
P0C7P0	CISD3	CISD3	CDGSH iron-sulfur domain-containing protein 3, mitochondrial	0.0002	-	+/-	-	0.0091	-/+
O75390	CISY	CS	Citrate synthase, mitochondrial	0.0259	0.0167	1.56	0.0317	0.0275	1.15
Q9H0W9	CK054	C11orf54	Ester hydrolase C11orf54	0.0150	0.0291	0.51	0.0261	0.0340	0.77
Q9H3H3	CK068	C11orf68	UPF0696 protein C11orf68	0.0008	0.0004	2.01	-	-	-/-
Q96JB5	CK5P3	CDK5RAP3	CDK5 regulatory subunit-associated protein 3	0.0040	0.0030	1.31	0.0009	0.0042	0.22
Q07065	CKAP4	CKAP4	Cytoskeleton-associated protein 4	0.0587	0.0103	5.69	0.0564	0.0146	3.86
Q14008	CKAP5	CKAP5	Cytoskeleton-associated protein 5	0.0001	-	+/-	0.0010	-	+/-
O75122	CLAP2	CLASP2	CLIP-associating protein 2	-	-	-/-	0.0012	-	+/-
Q9H2X3	CLC4M	CLEC4M	C-type lectin domain family 4 member M	-	-	-/-	-	0.0047	-/+
P09496	CLCA	CLTA	Clathrin light chain A	0.0026	0.0009	2.83	-	-	-/-
P09497	CLCB	CLTB	Clathrin light chain B	0.0052	-	+/-	-	-	-/-
Q00610	CLH1	CLTC	Clathrin heavy chain 1	0.0596	0.0151	3.96	0.1139	0.0738	1.54
P53675	CLH2	CLTCL1	Clathrin heavy chain 2	0.0004	-	+/-	-	-	-/-
O00299	CLIC1	CLIC1	Chloride intracellular channel protein 1	0.1139	0.0362	3.14	0.0769	0.0675	1.14
O15247	CLIC2	CLIC2	Chloride intracellular channel protein 2	0.0009	0.0063	0.13	-	-	-/-
Q9Y696	CLIC4	CLIC4	Chloride intracellular channel protein 4	0.0249	0.0181	1.38	0.0173	0.0179	0.97
Q9NZA1	CLIC5	CLIC5	Chloride intracellular channel protein 5	0.0006	0.0003	2.00	-	-	-/-
P30622	CLIP1	CLIP1	CAP-Gly domain-containing linker protein 1	0.0001	-	+/-	-	-	-/-

Q96JQ2	CLMN	CLMN	Calmin	-	-	-/-	-	0.0029	-/+
O75503	CLN5	CLN5	Ceroid-lipofuscinosis neuronal protein 5	-	-	-/-	0.0056	-	+/-
Q9NWW5	CLN6	CLN6	Ceroid-lipofuscinosis neuronal protein 6	-	-	-/-	0.0147	-	+/-
Q96KA5	CLP1L	CLPTM1L	Cleft lip and palate transmembrane protein 1-like protein	0.0001	-	+/-	0.0076	0.0037	2.02
Q9H078	CLPB	CLPB	Caseinolytic peptidase B protein homolog	0.0015	0.0006	2.54	0.0028	0.0029	0.97
Q16740	CLPP	CLPP	ATP-dependent Clp protease proteolytic subunit, mitochondrial	0.0062	0.0134	0.46	0.0081	0.0083	0.97
O96005	CLPT1	CLPTM1	Cleft lip and palate transmembrane protein 1	0.0001	-	+/-	0.0103	0.0050	2.05
O76031	CLPX	CLPX	ATP-dependent Clp protease ATP-binding subunit clpX-like, mitochondrial	0.0013	0.0014	0.95	0.0059	0.0075	0.79
O75153	CLU	CLUH	Clustered mitochondria protein homolog	0.0000	0.0001	0.33	0.0065	0.0059	1.10
P10909	CLUS	CLU	Clusterin	0.0538	0.0283	1.90	0.0154	0.0137	1.12
P10909-3	CLUS	CLU	Isoform 3 of Clusterin	-	-	-/-	0.0155	-	+/-
Q8N0X4	CLYBL	CLYBL	Citramalyl-CoA lyase, mitochondrial	0.0177	0.0312	0.57	0.0143	0.0524	0.27
P23946	CMA1	CMA1	Chymase	0.0011	0.0030	0.36	-	0.0083	-/+
Q96DG6	CMBL	CMBL	Carboxymethylenebutenolidase homolog	0.0498	0.1343	0.37	0.0388	0.1455	0.27
O75746	CMC1	SLC25A12	Calcium-binding mitochondrial carrier protein Aralar1	0.0016	0.0002	8.36	-	-	-/-
Q9UJS0	CMC2	SLC25A13	Calcium-binding mitochondrial carrier protein Aralar2	0.1927	0.0673	2.86	0.1039	0.0939	1.11

Q86VU5	CMTD1	COMTD1	Catechol O-methyltransferase domain-containing protein 1	0.0018	0.0042	0.43	-	0.0143	-/+
P09543	CN37	CNP	2',3'-cyclic-nucleotide 3'-phosphodiesterase	0.0031	0.0042	0.74	0.0114	0.0081	1.40
P62633	CNBP	CNBP	Cellular nucleic acid-binding protein	0.0015	0.0009	1.63	-	-	-/-
Q9BPX3	CND3	NCAPG	Condensin complex subunit 3	0.0000	-	+/-	-	-	-/-
Q96KN2	CNDP1	CNDP1	Beta-Ala-His dipeptidase	0.0003	-	+/-	-	-	-/-
Q96KP4-2	CNDP2	CNDP2	Isoform 2 of Cytosolic non-specific dipeptidase	-	-	-/-	-	0.0095	-/+
Q96KP4	CNDP2	CNDP2	Cytosolic non-specific dipeptidase	0.0669	0.0788	0.85	0.0797	0.1212	0.66
Q9P003	CNIH4	CNIH4	Protein cornichon homolog 4	0.0009	-	+/-	0.0266	-	+/-
P51911	CNN1	CNN1	Calponin-1	0.0023	0.0003	8.25	-	-	-/-
Q99439	CNN2	CNN2	Calponin-2	0.0087	0.0042	2.09	0.0179	0.0103	1.74
Q15417	CNN3	CNN3	Calponin-3	0.0134	0.0057	2.35	0.0173	-	+/-
A5YKK6	CNOT1	CNOT1	CCR4-NOT transcription complex subunit 1	-	-	-/-	0.0022	0.0009	2.46
Q9UIV1	CNOT7	CNOT7	CCR4-NOT transcription complex subunit 7	0.0003	-	+/-	-	-	-/-
Q92600	CNOT9	CNOT9	CCR4-NOT transcription complex subunit 9	0.0002	-	+/-	-	-	-/-
Q9Y2B0	CNPY2	CNPY2	Protein canopy homolog 2	0.0565	0.0355	1.59	0.0498	0.0118	4.22
Q9BT09	CNPY3	CNPY3	Protein canopy homolog 3	0.0008	0.0013	0.65	0.0050	-	+/-
Q96F85	CNRP1	CNRIP1	CB1 cannabinoid receptor-interacting protein 1	0.0042	0.0031	1.35	0.0109	-	+/-
P02452	CO1A1	COL1A1	Collagen alpha-1 (I) chain	0.0032	0.0009	3.45	0.0143	0.0127	1.13

P08123	CO1A2	COL1A2	Collagen alpha-2 (I) chain	0.0064	0.0015	4.30	0.0178	0.0154	1.16
P06681	CO2	C2	Complement C2	0.0014	-	+/-	0.0131	0.0027	4.87
P01024	CO3	C3	Complement C3	0.0990	0.0398	2.49	0.0845	0.0600	1.41
P02461	CO3A1	COL3A1	Collagen alpha-1(III) chain	0.0008	-	+/-	0.0016	0.0004	3.74
P0C0L4	CO4A	C4A	Complement C4-A	0.0626	0.0208	3.01	0.0569	0.0333	1.71
P02462	CO4A1	COL4A1	Collagen alpha-1(IV) chain	0.0002	-	+/-	0.0100	0.0059	1.69
P08572	CO4A2	COL4A2	Collagen alpha-2 (IV) chain	0.0019	0.0004	4.71	0.0096	0.0059	1.62
P0C0L5	CO4B	C4B	Complement C4-B	0.0251	0.0033	7.59	0.0574	0.0345	1.66
P01031	CO5	C5	Complement C5	0.0024	0.0002	14.58	0.0063	0.0055	1.14
P20908	CO5A1	COL5A1	Collagen alpha-1 (V) chain	0.0003	-	+/-	-	-	-/-
P05997	CO5A2	COL5A2	Collagen alpha-2(V) chain	0.0002	-	+/-	0.0004	-	+/-
P13671	CO6	C6	Complement component C6	0.0030	0.0003	10.44	0.0044	0.0026	1.65
P12109	CO6A1	COL6A1	Collagen alpha-1 (VI) chain	0.0914	0.0563	1.63	0.0489	0.0448	1.09
P12110-2	CO6A2	COL6A2	Isoform 2C2A of Collagen alpha-2 (VI) chain	0.0090	0.0112	0.80	-	0.0228	-/+
P12110	CO6A2	COL6A2	Collagen alpha-1 (VI) chain	0.0649	0.0333	1.95	0.0322	0.0249	1.29
P12111-4	CO6A3	COL6A3	Isoform 4 of Collagen alpha-3 (VI) chain	0.0188	0.0049	3.81	0.0092	-	+/-
P12111-3	CO6A3	COL6A3	Isoform 3 of Collagen alpha-3(VI) chain	0.0034	-	+/-	-	-	-/-
P12111-2	CO6A3	COL6A3	Isoform 2 of Collagen alpha-3(VI) chain	0.0000	0.0001	0.51	-	-	-/-
P12111	CO6A3	COL6A3	Collagen alpha-3 (VI) chain	0.1379	0.0689	2.00	0.0468	0.0448	1.04
A6NMZ7	CO6A6	COL6A6	Collagen alpha-6 (VI) chain	0.0004	0.0006	0.66	-	0.0015	-/+

P10643	CO7	C7	Complement component C7	0.0043	0.0008	5.34	0.0083	0.0047	1.76
P07357	CO8A	C8A	Complement component C8 alpha chain	0.0059	0.0013	4.73	0.0059	0.0061	0.97
P07358	CO8B	C8B	Complement component C8 beta chain	0.0049	0.0003	16.09	0.0060	0.0041	1.47
P07360	CO8G	C8G	Complement component C8 gamma chain	0.0167	0.0108	1.55	0.0200	-	+/-
P02748	CO9	C9	Complement component C9	0.0147	0.0054	2.71	0.0134	0.0128	1.05
Q9Y2R0	COA3	COA3	Cytochrome c oxidase assembly factor 3 homolog, mitochondrial	0.0057	0.0015	3.81	-	-	-/-
Q96BR5	COA7	COA7	Cytochrome c oxidase assembly factor 7	0.0008	-	+/-	-	-	-/-
Q96CD2	COAC	PPCDC	Phosphopantothenoylcysteine decarboxylase	0.0006	0.0022	0.30	-	-	-/-
Q13057	COASY	COASY	Bifunctional coenzyme A synthase	0.0016	0.0027	0.59	0.0076	0.0123	0.62
Q53SF7	COBL1	COBLL1	Cordon-bleu protein-like 1	0.0004	-	+/-	-	0.0016	-/+
Q99715	COCA1	COL12A1	Collagen alpha-1 (XII) chain	0.0009	-	+/-	0.0054	0.0010	5.43
Q05707	COEA1	COL14A1	Collagen alpha-1 (XIV) chain	0.0752	0.0141	5.32	0.0645	0.0268	2.41
P23528	COF1	CFL1	Cofilin-1	0.1604	0.0998	1.61	0.1293	0.0789	1.64
Q9Y281	COF2	CFL2	Cofilin-2	0.0246	0.0407	0.60	0.0544	0.0657	0.83
P39059	COFA1	COL15A1	Collagen alpha-1(XV) chain	0.0013	-	+/-	0.0054	-	+/-
Q8WTW3	COG1	COG1	Conserved oligomeric Golgi complex subunit 1	-	-	-/-	0.0028	-	+/-
Q96JB2	COG3	COG3	Conserved oligomeric Golgi complex subunit 3	-	-	-/-	0.0024	-	+/-

Q9Y2V7	COG6	COG6	Conserved oligomeric Golgi complex subunit 6	-	-	-/-	0.0048	-	+/-
P83436	COG7	COG7	Conserved oligomeric Golgi complex subunit 7	0.0001	-	+/-	0.0028	-	+/-
Q96MW5	COG8	COG8	Conserved oligomeric Golgi complex subunit 8	-	-	-/-	0.0034	-	+/-
P39060	COIA1	COL18A1	Collagen alpha-1 (XVIII) chain	0.0054	0.0044	1.23	0.0100	0.0083	1.21
Q9BWP8	COL11	COLEC11	Collectin-11	0.0018	0.0019	0.94	-	-	-/-
Q8N668	COMD1	COMMD1	COMM domain-containing protein 1	0.0043	0.0030	1.42	-	-	-/-
Q86X83	COMD2	COMMD2	COMM domain-containing protein 2	0.0025	0.0034	0.76	-	-	-/-
Q9UBI1	COMD3	COMMD3	COMM domain-containing protein 3	0.0034	0.0076	0.44	0.0098	0.0101	0.97
Q9H0A8	COMD4	COMMD4	COMM domain-containing protein 4	0.0005	0.0046	0.12	-	-	-/-
Q9GZQ3	COMD5	COMMD5	COMM domain-containing protein 5	0.0031	0.0061	0.51	-	-	-/-
Q7Z4G1	COMD6	COMMD6	COMM domain-containing protein 6	0.0059	0.0144	0.41	-	-	-/-
Q86VX2	COMD7	COMMD7	COMM domain-containing protein 7	0.0012	0.0025	0.48	-	-	-/-
Q9NX08	COMD8	COMMD8	COMM domain-containing protein 8	0.0014	0.0038	0.36	-	-	-/-
Q9P000	COMD9	COMMD9	COMM domain-containing protein 9	0.0010	0.0121	0.08	-	-	-/-
Q9Y6G5	COMDA	COMMD10	COMM domain-containing protein 10	0.0029	0.0063	0.46	-	-	-/-
P21964	COMT	COMT	Catechol O-methyltransferase	0.0613	0.2556	0.24	0.0656	0.1946	0.34
Q17RW2	COOA1	COL24A1	Collagen alpha-1(XXIV) chain	-	0.0001	-/+	-	-	-/-
P53621	COPA	COPA	Coatomer subunit alpha	0.0139	0.0060	2.30	0.0534	0.0336	1.59

P53618	COPB	COPB1	Coatomer subunit beta	0.0068	0.0040	1.69	0.0478	0.0321	1.49
P35606	COPB2	COPB2	Coatomer subunit beta'	0.0093	0.0028	3.31	0.0393	0.0268	1.47
P48444	COPD	ARCN1	Coatomer subunit delta	0.0196	0.0061	3.20	0.0280	0.0175	1.59
O14579	COPE	COPE	Coatomer subunit epsilon	0.0463	0.0373	1.24	0.0272	0.0143	1.91
Q9Y678	COPG1	COPG1	Coatomer subunit gamma-1	0.0114	0.0045	2.55	0.0361	0.0310	1.17
O15431	COPT1	SLC31A1	High affinity copper uptake protein 1	-	0.0012	-/+	0.0233	-	+/-
P61923	COPZ1	COPZ1	Coatomer subunit zeta-1	0.0019	0.0055	0.34	0.0266	0.0193	1.38
Q9P299	COPZ2	COPZ2	Coatomer subunit zeta-2	0.0004	0.0014	0.26	-	-	-/-
Q9NZJ6	COQ3	COQ3	Ubiquinone biosynthesis O-methyltransferase, mitochondrial	0.0010	0.0034	0.28	-	0.0062	-/+
Q9Y3A0	COQ4	COQ4	Ubiquinone biosynthesis protein COQ4 homolog, mitochondrial	0.0003	0.0004	0.77	-	-	-/-
Q5HYK3	COQ5	COQ5	2-methoxy-6-polyprenyl-1,4-benzoquinol methylase, mitochondrial	0.0127	0.0117	1.08	0.0077	0.0211	0.37
Q9Y2Z9	COQ6	COQ6	Ubiquinone biosynthesis monooxygenase COQ6, mitochondrial	0.0016	0.0039	0.40	-	0.0078	-/+
Q99807	COQ7	COQ7	5-demethoxyubiquinone hydroxylase, mitochondrial	0.0173	0.0106	1.64	-	0.0115	-/+
Q8NI60	COQ8A	COQ8A	Atypical kinase COQ8A, mitochondrial	0.0013	0.0041	0.32	0.0083	0.0041	2.03
O75208	COQ9	COQ9	Ubiquinone biosynthesis protein COQ9, mitochondrial	0.0179	0.0160	1.12	0.0103	0.0148	0.70
P31146	COR1A	CORO1A	Coronin-1A	0.0390	0.0074	5.29	0.0433	0.0156	2.78

Q9BR76	COR1B	CORO1B	Coronin-1B	0.0075	0.0067	1.11	0.0155	0.0115	1.35
Q9ULV4	COR1C	CORO1C	Coronin-1C	0.0045	0.0009	4.76	0.0221	0.0170	1.30
P57737	CORO7	CORO7	Coronin-7	0.0015	0.0010	1.48	0.0096	0.0023	4.20
Q14019	COTL1	COTL1	Coactosin-like protein	0.0420	0.0257	1.63	0.0484	0.0301	1.61
Q9Y6N1	COX11	COX11	Cytochrome c oxidase assembly protein COX11, mitochondrial	0.0003	0.0006	0.50	-	-	-/-
Q14061	COX17	COX17	Cytochrome c oxidase copper chaperone	-	0.0014	-/+	-	-	-/-
P00403	COX2	MT-CO2	Cytochrome c oxidase subunit 2	0.0560	0.0844	0.66	0.1240	0.1999	0.62
Q5RI15	COX20	COX20	Cytochrome c oxidase assembly protein COX20, mitochondrial	0.0007	0.0015	0.48	0.0116	0.0120	0.97
P13073	COX41	COX4I1	Cytochrome c oxidase subunit 4 isoform 1, mitochondrial	0.0508	0.0456	1.11	0.0530	0.0444	1.19
P20674	COX5A	COX5A	Cytochrome c oxidase subunit 5A, mitochondrial	0.1058	0.0381	2.78	0.0167	0.0029	5.71
P10606	COX5B	COX5B	Cytochrome c oxidase subunit 5B, mitochondrial	0.0163	0.0185	0.89	0.0153	0.0207	0.74
P09669	COX6C	COX6C	Cytochrome c oxidase subunit 6C	0.0046	0.0223	0.21	0.0266	0.0510	0.52
P15954	COX7C	COX7C	Cytochrome c oxidase subunit 7C, mitochondrial	0.0059	-	+/-	-	-	-/-
O14548	COX7R	COX7A2L	Cytochrome c oxidase subunit 7A-related protein, mitochondrial	0.0091	0.0049	1.87	-	-	-/-
Q7Z7K0	COXM1	CMC1	COX assembly mitochondrial protein homolog	-	0.0005	-/+	-	-	-/-

Q9NRP2	COXM2	CMC2	COX assembly mitochondrial protein 2 homolog	0.0004	-	+/-	-	-	-/-
P05177	CP1A2	CYP1A2	Cytochrome P450 1A2	0.0087	0.0197	0.44	0.0105	0.0525	0.20
Q6UW02	CP20A	CYP20A1	Cytochrome P450 20A1	-	-	-/-	0.0082	0.0085	0.97
Q02318	CP27A	CYP27A1	Sterol 26-hydroxylase, mitochondrial	0.0206	0.0139	1.48	0.0561	0.0477	1.18
P11509	CP2A6	CYP2A6	Cytochrome P450 2A6	0.0218	0.0600	0.36	0.0471	0.1282	0.37
P20853	CP2A7	CYP2A7	Cytochrome P450 2A7	-	0.0022	-/+	-	-	-/-
P20813	CP2B6	CYP2B6	Cytochrome P450 2B6	0.0001	0.0018	0.06	0.0077	0.0480	0.16
P10632	CP2C8	CYP2C8	Cytochrome P450 2C8	0.0144	0.0271	0.53	0.0367	0.0841	0.44
P11712	CP2C9	CYP2C9	Cytochrome P450 2C9	0.0265	0.0589	0.45	0.0574	0.0965	0.60
P33260	CP2CI	CYP2C18	Cytochrome P450 2C18	-	-	-/-	-	0.0164	-/+
P33261	CP2CJ	CYP2C19	Cytochrome P450 2C19	0.0014	0.0011	1.26	-	-	-/-
P10635	CP2D6	CYP2D6	Cytochrome P450 2D6	0.0058	0.0080	0.72	0.0402	0.0547	0.73
P05181	CP2E1	CYP2E1	Cytochrome P450 2E1	0.0131	0.0280	0.47	0.0413	0.0742	0.56
P51589	CP2J2	CYP2J2	Cytochrome P450 2J2	-	-	-/-	0.0054	0.0077	0.71
P08684	CP3A4	CYP3A4	Cytochrome P450 3A4	0.0110	0.0329	0.34	0.0283	0.0842	0.34
P20815	CP3A5	CYP3A5	Cytochrome P450 3A5	0.0001	-	+/-	0.0066	0.0034	1.96
P24462	CP3A7	CYP3A7	Cytochrome P450 3A7	-	-	-/-	0.0138	-	+/-
Q02928	CP4AB	CYP4A11	Cytochrome P450 4A11	0.0085	0.0414	0.21	0.0143	0.0865	0.17
P78329	CP4F2	CYP4F2	Cytochrome P450 4F2	0.0145	0.0115	1.25	0.0382	0.0509	0.75

Q08477	CP4F3	CYP4F3	Cytochrome P450 4F3	0.0065	0.0037	1.77	-	-	-/-
Q08477-2	CP4F3	CYP4F3	Isoform CYP4F3B of Cytochrome P450 4F3	0.0009	0.0049	0.18	0.0317	0.0540	0.59
P98187	CP4F8	CYP4F8	Cytochrome P450 4F8	-	-	-/-	0.0047	-	+/-
Q9HBI6	CP4FB	CYP4F11	Cytochrome P450 4F11	0.0182	0.0017	10.78	0.0657	0.0379	1.73
Q9HCS2	CP4FC	CYP4F12	Cytochrome P450 4F12	-	0.0006	-/+	-	0.0224	-/+
Q6ZWL3	CP4V2	CYP4V2	Cytochrome P450 4V2	-	0.0004	-/+	-	0.0101	-/+
Q16850	CP51A	CYP51A1	Lanosterol 14-alpha demethylase	-	0.0007	-/+	0.0094	0.0115	0.81
O75881	CP7B1	CYP7B1	Cytochrome P450 7B1	-	-	-/-	-	0.0032	-/+
Q9UNU6	CP8B1	CYP8B1	7-alpha-hydroxycholest-4-en-3-one 12-alpha-hydroxylase	0.0013	0.0041	0.31	-	0.0352	-/+
Q6FI81	CPIN1	CIAPIN1	Anamorsin	0.0018	0.0017	1.06	-	-	-/-
P22792	CPN2	CPN2	Carboxypeptidase N subunit 2	0.0025	0.0026	0.98	0.0143	0.0068	2.09
Q99829	CPNE1	CPNE1	Copine-1	0.0028	0.0017	1.66	0.0075	0.0091	0.82
Q96FN4	CPNE2	CPNE2	Copine-2	-	-	-/-	0.0058	-	+/-
O75131	CPNE3	CPNE3	Copine-3	0.0108	0.0064	1.69	0.0453	0.0294	1.54
P04632	CPNS1	CAPNS1	Calpain small subunit 1	0.0542	0.0359	1.51	0.1201	0.0563	2.13
Q9BRF8	CPPED	CPPED1	Serine/threonine-protein phosphatase CPPED1	0.0061	0.0061	1.01	0.0200	0.0207	0.97
Q10570	CPSF1	CPSF1	Cleavage and polyadenylation specificity factor subunit 1	0.0002	0.0001	2.31	-	-	-/-

Q9P2I0	CPSF2	CPSF2	Cleavage and polyadenylation specificity factor subunit 2	0.0004	0.0007	0.52	0.0032	-	+/-
Q9UKF6	CPSF3	CPSF3	Cleavage and polyadenylation specificity factor subunit 3	0.0001	-	+/-	-	-	-/-
O43809	CPSF5	NUDT21	Cleavage and polyadenylation specificity factor subunit 5	0.0144	0.0121	1.19	0.0294	0.0184	1.60
Q16630	CPSF6	CPSF6	Cleavage and polyadenylation specificity factor subunit 6	0.0006	-	+/-	0.0047	0.0032	1.48
Q8N684	CPSF7	CPSF7	Cleavage and polyadenylation specificity factor subunit 7	0.0007	-	+/-	-	-	-/-
P31327	CPSM	CPS1	Carbamoyl-phosphate synthase [ammonia], mitochondrial	0.2351	0.5028	0.47	0.1747	0.5641	0.31
P50416	CPT1A	CPT1A	Carnitine O-palmitoyltransferase 1, liver isoform	0.0047	0.0011	4.29	0.0196	0.0184	1.06
P23786	CPT2	CPT2	Carnitine O-palmitoyltransferase 2, mitochondrial	0.0165	0.0323	0.51	0.0249	0.0518	0.48
Q9H3G5	CPVL	CPVL	Probable serine carboxypeptidase CPVL	0.0042	0.0057	0.73	0.0069	0.0072	0.97
Q96MF6	CQ10A	COQ10A	Coenzyme Q-binding protein COQ10 homolog A, mitochondrial	0.0005	0.0010	0.48	-	-	-/-
Q9H8M1	CQ10B	COQ10B	Coenzyme Q-binding protein COQ10 homolog B, mitochondrial	0.0008	0.0012	0.69	-	-	-/-
Q9NQ79	CRAC1	CRTAC1	Cartilage acidic protein 1	0.0006	0.0005	1.29	-	-	-/-
P78560	CRADD	CRADD	Death domain-containing protein CRADD	0.0070	0.0096	0.73	-	-	-/-

P53674	CRBB1	CRYBB1	Beta-crystallin B1	-	0.0006	-/+	-	-	-/-
Q96SW2	CRBN	CRBN	Protein cereblon	-	-	-/-	0.0043	-	+/-
O75629	CREG1	CREG1	Protein CREG1	0.0009	-	+/-	0.0175	-	+/-
P52943	CRIP2	CRIP2	Cysteine-rich protein 2	0.0010	0.0008	1.27	0.0074	-	+/-
P46108	CRK	CRK	Adapter molecule crk	0.0142	0.0162	0.88	-	-	-/-
P46108-2	CRK	CRK	Isoform Crk-I of Adapter molecule crk	0.0035	0.0143	0.25	-	-	-/-
P46109	CRKL	CRKL	Crk-like protein	0.0083	0.0109	0.76	0.0062	-	+/-
Q8IUI8	CRLF3	CRLF3	Cytokine receptor-like factor 3	0.0002	0.0008	0.18	-	-	-/-
Q9UBG3	CRNN	CRNN	Cornulin	-	0.0004	-/+	-	-	-/-
P02741	CRP	CRP	C-reactive protein	0.0575	0.0016	35.27	0.0357	-	+/-
O75718	CRTAP	CRTAP	Cartilage-associated protein	-	0.0002	-/+	0.0040	-	+/-
P02511	CRYAB	CRYAB	Alpha-crystallin B chain	0.0091	0.0121	0.75	-	-	-/-
Q9Y2S2	CRYL1	CRYL1	Lambda-crystallin homolog	0.0335	0.0664	0.50	0.0589	0.1140	0.52
Q14894	CRYM	CRYM	Ketimine reductase mu-crystallin	0.0164	0.0219	0.75	0.0133	0.0087	1.52
Q9NSK7	CS012	C19orf12	Protein C19orf12	-	0.0016	-/+	-	-	-/-
O94886	CSCL1	TMEM63A	CSC1-like protein 1	0.0014	-	+/-	0.0059	0.0029	2.00
O75534	CSDE1	CSDE1	Cold shock domain-containing protein E1	0.0013	0.0009	1.49	0.0070	0.0040	1.75
P41240	CSK	CSK	Tyrosine-protein kinase CSK	0.0011	-	+/-	0.0114	0.0036	3.16
P68400	CSK21	CSNK2A1	Casein kinase II subunit alpha	0.0026	0.0023	1.13	0.0136	0.0042	3.21
P19784	CSK22	CSNK2A2	Casein kinase II subunit alpha'	0.0004	0.0009	0.43	0.0084	-	+/-

P67870	CSK2B	CSNK2B	Casein kinase II subunit beta	0.0061	0.0068	0.90	0.0124	-	+/-
O14936	CSKP	CASK	Peripheral plasma membrane protein CASK	-	-	-/-	0.0018	0.0027	0.64
Q13098	CSN1	GPS1	COP9 signalosome complex subunit 1	0.0001	-	+/-	0.0049	0.0050	0.97
P61201	CSN2	COPS2	COP9 signalosome complex subunit 2	0.0005	0.0018	0.27	0.0089	-	+/-
Q9UNS2	CSN3	COPS3	COP9 signalosome complex subunit 3	0.0007	-	+/-	0.0053	0.0055	0.97
Q9BT78	CSN4	COPS4	COP9 signalosome complex subunit 4	0.0093	0.0057	1.63	0.0068	0.0045	1.49
Q92905	CSN5	COPS5	COP9 signalosome complex subunit 5	0.0043	0.0041	1.05	0.0090	-	+/-
Q7L5N1	CSN6	COPS6	COP9 signalosome complex subunit 6	0.0032	0.0061	0.52	0.0112	-	+/-
Q9UBW8	CSN7A	COPS7A	COP9 signalosome complex subunit 7a	0.0023	0.0022	1.07	0.0056	0.0090	0.62
Q99627	CSN8	COPS8	COP9 signalosome complex subunit 8	0.0074	0.0133	0.56	-	0.0275	-/+
P13611	CSPG2	VCAN	Versican core protein	0.0017	-	+/-	0.0044	-	+/-
P21291	CSRP1	CSRP1	Cysteine and glycine-rich protein 1	0.0160	0.0140	1.14	0.0343	0.0275	1.24
Q16527	CSRP2	CSRP2	Cysteine and glycine-rich protein 2	0.0031	0.0023	1.30	0.0077	-	+/-
Q05048	CSTF1	CSTF1	Cleavage stimulation factor subunit 1	0.0004	0.0009	0.47	-	-	-/-
Q12996	CSTF3	CSTF3	Cleavage stimulation factor subunit 3	-	-	-/-	0.0018	-	+/-
Q9GZN8	CT027	C20orf27	UPF0687 protein C20orf27	0.0028	0.0022	1.30	-	-	-/-
Q5JQC4	CT47A	CT47A1	Cancer/testis antigen 47A	-	-	-/-	0.0023	-	+/-
Q13363	CTBP1	CTBP1	C-terminal-binding protein 1	0.0047	0.0055	0.86	0.0049	0.0107	0.46
P56545	CTBP2	CTBP2	C-terminal-binding protein 2	-	0.0004	-/+	-	-	-/-

Q96CG8	CTHR1	CTHRC1	Collagen triple helix repeat-containing protein 1	0.0005	-	+/-	-	-	-/-
Q8WWI5	CTL1	SLC44A1	Choline transporter-like protein 1	-	-	-/-	-	0.0035	-/+
P35221	CTNA1	CTNNA1	Catenin alpha-1	0.0130	0.0129	1.01	0.0109	0.0267	0.41
P26232	CTNA2	CTNNA2	Catenin alpha-2	0.0001	-	+/-	-	-	-/-
P35222	CTNB1	CTNNB1	Catenin beta-1	0.0053	0.0028	1.92	0.0252	0.0361	0.70
O60716	CTND1	CTNND1	Catenin delta-1	0.0042	0.0039	1.10	0.0097	0.0117	0.83
Q13616	CUL1	CUL1	Cullin-1	0.0001	-	+/-	0.0027	0.0028	0.97
Q13617	CUL2	CUL2	Cullin-2	0.0004	-	+/-	0.0031	-	+/-
Q13618	CUL3	CUL3	Cullin-3	0.0002	-	+/-	0.0051	0.0035	1.48
Q13619	CUL4A	CUL4A	Cullin-4A	-	-	-/-	0.0046	0.0018	2.50
Q93034	CUL5	CUL5	Cullin-5	0.0002	-	+/-	0.0042	0.0017	2.49
O60888	CUTA	CUTA	Protein CutA	0.0232	0.0357	0.65	0.0254	0.0263	0.97
Q9NTM9	CUTC	CUTC	Copper homeostasis protein cutC homolog	0.0003	0.0005	0.50	-	-	-/-
Q6UX04	CWC27	CWC27	Spliceosome-associated protein CWC27 homolog	-	-	-/-	0.0035	-	+/-
Q8TB03	CX038	CXorf38	Uncharacterized protein CXorf38	-	0.0003	-/+	-	-	-/-
P12074	CX6A1	COX6A1	Cytochrome c oxidase subunit 6A1, mitochondrial	-	0.0050	-/+	-	-	-/-
P14854	CX6B1	COX6B1	Cytochrome c oxidase subunit 6B1	0.0123	0.0093	1.32	0.0230	0.0237	0.97

P14406	CX7A2	COX7A2	Cytochrome c oxidase subunit 7A2, mitochondrial	0.0223	0.0206	1.08	0.0238	0.0273	0.87
P08034	CXB1	GJB1	Gap junction beta-1 protein	-	-	-/-	0.0064	-	+/-
P02775	CXCL7	PPBP	Platelet basic protein	0.0013	-	+/-	-	-	-/-
P08574	CY1	CYC1	Cytochrome c1, heme protein, mitochondrial	0.0418	0.0364	1.15	0.0323	0.0381	0.85
P04839	CY24B	CYBB	Cytochrome b-245 heavy chain	0.0014	-	+/-	0.0233	0.0087	2.67
P00167	CYB5	CYB5A	Cytochrome b5	0.3826	0.4057	0.94	0.1769	0.1813	0.98
O43169	CYB5B	CYB5B	Cytochrome b5 type B	0.0184	0.0159	1.16	0.0233	0.0476	0.49
Q9BQA9	CYBC1	CYBC1	Cytochrome b-245 chaperone 1	-	-	-/-	0.0103	-	+/-
Q9HB71	CYBP	CACYBP	Calcyclin-binding protein	0.0086	0.0051	1.68	0.0077	-	+/-
P99999	CYC	CYCS	Cytochrome c	0.0046	0.0074	0.62	0.0255	0.0254	1.01
Q7L576	CYFP1	CYFIP1	Cytoplasmic FMR1-interacting protein 1	0.0005	-	+/-	0.0098	0.0048	2.05
Q8WWM9	CYGB	CYGB	Cytoglobin	0.0013	0.0016	0.81	-	-	-/-
Q15438	CYH1	CYTH1	Cytohesin-1	-	-	-/-	0.0033	-	+/-
P01040	CYTA	CSTA	Cystatin-A	0.0996	0.1782	0.56	-	-	-/-
P04080	CYTB	CSTB	Cystatin-B	0.0841	0.0789	1.07	0.0348	0.0360	0.97
P01034	CYTC	CST3	Cystatin-C	0.0008	0.0046	0.18	-	-	-/-
Q9NWV4	CZIB	CZIB	CXXC motif containing zinc binding protein	0.0143	0.0221	0.65	-	-	-/-
Q2PZI1	D19L1	DPY19L1	Probable C-mannosyltransferase DPY19L1	-	-	-/-	0.0031	-	+/-
Q8N465	D2HDH	D2HGDH	D-2-hydroxyglutarate dehydrogenase, mitochondrial	-	0.0002	-/+	-	0.0087	-/+

Q9NRG7	D39U1	SDR39U1	Epimerase family protein SDR39U1	0.0036	0.0113	0.32	-	-	-/-
Q86T65	DAAM2	DAAM2	Disheveled-associated activator of morphogenesis 2	-	-	-/-	0.0012	-	+/-
P98082	DAB2	DAB2	Disabled homolog 2	0.0009	0.0002	4.53	0.0028	-	+/-
P61803	DAD1	DAD1	Dolichyl-diphosphooligosaccharide--protein glycosyltransferase subunit DAD1	0.0458	0.0122	3.74	0.0671	0.0465	1.44
Q14118	DAG1	DAG1	Dystroglycan	0.0001	-	+/-	-	-	-/-
Q96EP5	DAZP1	DAZAP1	DAZ-associated protein 1	0.0013	0.0012	1.06	0.0100	0.0103	0.97
Q9NR28	DBLOH	DIABLO	Diablo homolog, mitochondrial	0.0218	0.0197	1.11	0.0074	0.0168	0.44
Q9UJU6-2	DBNL	DBNL	Isoform 2 of Drebrin-like protein	0.0004	0.0002	1.79	-	-	-/-
Q9UJU6	DBNL	DBNL	Drebrin-like protein	0.0124	0.0118	1.05	0.0103	0.0040	2.59
Q9UJU6-6	DBNL	DBNL	Isoform 6 of Drebrin-like protein	-	-	-/-	-	0.0044	-/+
Q9UJU6-4	DBNL	DBNL	Isoform 4 of Drebrin-like protein	-	-	-/-	-	0.0050	-/+
Q13409-2	DC1I2	DYNC1I2	Isoform 2B of Cytoplasmic dynein 1 intermediate chain 2	0.0009	-	+/-	-	-	-/-
Q13409	DC1I2	DYNC1I2	Cytoplasmic dynein 1 intermediate chain 2	0.0050	0.0019	2.58	0.0058	0.0044	1.31
Q9Y6G9	DC1L1	DYNC1LI1	Cytoplasmic dynein 1 light intermediate chain 1	0.0023	0.0009	2.54	0.0033	-	+/-
O43237	DC1L2	DYNC1LI2	Cytoplasmic dynein 1 light intermediate chain 2	0.0013	0.0006	2.12	0.0034	-	+/-
Q8TEB1	DCA11	DCAF11	DDB1- and CUL4-associated factor 11	0.0002	0.0011	0.14	-	0.0045	-/+
Q9Y4B6	DCAF1	DCAF1	DDB1- and CUL4-associated factor 1	0.0001	-	+/-	-	-	-/-

P61962	DCAF7	DCAF7	DDB1- and CUL4-associated factor 7	0.0004	0.0020	0.21	0.0066	0.0068	0.97
Q5TAQ9	DCAF8	DCAF8	DDB1- and CUL4-associated factor 8	0.0003	0.0013	0.25	0.0026	-	+/-
Q8WVC6	DCAKD	DCAKD	Dephospho-CoA kinase domain-containing protein	-	0.0010	-/+	-	-	-/-
P81605	DCD	DCD	Dermcidin	0.1885	0.1612	1.17	-	-	-/-
P27707	DCK	DCK	Deoxycytidine kinase	-	0.0011	-/+	-	-	-/-
O95822	DCMC	MLYCD	Malonyl-CoA decarboxylase, mitochondrial	0.0033	0.0068	0.49	0.0088	0.0252	0.35
Q96GG9	DCNL1	DCUN1D1	DCN1-like protein 1	0.0016	0.0015	1.07	0.0058	0.0093	0.62
Q96C86	DCPS	DCPS	m7GpppX diphosphatase	0.0022	0.0029	0.75	0.0103	0.0050	2.05
P32321	DCTD	DCTD	Deoxycytidylate deaminase	0.0018	0.0112	0.16	0.0093	0.0096	0.97
Q14203	DCTN1	DCTN1	Dynactin subunit 1	0.0016	0.0018	0.86	0.0060	0.0063	0.97
Q13561	DCTN2	DCTN2	Dynactin subunit 2	0.0177	0.0144	1.23	0.0113	0.0091	1.24
O75935	DCTN3	DCTN3	Dynactin subunit 3	0.0173	0.0037	4.61	-	-	-/-
Q9UJW0	DCTN4	DCTN4	Dynactin subunit 4	0.0005	0.0002	2.82	0.0032	0.0051	0.63
Q9BTE1	DCTN5	DCTN5	Dynactin subunit 5	-	0.0009	-/+	-	-	-/-
O00399	DCTN6	DCTN6	Dynactin subunit 6	0.0026	0.0032	0.83	-	-	-/-
P06132	DCUP	UROD	Uroporphyrinogen decarboxylase	0.0023	0.0030	0.77	0.0084	0.0120	0.70
Q7Z4W1	DCXR	DCXR	L-xylulose reductase	0.3205	0.7437	0.43	0.1034	0.6929	0.15
Q9NUU7	DD19A	DDX19A	ATP-dependent RNA helicase DDX19A	0.0007	0.0016	0.44	0.0047	0.0049	0.97
O94760	DDAH1	DDAH1	N (G),N (G)-dimethylarginine dimethylaminohydrolase 1	0.0303	0.0503	0.60	0.0096	0.0428	0.22

O95865	DDAH2	DDAH2	N (G),N (G)-dimethylarginine dimethylaminohydrolase 2	0.0274	0.0154	1.79	0.0196	-	+/-
Q16531	DDB1	DDB1	DNA damage-binding protein 1	0.0063	0.0058	1.08	0.0281	0.0257	1.09
P20711	DDC	DDC	Aromatic-L-amino-acid decarboxylase	-	-	-/-	-	0.0037	-/+
Q5TDH0	DDI2	DDI2	Protein DDI1 homolog 2	0.0054	0.0096	0.57	0.0075	0.0078	0.97
Q96HY6	DDRGK	DDRGK1	DDRGK domain-containing protein 1	0.0008	0.0007	1.13	0.0079	0.0041	1.95
Q92499	DDX1	DDX1	ATP-dependent RNA helicase DDX1	0.0059	0.0026	2.31	0.0102	0.0080	1.28
Q92841	DDX17	DDX17	Probable ATP-dependent RNA helicase DDX17	0.0118	0.0086	1.37	0.0381	0.0279	1.36
Q9NVP1	DDX18	DDX18	ATP-dependent RNA helicase DDX18	0.0001	-	+/-	0.0047	0.0029	1.65
Q9NR30	DDX21	DDX21	Nucleolar RNA helicase 2	0.0001	-	+/-	0.0021	-	+/-
Q9BUQ8	DDX23	DDX23	Probable ATP-dependent RNA helicase DDX23	-	-	-/-	0.0016	-	+/-
Q9GZR7	DDX24	DDX24	ATP-dependent RNA helicase DDX24	0.0002	-	+/-	-	-	-/-
O00571	DDX3X	DDX3X	ATP-dependent RNA helicase DDX3X	0.0074	0.0028	2.61	0.0232	0.0141	1.65
O15523	DDX3Y	DDX3Y	ATP-dependent RNA helicase DDX3Y	-	-	-/-	-	0.0115	-/+
Q9UJV9	DDX41	DDX41	Probable ATP-dependent RNA helicase DDX41	0.0002	-	+/-	-	-	-/-
Q86XP3	DDX42	DDX42	ATP-dependent RNA helicase DDX42	0.0007	0.0002	2.99	0.0032	0.0016	1.97
Q7L014	DDX46	DDX46	Probable ATP-dependent RNA helicase DDX46	0.0002	-	+/-	0.0038	0.0010	4.00

Q9H0S4	DDX47	DDX47	Probable ATP-dependent RNA helicase DDX47	-	0.0002	-/+	-	-	-/-
P17844	DDX5	DDX5	Probable ATP-dependent RNA helicase DDX5	0.0110	0.0072	1.53	0.0341	0.0245	1.39
O95786	DDX58	DDX58	Antiviral innate immune response receptor RIG-I	-	-	-/-	0.0042	-	+/-
P26196	DDX6	DDX6	Probable ATP-dependent RNA helicase DDX6	0.0024	0.0025	0.95	0.0186	0.0120	1.55
Q8IY21	DDX60	DDX60	Probable ATP-dependent RNA helicase DDX60	-	-	-/-	0.0017	-	+/-
Q16698	DECR	DECR1	2,4-dienoyl-CoA reductase, mitochondrial	0.1268	0.2255	0.56	0.1148	0.2358	0.49
Q9NUI1	DECR2	DECR2	Peroxisomal 2,4-dienoyl-CoA reductase	0.0513	0.0322	1.59	0.0963	0.0293	3.28
P59665	DEF1	DEFA1	Neutrophil defensin 1	0.0083	0.0165	0.50	0.0564	0.0296	1.91
P12838	DEF4	DEFA4	Neutrophil defensin 4	-	-	-/-	0.0186	-	+/-
Q9HBH1	DEFM	PDF	Peptide deformylase, mitochondrial	0.0011	0.0004	2.91	-	0.0071	-/+
P35659	DEK	DEK	Protein DEK	0.0001	0.0002	0.77	0.0024	0.0025	0.97
Q8TCE6	DEN10	DENND10	DENN domain-containing protein 10	0.0005	0.0017	0.28	-	-	-/-
Q9H6A0	DEN2D	DENND2D	DENN domain-containing protein 2D	0.0001	-	+/-	-	-	-/-
O43583	DENR	DENR	Density-regulated protein	0.0024	0.0042	0.56	-	-	-/-
Q9Y315	DEOC	DERA	Deoxyribose-phosphate aldolase	0.0041	0.0140	0.29	0.0198	0.0241	0.82
Q9BUN8	DERL1	DERL1	Derlin-1	0.0022	-	+/-	0.0293	0.0193	1.52
Q07507	DERM	DPT	Dermatopontin	0.0294	0.0104	2.83	0.0566	0.0161	3.51

Q6ICB0	DESI1	DESI1	Desumoylating isopeptidase 1	0.0027	0.0050	0.55	-	-	-/-
Q9BSY9	DESI2	DESI2	Deubiquitinase DESI2	-	0.0011	-/+	-	-	-/-
P17661	DESM	DES	Desmin	0.0053	0.0059	0.89	0.0112	-	+/-
P15924	DESP	DSP	Desmoplakin	0.0046	0.0179	0.26	0.0028	0.0026	1.09
P60981	DEST	DSTN	Destrin	0.0199	0.0202	0.98	0.0271	0.0176	1.54
O00273	DFFA	DFFA	DNA fragmentation factor subunit alpha	0.0015	0.0041	0.37	-	-	-/-
O75907	DGAT1	DGAT1	Diacylglycerol O-acyltransferase 1	-	-	-/-	-	0.0042	-/+
P23743	DGKA	DGKA	Diacylglycerol kinase alpha	0.0001	-	+/-	-	-	-/-
Q8NBQ5	DHB11	HSD17B11	Estradiol 17-beta-dehydrogenase 11	0.0192	0.0234	0.82	0.0643	0.0620	1.04
Q53GQ0	DHB12	HSD17B12	Very-long-chain 3-oxoacyl-CoA reductase	0.0099	0.0181	0.55	0.0711	0.0689	1.03
Q7Z5P4	DHB13	HSD17B13	17-beta-hydroxysteroid dehydrogenase 13	0.0453	0.1270	0.36	0.0141	0.2201	0.06
P37059	DHB2	HSD17B2	Estradiol 17-beta-dehydrogenase 2	0.0083	0.0074	1.12	0.0393	0.0562	0.70
P51659	DHB4	HSD17B4	Peroxisomal multifunctional enzyme type 2	0.1472	0.1016	1.45	0.1799	0.1316	1.37
Q92506	DHB8	HSD17B8	Estradiol 17-beta-dehydrogenase 8	0.0207	0.0315	0.66	0.0147	0.0440	0.33
Q15392	DHC24	DHCR24	Delta (24)-sterol reductase	0.0027	0.0004	6.25	0.0228	0.0126	1.81
Q9UBM7	DHCR7	DHCR7	7-dehydrocholesterol reductase	0.0008	0.0004	1.96	0.0135	0.0192	0.70
P00367	DHE3	GLUD1	Glutamate dehydrogenase 1, mitochondrial	0.1536	0.3281	0.47	0.0986	0.2542	0.39
P49448	DHE4	GLUD2	Glutamate dehydrogenase 2, mitochondrial	0.0116	-	+/-	0.0341	-	+/-
P28845	DHI1	HSD11B1	Corticosteroid 11-beta-dehydrogenase isozyme 1	0.1071	0.1021	1.05	0.1182	0.1217	0.97

P09417	DHPR	QDPR	Dihydropteridine reductase	0.0580	0.1046	0.55	0.0583	0.1482	0.39
Q6UWP2	DHR11	DHRS11	Dehydrogenase/reductase SDR family member 11	0.0031	0.0090	0.34	-	0.0091	-/+
Q96LJ7	DHRS1	DHRS1	Dehydrogenase/reductase SDR family member 1	0.0215	0.0391	0.55	0.0420	0.0824	0.51
Q13268	DHRS2	DHRS2	Dehydrogenase/reductase SDR family member 2, mitochondrial	0.0045	0.0215	0.21	-	0.0143	-/+
O75911	DHRS3	DHRS3	Short-chain dehydrogenase/reductase 3	0.0004	0.0035	0.12	-	0.0060	-/+
Q9BTZ2	DHRS4	DHRS4	Dehydrogenase/reductase SDR family member 4	0.0126	0.0251	0.50	0.0093	0.0260	0.36
Q9Y394	DHRS7	DHRS7	Dehydrogenase/reductase SDR family member 7	0.0152	0.0107	1.42	0.0565	0.0323	1.75
Q8N5I4	DHRSX	DHRSX	Dehydrogenase/reductase SDR family member on chromosome X	-	-	-/-	0.0056	-	+/-
Q00796	DHSO	SORD	Sorbitol dehydrogenase	0.1250	0.2661	0.47	0.1594	0.2928	0.54
Q96HY7	DHTK1	DHTKD1	Probable 2-oxoglutarate dehydrogenase E1 component DHKTD1, mitochondrial	0.0035	0.0050	0.70	0.0179	0.0292	0.61
O43143	DHX15	DHX15	Pre-mRNA-splicing factor ATP-dependent RNA helicase DHX15	0.0010	0.0002	5.25	0.0087	0.0048	1.82
Q7L2E3	DHX30	DHX30	ATP-dependent RNA helicase DHX30	-	-	-/-	0.0044	0.0019	2.33
Q08211	DHX9	DHX9	ATP-dependent RNA helicase A	0.0101	0.0034	2.96	0.0267	0.0136	1.96
P49366	DHYS	DHPS	Deoxyhypusine synthase	-	0.0007	-/+	-	-	-/-
Q8IYB7	DI3L2	DIS3L2	DIS3-like exonuclease 2	0.0008	-	+/-	-	-	-/-

Q01459	DIAC	CTBS	Di-N-acetylchitobiase	0.0022	-	+/-	0.0053	-	+/-
O60610	DIAP1	DIAPH1	Protein diaphanous homolog 1	0.0013	0.0029	0.43	0.0076	0.0093	0.82
O60879	DIAP2	DIAPH2	Protein diaphanous homolog 2	0.0002	-	+/-	0.0044	0.0019	2.33
Q9UBX3	DIC	SLC25A10	Mitochondrial dicarboxylate carrier	0.0149	0.0215	0.69	0.0385	0.0555	0.69
Q9P265	DIP2B	DIP2B	Disco-interacting protein 2 homolog B	-	-	-/-	0.0026	0.0020	1.30
Q9UBS4	DJB11	DNAJB11	DnaJ homolog subfamily B member 11	0.0245	0.0077	3.17	0.0185	0.0089	2.07
Q9NXW2	DJB12	DNAJB12	DnaJ homolog subfamily B member 12	0.0006	0.0004	1.34	-	-	-/-
Q8IXB1	DJC10	DNAJC10	DnaJ homolog subfamily C member 10	0.0002	-	+/-	0.0021	-	+/-
Q9NVH1	DJC11	DNAJC11	DnaJ homolog subfamily C member 11	0.0022	0.0006	3.72	0.0081	0.0099	0.82
O75165	DJC13	DNAJC13	DnaJ homolog subfamily C member 13	0.0001	-	+/-	0.0034	0.0015	2.23
Q5F1R6	DJC21	DNAJC21	DnaJ homolog subfamily C member 21	-	0.0001	-/+	-	-	-/-
Q9NZQ0	DJC27	DNAJC27	DnaJ homolog subfamily C member 27	0.0001	0.0003	0.50	-	-	-/-
O60832	DKC1	DKC1	H/ACA ribonucleoprotein complex subunit DKC1	0.0010	0.0001	9.39	0.0067	0.0033	2.01
P09622	DLDH	DLD	Dihydrolipoyl dehydrogenase, mitochondrial	0.0224	0.0188	1.19	0.0195	0.0237	0.82
Q12959	DLG1	DLG1	Disks large homolog 1	0.0011	0.0007	1.48	0.0019	-	+/-
Q9NP97	DLRB1	DYNLRB1	Dynein light chain roadblock-type 1	0.0027	0.0064	0.43	-	-	-/-
Q9UGM3	DMBT1	DMBT1	Deleted in malignant brain tumors 1 protein	0.0001	0.0001	1.04	-	-	-/-
P11532	DMD	DMD	Dystrophin	0.0000	0.0001	0.36	-	-	-/-
Q96M86	DNHD1	DNHD1	Dynein heavy chain domain-containing protein 1	-	0.0000	-/+	-	-	-/-

P31689	DNJA1	DNAJA1	DnaJ homolog subfamily A member 1	0.0058	0.0044	1.31	0.0093	0.0109	0.86
O60884	DNJA2	DNAJA2	DnaJ homolog subfamily A member 2	0.0048	0.0029	1.65	0.0099	0.0115	0.86
Q96EY1-2	DNJA3	DNAJA3	Isoform 2 of DnaJ homolog subfamily A member 3, mitochondrial	0.0007	-	+/-	-	-	-/-
Q96EY1	DNJA3	DNAJA3	DnaJ homolog subfamily A member 3, mitochondrial	0.0046	0.0053	0.87	0.0069	0.0099	0.69
P25685	DNJB1	DNAJB1	DnaJ homolog subfamily B member 1	0.0036	0.0026	1.37	0.0076	-	+/-
P25686	DNJB2	DNAJB2	DnaJ homolog subfamily B member 2	0.0049	0.0009	5.68	-	-	-/-
Q9UDY4	DNJB4	DNAJB4	DnaJ homolog subfamily B member 4	0.0001	0.0002	0.76	-	-	-/-
O75190	DNJB6	DNAJB6	DnaJ homolog subfamily B member 6	0.0012	0.0011	1.02	-	-	-/-
Q9UBS3	DNJB9	DNAJB9	DnaJ homolog subfamily B member 9	0.1033	0.2072	0.50	0.0599	0.1533	0.39
Q96KC8	DNJC1	DNAJC1	DnaJ homolog subfamily C member 1	0.0002	-	+/-	-	-	-/-
Q99543	DNJC2	DNAJC2	DnaJ homolog subfamily C member 2	-	-	-/-	0.0015	-	+/-
Q13217	DNJC3	DNAJC3	DnaJ homolog subfamily C member 3	0.0094	0.0026	3.66	0.0189	0.0066	2.85
Q9H3Z4	DNJC5	DNAJC5	DnaJ homolog subfamily C member 5	-	-	-/-	0.0109	-	+/-
O75937	DNJC8	DNAJC8	DnaJ homolog subfamily C member 8	0.0010	0.0004	2.55	-	-	-/-
Q8WXX5	DNJC9	DNAJC9	DnaJ homolog subfamily C member 9	0.0020	0.0019	1.04	-	-	-/-
P49916	DNLI3	LIG3	DNA ligase 3	0.0001	-	+/-	0.0013	-	+/-
O00429	DNM1L	DNM1L	Dynamin-1-like protein	0.0005	0.0006	0.89	0.0066	0.0068	0.97
Q9ULA0	DNPEP	DNPEP	Aspartyl aminopeptidase	0.0036	0.0050	0.71	0.0285	0.0294	0.97

O43598	DNPH1	DNPH1	2'-deoxynucleoside 5'-phosphate N-hydrolase 1	0.0218	0.0173	1.26	0.0359	0.0343	1.04
O00115	DNS2A	DNASE2	Deoxyribonuclease-2-alpha	0.0003	-	+/-	-	-	-/-
Q5JSL3	DOC11	DOCK11	Dedicator of cytokinesis protein 11	0.0001	-	+/-	-	-	-/-
Q92608	DOCK2	DOCK2	Dedicator of cytokinesis protein 2	0.0000	-	+/-	0.0017	0.0013	1.30
Q8N1I0	DOCK4	DOCK4	Dedicator of cytokinesis protein 4	-	-	-/-	0.0008	-	+/-
Q96N67	DOCK7	DOCK7	Dedicator of cytokinesis protein 7	-	-	-/-	-	0.0012	-/+
Q9BU89	DOHH	DOHH	Deoxyhypusine hydroxylase	0.0007	0.0016	0.42	-	-	-/-
P30046	DOPD	DDT	D-dopachrome decarboxylase	0.1515	0.4008	0.38	0.2256	0.4340	0.52
Q9UKG1	DP13A	APPL1	DCC-interacting protein 13-alpha	0.0005	0.0018	0.28	0.0063	0.0025	2.53
Q8NEU8	DP13B	APPL2	DCC-interacting protein 13-beta	-	0.0011	-/+	-	-	-/-
P04440	DPB1	HLA-DPB1	HLA class II histocompatibility antigen, DP beta 1 chain	-	0.0004	-/+	-	-	-/-
P16444	DPEP1	DPEP1	Dipeptidase 1	0.0005	-	+/-	0.0051	-	+/-
Q92782	DPF1	DPF1	Zinc finger protein neuro-d4	0.0007	0.0017	0.40	-	-	-/-
Q9H2P9	DPH5	DPH5	Diphthine methyl ester synthase	0.0002	-	+/-	-	-	-/-
O60762	DPM1	DPM1	Dolichol-phosphate mannosyltransferase subunit 1	0.0046	0.0059	0.77	0.0169	0.0175	0.97
Q9NRF9	DPOE3	POLE3	DNA polymerase epsilon subunit 3	-	0.0005	-/+	-	-	-/-
P06746	DPOLB	POLB	DNA polymerase beta	0.0008	0.0005	1.79	-	-	-/-
Q9UHL4	DPP2	DPP7	Dipeptidyl peptidase 2	0.0087	0.0050	1.73	0.0285	0.0089	3.19

Q9NY33	DPP3	DPP3	Dipeptidyl peptidase 3	0.0017	0.0037	0.45	0.0160	0.0131	1.22
P27487	DPP4	DPP4	Dipeptidyl peptidase 4	0.0167	-	+/-	0.0445	-	+/-
Q86TI2	DPP9	DPP9	Dipeptidyl peptidase 9	0.0002	-	+/-	0.0073	0.0024	3.07
Q12882	DPYD	DPYD	Dihydropyrimidine dehydrogenase [NADP(+)]	0.0005	0.0003	1.65	0.0098	0.0136	0.72
Q14194	DPYL1	CRMP1	Dihydropyrimidinase-related protein 1	0.0005	-	+/-	-	-	-/-
Q16555	DPYL2	DPYSL2	Dihydropyrimidinase-related protein 2	0.0352	0.0200	1.76	0.0536	0.0441	1.21
Q14195-2	DPYL3	DPYSL3	Isoform LCRMP-4 of Dihydropyrimidinase-related protein 3	0.0050	0.0003	15.86	0.0371	-	+/-
Q14195	DPYL3	DPYSL3	Dihydropyrimidinase-related protein 3	0.0170	0.0015	11.33	-	0.0094	-/+
Q14117	DPYS	DPYS	Dihydropyrimidinase	0.0148	0.0408	0.36	0.0218	0.0724	0.30
P01906	DQA2	HLA-DQA2	HLA class II histocompatibility antigen, DQ alpha 2 chain	-	0.0011	-/+	0.0155	-	+/-
P01920	DQB1	HLA-DQB1	HLA class II histocompatibility antigen, DQ beta 1 chain	0.0029	0.0011	2.65	-	-	-/-
P01903	DRA	HLA-DRA	HLA class II histocompatibility antigen, DR alpha chain	0.0526	0.0146	3.62	0.0537	0.0381	1.41
P01911	DRB1	HLA-DRB1	HLA class II histocompatibility antigen, DRB1 beta chain	0.0094	-	+/-	0.0019	-	+/-
P79483	DRB3	HLA-DRB3	HLA class II histocompatibility antigen, DR beta 3 chain	0.0121	0.0027	4.51	0.0112	0.0161	0.69
P13762	DRB4	HLA-DRB4	HLA class II histocompatibility antigen, DR beta 4 chain	-	0.0004	-/+	-	-	-/-

Q30154	DRB5	HLA-DRB5	HLA class II histocompatibility antigen, DR beta 5 chain	0.0005	-	+/-	-	-	-/-
Q8NEE6	DRC6	FBXL13	Dynein regulatory complex subunit 6	-	0.0001	-/+	-	-	-/-
Q16643	DREB	DBN1	Drebrin	0.0046	0.0005	9.58	-	-	-/-
Q9Y295	DRG1	DRG1	Developmentally-regulated GTP-binding protein 1	0.0009	0.0031	0.28	0.0130	0.0085	1.52
P55039	DRG2	DRG2	Developmentally-regulated GTP-binding protein 2	-	0.0003	-/+	-	-	-/-
Q6IAN0	DRS7B	DHRS7B	Dehydrogenase/reductase SDR family member 7B	0.0006	0.0003	2.37	0.0077	0.0110	0.70
Q08554	DSC1	DSC1	Desmocollin-1	0.0015	0.0115	0.13	-	-	-/-
Q14574	DSC3	DSC3	Desmocollin-3	-	0.0005	-/+	-	-	-/-
Q02413	DSG1	DSG1	Desmoglein-1	0.0120	0.0782	0.15	-	-	-/-
Q14126	DSG2	DSG2	Desmoglein-2	0.0024	0.0013	1.86	0.0029	0.0051	0.57
P55265	DSRAD	ADAR	Double-stranded RNA-specific adenosine deaminase	0.0005	-	+/-	0.0044	0.0012	3.51
Q96EV8	DTBP1	DTNBP1	Dysbindin	0.0002	-	+/-	-	-	-/-
Q8TEA8	DTD1	DTD1	D-aminoacyl-tRNA deacylase 1	0.0007	-	+/-	-	-	-/-
Q8TDB6	DTX3L	DTX3L	E3 ubiquitin-protein ligase DTX3L	0.0009	0.0002	4.24	0.0039	-	+/-
Q9NRD8	DUOX2	DUOX2	Dual oxidase 2	-	-	-/-	0.0012	-	+/-
Q9BVJ7	DUS23	DUSP23	Dual specificity protein phosphatase 23	0.0093	0.0179	0.52	0.0547	0.0138	3.98
P51452	DUS3	DUSP3	Dual specificity protein phosphatase 3	0.0272	0.0296	0.92	0.0397	0.0623	0.64

P33316	DUT	DUT	Deoxyuridine 5'-triphosphate nucleotidohydrolase, mitochondrial	0.0047	0.0038	1.24	-	-	-/-
O00148	DX39A	DDX39A	ATP-dependent RNA helicase DDX39A	0.0110	0.0043	2.58	0.0121	-	+/-
Q13838	DX39B	DDX39B	Spliceosome RNA helicase DDX39B	0.0090	0.0070	1.29	0.0385	0.0367	1.05
Q96DT5	DYH11	DNAH11	Dynein axonemal heavy chain 11	0.0000	-	+/-	-	0.0001	-/+
Q0VDD8-4	DYH14	DNAH14	Isoform 4 of Dynein heavy chain 14, axonemal	0.0000	-	+/-	-	-	-/-
Q9UFH2	DYH17	DNAH17	Dynein axonemal heavy chain 17	0.0000	-	+/-	-	-	-/-
Q9P225	DYH2	DNAH2	Dynein heavy chain 2, axonemal	0.0000	-	+/-	0.0001	-	+/-
Q8TE73	DYH5	DNAH5	Dynein axonemal heavy chain 5	-	-	-/-	0.0003	-	+/-
Q9C0G6	DYH6	DNAH6	Dynein axonemal heavy chain 6	0.0000	-	+/-	-	-	-/-
Q8WXX0	DYH7	DNAH7	Dynein heavy chain 7, axonemal	0.0000	-	+/-	-	-	-/-
Q14204	DYHC1	DYNC1H1	Cytoplasmic dynein 1 heavy chain 1	0.0026	0.0005	5.56	0.0178	0.0104	1.70
P63167	DYL1	DYNLL1	Dynein light chain 1, cytoplasmic	0.0234	0.0129	1.82	0.0344	0.0326	1.05
Q96FJ2	DYL2	DYNLL2	Dynein light chain 2, cytoplasmic	0.0055	0.0244	0.23	0.0411	0.0366	1.12
P63172	DYLT1	DYNLT1	Dynein light chain Tctex-type 1	0.0052	0.0075	0.69	-	-	-/-
P51808	DYLT3	DYNLT3	Dynein light chain Tctex-type 3	-	-	-/-	0.0266	-	+/-
P50570	DYN2	DNM2	Dynamin-2	0.0029	0.0001	23.61	0.0193	0.0101	1.91
Q9UQ16	DYN3	DNM3	Dynamin-3	-	-	-/-	0.0055	-	+/-
P00374	DYR	DHFR	Dihydrofolate reductase	0.0008	0.0040	0.20	-	-	-/-
O75923	DYSF	DYSF	Dysferlin	0.0035	0.0001	61.93	0.0075	0.0035	2.15

O75923-15	DYSF	DYSF	Isoform 15 of Dysferlin	-	-	-/-	0.0091	-	+/-
Q03001	DYST	DST	Dystonin	0.0001	-	+/-	0.0005	0.0002	2.43
P19525	E2AK2	EIF2AK2	Interferon-induced, double-stranded RNA-activated protein kinase	0.0007	0.0007	1.09	0.0023	0.0036	0.64
Q9NZJ5	E2AK3	EIF2AK3	Eukaryotic translation initiation factor 2-alpha kinase 3	0.0001	-	+/-	-	-	-/-
O43491	E41L2	EPB41L2	Band 4.1-like protein 2	0.0034	0.0015	2.25	0.0074	0.0044	1.67
Q9Y2J2	E41L3	EPB41L3	Band 4.1-like protein 3	0.0004	0.0003	1.15	-	-	-/-
Q15125	EBP	EBP	3-beta-hydroxysteroid-Delta (8),Delta (7)-isomerase	0.0029	0.0022	1.33	0.0246	0.0236	1.04
Q13011	ECH1	ECH1	Delta (3,5)-Delta (2,4)-dienoyl-CoA isomerase, mitochondrial	0.1696	0.1687	1.01	0.0811	0.1308	0.62
P40939	ECHA	HADHA	Trifunctional enzyme subunit alpha, mitochondrial	0.1170	0.1536	0.76	0.0970	0.1464	0.66
P55084	ECHB	HADHB	Trifunctional enzyme subunit beta, mitochondrial	0.1245	0.2078	0.60	0.1276	0.2174	0.59
Q9NTX5	ECHD1	ECHDC1	Ethylmalonyl-CoA decarboxylase	0.0107	0.0171	0.63	0.0116	0.0434	0.27
Q86YB7-2	ECHD2	ECHDC2	Isoform 2 of Enoyl-CoA hydratase domain-containing protein 2, mitochondrial	-	0.0014	-/+	-	-	-/-
Q86YB7	ECHD2	ECHDC2	Enoyl-CoA hydratase domain-containing protein 2, mitochondrial	0.0250	0.0814	0.31	0.0166	0.0590	0.28
Q96DC8	ECHD3	ECHDC3	Enoyl-CoA hydratase domain-containing protein 3, mitochondrial	0.0170	0.0530	0.32	0.0261	0.0848	0.31

P30084	ECHM	ECHS1	Enoyl-CoA hydratase, mitochondrial	0.2703	0.4551	0.59	0.1658	0.4168	0.40
Q08426	ECHP	EHHADH	Peroxisomal bifunctional enzyme	0.1229	0.1525	0.81	0.1747	0.2329	0.75
P42126	ECI1	ECI1	Enoyl-CoA delta isomerase 1, mitochondrial	0.0288	0.0384	0.75	0.0161	0.0545	0.30
O75521	ECI2	ECI2	Enoyl-CoA delta isomerase 2, mitochondrial	0.0200	0.0326	0.61	0.0437	0.0718	0.61
Q16610	ECM1	ECM1	Extracellular matrix protein 1	-	-	-/-	0.0038	-	+/-
Q5VYK3	ECM29	ECPAS	Proteasome adapter and scaffold protein ECM29	0.0001	-	+/-	0.0019	0.0030	0.64
P12724	ECP	RNASE3	Eosinophil cationic protein	0.0011	-	+/-	0.0202	-	+/-
Q9BQ95	ECSIT	ECSIT	Evolutionarily conserved signaling intermediate in Toll pathway, mitochondrial	0.0019	0.0018	1.06	0.0122	0.0047	2.63
Q6P2E9	EDC4	EDC4	Enhancer of mRNA-decapping protein 4	-	-	-/-	0.0016	-	+/-
Q9BZQ6	EDEM3	EDEM3	ER degradation-enhancing alpha-mannosidase-like protein 3	0.0002	-	+/-	-	-	-/-
O60869	EDF1	EDF1	Endothelial differentiation-related factor 1	0.0007	0.0004	1.89	0.0071	-	+/-
Q15075	EEA1	EEA1	Early endosome antigen 1	0.0006	-	+/-	-	-	-/-
P68104	EF1A1	EEF1A1	Elongation factor 1-alpha 1	0.1285	0.1202	1.07	0.0648	0.0769	0.84
Q05639	EF1A2	EEF1A2	Elongation factor 1-alpha 2	0.0211	0.0098	2.15	0.0367	0.0193	1.90
P24534	EF1B	EEF1B2	Elongation factor 1-beta	0.0304	0.0263	1.16	0.0169	0.0164	1.03
P29692-2	EF1D	EEF1D	Isoform 2 of Elongation factor 1-delta	0.0002	-	+/-	-	-	-/-
P29692-3	EF1D	EEF1D	Isoform 3 of Elongation factor 1-delta	0.0187	0.0209	0.90	-	-	-/-
P29692-4	EF1D	EEF1D	Isoform 4 of Elongation factor 1-delta	0.0047	0.0097	0.49	-	-	-/-

P29692	EF1D	EEF1D	Elongation factor 1-delta	0.1058	0.0418	2.54	0.0410	0.0253	1.62
P26641	EF1G	EEF1G	Elongation factor 1-gamma	0.0323	0.0227	1.42	0.0749	0.0442	1.70
P13639	EF2	EEF2	Elongation factor 2	0.0790	0.0417	1.89	0.0981	0.0871	1.13
O00418	EF2K	EEF2K	Eukaryotic elongation factor 2 kinase	-	0.0003	-/+	-	-	-/-
Q96RP9	EFGM	GFM1	Elongation factor G, mitochondrial	0.0012	0.0007	1.69	0.0053	0.0067	0.80
Q9BUP0	EFHD1	EFHD1	EF-hand domain-containing protein D1	0.0066	0.0111	0.60	-	0.0053	-/+
Q96C19	EFHD2	EFHD2	EF-hand domain-containing protein D2	0.0376	0.0153	2.45	0.0180	0.0117	1.55
Q7Z2Z2	EFL1	EFL1	Elongation factor-like GTPase 1	0.0001	-	+/-	-	-	-/-
Q5JPI9	EFMT2	EEF1AKMT2	EEF1A lysine methyltransferase 2	0.0002	0.0012	0.19	-	-	-/-
Q8N6R0	EFNMT	EEF1AKNMT	eEF1A lysine and N-terminal methyltransferase	0.0005	-	+/-	-	-	-/-
P43897	EFTS	TSFM	Elongation factor Ts, mitochondrial	0.0118	0.0052	2.27	0.0077	0.0052	1.49
P49411	EFTU	TUFM	Elongation factor Tu, mitochondrial	0.1101	0.0698	1.58	0.0705	0.0777	0.91
Q9UHF1	EGFL7	EGFL7	Epidermal growth factor-like protein 7	0.0002	-	+/-	-	-	-/-
P00533	EGFR	EGFR	Epidermal growth factor receptor	0.0008	-	+/-	0.0031	0.0016	1.97
P17813	EGLN	ENG	Endoglin	0.0016	-	+/-	-	-	-/-
Q9GZT9	EGLN1	EGLN1	Egl nine homolog 1	0.0009	-	+/-	-	-	-/-
Q9H4M9	EHD1	EHD1	EH domain-containing protein 1	0.0028	0.0004	6.86	0.0165	0.0224	0.74
Q9NZN4	EHD2	EHD2	EH domain-containing protein 2	0.0007	0.0024	0.29	0.0161	0.0147	1.09
Q9NZN3	EHD3	EHD3	EH domain-containing protein 3	0.0011	0.0028	0.37	-	0.0206	-/+

Q9H223	EHD4	EHD4	EH domain-containing protein 4	0.0045	0.0021	2.14	0.0292	0.0149	1.96
Q14232	EI2BA	EIF2B1	Translation initiation factor eIF-2B subunit alpha	0.0006	0.0004	1.53	-	0.0053	-/+
P49770	EI2BB	EIF2B2	Translation initiation factor eIF-2B subunit beta	0.0007	0.0016	0.41	0.0054	0.0056	0.97
Q9UI10	EI2BD	EIF2B4	Translation initiation factor eIF-2B subunit delta	0.0005	0.0004	1.04	0.0041	0.0042	0.97
Q13144	EI2BE	EIF2B5	Translation initiation factor eIF-2B subunit epsilon	-	-	-/-	0.0058	0.0060	0.97
Q9NR50	EI2BG	EIF2B3	Translation initiation factor eIF-2B subunit gamma	0.0001	0.0022	0.06	-	-	-/-
P41567	EIF1	EIF1	Eukaryotic translation initiation factor 1	0.0018	0.0094	0.19	-	-	-/-
O60739	EIF1B	EIF1B	Eukaryotic translation initiation factor 1b	0.0058	0.0057	1.01	-	-	-/-
Q9BY44	EIF2A	EIF2A	Eukaryotic translation initiation factor 2A	0.0011	0.0021	0.54	0.0079	0.0031	2.55
Q14152	EIF3A	EIF3A	Eukaryotic translation initiation factor 3 subunit A	0.0012	0.0010	1.26	0.0056	0.0040	1.40
P55884	EIF3B	EIF3B	Eukaryotic translation initiation factor 3 subunit B	0.0043	0.0053	0.81	0.0191	0.0114	1.67
Q99613	EIF3C	EIF3C	Eukaryotic translation initiation factor 3 subunit C	0.0001	-	+/-	-	-	-/-
O15371	EIF3D	EIF3D	Eukaryotic translation initiation factor 3 subunit D	0.0009	0.0008	1.22	0.0135	0.0038	3.58

P60228	EIF3E	EIF3E	Eukaryotic translation initiation factor 3 subunit E	0.0010	0.0015	0.68	0.0311	0.0123	2.53
O00303	EIF3F	EIF3F	Eukaryotic translation initiation factor 3 subunit F	0.0591	0.0270	2.19	0.0511	0.0339	1.51
O75821	EIF3G	EIF3G	Eukaryotic translation initiation factor 3 subunit G	0.0085	0.0058	1.47	0.0042	0.0043	0.97
O15372	EIF3H	EIF3H	Eukaryotic translation initiation factor 3 subunit H	0.0102	0.0039	2.60	0.0077	0.0052	1.49
Q13347	EIF3I	EIF3I	Eukaryotic translation initiation factor 3 subunit I	0.0312	0.0151	2.07	0.0529	0.0107	4.95
O75822	EIF3J	EIF3J	Eukaryotic translation initiation factor 3 subunit J	0.0030	0.0040	0.75	0.0042	0.0067	0.63
Q9UBQ5	EIF3K	EIF3K	Eukaryotic translation initiation factor 3 subunit K	0.0085	0.0097	0.88	0.0186	0.0120	1.55
Q9Y262	EIF3L	EIF3L	Eukaryotic translation initiation factor 3 subunit L	0.0013	0.0002	8.03	0.0135	0.0065	2.09
Q7L2H7	EIF3M	EIF3M	Eukaryotic translation initiation factor 3 subunit M	-	0.0020	-/+	0.0213	0.0127	1.68
B5ME19	EIFCL	EIF3CL	Eukaryotic translation initiation factor 3 subunit C-like protein	0.0007	0.0001	7.09	0.0092	0.0070	1.32
Q53HC9	EIPR1	EIPR1	EARP and GARP complex-interacting protein 1	0.0021	0.0035	0.59	-	-	-/-
Q15717	ELAV1	ELAVL1	ELAV-like protein 1	0.0167	0.0203	0.82	0.0116	0.0120	0.97
Q8IZ81	ELMD2	ELMOD2	ELMO domain-containing protein 2	-	-	-/-	0.0082	0.0055	1.50

Q92556	ELMO1	ELMO1	Engulfment and cell motility protein 1	-	-	-/-	0.0034	-	+/-
Q96JJ3	ELMO2	ELMO2	Engulfment and cell motility protein 2	-	-	-/-	0.0035	-	+/-
P15502	ELN	ELN	Elastin	-	-	-/-	0.0169	0.0110	1.54
P08246	ELNE	ELANE	Neutrophil elastase	0.0319	-	+/-	0.1937	0.0312	6.22
Q15370	ELOB	ELOB	Elongin-B	0.0350	0.0521	0.67	-	-	-/-
Q15369	ELOC	ELOC	Elongin-C	0.0512	0.0603	0.85	0.0397	0.0623	0.64
O95163	ELP1	ELP1	Elongator complex protein 1	0.0001	-	+/-	0.0022	-	+/-
Q9H9T3	ELP3	ELP3	Elongator complex protein 3	-	-	-/-	0.0040	0.0027	1.47
Q8TE02	ELP5	ELP5	Elongator complex protein 5	0.0020	-	+/-	-	-	-/-
Q00013	EM55	MPP1	55 kDa erythrocyte membrane protein	0.0013	0.0002	6.79	0.0074	0.0037	2.02
O95834	EMAL2	EML2	Echinoderm microtubule-associated protein-like 2	0.0003	0.0009	0.37	0.0076	0.0037	2.02
Q32P44	EMAL3	EML3	Echinoderm microtubule-associated protein-like 3	-	-	-/-	0.0023	-	+/-
Q9HC35	EMAL4	EML4	Echinoderm microtubule-associated protein-like 4	0.0030	0.0019	1.59	0.0191	0.0236	0.81
Q6ZMW3	EMAL6	EML6	Echinoderm microtubule-associated protein-like 6	-	0.0001	-/+	-	-	-/-
Q8N766	EMC1	EMC1	ER membrane protein complex subunit 1	0.0016	-	+/-	0.0109	0.0042	2.60
Q5UCC4	EMC10	EMC10	ER membrane protein complex subunit 10	0.0029	0.0005	5.85	-	-	-/-
Q15006	EMC2	EMC2	ER membrane protein complex subunit 2	0.0046	0.0022	2.12	0.0119	-	+/-

Q9P0I2	EMC3	EMC3	ER membrane protein complex subunit 3	0.0005	-	+/-	0.0116	-	+/-
Q5J8M3	EMC4	EMC4	ER membrane protein complex subunit 4	0.0004	0.0006	0.67	-	-	-/-
Q9NPA0	EMC7	EMC7	ER membrane protein complex subunit 7	0.0074	0.0040	1.86	0.0074	-	+/-
O43402	EMC8	EMC8	ER membrane protein complex subunit 8	0.0074	0.0055	1.34	-	-	-/-
Q9Y3B6	EMC9	EMC9	ER membrane protein complex subunit 9	0.0012	-	+/-	-	-	-/-
P50402	EMD	EMD	Emerin	0.0010	0.0023	0.43	0.0361	-	+/-
Q9Y6C2	EMIL1	EMILIN1	EMILIN-1	0.0266	0.0173	1.54	0.0331	0.0197	1.68
Q8N8S7	ENAH	ENAH	Protein enabled homolog	0.0003	-	+/-	-	-	-/-
O94919	ENDD1	ENDOD1	Endonuclease domain-containing 1 protein	0.0007	-	+/-	-	-	-/-
P06733	ENOA	ENO1	Alpha-enolase	0.2169	0.2233	0.97	0.2384	0.2529	0.94
P13929	ENOB	ENO3	Beta-enolase	0.0065	0.0313	0.21	-	0.0504	-/+
Q7L5Y1	ENOF1	ENOSF1	Mitochondrial enolase superfamily member 1	0.0001	0.0007	0.19	0.0038	0.0060	0.63
P09104	ENOG	ENO2	Gamma-enolase	0.0023	0.0019	1.19	-	0.0089	-/+
Q9UHY7	ENOPH	ENOPH1	Enolase-phosphatase E1	0.0103	0.0136	0.75	0.0207	0.0214	0.97
P14625	ENPL	HSP90B1	Endoplasmin	0.2714	0.1584	1.71	0.1338	0.1160	1.15
Q58FF3	ENPLL	HSP90B2P	Putative endoplasmin-like protein	0.0007	-	+/-	-	-	-/-
P22413	ENPP1	ENPP1	Ectonucleotide pyrophosphatase/phosphodiesterase family member 1	0.0004	0.0004	1.08	0.0019	0.0030	0.64
Q9Y6X5	ENPP4	ENPP4	Bis (5'-adenosyl)-triphosphatase ENPP4	0.0021	-	+/-	0.0056	-	+/-

P49961	ENTP1	ENTPD1	Ectonucleoside triphosphate diphosphohydrolase 1	-	-	-/-	0.0044	-	+/-
O75356	ENTP5	ENTPD5	Ectonucleoside triphosphate diphosphohydrolase 5	0.0530	0.0241	2.20	0.0508	0.0454	1.12
Q9UBC2	EP15R	EPS15L1	Epidermal growth factor receptor substrate 15-like 1	0.0026	0.0044	0.59	-	-	-/-
Q96L91	EP400	EP400	E1A-binding protein p400	-	-	-/-	-	0.0007	-/+
P16452	EPB42	EPB42	Erythrocyte membrane protein band 4.2	0.0002	-	+/-	-	-	-/-
Q9UNN8	EPCR	PROCR	Endothelial protein C receptor	0.0011	-	+/-	-	-	-/-
Q9UM22	EPDR1	EPDR1	Mammalian ependymin-related protein 1	0.0024	-	+/-	0.0207	-	+/-
P29317	EPHA2	EPHA2	Ephrin type-A receptor 2	-	-	-/-	0.0019	-	+/-
P58107	EPIPL	EPPK1	Epiplakin	0.0006	0.0000	29.61	0.0003	-	+/-
O95278	EPM2A	EPM2A	Laforin	-	0.0012	-/+	-	-	-/-
Q9Y6I3	EPN1	EPN1	Epsin-1	-	0.0023	-/+	-	-	-/-
Q14677	EPN4	CLINT1	Clathrin interactor 1	0.0009	0.0012	0.74	0.0030	-	+/-
P42566	EPS15	EPS15	Epidermal growth factor receptor substrate 15	0.0026	0.0025	1.05	-	0.0024	-/+
Q12929	EPS8	EPS8	Epidermal growth factor receptor kinase substrate 8	-	-	-/-	0.0019	-	+/-
Q9NZ08	ERAP1	ERAP1	Endoplasmic reticulum aminopeptidase 1	0.0024	0.0003	7.14	0.0146	0.0151	0.97
Q6P179	ERAP2	ERAP2	Endoplasmic reticulum aminopeptidase 2	-	-	-/-	0.0075	0.0031	2.37
Q96RT1	ERBIN	ERBIN	Erbin	0.0006	0.0001	6.64	-	-	-/-

P24390	ERD21	KDELR1	ER lumen protein-retaining receptor 1	0.0004	-	+/-	-	-	-/-
P33947	ERD22	KDELR2	ER lumen protein-retaining receptor 2	0.0021	-	+/-	0.0056	-	+/-
P62495	ERF1	ETF1	Eukaryotic peptide chain release factor subunit 1	0.0030	0.0018	1.65	0.0093	0.0036	2.58
P15170-2	ERF3A	GSPT1	Isoform 2 of Eukaryotic peptide chain release factor GTP-binding subunit ERF3A	0.0005	0.0005	1.01	-	-	-/-
P15170	ERF3A	GSPT1	Eukaryotic peptide chain release factor GTP-binding subunit ERF3A	0.0009	0.0002	5.21	0.0047	0.0066	0.71
O76062	ERG24	TM7SF2	Delta(14)-sterol reductase TM7SF2	-	0.0003	-/+	0.0062	0.0099	0.62
Q9UKR5	ERG28	ERG28	Ergosterol biosynthetic protein 28 homolog	-	-	-/-	-	0.0175	-/+
P48449	ERG7	LSS	Lanosterol synthase	0.0057	0.0096	0.60	0.0102	0.0390	0.26
Q969X5	ERGI1	ERGIC1	Endoplasmic reticulum-Golgi intermediate compartment protein 1	0.0181	0.0155	1.16	0.0405	0.0476	0.85
Q96RQ1	ERGI2	ERGIC2	Endoplasmic reticulum-Golgi intermediate compartment protein 2	-	-	-/-	0.0058	-	+/-
P84090	ERH	ERH	Enhancer of rudimentary homolog	0.0060	0.0059	1.00	0.0089	-	+/-
O43414	ERI3	ERI3	ERI1 exoribonuclease 3	0.0009	0.0018	0.51	0.0121	0.0125	0.97
Q96DZ1	ERLEC	ERLEC1	Endoplasmic reticulum lectin 1	0.0014	0.0008	1.79	0.0055	-	+/-
O75477	ERLN1	ERLIN1	Erlin-1	0.0062	0.0098	0.63	0.0217	0.0347	0.63
O94905-3	ERLN2	ERLIN2	Isoform 3 of Erlin-2	0.0007	-	+/-	-	-	-/-
O94905	ERLN2	ERLIN2	Erlin-2	0.0840	0.0372	2.26	0.0904	0.0710	1.27
Q7Z2K6	ERMP1	ERMP1	Endoplasmic reticulum metallopeptidase 1	-	-	-/-	0.0133	0.0027	4.88

Q96HE7	ERO1A	ERO1A	ERO1-like protein alpha	0.0172	0.0069	2.48	0.0376	0.0289	1.30
P30040	ERP29	ERP29	Endoplasmic reticulum resident protein 29	0.0469	0.0559	0.84	0.0563	0.0478	1.18
Q9BS26	ERP44	ERP44	Endoplasmic reticulum resident protein 44	0.0653	0.0293	2.23	0.0396	0.0374	1.06
Q9H6S3	ES8L2	EPS8L2	Epidermal growth factor receptor kinase substrate 8-like protein 2	0.0017	0.0027	0.62	0.0022	0.0046	0.47
Q14674	ESPL1	ESPL1	Separin	0.0001	-	+/-	-	-	-/-
Q9H6T0	ESRP2	ESRP2	Epithelial splicing regulatory protein 2	0.0001	0.0006	0.17	-	-	-/-
P23141-3	EST1	CES1	Isoform 3 of Liver carboxylesterase 1	0.0112	0.0507	0.22	0.6676	0.6598	1.01
P23141	EST1	CES1	Liver carboxylesterase 1	0.7082	0.5474	1.29	0.7173	0.7259	0.99
O00748	EST2	CES2	Cocaine esterase	0.0857	0.0600	1.43	0.0656	0.0748	0.88
Q6UWW8	EST3	CES3	Carboxylesterase 3	-	0.0011	-/+	-	-	-/-
P10768	ESTD	ESD	S-formylglutathione hydrolase	0.0414	0.0554	0.75	0.0862	0.1037	0.83
Q9BSJ8	ESYT1	ESYT1	Extended synaptotagmin-1	0.0103	0.0017	5.99	0.0290	0.0194	1.49
A0FGR8	ESYT2	ESYT2	Extended synaptotagmin-2	0.0002	0.0001	2.37	0.0034	0.0017	1.97
P13804	ETFA	ETFA	Electron transfer flavoprotein subunit alpha, mitochondrial	0.0924	0.1089	0.85	0.0894	0.1053	0.85
P38117	ETFB	ETFB	Electron transfer flavoprotein subunit beta	0.0909	0.0993	0.92	0.0542	0.0889	0.61
Q16134	ETFD	ETFDH	Electron transfer flavoprotein-ubiquinone oxidoreductase, mitochondrial	0.0188	0.0422	0.45	0.0362	0.0676	0.54
O95571	ETHE1	ETHE1	Persulfide dioxygenase ETHE1, mitochondrial	0.0316	0.0261	1.21	0.0186	0.0254	0.73

Q8IXQ9	ETKMT	ETFBKMT	Electron transfer flavoprotein beta subunit lysine methyltransferase	-	0.0005	-/+	-	-	-/-
Q9UI08	EVL	EVL	Ena/VASP-like protein	0.0027	0.0008	3.59	0.0069	-	+/-
Q9UI08-2	EVL	EVL	Isoform 1 of Ena/VASP-like protein	0.0001	-	+/-	-	-	-/-
Q01844	EWS	EWSR1	RNA-binding protein EWS	0.0005	-	+/-	0.0051	-	+/-
Q9NVH0	EXD2	EXD2	Exonuclease 3'-5' domain-containing protein 2	0.0003	-	+/-	-	-	-/-
Q96KP1	EXOC2	EXOC2	Exocyst complex component 2	-	-	-/-	0.0037	0.0018	1.98
O60645	EXOC3	EXOC3	Exocyst complex component 3	0.0002	-	+/-	-	-	-/-
O00471	EXOC5	EXOC5	Exocyst complex component 5	-	-	-/-	0.0042	-	+/-
Q8TAG9	EXOC6	EXOC6	Exocyst complex component 6	-	-	-/-	0.0019	-	+/-
Q9UPT5	EXOC7	EXOC7	Exocyst complex component 7	0.0002	0.0002	1.28	-	-	-/-
Q8IYI6	EXOC8	EXOC8	Exocyst complex component 8	0.0001	-	+/-	-	-	-/-
Q9Y3B2	EXOS1	EXOSC1	Exosome complex component CSL4	-	0.0010	-/+	-	-	-/-
Q13868	EXOS2	EXOSC2	Exosome complex component RRP4	0.0007	0.0003	2.33	-	-	-/-
Q9NPD3	EXOS4	EXOSC4	Exosome complex component RRP41	0.0023	0.0022	1.03	-	-	-/-
Q5RKV6	EXOS6	EXOSC6	Exosome complex component MTR3	0.0004	0.0016	0.24	-	-	-/-
Q06265	EXOS9	EXOSC9	Exosome complex component RRP45	0.0002	-	+/-	-	-	-/-
Q01780	EXOSX	EXOSC10	Exosome component 10	-	-	-/-	0.0016	-	+/-
P15311	EZRI	EZR	Ezrin	0.0145	0.0114	1.27	0.0151	0.0191	0.79
Q5T9C2	F102A	FAM102A	Protein FAM102A	-	0.0007	-/+	-	-	-/-

Q9H098	F107B	FAM107B	Protein FAM107B	0.0004	-	+/-	-	-	-/-
P50502	F10A1	ST13	Hsc70-interacting protein	0.0026	0.0042	0.62	0.0008	0.0073	0.11
Q9NRY5	F1142	FAM114A2	Protein FAM114A2	-	0.0025	-/+	-	-	-/-
Q9NZB2	F120A	FAM120A	Constitutive coactivator of PPAR-gamma-like protein 1	0.0014	0.0005	3.08	0.0109	0.0082	1.33
Q96C01	F136A	FAM136A	Protein FAM136A	0.0023	0.0034	0.68	-	-	-/-
P00488	F13A	F13A1	Coagulation factor XIII A chain	0.0021	-	+/-	0.0207	-	+/-
Q96A26	F162A	FAM162A	Protein FAM162A	0.0033	0.0083	0.40	0.0016	0.0016	1.06
P09467	F16P1	FBP1	Fructose-1,6-bisphosphatase 1	0.0792	0.2556	0.31	0.0682	0.2816	0.24
O00757	F16P2	FBP2	Fructose-1,6-bisphosphatase isozyme 2	0.0099	0.0231	0.43	0.0079	0.0307	0.26
Q8WUF8	F172A	FAM172A	Cotranscriptional regulator FAM172A	0.0001	-	+/-	-	-	-/-
Q8N128	F177A	FAM177A1	Protein FAM177A1	0.0055	0.0031	1.79	-	-	-/-
Q8N0U4	F185A	FAM185A	Protein FAM185A	0.0001	0.0014	0.09	-	-	-/-
Q8IYM0	F186B	FAM186B	Protein FAM186B	-	-	-/-	0.0003	-	+/-
Q96ND0	F210A	FAM210A	Protein FAM210A	0.0004	0.0002	1.66	-	-	-/-
Q9NTX9	F217B	FAM217B	Protein FAM217B	0.0015	-	+/-	-	-	-/-
P16118	F261	PFKFB1	6-phosphofructo-2-kinase/fructose-2,6-bisphosphatase 1	-	0.0005	-/+	-	0.0072	-/+
O60825	F262	PFKFB2	6-phosphofructo-2-kinase/fructose-2,6-bisphosphatase 2	-	-	-/-	0.0028	-	+/-
P00742	FA10	F10	Coagulation factor X	0.0008	0.0002	3.78	0.0032	-	+/-

P03951	FA11	F11	Coagulation factor XI	0.0004	0.0002	1.50	-	-	-/-
P00748	FA12	F12	Coagulation factor XII	0.0019	0.0006	3.02	0.0054	0.0037	1.48
Q9H0Q0	FA49A	FAM49A	Protein FAM49A	0.0001	-	+/-	0.0113	-	+/-
Q9NUQ9	FA49B	FAM49B	Protein FAM49B	0.0026	0.0003	9.80	0.0426	0.0113	3.77
P12259	FA5	F5	Coagulation factor V	0.0002	-	+/-	0.0018	-	+/-
Q14320	FA50A	FAM50A	Protein FAM50A	0.0002	-	+/-	-	-	-/-
P08709	FA7	F7	Coagulation factor VII	0.0005	0.0014	0.37	0.0041	-	+/-
Q9UBU6	FA8A1	FAM8A1	Protein FAM8A1	-	-	-/-	0.0058	-	+/-
P00740	FA9	F9	Coagulation factor IX	0.0149	0.0072	2.09	0.0217	0.0169	1.29
Q8NCA5	FA98A	FAM98A	Protein FAM98A	0.0015	0.0004	3.39	0.0069	0.0034	2.01
Q52LJ0	FA98B	FAM98B	Protein FAM98B	0.0018	0.0009	1.96	-	0.0035	-/+
Q17RN3	FA98C	FAM98C	Protein FAM98C	0.0011	-	+/-	-	-	-/-
P16930	FAAA	FAH	Fumarylacetoacetase	0.0703	0.1624	0.43	0.0883	0.3272	0.27
O00519	FAAH1	FAAH	Fatty-acid amide hydrolase 1	-	-	-/-	0.0114	0.0096	1.19
Q8IVS2	FABD	MCAT	Malonyl-CoA-acyl carrier protein transacylase, mitochondrial	-	-	-/-	-	0.0048	-/+
P15090	FABP4	FABP4	Fatty acid-binding protein, adipocyte	0.0111	0.0049	2.28	-	-	-/-
Q01469	FABP5	FABP5	Fatty acid-binding protein 5	0.0481	0.0653	0.74	0.0103	0.0107	0.97
P05413	FABPH	FABP3	Fatty acid-binding protein, heart	0.0182	0.0102	1.79	-	-	-/-
P07148	FABPL	FABP1	Fatty acid-binding protein, liver	0.5784	1.2160	0.48	0.2510	0.7134	0.35

O75844	FACE1	ZMPSTE24	CAAX prenyl protease 1 homolog	0.0001	-	+/-	0.0152	-	+/-
Q8NFF5	FAD1	FLAD1	FAD synthase	0.0006	0.0004	1.52	0.0052	0.0035	1.48
Q96CS3	FAF2	FAF2	FAS-associated factor 2	0.0005	0.0013	0.42	0.0051	0.0072	0.71
Q96GK7	FAH2A	FAHD2A	Fumarylacetoacetate hydrolase domain-containing protein 2A	0.0132	0.0243	0.54	0.0093	0.0436	0.21
Q6P2I3	FAH2B	FAHD2B	Fumarylacetoacetate hydrolase domain-containing protein 2B	0.0025	0.0010	2.58	-	-	-/-
Q6P587	FAHD1	FAHD1	Acylpyruvase FAHD1, mitochondrial	0.0183	0.0231	0.79	0.0233	0.0373	0.62
Q53R41	FAKD1	FASTKD1	FAST kinase domain-containing protein 1, mitochondrial	0.0001	-	+/-	-	-	-/-
Q969Z0	FAKD4	TBRG4	FAST kinase domain-containing protein 4	0.0001	-	+/-	-	0.0030	-/+
Q92520	FAM3C	FAM3C	Protein FAM3C	0.0013	0.0005	2.74	0.0172	-	+/-
Q9Y2M0	FAN1	FAN1	Fanconi-associated nuclease 1	0.0001	-	+/-	-	-	-/-
Q9Y4F1	FARP1	FARP1	FERM, ARHGEF and pleckstrin domain-containing protein 1	0.0001	0.0001	0.51	0.0047	0.0057	0.82
Q9Y4F1-2	FARP1	FARP1	Isoform 2 of FERM, ARHGEF and pleckstrin domain-containing protein 1	-	-	-/-	-	0.0023	-/+
P49327	FAS	FASN	Fatty acid synthase	0.0572	0.0683	0.84	0.0593	0.1326	0.45
Q14517	FAT1	FAT1	Protocadherin Fat 1	0.0000	-	+/-	-	-	-/-
Q8TDW7	FAT3	FAT3	Protocadherin Fat 3	0.0000	-	+/-	-	-	-/-
P23142	FBLN1	FBLN1	Fibulin-1	0.0222	0.0118	1.87	0.0214	0.0113	1.89
P23142-4	FBLN1	FBLN1	Isoform C of Fibulin-1	0.0080	0.0048	1.66	0.0107	0.0091	1.18

P98095	FBLN2	FBLN2	Fibulin-2	0.0063	-	+/-	0.0127	-	+/-
Q12805	FBLN3	EFEMP1	EGF-containing fibulin-like extracellular matrix protein 1	0.0259	0.0023	11.39	0.0914	0.0053	17.20
O95967	FBLN4	EFEMP2	EGF-containing fibulin-like extracellular matrix protein 2	0.0010	0.0005	2.02	0.0107	-	+/-
Q9UBX5	FBLN5	FBLN5	Fibulin-5	0.0356	0.0052	6.91	0.0619	0.0186	3.32
P35555	FBN1	FBN1	Fibrillin-1	0.0021	-	+/-	0.0659	0.0283	2.33
Q5T0N5	FBP1L	FNBP1L	Formin-binding protein 1-like	0.0001	-	+/-	-	-	-/-
P22087	FBRL	FBL	rRNA 2'-O-methyltransferase fibrillarin	0.0204	0.0164	1.24	0.0301	0.0275	1.09
Q9UK22	FBX2	FBXO2	F-box only protein 2	0.0006	0.0029	0.21	-	0.0087	-/+
Q8NEZ5	FBX22	FBXO22	F-box only protein 22	-	-	-/-	0.0071	-	+/-
Q9UKT5	FBX4	FBXO4	F-box only protein 4	0.0003	0.0003	1.06	-	-	-/-
Q6ZVX7	FBX50	NCCRP1	F-box only protein 50	-	0.0063	-/+	-	-	-/-
P08637	FCG3A	FCGR3A	Low affinity immunoglobulin gamma Fc region receptor III-A	-	-	-/-	0.0074	-	+/-
Q9Y6R7	FCGBP	FCGBP	IgGFc-binding protein	0.0001	-	+/-	-	-	-/-
P55899	FCGRN	FCGRT	IgG receptor FcRn large subunit p51	0.0018	-	+/-	0.0284	0.0148	1.91
Q13630	FCL	TSTA3	GDP-L-fucose synthase	0.0090	0.0065	1.37	0.0215	0.0179	1.21
O00602	FCN1	FCN1	Ficolin-1	0.0075	0.0006	11.97	-	-	-/-
Q15485	FCN2	FCN2	Ficolin-2	0.0006	-	+/-	-	-	-/-
O75636	FCN3	FCN3	Ficolin-3	0.0036	0.0024	1.53	-	-	-/-

Q8N0W3	FCSK	FCSK	L-fucose kinase	0.0001	-	+/-	0.0033	-	+/-
P37268	FDFT	FDFT1	Squalene synthase	-	-	-/-	0.0010	-	+/-
P39748	FEN1	FEN1	Flap endonuclease 1	0.0006	0.0008	0.72	-	-	-/-
Q96AC1	FERM2	FERMT2	Fermitin family homolog 2	0.0006	0.0016	0.40	0.0112	0.0189	0.59
P02771	FETA	AFP	Alpha-fetoprotein	0.0012	-	+/-	-	-	-/-
P02765	FETUA	AHSG	Alpha-2-HS-glycoprotein	0.0133	0.0096	1.38	0.0134	0.0129	1.03
Q96C11	FGGY	FGGY	FGGY carbohydrate kinase domain-containing protein	0.0002	0.0035	0.06	0.0088	0.0235	0.38
Q08830	FGL1	FGL1	Fibrinogen-like protein 1	0.0095	0.0020	4.72	0.0090	-	+/-
P09769	FGR	FGR	Tyrosine-protein kinase Fgr	0.0002	-	+/-	-	-	-/-
Q5W0V3	FHI2A	FHIP2A	FHF complex subunit HOOK interacting protein 2A	-	-	-/-	0.0028	-	+/-
P49789	FHIT	FHIT	Bis(5'-adenosyl)-triphosphatase	0.0112	0.0181	0.62	-	-	-/-
Q13642	FHL1	FHL1	Four and a half LIM domains protein 1	0.0006	0.0004	1.67	-	-	-/-
Q13642-1	FHL1	FHL1	Isoform 1 of Four and a half LIM domains protein 1	0.0005	0.0011	0.46	0.0151	0.0127	1.19
Q14192	FHL2	FHL2	Four and a half LIM domains protein 2	0.0010	0.0023	0.42	-	-	-/-
Q13643	FHL3	FHL3	Four and a half LIM domains protein 3	0.0015	-	+/-	-	-	-/-
Q03591	FHR1	CFHR1	Complement factor H-related protein 1	0.0050	0.0047	1.05	0.0479	0.0241	1.99
P36980	FHR2	CFHR2	Complement factor H-related protein 2	0.0082	0.0032	2.57	0.0567	0.0087	6.50
Q02985	FHR3	CFHR3	Complement factor H-related protein 3	0.0028	0.0033	0.84	0.0233	-	+/-

Q92496	FHR4	CFHR4	Complement factor H-related protein 4	0.0010	-	+/-	-	-	-/-
Q9BXR6	FHR5	CFHR5	Complement factor H-related protein 5	0.0001	0.0012	0.09	0.0103	0.0033	3.14
P02671-2	FIBA	FGA	Isoform 2 of Fibrinogen alpha chain	0.0070	-	+/-	-	-	-/-
P02671	FIBA	FGA	Fibrinogen alpha chain	0.1501	0.0452	3.32	0.0746	0.0396	1.88
P02675	FIBB	FGB	Fibrinogen beta chain	0.2768	0.0643	4.31	0.3271	0.1368	2.39
P02679	FIBG	FGG	Fibrinogen gamma chain	0.4795	0.1230	3.90	0.2145	0.1621	1.32
P20930	FILA	FLG	Filaggrin	0.0002	0.0003	0.52	-	-	-/-
Q5D862	FILA2	FLG2	Filaggrin-2	0.0006	0.0009	0.64	-	-	-/-
P02751-1	FINC	FN1	Isoform 1 of Fibronectin	0.0210	0.0060	3.52	0.0665	0.0283	2.35
P02751	FINC	FN1	Fibronectin	0.1344	0.0247	5.44	0.0690	0.0301	2.29
Q9Y3D6	FIS1	FIS1	Mitochondrial fission 1 protein	0.0067	0.0213	0.32	0.0225	0.0242	0.93
Q96AY3	FKB10	FKBP10	Peptidyl-prolyl cis-trans isomerase FKBP10	0.0026	-	+/-	-	-	-/-
Q9NYL4	FKB11	FKBP11	Peptidyl-prolyl cis-trans isomerase FKBP11	0.0081	0.0032	2.56	0.0169	-	+/-
Q5T1M5	FKB15	FKBP15	FK506-binding protein 15	0.0001	-	+/-	-	-	-/-
P62942	FKB1A	FKBP1A	Peptidyl-prolyl cis-trans isomerase FKBP1A	0.0235	0.0094	2.51	0.0544	0.0161	3.39
P68106	FKB1B	FKBP1B	Peptidyl-prolyl cis-trans isomerase FKBP1B	-	0.0008	-/+	-	-	-/-
P26885	FKBP2	FKBP2	Peptidyl-prolyl cis-trans isomerase FKBP2	0.0143	0.0271	0.53	0.0431	0.0318	1.35
Q00688	FKBP3	FKBP3	Peptidyl-prolyl cis-trans isomerase FKBP3	0.0088	0.0074	1.19	0.0137	0.0080	1.71
Q02790	FKBP4	FKBP4	Peptidyl-prolyl cis-trans isomerase FKBP4	0.0178	0.0086	2.08	0.0145	0.0164	0.89
Q13451	FKBP5	FKBP5	Peptidyl-prolyl cis-trans isomerase FKBP5	0.0044	0.0024	1.84	0.0122	0.0091	1.33

Q9Y680	FKBP7	FKBP7	Peptidyl-prolyl cis-trans isomerase FKBP7	0.0011	0.0004	2.71	-	-	-/-
Q14318	FKBP8	FKBP8	Peptidyl-prolyl cis-trans isomerase FKBP8	0.0047	0.0022	2.15	0.0075	0.0078	0.97
O95302	FKBP9	FKBP9	Peptidyl-prolyl cis-trans isomerase FKBP9	0.0037	0.0017	2.20	0.0062	-	+/-
Q13045	FLII	FLII	Protein flightless-1 homolog	0.0002	0.0001	2.63	0.0065	0.0032	2.01
P21333	FLNA	FLNA	Filamin-A	0.0686	0.0209	3.28	0.0681	0.0360	1.89
O75369	FLNB	FLNB	Filamin-B	0.0528	0.0432	1.22	0.0373	0.0459	0.81
Q14315	FLNC	FLNC	Filamin-C	0.0035	0.0003	13.22	0.0005	-	+/-
O75955	FLOT1	FLOT1	Flotillin-1	0.0163	0.0096	1.69	0.0302	0.0285	1.06
Q14254	FLOT2	FLOT2	Flotillin-2	0.0174	0.0024	7.36	0.0364	0.0189	1.93
O95466	FMNL1	FMNL1	Formin-like protein 1	-	-	-/-	0.0015	-	+/-
P31513	FMO3	FMO3	Dimethylaniline monooxygenase [N-oxide-forming] 3	0.1082	0.0874	1.24	0.2154	0.3050	0.71
P31512	FMO4	FMO4	Dimethylaniline monooxygenase [N-oxide-forming] 4	0.0008	-	+/-	-	0.0076	-/+
P49326	FMO5	FMO5	Dimethylaniline monooxygenase [N-oxide-forming] 5	0.0446	0.0207	2.16	0.0509	0.0552	0.92
Q06828	FMOD	FMOD	Fibromodulin	0.0112	-	+/-	0.0016	-	+/-
Q06787	FMR1	FMR1	Synaptic functional regulator FMR1	0.0001	-	+/-	0.0032	-	+/-
Q06787-2	FMR1	FMR1	Isoform 1 of Synaptic functional regulator FMR1	0.0001	-	+/-	-	-	-/-
Q9H479	FN3K	FN3K	Fructosamine-3-kinase	0.0038	0.0088	0.43	-	0.0146	-/+

Q96RU3	FNBP1	FNBP1	Formin-binding protein 1	-	0.0001	-/+	0.0021	-	+/-
Q9Y2H6	FND3A	FNDC3A	Fibronectin type-III domain-containing protein 3A	0.0006	0.0001	5.11	0.0026	0.0017	1.47
Q53EP0	FND3B	FNDC3B	Fibronectin type III domain-containing protein 3B	-	-	-/-	0.0029	-	+/-
Q4ZHG4	FNDC1	FNDC1	Fibronectin type III domain-containing protein 1	0.0000	-	+/-	-	-	-/-
P49354	FNTA	FNTA	Protein farnesyltransferase/geranylgeranyltransferase type-1 subunit alpha	0.0014	0.0032	0.45	0.0047	-	+/-
P14324	FPPS	FDPS	Farnesyl pyrophosphate synthase	0.0110	0.0153	0.72	0.0188	0.0227	0.83
Q9P2B2	FPRP	PTGFRN	Prostaglandin F2 receptor negative regulator	0.0013	0.0001	9.21	0.0031	-	+/-
Q16595	FRDA	FXN	Frataxin, mitochondrial	0.0013	0.0097	0.13	-	-	-/-
Q5H8C1	FREM1	FREM1	FRAS1-related extracellular matrix protein 1	0.0001	-	+/-	-	-	-/-
Q14331	FRG1	FRG1	Protein FRG1	0.0001	-	+/-	-	-	-/-
P02794	FRIH	FTH1	Ferritin heavy chain	0.0449	0.0199	2.26	0.0310	0.0410	0.76
P02792	FRIL	FTL	Ferritin light chain	0.0959	0.0317	3.03	0.0545	0.0488	1.12
Q16658	FSCN1	FSCN1	Fascin	0.0121	0.0020	6.01	0.0168	0.0158	1.06
Q9BRQ8	FSP1	AIFM2	Ferroptosis suppressor protein 1	0.0025	0.0003	9.30	0.0141	-	+/-
O95954-2	FTCD	FTCD	Isoform C of Formimidoyltransferase-cyclodeaminase	0.0002	-	+/-	-	-	-/-
O95954	FTCD	FTCD	Formimidoyltransferase-cyclodeaminase	0.0632	0.1502	0.42	0.0553	0.1505	0.37

Q9C0B1	FTO	FTO	Alpha-ketoglutarate-dependent dioxygenase FTO	0.0001	0.0005	0.23	-	-	-/-
Q96AE4	FUBP1	FUBP1	Far upstream element-binding protein 1	0.0078	0.0032	2.45	0.0095	-	+/-
Q96AE4-2	FUBP1	FUBP1	Isoform 2 of Far upstream element-binding protein 1	0.0009	-	+/-	-	-	-/-
Q92945	FUBP2	KHSRP	Far upstream element-binding protein 2	0.0057	0.0043	1.32	0.0161	0.0086	1.88
Q96I24	FUBP3	FUBP3	Far upstream element-binding protein 3	0.0058	0.0053	1.10	0.0071	0.0074	0.97
A2VDF0	FUCM	FUOM	Fucose mutarotase	0.0086	0.0229	0.38	0.0124	0.0326	0.38
P04066	FUCO	FUCA1	Tissue alpha-L-fucosidase	0.0051	0.0023	2.22	0.0310	0.0138	2.26
Q9BTY2	FUCO2	FUCA2	Plasma alpha-L-fucosidase	0.0002	-	+/-	-	-	-/-
P07954	FUMH	FH	Fumarate hydratase, mitochondrial	0.0615	0.0890	0.69	0.0777	0.1089	0.71
Q9BWH2	FUND2	FUNDC2	FUN14 domain-containing protein 2	0.0011	0.0004	2.73	-	-	-/-
P35637	FUS	FUS	RNA-binding protein FUS	0.0022	0.0009	2.52	0.0081	0.0061	1.32
P51114	FXR1	FXR1	Fragile X mental retardation syndrome-related protein 1	0.0013	0.0001	10.85	0.0042	0.0033	1.31
P51116	FXR2	FXR2	Fragile X mental retardation syndrome-related protein 2	-	-	-/-	0.0032	-	+/-
Q9BQS8	FYCO1	FYCO1	FYVE and coiled-coil domain-containing protein 1	0.0011	0.0007	1.59	0.0011	-	+/-
Q9Y2I7	FYV1	PIKFYVE	1-phosphatidylinositol 3-phosphate 5-kinase	-	-	-/-	0.0002	-	+/-
Q13283	G3BP1	G3BP1	Ras GTPase-activating protein-binding protein 1	0.0066	0.0014	4.66	0.0168	0.0087	1.93

Q9UN86-2	G3BP2	G3BP2	Isoform B of Ras GTPase-activating protein-binding protein 2	-	0.0002	-/+	-	-	-/-
Q9UN86	G3BP2	G3BP2	Ras GTPase-activating protein-binding protein 2	0.0008	0.0002	3.43	0.0034	0.0008	4.25
P04406	G3P	GAPDH	Glyceraldehyde-3-phosphate dehydrogenase	0.4675	1.1184	0.42	0.6226	1.1451	0.54
O14556	G3PT	GAPDHS	Glyceraldehyde-3-phosphate dehydrogenase, testis-specific	0.0004	-	+/-	-	-	-/-
P35575	G6PC1	G6PC1	Glucose-6-phosphatase catalytic subunit 1	-	-	-/-	-	0.0203	-/+
P11413	G6PD	G6PD	Glucose-6-phosphate 1-dehydrogenase	0.0136	-	+/-	0.0392	0.0089	4.41
O95479	G6PE	H6PD	GDH/6PGL endoplasmic bifunctional protein	0.0311	0.0215	1.45	0.0717	0.0761	0.94
P06744	G6PI	GPI	Glucose-6-phosphate isomerase	0.0482	0.0598	0.81	0.1032	0.1088	0.95
O43826	G6PT1	SLC37A4	Glucose-6-phosphate exchanger SLC37A4	0.0019	0.0007	2.66	0.0105	0.0094	1.11
Q13480	GAB1	GAB1	GRB2-associated-binding protein 1	-	-	-/-	0.0030	-	+/-
Q06546	GABPA	GABPA	GA-binding protein alpha chain	-	0.0002	-/+	-	-	-/-
P80404	GABT	ABAT	4-aminobutyrate aminotransferase, mitochondrial	0.0648	0.1399	0.46	0.0958	0.2012	0.48
O14976	GAK	GAK	Cyclin-G-associated kinase	0.0001	-	+/-	0.0024	-	+/-
P0DPI2	GAL3A	GATD3	Glutamine amidotransferase-like class 1 domain-containing protein 3, mitochondrial	0.0182	0.0113	1.61	0.0112	0.0526	0.21
A0A0B4J2D5	GAL3B	GATD3B	Glutamine amidotransferase-like class 1 domain-containing protein 3B, mitochondrial	0.0214	0.0363	0.59	-	0.0071	-/+
P54803	GALC	GALC	Galactocerebrosidase	0.0002	-	+/-	-	-	-/-

Q8NB37	GALD1	GATD1	Glutamine amidotransferase-like class 1 domain-containing protein 1	0.0043	0.0059	0.73	0.0143	-	+/-
Q14376	GALE	GALE	UDP-glucose 4-epimerase	0.0113	0.0307	0.37	0.0336	0.0649	0.52
P51570-2	GALK1	GALK1	Isoform 2 of Galactokinase	0.0008	-	+/-	-	-	-/-
P51570	GALK1	GALK1	Galactokinase	0.0357	0.0357	1.00	0.0297	0.0400	0.74
Q01415	GALK2	GALK2	N-acetylgalactosamine kinase	-	0.0002	-/+	0.0043	0.0068	0.63
Q96C23	GALM	GALM	Aldose 1-epimerase	0.0147	0.0287	0.51	0.0215	0.0405	0.53
P34059	GALNS	GALNS	N-acetylgalactosamine-6-sulfatase	0.0007	0.0017	0.43	0.0050	-	+/-
P07902	GALT	GALT	Galactose-1-phosphate uridylyltransferase	0.0034	0.0142	0.24	0.0079	0.0299	0.27
Q10472	GALT1	GALNT1	Polypeptide N-acetylgalactosaminyltransferase 1	0.0004	-	+/-	-	-	-/-
Q10471	GALT2	GALNT2	Polypeptide N-acetylgalactosaminyltransferase 2	0.0042	0.0001	32.80	0.0348	0.0066	5.24
Q14353	GAMT	GAMT	Guanidinoacetate N-methyltransferase	0.0251	0.0739	0.34	0.0494	0.1409	0.35
Q14697	GANAB	GANAB	Neutral alpha-glucosidase AB	0.0943	0.0542	1.74	0.1095	0.0861	1.27
Q14697-2	GANAB	GANAB	Isoform 2 of Neutral alpha-glucosidase AB	0.0210	0.0043	4.93	0.0846	0.0748	1.13
Q14C86	GAPD1	GAPVD1	GTPase-activating protein and VPS9 domain-containing protein 1	-	-	-/-	0.0012	-	+/-
Q9H4G4	GAPR1	GLIPR2	Golgi-associated plant pathogenesis-related protein 1	0.0015	0.0024	0.62	0.0291	-	+/-
Q9NY12	GAR1	GAR1	H/ACA ribonucleoprotein complex subunit 1	0.0008	-	+/-	0.0053	-	+/-
P41250	GARS	GARS	Glycine--tRNA ligase	0.0050	0.0015	3.35	0.0239	0.0103	2.33

O43903	GAS2	GAS2	Growth arrest-specific protein 2	0.0005	0.0013	0.39	-	0.0070	-/+
Q9H0R6	GATA	QRSL1	Glutamyl-tRNA(Gln) amidotransferase subunit A, mitochondrial	-	0.0009	-/+	-	-	-/-
O43716	GATC	GATC	Glutamyl-tRNA(Gln) amidotransferase subunit C, mitochondrial	0.0009	0.0083	0.11	-	-	-/-
P50440	GATM	GATM	Glycine amidinotransferase, mitochondrial	0.0643	0.1165	0.55	0.1534	0.2563	0.60
Q9H227	GBA3	GBA3	Cytosolic beta-glucosidase	-	0.0017	-/+	-	0.0068	-/+
P62873	GBB1	GNB1	Guanine nucleotide-binding protein G(I)/G(T) subunit beta-1	0.0363	0.0185	1.96	0.0350	0.0329	1.07
P62879	GBB2	GNB2	Guanine nucleotide-binding protein G(I)/G(T) subunit beta-2	0.0232	0.0119	1.96	0.0665	0.0568	1.17
P16520	GBB3	GNB3	Guanine nucleotide-binding protein G(I)/GS)/G(T) subunit beta-3	-	0.0003	-/+	-	-	-/-
Q9HAV0	GBB4	GNB4	Guanine nucleotide-binding protein subunit beta-4	0.0059	0.0050	1.17	0.0173	0.0192	0.90
Q92538	GBF1	GBF1	Golgi-specific brefeldin A-resistance guanine nucleotide exchange factor 1	-	-	-/-	0.0016	-	+/-
Q9UBI6	GBG12	GNG12	Guanine nucleotide-binding protein G(I)/G(S)/G(O) subunit gamma-12	0.0015	0.0031	0.49	0.0186	-	+/-
P32455	GBP1	GBP1	Guanylate-binding protein 1	0.0011	0.0006	1.82	0.0063	0.0093	0.67
P32456	GBP2	GBP2	Guanylate-binding protein 2	-	-	-/-	0.0050	-	+/-
Q9H0R5	GBP3	GBP3	Guanylate-binding protein 3	-	-	-/-	0.0024	-	+/-

O95166	GBRAP	GABARAP	Gamma-aminobutyric acid receptor-associated protein	0.0019	-	+/-	0.0098	-	+/-
Q9H0R8	GBRL1	GABARAPL1	Gamma-aminobutyric acid receptor-associated protein-like 1	0.0017	-	+/-	-	-	-/-
P60520	GBRL2	GABARAPL2	Gamma-aminobutyric acid receptor-associated protein-like 2	0.0026	-	+/-	0.0175	-	+/-
Q8IWJ2-3	GCC2	GCC2	Isoform 2 of GRIP and coiled-coil domain-containing protein 2	0.0009	-	+/-	-	-	-/-
Q92947	GCDH	GCDH	Glutaryl-CoA dehydrogenase, mitochondrial	0.0113	0.0486	0.23	0.0261	0.0664	0.39
P30793	GCH1	GCH1	GTP cyclohydrolase 1	-	0.0006	-/+	-	-	-/-
Q14397	GCKR	GCKR	Glucokinase regulatory protein	-	0.0002	-/+	-	0.0087	-/+
Q92616	GCN1	GCN1	eIF-2-alpha kinase activator GCN1	0.0003	0.0000	9.01	0.0103	0.0050	2.03
Q9P109	GCNT4	GCNT4	Beta-1,3-galactosyl-O-glycosyl-glycoprotein beta-1,6-N-acetylglucosaminyltransferase 4	0.0015	-	+/-	-	-	-/-
Q96CW5	GCP3	TUBGCP3	Gamma-tubulin complex component 3	-	-	-/-	0.0017	-	+/-
Q9H3P7	GCP60	ACBD3	Golgi resident protein GCP60	0.0025	0.0016	1.55	0.0044	-	+/-
Q8N6F7	GCSAM	GCSAM	Germinal center-associated signaling and motility protein	0.0049	-	+/-	-	-	-/-
P23434	GCSH	GCSH	Glycine cleavage system H protein, mitochondrial	0.0049	0.0167	0.29	-	0.0029	-/+
P23378	GCSP	GLDC	Glycine dehydrogenase (decarboxylating), mitochondrial	0.0097	0.0291	0.33	0.0125	0.0616	0.20
P48728	GCST	AMT	Aminomethyltransferase, mitochondrial	0.0059	0.0077	0.76	-	0.0193	-/+

Q02153	GCYB1	GUCY1B1	Guanylate cyclase soluble subunit beta-1	-	-	-/-	0.0045	-	+/-
Q8TB36	GDAP1	GDAP1	Ganglioside-induced differentiation-associated protein 1	0.0007	-	+/-	-	-	-/-
P35573	GDE	AGL	Glycogen debranching enzyme	0.0218	0.0289	0.75	0.0504	0.1092	0.46
P31150	GDIA	GDI1	Rab GDP dissociation inhibitor alpha	0.0219	0.0224	0.97	0.0515	0.0428	1.20
P50395	GDIB	GDI2	Rab GDP dissociation inhibitor beta	0.0436	0.0631	0.69	0.1425	0.1472	0.97
P52565	GDIR1	ARHGDIA	Rho GDP-dissociation inhibitor 1	0.0513	0.0354	1.45	0.0363	0.0340	1.07
P52566	GDIR2	ARHGDIB	Rho GDP-dissociation inhibitor 2	0.0616	0.0389	1.58	0.0618	0.0372	1.66
P07093	GDN	SERPINE2	Glia-derived nexin	0.0005	-	+/-	0.0100	-	+/-
Q6ZNW5	GDPP1	GDPGP1	GDP-D-glucose phosphorylase 1	0.0004	-	+/-	-	-	-/-
P52306	GDS1	RAP1GDS1	Rap1 GTPase-GDP dissociation stimulator 1	0.0005	0.0009	0.59	0.0032	-	+/-
P06396	GELS	GSN	Gelsolin	0.0552	0.0128	4.32	0.0488	0.0183	2.67
P57678	GEMI4	GEMIN4	Gem-associated protein 4	-	-	-/-	0.0019	-	+/-
Q8TEQ6	GEMI5	GEMIN5	Gem-associated protein 5	0.0000	-	+/-	-	-	-/-
Q8WXD5	GEMI6	GEMIN6	Gem-associated protein 6	0.0032	0.0029	1.10	-	-	-/-
Q9NQX3	GEPH	GPHN	Gephyrin	0.0010	0.0051	0.19	-	0.0091	-/+
P14136	GFAP	GFAP	Glial fibrillary acidic protein	-	0.0004	-/+	-	-	-/-
Q3B7J2	GFOD2	GFOD2	Glucose-fructose oxidoreductase domain-containing protein 2	-	0.0003	-/+	-	-	-/-
Q06210	GFPT1	GFPT1	Glutamine--fructose-6-phosphate aminotransferase [isomerizing] 1	0.0062	0.0025	2.45	0.0424	0.0180	2.36

P30047	GFRP	GCHFR	GTP cyclohydrolase 1 feedback regulatory protein	0.0488	0.1245	0.39	-	0.0360	-/+
Q8N7Z2	GG6L1	GOLGA6L1	Golgin subfamily A member 6-like protein 1	-	-	-/-	0.0014	-	+/-
Q9BVM4	GGACT	GGACT	Gamma-glutamylaminecyclotransferase	0.0025	0.0093	0.27	-	-	-/-
O75223	GGCT	GGCT	Gamma-glutamylcyclotransferase	0.0217	0.0360	0.60	0.0098	0.0227	0.43
Q92820	GGH	GGH	Gamma-glutamyl hydrolase	0.0762	0.0188	4.05	0.1200	0.0358	3.35
O95749	GGPPS	GGPS1	Geranylgeranyl pyrophosphate synthase	0.0008	0.0005	1.73	0.0056	-	+/-
P19440	GGT1	GGT1	Glutathione hydrolase 1 proenzyme	0.0069	0.0004	15.36	0.0077	0.0059	1.32
P36269	GGT5	GGT5	Glutathione hydrolase 5 proenzyme	0.0311	0.0245	1.27	0.0344	0.0410	0.84
Q6Y7W6	GGYF2	GIGYF2	GRB10-interacting GYF protein 2	0.0000	-	+/-	-	-	-/-
Q9H936	GHC1	SLC25A22	Mitochondrial glutamate carrier 1	0.0013	0.0004	3.19	0.0081	0.0109	0.74
Q9H1K4	GHC2	SLC25A18	Mitochondrial glutamate carrier 2	0.0093	0.0064	1.45	0.0142	0.0172	0.83
Q9H3K2	GHITM	GHITM	Growth hormone-inducible transmembrane protein	0.0022	0.0005	4.48	-	-	-/-
Q9NWU2	GID8	GID8	Glucose-induced degradation protein 8 homolog	0.0010	0.0014	0.75	-	-	-/-
P13284	GILT	IFI30	Gamma-interferon-inducible lysosomal thiol reductase	0.0022	-	+/-	0.0033	-	+/-
Q8WWP7	GIMA1	GIMAP1	GTPase IMAP family member 1	0.0006	-	+/-	0.0045	-	+/-
Q9NUV9	GIMA4	GIMAP4	GTPase IMAP family member 4	0.0007	0.0007	1.10	0.0074	0.0056	1.32
O14908	GIPC1	GIPC1	PDZ domain-containing protein GIPC1	0.0010	0.0017	0.59	-	-	-/-

Q8TF65	GIPC2	GIPC2	PDZ domain-containing protein GIPC2	0.0001	0.0024	0.05	-	0.0065	-/+
Q8TF64	GIPC3	GIPC3	PDZ domain-containing protein GIPC3	-	0.0003	-/+	-	-	-/-
Q9Y2X7	GIT1	GIT1	ARF GTPase-activating protein GIT1	-	0.0001	-/+	-	-	-/-
Q14161	GIT2	GIT2	ARF GTPase-activating protein GIT2	-	-	-/-	0.0022	-	+/-
O94923	GLCE	GLCE	D-glucuronyl C5-epimerase	0.0001	-	+/-	-	-	-/-
P04062	GLCM	GBA	Lysosomal acid glucosylceramidase	0.0053	0.0002	23.83	0.0116	-	+/-
Q9Y223	GLCNE	GNE	Bifunctional UDP-N-acetylglucosamine 2-epimerase/N-acetylmannosamine kinase	0.0009	0.0005	2.03	0.0096	0.0083	1.15
Q8IVS8	GLCTK	GLYCTK	Glycerate kinase	0.0020	0.0083	0.24	-	0.0049	-/+
Q04446	GLGB	GBE1	1,4-alpha-glucan-branching enzyme	0.0102	0.0158	0.64	0.0624	0.0760	0.82
P15104	GLNA	GLUL	Glutamine synthetase	0.0063	0.0108	0.58	0.0192	0.0234	0.82
Q16775	GLO2	HAGH	Hydroxyacylglutathione hydrolase, mitochondrial	0.0153	0.0262	0.58	0.0204	0.0344	0.59
Q9HC38-2	GLOD4	GLOD4	Isoform 2 of Glyoxalase domain-containing protein 4	0.0012	-	+/-	-	-	-/-
Q9HC38	GLOD4	GLOD4	Glyoxalase domain-containing protein 4	0.0234	0.0341	0.68	0.0180	0.0405	0.45
A6NK44	GLOD5	GLOD5	Glyoxalase domain-containing protein 5	-	0.0005	-/+	-	-	-/-
Q9H4A5	GLP3L	GOLPH3L	Golgi phosphoprotein 3-like	0.0005	0.0003	2.06	-	-	-/-
P32189	GLPK	GK	Glycerol kinase	0.0017	0.0016	1.07	0.0152	0.0107	1.42
P35754	GLRX1	GLRX	Glutaredoxin-1	0.0113	0.0308	0.37	0.0348	0.0263	1.32
O76003	GLRX3	GLRX3	Glutaredoxin-3	0.0144	0.0095	1.51	0.0118	0.0142	0.83

Q86SX6	GLRX5	GLRX5	Glutaredoxin-related protein 5, mitochondrial	0.0157	0.0141	1.11	-	-	-/-
O94925	GLSK	GLS	Glutaminase kidney isoform, mitochondrial	0.0010	0.0002	6.30	0.0135	-	+/-
Q9UI32	GLSL	GLS2	Glutaminase liver isoform, mitochondrial	0.0004	0.0042	0.09	-	0.0295	-/+
Q6IS24	GLT17	GALNT17	Polypeptide N-acetylgalactosaminyltransferase 17	-	-	-/-	-	0.0025	-/+
P14314	GLU2B	PRKCSH	Glucosidase 2 subunit beta	0.0314	0.0427	0.74	0.0494	0.0366	1.35
Q6IB77	GLYAT	GLYAT	Glycine N-acyltransferase	0.0728	0.2805	0.26	0.0586	0.2959	0.20
P34896	GLYC	SHMT1	Serine hydroxymethyltransferase, cytosolic	0.0393	0.1079	0.36	0.0413	0.1008	0.41
P46976	GLYG	GYG1	Glycogenin-1	0.0002	0.0004	0.61	0.0107	0.0111	0.97
O15488	GLYG2	GYG2	Glycogenin-2	-	0.0003	-/+	-	0.0058	-/+
Q969I3	GLYL1	GLYATL1	Glycine N-acyltransferase-like protein 1	0.0098	0.0145	0.68	0.0084	0.0233	0.36
P34897	GLYM	SHMT2	Serine hydroxymethyltransferase, mitochondrial	0.0497	0.0479	1.04	0.0426	0.0580	0.73
Q49A26	GLYR1	GLYR1	Putative oxidoreductase GLYR1	0.0005	0.0005	1.02	0.0026	-	+/-
O60547	GMDS	GMDS	GDP-mannose 4,6 dehydratase	0.0022	0.0007	3.21	0.0213	-	+/-
Q9UKD1	GMEB2	GMEB2	Glucocorticoid modulatory element-binding protein 2	0.0002	0.0009	0.29	-	-	-/-
P60983	GMFB	GMFB	Glia maturation factor beta	0.0219	0.0063	3.47	0.0147	0.0096	1.53
O60234	GMFG	GMFG	Glia maturation factor gamma	0.0113	0.0118	0.96	0.0103	-	+/-
Q96IJ6	GMPPA	GMPPA	Mannose-1-phosphate guanyltransferase alpha	0.0081	0.0045	1.79	0.0128	0.0172	0.75

Q9Y5P6	GMPPB	GMPPB	Mannose-1-phosphate guanyltransferase beta	0.0095	0.0073	1.30	0.0310	0.0376	0.83
Q9P2T1	GMPR2	GMPR2	GMP reductase 2	0.0013	0.0019	0.71	0.0075	0.0050	1.49
Q96EK6	GNA1	GNPNAT1	Glucosamine 6-phosphate N-acetyltransferase	0.0117	0.0310	0.38	0.0460	0.0694	0.66
P29992	GNA11	GNA11	Guanine nucleotide-binding protein subunit alpha-11	0.0015	0.0003	5.84	0.0081	0.0061	1.32
Q14344	GNA13	GNA13	Guanine nucleotide-binding protein subunit alpha-13	-	-	-/-	0.0097	0.0037	2.58
P63096	GNAI1	GNAI1	Guanine nucleotide-binding protein G(i) subunit alpha-1	0.0008	0.0016	0.48	0.0155	0.0134	1.16
P04899	GNAI2	GNAI2	Guanine nucleotide-binding protein G(i) subunit alpha-2	0.0332	0.0133	2.49	0.0735	0.0610	1.20
P08754	GNAI3	GNAI3	Guanine nucleotide-binding protein G(i) subunit alpha	0.0042	0.0043	0.97	0.0200	0.0143	1.40
P09471	GNAO	GNAO1	Guanine nucleotide-binding protein G (o) subunit alpha	0.0005	0.0024	0.22	-	0.0072	-/+
P50148	GNAQ	GNAQ	Guanine nucleotide-binding protein G (q) subunit alpha	0.0008	0.0010	0.75	0.0059	-	+/-
Q5JWF2	GNAS1	GNAS	Guanine nucleotide-binding protein G(s) subunit alpha isoforms XLas	-	-	-/-	0.0079	-	+/-
P63092	GNAS2	GNAS	Guanine nucleotide-binding protein G(s) subunit alpha isoforms short	0.0021	0.0021	0.98	0.0180	0.0140	1.28
P36915	GNL1	GNL1	Guanine nucleotide-binding protein-like 1	-	-	-/-	0.0051	-	+/-
Q14749	GNMT	GNMT	Glycine N-methyltransferase	0.0215	0.0287	0.75	0.0324	0.0429	0.75

O15228	GNPAT	GNPAT	Dihydroxyacetone phosphate acyltransferase	-	-	-/-	0.0087	-	+/-
P46926	GNPI1	GNPDA1	Glucosamine-6-phosphate isomerase 1	0.0163	0.0037	4.36	0.0251	0.0096	2.61
Q8TDQ7	GNPI2	GNPDA2	Glucosamine-6-phosphate isomerase 2	0.0008	-	+/-	-	-	-/-
P15586	GNS	GNS	N-acetylglucosamine-6-sulfatase	0.0039	-	+/-	0.0159	-	+/-
Q5T6J7	GNTK	IDNK	Probable gluconokinase	0.0024	0.0048	0.51	-	0.0096	-/+
Q08379	GOGA2	GOLGA2	Golgin subfamily A member 2	0.0010	0.0011	0.95	-	-	-/-
Q13439	GOGA4	GOLGA4	Golgin subfamily A member 4	-	-	-/-	-	0.0005	-/+
Q14789	GOGB1	GOLGB1	Golgin subfamily B member 1	0.0010	0.0001	10.38	-	-	-/-
Q8NBJ4	GOLM1	GOLM1	Golgi membrane protein 1	-	-	-/-	0.0076	-	+/-
Q9H4A6	GOLP3	GOLPH3	Golgi phosphoprotein 3	0.0016	0.0010	1.60	0.0044	-	+/-
Q9HD26	GOPC	GOPC	Golgi-associated PDZ and coiled-coil motif-containing protein	0.0007	0.0004	1.83	-	-	-/-
Q9BQQ3	GORS1	GORASP1	Golgi reassembly-stacking protein 1	0.0009	-	+/-	-	-	-/-
Q9H8Y8	GORS2	GORASP2	Golgi reassembly-stacking protein 2	0.0332	0.0343	0.97	0.0560	0.0101	5.54
O95249	GOSR1	GOSR1	Golgi SNAP receptor complex member 1	0.0001	-	+/-	0.0053	0.0055	0.97
O14653	GOSR2	GOSR2	Golgi SNAP receptor complex member 2	-	-	-/-	0.0107	-	+/-
Q9Y3E0	GOT1B	GOLT1B	Vesicle transport protein GOT1B	-	0.0009	-/+	-	-	-/-
Q5VW38	GP107	GPR107	Protein GPR107	0.0003	-	+/-	-	-	-/-
O14626	GP171	GPR171	Probable G-protein coupled receptor 171	0.0005	-	+/-	-	-	-/-
Q86V85	GP180	GPR180	Integral membrane protein GPR180	0.0006	-	+/-	-	-	-/-

P07359	GP1BA	GP1BA	Platelet glycoprotein Ib alpha chain	0.0004	-	+/-	-	-	-/-
O43292	GPAA1	GPAA1	Glycosylphosphatidylinositol anchor attachment 1 protein	-	-	-/-	0.0098	-	+/-
Q9HCL2	GPAT1	GPAM	Glycerol-3-phosphate acyltransferase 1, mitochondrial	-	0.0001	-/+	0.0033	0.0034	0.97
P35052	GPC1	GPC1	Glypican-1	0.0003	-	+/-	-	-	-/-
P51654	GPC3	GPC3	Glypican-3	-	-	-/-	0.0050	-	+/-
Q9Y625	GPC6	GPC6	Glypican-6	0.0003	0.0002	1.78	0.0073	-	+/-
Q8N335	GPD1L	GPD1L	Glycerol-3-phosphate dehydrogenase 1-like protein	0.0003	0.0006	0.55	-	-	-/-
P21695	GPDA	GPD1	Glycerol-3-phosphate dehydrogenase [NAD(+)], cytoplasmic	0.0628	0.1342	0.47	0.0779	0.2419	0.32
P43304	GPDM	GPD2	Glycerol-3-phosphate dehydrogenase, mitochondrial	0.0012	0.0002	6.38	0.0044	-	+/-
B7ZAQ6-2	GPHRA	GPR89A	Isoform 2 of Golgi pH regulator A	-	-	-/-	0.0058	-	+/-
B7ZAQ6	GPHRA	GPR89A	Golgi pH regulator A	-	-	-/-	0.0071	0.0048	1.49
Q14956	GPNMB	GPNMB	Transmembrane glycoprotein NMB	0.0003	0.0005	0.71	0.0063	0.0042	1.49
P07203	GPX1	GPX1	Glutathione peroxidase 1	0.0747	0.0900	0.83	0.1613	0.2220	0.73
P18283	GPX2	GPX2	Glutathione peroxidase 2	0.0316	0.0282	1.12	0.1066	0.0144	7.38
P22352	GPX3	GPX3	Glutathione peroxidase 3	0.0182	0.0123	1.48	0.0330	0.0138	2.40
P36969	GPX4	GPX4	Phospholipid hydroperoxide glutathione peroxidase	0.0124	0.0195	0.64	0.0222	0.0289	0.77

Q96SL4	GPX7	GPX7	Glutathione peroxidase 7	0.0010	-	+/-	-	-	-/-
P12544	GRAA	GZMA	Granzyme A	0.0010	0.0007	1.53	-	-	-/-
P49863	GRAK	GZMK	Granzyme K	0.0007	0.0009	0.78	0.0103	-	+/-
Q6IC98	GRAM4	GRAMD4	GRAM domain-containing protein 4	-	-	-/-	0.0023	-	+/-
P28676	GRAN	GCA	Grancalcin	0.0462	0.0148	3.13	0.0489	0.0193	2.53
Q4V328	GRAP1	GRIPAP1	GRIP1-associated protein 1	0.0016	0.0036	0.44	-	-	-/-
O75791	GRAP2	GRAP2	GRB2-related adapter protein 2	0.0002	-	+/-	-	-	-/-
P62993	GRB2	GRB2	Growth factor receptor-bound protein 2	0.0361	0.0089	4.07	-	-	-/-
Q6ISB3	GRHL2	GRHL2	Grainyhead-like protein 2 homolog	-	-	-/-	-	0.0024	-/+
Q9UBQ7	GRHPR	GRHPR	Glyoxylate reductase/hydroxypyruvate reductase	0.0986	0.2760	0.36	0.0956	0.2347	0.41
P42262	GRIA2	GRIA2	Glutamate receptor 2	-	0.0001	-/+	-	-	-/-
P38646	GRP75	HSPA9	Stress-70 protein, mitochondrial	0.1157	0.0548	2.11	0.0611	0.0582	1.05
Q9HAV7	GRPE1	GRPEL1	GrpE protein homolog 1, mitochondrial	0.0106	0.0058	1.82	0.0060	0.0062	0.97
Q12849	GRSF1	GRSF1	G-rich sequence factor 1	0.0007	0.0004	1.60	-	-	-/-
Q9BQ67	GRWD1	GRWD1	Glutamate-rich WD repeat-containing protein 1	0.0020	0.0018	1.14	0.0116	-	+/-
P57764	GSDMD	GSDMD	Gasdermin-D	0.0006	0.0005	1.16	0.0109	-	+/-
O60443	GSDME	GSDME	Gasdermin-E	-	-	-/-	0.0040	-	+/-
P48507	GSH0	GCLM	Glutamate--cysteine ligase regulatory subunit	0.0122	0.0035	3.51	0.0194	0.0111	1.75
P48506	GSH1	GCLC	Glutamate--cysteine ligase catalytic subunit	0.0027	0.0028	0.96	0.0202	0.0298	0.68

P48637	GSHB	GSS	Glutathione synthetase	0.0142	0.0142	1.00	0.0239	0.0279	0.86
P00390	GSHR	GSR	Glutathione reductase, mitochondrial	0.0162	0.0099	1.63	0.0215	0.0174	1.24
P49840	GSK3A	GSK3A	Glycogen synthase kinase-3 alpha	-	-	-/-	0.0042	-	+/-
Q9P0R6	GSKIP	GSKIP	GSK3B-interacting protein	-	0.0019	-/+	-	-	-/-
Q92896	GSLG1	GLG1	Golgi apparatus protein 1	0.0008	-	+/-	0.0023	-	+/-
P0CG29	GST2	GSTT2	Glutathione S-transferase theta-2	0.0009	0.0028	0.31	0.0222	0.0175	1.27
P08263	GSTA1	GSTA1	Glutathione S-transferase A1	0.1381	0.5786	0.24	0.0844	0.2774	0.30
P09210	GSTA2	GSTA2	Glutathione S-transferase A2	-	0.0666	-/+	-	-	-/-
Q16772	GSTA3	GSTA3	Glutathione S-transferase A3	-	0.0127	-/+	-	-	-/-
O15217	GSTA4	GSTA4	Glutathione S-transferase A4	0.0008	0.0019	0.40	-	-	-/-
Q7RTV2	GSTA5	GSTA5	Glutathione S-transferase A5	-	-	-/-	-	0.0030	-/+
Q9Y2Q3	GSTK1	GSTK1	Glutathione S-transferase kappa 1	0.1765	0.2477	0.71	0.1770	0.1842	0.96
P09488	GSTM1	GSTM1	Glutathione S-transferase Mu 1	0.0769	0.2194	0.35	0.0642	0.1463	0.44
P28161	GSTM2	GSTM2	Glutathione S-transferase Mu 2	0.0056	0.0145	0.39	-	0.0337	-/+
P21266	GSTM3	GSTM3	Glutathione S-transferase Mu 3	0.0360	0.0411	0.88	0.0437	0.0399	1.09
Q03013	GSTM4	GSTM4	Glutathione S-transferase Mu 4	0.0015	0.0197	0.08	0.0245	0.1167	0.21
P46439	GSTM5	GSTM5	Glutathione S-transferase Mu 5	0.0010	0.0021	0.46	-	-	-/-
P78417	GSTO1	GSTO1	Glutathione S-transferase omega-1	0.1410	0.1451	0.97	0.0753	0.1201	0.63
P09211	GSTP1	GSTP1	Glutathione S-transferase P	0.1405	0.1016	1.38	0.1529	0.1036	1.48
P30711	GSTT1	GSTT1	Glutathione S-transferase theta-1	0.0092	0.0213	0.43	0.0266	0.0762	0.35

P0CG30	GSTT2	GSTT2B	Glutathione S-transferase theta-2B	0.0014	0.0052	0.28	-	0.0241	-/+
Q8NBJ5	GT251	COLGALT1	Procollagen galactosyltransferase 1	0.0028	0.0004	6.51	0.0075	-	+/-
P78347	GTF2I	GTF2I	General transcription factor II-I	0.0018	0.0005	3.95	0.0070	0.0039	1.81
P11166	GTR1	SLC2A1	Solute carrier family 2, facilitated glucose transporter member 1	0.0008	-	+/-	0.0013	0.0014	0.92
P11168	GTR2	SLC2A2	Solute carrier family 2, facilitated glucose transporter member 2	0.0080	0.0073	1.10	0.0204	0.0344	0.59
P49915	GUAA	GMPS	GMP synthase [glutamine-hydrolyzing]	0.0002	0.0009	0.18	0.0063	0.0051	1.23
Q9Y2T3-3	GUAD	GDA	Isoform 3 of Guanine deaminase	-	0.0080	-/+	-	-	-/-
Q9Y2T3	GUAD	GDA	Guanine deaminase	0.0131	0.0154	0.85	0.0298	0.0633	0.47
P13807	GYS1	GYS1	Glycogen [starch] synthase, muscle	0.0002	-	+/-	0.0024	0.0038	0.63
P54840	GYS2	GYS2	Glycogen [starch] synthase, liver	0.0021	0.0064	0.33	0.0087	0.0362	0.24
P07305	H10	H1F0	Histone H1.0	0.0011	-	+/-	0.0082	0.0083	0.99
P16403	H12	HIST1H1C	Histone H1.2	0.0020	-	+/-	0.0017	0.0020	0.87
P16402	H13	H1-3	Histone H1.3	0.0004	-	+/-	-	0.0092	-/+
P10412	H14	HIST1H1E	Histone H1.4	0.0106	0.0066	1.61	0.0227	0.0117	1.95
P16401	H15	HIST1H1B	Histone H1.5	0.0039	-	+/-	0.0091	-	+/-
O14756	H17B6	HSD17B6	17-beta-hydroxysteroid dehydrogenase type 6	0.0590	0.0530	1.11	0.1149	0.1480	0.78
Q53T59	H1BP3	HS1BP3	HCLS1-binding protein 3	0.0086	0.0117	0.74	0.0053	0.0055	0.97
P22492	H1T	HIST1H1T	Histone H1t	0.0001	-	+/-	-	-	-/-
Q92522	H1X	H1FX	Histone H1x	0.0002	-	+/-	-	-	-/-

P0C0S8	H2A1	HIST1H2AG	Histone H2A type 1	0.0054	0.0181	0.30	-	0.0080	-/+
P04908	H2A1B	HIST1H2AB	Histone H2A type 1-B/E	0.0090	0.0555	0.16	0.0206	0.0098	2.10
Q16777	H2A2C	HIST2H2AC	Histone H2A type 2-C	0.0048	0.0166	0.29	0.0372	0.0260	1.43
Q9P0M6	H2AW	MACROH2A2	Core histone macro-H2A.2	0.0003	0.0002	2.04	0.0065	0.0032	2.01
P16104	H2AX	H2AFX	Histone H2AX	0.0013	0.0107	0.12	0.0028	0.0032	0.87
O75367	H2AY	H2AFY	Core histone macro-H2A.1	0.0095	0.0025	3.74	0.0260	0.0127	2.05
P0C0S5	H2AZ	H2AZ1	Histone H2A.Z	0.0029	0.0050	0.59	0.0084	0.0110	0.77
P06899	H2B1J	H2BC11	Histone H2B type 1-J	0.0048	0.0020	2.43	0.0071	0.0074	0.97
O60814	H2B1K	HIST1H2BK	Histone H2B type 1-K	0.1212	0.2649	0.46	0.2308	0.1299	1.78
P23527	H2B1O	H2BC17	Histone H2B type 1-O	0.0012	-	+/-	-	-	-/-
Q6DN03	H2B2C	H2BC20P	Putative histone H2B type 2-C	0.0015	0.0069	0.22	0.0109	0.0053	2.06
P68431	H31	HIST1H3A	Histone H3.1	0.0245	0.0127	1.93	0.0049	0.0166	0.30
Q16695	H31T	H3-4	Histone H3.1t	-	-	-/-	0.0143	-	+/-
Q5TEC6	H3PS2	H3-2	Histone HIST2H3PS2	0.0071	0.0019	3.70	0.0397	0.0193	2.06
P62805	H4	HIST1H4A	Histone H4	0.3204	0.3415	0.94	0.2047	0.1517	1.35
Q58FF8	H90B2	HSP90AB2P	Putative heat shock protein HSP 90-beta 2	0.0006	0.0032	0.19	-	-	-/-
Q58FF7	H90B3	HSP90AB3P	Putative heat shock protein HSP 90-beta-3	0.0004	0.0080	0.04	-	-	-/-
Q58FF6	H90B4	HSP90AB4P	Putative heat shock protein HSP 90-beta 4	0.0011	-	+/-	-	-	-/-
Q14520	HABP2	HABP2	Hyaluronan-binding protein 2	0.0005	0.0005	1.00	-	-	-/-

Q9P035	HACD3	HACD3	Very-long-chain (3R)-3-hydroxyacyl-CoA dehydratase 3	0.0078	0.0036	2.19	0.0428	0.0392	1.09
Q9UJ83	HACL1	HACL1	2-hydroxyacyl-CoA lyase 1	0.0003	0.0008	0.32	0.0127	0.0131	0.97
Q9UJM8	HAOX1	HAO1	Hydroxyacid oxidase 1	0.0603	0.1213	0.50	0.0931	0.1437	0.65
Q9NYQ3	HAOX2	HAO2	Hydroxyacid oxidase 2	0.0032	0.0065	0.49	-	0.0146	-/+
Q13442	HAP28	PDAP1	28 kDa heat- and acid-stable phosphoprotein	0.0002	-	+/-	-	-	-/-
O00165	HAX1	HAX1	HCLS1-associated protein X-1	0.0084	0.0073	1.15	-	-	-/-
P69905	HBA	HBA1	Hemoglobin subunit alpha	1.2471	2.9129	0.43	1.4554	1.0321	1.41
P09105	HBAT	HBQ1	Hemoglobin subunit theta-1	0.0012	0.0027	0.42	-	-	-/-
P02008	HBAZ	HBZ	Hemoglobin subunit zeta	0.0008	0.0037	0.22	-	-	-/-
P68871	HBB	HBB	Hemoglobin subunit beta	3.6841	4.4660	0.82	2.1517	2.7857	0.77
P02042	HBD	HBD	Hemoglobin subunit delta	0.7329	0.6033	1.21	1.0124	1.0145	1.00
P69891	HBG1	HBG1	Hemoglobin subunit gamma-1	0.1429	0.0363	3.94	0.0594	0.0422	1.41
P69892	HBG2	HBG2	Hemoglobin subunit gamma-2	0.0896	0.0199	4.51	-	-	-/-
Q99714	HCD2	HSD17B10	3-hydroxyacyl-CoA dehydrogenase type-2	0.2431	0.3196	0.76	0.1814	0.3869	0.47
Q16836	HCDH	HADH	Hydroxyacyl-coenzyme A dehydrogenase, mitochondrial	0.0642	0.1363	0.47	0.0482	0.0778	0.62
P51610	HCFC1	HCFC1	Host cell factor 1	-	0.0001	-/+	0.0042	-	+/-
P08631	HCK	HCK	Tyrosine-protein kinase HCK	-	-	-/-	0.0065	-	+/-
P14317	HCLS1	HCLS1	Hematopoietic lineage cell-specific protein	0.0066	0.0020	3.31	0.0063	0.0031	2.01
P42858	HD	HTT	Huntingtin	-	-	-/-	0.0014	-	+/-

Q13547	HDAC1	HDAC1	Histone deacetylase 1	0.0027	0.0010	2.70	0.0050	0.0052	0.97
Q92769	HDAC2	HDAC2	Histone deacetylase 2	0.0004	0.0006	0.74	-	-	-/-
Q9UBN7	HDAC6	HDAC6	Histone deacetylase 6	0.0010	0.0020	0.49	0.0006	0.0120	0.05
P51858	HDGF	HDGF	Hepatoma-derived growth factor	0.0268	0.0102	2.62	0.0084	0.0028	2.97
Q08623	HDHD1	PUDP	Pseudouridine-5'-phosphatase	0.0008	0.0177	0.04	-	-	-/-
Q08623-4	HDHD1	PUDP	Isoform 4 of Pseudouridine-5'-phosphatase	-	0.0013	-/+	-	-	-/-
Q9H0R4	HDHD2	HDHD2	Haloacid dehalogenase-like hydrolase domain-containing protein 2	0.0049	0.0082	0.59	0.0064	0.0066	0.97
Q9BSH5	HDHD3	HDHD3	Haloacid dehalogenase-like hydrolase domain-containing protein 3	0.0294	0.0259	1.14	0.0186	0.0395	0.47
Q9BXW7	HDHD5	HDHD5	Haloacid dehalogenase-like hydrolase domain-containing 5	0.0064	0.0105	0.61	0.0084	0.0114	0.74
Q9H583	HEAT1	HEATR1	HEAT repeat-containing protein 1	-	-	-/-	0.0008	-	+/-
Q9NRV9	HEBP1	HEBP1	Heme-binding protein 1	0.0248	0.0471	0.53	0.0276	0.0575	0.48
Q9Y5Z4	HEBP2	HEBP2	Heme-binding protein 2	0.0271	0.0309	0.88	0.0109	0.0369	0.30
Q14CZ8	HECAM	HEPACAM	Hepatocyte cell adhesion molecule	-	-	-/-	0.0012	0.0040	0.29
Q9ULT8	HECD1	HECTD1	E3 ubiquitin-protein ligase HECTD1	-	-	-/-	0.0007	-	+/-
Q5T447	HECD3	HECTD3	E3 ubiquitin-protein ligase HECTD3	-	0.0002	-/+	0.0019	-	+/-
Q9Y4D8	HECD4	HECTD4	Probable E3 ubiquitin-protein ligase HECTD4	0.0000	-	+/-	-	-	-/-
P13716	HEM2	ALAD	Delta-aminolevulinic acid dehydratase	0.0418	0.1031	0.41	0.0221	0.1157	0.19

P08397	HEM3	HMBS	Porphobilinogen deaminase	0.0005	0.0006	0.79	-	-	-/-
P10746	HEM4	UROS	Uroporphyrinogen-III synthase	-	0.0010	-/+	-	-	-/-
P36551	HEM6	CPOX	Oxygen-dependent coproporphyrinogen-III oxidase, mitochondrial	0.0016	0.0120	0.13	0.0114	0.0269	0.42
P22830	HEMH	FECH	Ferrochelatase, mitochondrial	0.0061	0.0081	0.75	0.0106	0.0279	0.38
P02790	HEMO	HPX	Hemopexin	0.0744	0.0195	3.82	0.0551	0.0485	1.13
P05546	HEP2	SERPIND1	Heparin cofactor 2	0.0080	0.0004	20.19	0.0035	-	+/-
Q5GLZ8	HERC4	HERC4	Probable E3 ubiquitin-protein ligase HERC4	-	-	-/-	0.0019	-	+/-
P06865	HEXA	HEXA	Beta-hexosaminidase subunit alpha	0.0163	0.0109	1.50	0.0202	0.0148	1.36
P07686	HEXB	HEXB	Beta-hexosaminidase subunit beta	0.0250	0.0099	2.52	0.0313	0.0098	3.20
P04233-2	HG2A	CD74	Isoform 2 of HLA class II histocompatibility antigen gamma chain	0.0097	0.0021	4.65	0.0603	-	+/-
P04233	HG2A	CD74	HLA class II histocompatibility antigen gamma chain	0.0147	0.0011	13.06	0.0032	0.0154	0.21
B2RPK0	HGB1A	HMGB1P1	Putative high mobility group protein B1-like 1	-	-	-/-	-	0.0068	-/+
Q93099	HGD	HGD	Homogentisate 1,2-dioxygenase	0.0216	0.0542	0.40	0.0942	0.1431	0.66
P26927	HGFL	MST1	Hepatocyte growth factor-like protein	0.0003	0.0001	1.86	-	-	-/-
Q9BTY7	HGH1	HGH1	Protein HGH1 homolog	0.0020	0.0054	0.36	-	-	-/-
O14964	HGS	HGS	Hepatocyte growth factor-regulated tyrosine kinase substrate	0.0003	0.0002	1.23	0.0025	-	+/-

Q6NVY1	HIBCH	HIBCH	3-hydroxyisobutyryl-CoA hydrolase, mitochondrial	0.0316	0.0787	0.40	0.0370	0.0833	0.44
Q96JB3	HIC2	HIC2	Hypermethylated in cancer 2 protein	-	-	-/-	-	0.0034	-/+
Q9Y241	HIG1A	HIGD1A	HIG1 domain family member 1A, mitochondrial	0.0071	0.0074	0.96	-	0.0214	-/+
Q9BW72	HIG2A	HIGD2A	HIG1 domain family member 2A, mitochondrial	0.0018	0.0010	1.85	-	-	-/-
Q53FT3	HIKES	HIKESHI	Protein Hikeshi	-	0.0045	-/+	-	-	-/-
P49773	HINT1	HINT1	Histidine triad nucleotide-binding protein 1	0.0604	0.0459	1.32	0.0155	0.0798	0.19
Q9BX68	HINT2	HINT2	Histidine triad nucleotide-binding protein 2, mitochondrial	0.0220	0.0301	0.73	0.0200	0.0326	0.61
Q9NQE9	HINT3	HINT3	Histidine triad nucleotide-binding protein 3	0.0047	0.0101	0.46	-	-	-/-
O00291	HIP1	HIP1	Huntingtin-interacting protein 1	0.0005	0.0001	5.70	0.0023	-	+/-
O75146	HIP1R	HIP1R	Huntingtin-interacting protein 1-related protein	0.0001	-	+/-	0.0014	-	+/-
Q6UWX4	HIPL2	HHIPL2	HHIP-like protein 2	-	0.0001	-/+	-	-	-/-
Q2TB90	HKDC1	HKDC1	Hexokinase HKDC1	0.0043	-	+/-	0.0177	-	+/-
P13747	HLAE	HLA-E	HLA class I histocompatibility antigen, alpha chain E	-	-	-/-	0.0133	-	+/-
P30511	HLAF	HLA-F	HLA class I histocompatibility antigen, alpha chain F	0.0041	0.0005	8.09	0.0124	-	+/-
P01893	HLAH	HLA-H	Putative HLA class I histocompatibility antigen, alpha chain H	0.0006	0.0014	0.41	0.0352	-	+/-

Q8TCT9	HM13	HM13	Minor histocompatibility antigen H13	0.0053	0.0010	5.33	0.0116	0.0077	1.52
Q01581	HMCS1	HMGCS1	Hydroxymethylglutaryl-CoA synthase, cytoplasmic	0.0088	0.0081	1.08	0.0104	0.0223	0.47
P54868	HMCS2	HMGCS2	Hydroxymethylglutaryl-CoA synthase, mitochondrial	0.1507	0.3859	0.39	0.1268	0.2906	0.44
P17096	HMGA1	HMGA1	High mobility group protein HMG-I/HMG-Y	0.0011	0.0018	0.59	-	-	-/-
P09429	HMGB1	HMGB1	High mobility group protein B1	0.0149	0.0031	4.87	0.0255	0.0138	1.86
P26583	HMGB2	HMGB2	High mobility group protein B2	0.0059	-	+/-	0.0065	-	+/-
O15347	HMGB3	HMGB3	High mobility group protein B3	0.0004	-	+/-	-	-	-/-
P35914	HMGCL	HMGCL	Hydroxymethylglutaryl-CoA lyase, mitochondrial	0.0538	0.0791	0.68	0.0432	0.1028	0.42
Q92619	HMHA1	ARHGAP45	Rho GTPase-activating protein 45	-	-	-/-	0.0014	-	+/-
P09601	HMOX1	HMOX1	Heme oxygenase 1	0.0015	0.0011	1.42	0.0120	0.0161	0.75
P30519	HMOX2	HMOX2	Heme oxygenase 2	0.0075	0.0091	0.83	0.0133	-	+/-
P50135	HNMT	HNMT	Histamine N-methyltransferase	0.0195	0.0266	0.73	0.0207	0.0318	0.65
B2RXH8	HNRC2	HNRNPCL2	Heterogeneous nuclear ribonucleoprotein C-like 2	0.0092	-	+/-	-	-	-/-
O14979	HNRDL	HNRNPDL	Heterogeneous nuclear ribonucleoprotein D-like	0.0055	0.0042	1.30	0.0067	0.0033	2.01
P31943	HNRH1	HNRNPH1	Heterogeneous nuclear ribonucleoprotein H	0.0363	0.0176	2.06	0.0357	0.0326	1.09
P55795	HNRH2	HNRNPH2	Heterogeneous nuclear ribonucleoprotein H2	0.0146	0.0088	1.65	0.0538	0.0224	2.40
P31942	HNRH3	HNRNPH3	Heterogeneous nuclear ribonucleoprotein H3	0.0221	0.0110	2.00	0.0100	0.0090	1.11

Q9BUJ2	HNRL1	HNRNPUL1	Heterogeneous nuclear ribonucleoprotein U-like protein 1	0.0006	0.0002	2.94	0.0111	0.0063	1.76
Q1KMD3	HNRL2	HNRNPUL2	Heterogeneous nuclear ribonucleoprotein U-like protein 2	0.0068	0.0041	1.68	0.0123	0.0049	2.52
P07910	HNRPC	HNRNPC	Heterogeneous nuclear ribonucleoproteins C1/C2	0.0553	0.0253	2.19	0.0124	0.0045	2.78
P07910-2	HNRPC	HNRNPC	Isoform C1 of Heterogeneous nuclear ribonucleoproteins C1/C2	0.0417	0.0267	1.56	0.0270	0.0223	1.21
Q14103-2	HNRPD	HNRNPD	Isoform 2 of Heterogeneous nuclear ribonucleoprotein D0	0.0008	-	+/-	-	-	-/-
Q14103	HNRPD	HNRNPD	Heterogeneous nuclear ribonucleoprotein D0	0.0282	0.0178	1.58	0.0270	0.0241	1.12
P52597	HNRPF	HNRNPF	Heterogeneous nuclear ribonucleoprotein F	0.0508	0.0244	2.09	0.0597	0.0380	1.57
P61978-3	HNRPK	HNRNPK	Isoform 3 of Heterogeneous nuclear ribonucleoprotein K	0.0045	0.0063	0.71	0.0594	0.0345	1.72
P61978	HNRPK	HNRNPK	Heterogeneous nuclear ribonucleoprotein K	0.1150	0.0471	2.44	0.0176	0.0560	0.31
P14866	HNRPL	HNRNPL	Heterogeneous nuclear ribonucleoprotein L	0.0130	0.0060	2.17	0.0231	0.0208	1.11
P52272	HNRPM	HNRNPM	Heterogeneous nuclear ribonucleoprotein M	0.0321	0.0278	1.16	0.0467	0.0263	1.78
O60506	HNRPQ	SYNCRIP	Heterogeneous nuclear ribonucleoprotein Q	0.0136	0.0091	1.50	0.0202	0.0168	1.20
O43390	HNRPR	HNRNPR	Heterogeneous nuclear ribonucleoprotein R	0.0155	0.0106	1.47	0.0219	0.0132	1.66
Q00839-2	HNRPU	HNRNPU	Isoform 2 of Heterogeneous nuclear ribonucleoprotein U	-	0.0008	-/+	-	-	-/-
Q00839	HNRPU	HNRNPU	Heterogeneous nuclear ribonucleoprotein U	0.0364	0.0119	3.05	0.0262	0.0167	1.57

Q86XE5	HOGA1	HOGA1	4-hydroxy-2-oxoglutarate aldolase, mitochondrial	0.0025	0.0164	0.15	-	0.0185	-/+
Q9BYM8	HOIL1	RBCK1	RanBP-type and C3HC4-type zinc finger-containing protein 1	0.0005	0.0002	2.41	-	-	-/-
Q9NSB8	HOME2	HOMER2	Homer protein homolog 2	-	0.0002	-/+	-	-	-/-
Q9UJC3	HOOK1	HOOK1	Protein Hook homolog 1	-	0.0010	-/+	-	-	-/-
Q86VS8	HOOK3	HOOK3	Protein Hook homolog 3	-	0.0004	-/+	-	-	-/-
Q86YZ3	HORN	HRNR	Hornerin	0.0064	0.0085	0.75	-	-	-/-
Q8IWW8	HOT	ADHFE1	Hydroxyacid-oxoacid transhydrogenase, mitochondrial	0.0036	0.0162	0.22	0.0262	0.0493	0.53
Q5SSJ5	HP1B3	HP1BP3	Heterochromatin protein 1-binding protein 3	0.0021	0.0005	4.20	0.0056	0.0030	1.87
Q9NZL4	HPBP1	HSPBP1	Hsp70-binding protein 1	-	0.0020	-/+	-	-	-/-
P37235	HPCL1	HPCAL1	Hippocalcin-like protein 1	0.0122	0.0083	1.46	0.0121	-	+/-
Q9NWY4	HPF1	HPF1	Histone PARylation factor 1	0.0002	-	+/-	-	-	-/-
Q96S86	HPLN3	HAPLN3	Hyaluronan and proteoglycan link protein 3	-	-	-/-	0.0050	-	+/-
P32754	HPPD	HPD	4-hydroxyphenylpyruvate dioxygenase	0.0546	0.1442	0.38	0.0574	0.2265	0.25
P00492	HPRT	HPRT1	Hypoxanthine-guanine phosphoribosyltransferase	0.0088	0.0153	0.57	0.0254	0.0321	0.79
P00738	HPT	HP	Haptoglobin	0.2460	0.1251	1.97	0.0961	0.0943	1.02
P00739	HPTR	HPR	Haptoglobin-related protein	0.0192	0.0154	1.25	0.0423	-	+/-
P04196	HRG	HRG	Histidine-rich glycoprotein	0.0204	0.0131	1.55	0.0183	0.0217	0.84

Q92598	HS105	HSPH1	Heat shock protein 105 kDa	0.0019	0.0009	2.09	0.0065	0.0047	1.38
O43301	HS12A	HSPA12A	Heat shock 70 kDa protein 12A	-	-	-/-	0.0025	-	+/-
Q7LGA3	HS2ST	HS2ST1	Heparan sulfate 2-O-sulfotransferase 1	-	-	-/-	0.0038	-	+/-
P0DMV8-2	HS71A	HSPA1A	Isoform 2 of Heat shock 70 kDa protein 1A	0.0002	0.0015	0.15	-	-	-/-
P0DMV8	HS71A	HSPA1A	Heat shock 70 kDa protein 1A	0.1630	0.0498	3.27	0.0851	0.0660	1.29
P34931	HS71L	HSPA1L	Heat shock 70 kDa protein 1-like	0.0002	0.0003	0.98	-	-	-/-
O95757	HS74L	HSPA4L	Heat shock 70 kDa protein 4L	0.0009	0.0006	1.50	0.0119	0.0037	3.17
P07900	HS90A	HSP90AA1	Heat shock protein HSP 90-alpha	0.1493	0.0786	1.90	0.0970	0.0881	1.10
P08238	HS90B	HSP90AB1	Heat shock protein HSP 90-beta	0.1815	0.0757	2.40	0.1130	0.0885	1.28
Q8IWL3	HSC20	HSCB	Iron-sulfur cluster co-chaperone protein HscB	0.0012	0.0006	1.98	-	-	-/-
Q6YN16	HSDL2	HSDL2	Hydroxysteroid dehydrogenase-like protein 2	0.0384	0.0476	0.81	0.0437	0.0929	0.47
P48723	HSP13	HSPA13	Heat shock 70 kDa protein 13	0.0012	0.0005	2.51	-	-	-/-
P54652	HSP72	HSPA2	Heat shock-related 70 kDa protein 2	0.0242	0.0026	9.35	0.0219	0.0177	1.24
P34932	HSP74	HSPA4	Heat shock 70 kDa protein 4	0.0096	0.0082	1.16	0.0166	0.0201	0.82
P17066	HSP76	HSPA6	Heat shock 70 kDa protein 6	0.0001	0.0002	0.77	-	-	-/-
P11142-2	HSP7C	HSPA8	Isoform 2 of Heat shock cognate 71 kDa protein	0.0029	-	+/-	-	-	-/-
P11142	HSP7C	HSPA8	Heat shock cognate 71 kDa protein	0.3045	0.1162	2.62	0.1505	0.1076	1.40
Q0VDF9	HSP7E	HSPA14	Heat shock 70 kDa protein 14	-	0.0004	-/+	-	-	-/-
P04792	HSPB1	HSPB1	Heat shock protein beta-1	0.4583	0.1471	3.12	0.2396	0.0762	3.14

O14558	HSPB6	HSPB6	Heat shock protein beta-6	0.0420	0.0131	3.20	-	-	-/-
Q9BUP3-3	HTAI2	HTATIP2	Isoform 3 of Oxidoreductase HTATIP2	0.0050	0.0034	1.47	0.0062	-	+/-
Q9BUP3	HTAI2	HTATIP2	Oxidoreductase HTATIP2	0.0194	0.0135	1.44	0.0335	0.0060	5.60
Q92743	HTRA1	HTRA1	Serine protease HTRA1	0.0002	-	+/-	-	-	-/-
O43464	HTRA2	HTRA2	Serine protease HTRA2, mitochondrial	0.0056	0.0041	1.35	0.0084	0.0087	0.97
O43719	HTSF1	HTATSF1	HIV Tat-specific factor 1	0.0001	-	+/-	-	-	-/-
P42357	HUTH	HAL	Histidine ammonia-lyase	0.0027	0.0043	0.63	0.0027	0.0177	0.15
Q96NU7	HUTI	AMDHD1	Probable imidazolonepropionase	0.0053	0.0178	0.30	0.0065	0.0388	0.17
Q96N76	HUTU	UROC1	Urocanate hydratase	0.0043	0.0176	0.24	-	0.0859	-/+
Q7Z6Z7	HUWE1	HUWE1	E3 ubiquitin-protein ligase HUWE1	-	0.0000	-/+	0.0018	0.0012	1.56
A0A0C4DH31	HV118	IGHV1-18	Immunoglobulin heavy variable 1-18	0.0024	-	+/-	-	-	-/-
P01743	HV146	IGHV1-46	Immunoglobulin heavy variable 1-46	-	-	-/-	0.0233	-	+/-
P01780	HV307	IGHV3-7	Immunoglobulin heavy variable 3-7	0.0099	0.0041	2.39	-	-	-/-
P01782	HV309	IGHV3-9	Immunoglobulin heavy variable 3-9	0.0067	0.0026	2.59	-	-	-/-
P01766	HV313	IGHV3-13	Immunoglobulin heavy variable 3-13	-	0.0015	-/+	-	-	-/-
A0A0B4J1V0	HV315	IGHV3-15	Immunoglobulin heavy variable 3-15	0.0006	-	+/-	-	-	-/-
A0A0B4J1V1	HV321	IGHV3-21	Immunoglobulin heavy variable 3-21	0.0124	0.0012	10.22	0.0233	0.0241	0.97
P01764	HV323	IGHV3-23	Immunoglobulin heavy variable 3-23	0.0557	0.0207	2.69	0.0454	0.0241	1.88
P01768	HV330	IGHV3-30	Immunoglobulin heavy variable 3-30	0.0019	-	+/-	-	-	-/-

A0A0C4DH35	HV335	IGHV3-35	Probable non-functional immunoglobulin heavy variable 3-35	0.0038	-	+/-	-	-	-/-
A0A0A0MS15	HV349	IGHV3-49	Immunoglobulin heavy variable 3-49	0.0020	-	+/-	-	-	-/-
A0A0B4J1Y9	HV372	IGHV3-72	Immunoglobulin heavy variable 3-72	-	0.0011	-/+	-	-	-/-
A0A0C4DH34	HV428	IGHV4-28	Immunoglobulin heavy variable 4-28	0.0033	-	+/-	-	-	-/-
P01824	HV439	IGHV4-39	Immunoglobulin heavy variable 4-39	0.0084	0.0023	3.58	-	-	-/-
A0A0C4DH41	HV461	IGHV4-61	Immunoglobulin heavy variable 4-61	-	-	-/-	0.0266	0.0275	0.97
A0A0C4DH38	HV551	IGHV5-51	Immunoglobulin heavy variable 5-51	0.0086	-	+/-	0.0460	0.0476	0.97
A0A0J9YX35	HV64D	IGHV3-64D	Immunoglobulin heavy variable 3-64D	-	0.0013	-/+	-	-	-/-
A0A0B4J2H0	HV69D	IGHV1-69D	Immunoglobulin heavy variable 1-69D	0.0017	-	+/-	-	-	-/-
P19367	HXK1	HK1	Hexokinase-1	0.0037	-	+/-	0.0124	0.0042	2.96
P52789	HXK2	HK2	Hexokinase-2	0.0007	-	+/-	-	-	-/-
P52790	HXK3	HK3	Hexokinase-3	0.0014	-	+/-	0.0192	0.0097	1.98
P35557	HXK4	GCK	Hexokinase-4	0.0003	0.0025	0.12	-	0.0072	-/+
P07099	HYEP	EPHX1	Epoxide hydrolase 1	0.2878	0.1510	1.91	0.3482	0.2103	1.66
P34913	HYES	EPHX2	Bifunctional epoxide hydrolase 2	0.0117	0.0439	0.27	0.0357	0.1252	0.29
Q5T013	HYI	HYI	Putative hydroxypyruvate isomerase	-	0.0129	-/+	-	-	-/-
Q9Y4L1	HYOU1	HYOU1	Hypoxia up-regulated protein 1	0.0393	0.0207	1.90	0.0414	0.0259	1.60
Q9UF12	HYPDH	PRODH2	Hydroxyproline dehydrogenase	0.0026	0.0060	0.43	0.0053	0.0329	0.16
Q9NX55	HYPK	HYPK	Huntingtin-interacting protein K	0.0003	0.0007	0.51	-	-	-/-

Q8IU81	I2BP1	IRF2BP1	Interferon regulatory factor 2-binding protein 1	0.0002	-	+/-	-	-	-/-
Q7Z5L9	I2BP2	IRF2BP2	Interferon regulatory factor 2-binding protein 2	0.0004	-	+/-	-	-	-/-
Q2TAA2	IAH1	IAH1	Isoamyl acetate-hydrolyzing esterase 1 homolog	0.0038	0.0107	0.35	0.0147	0.0152	0.97
P08833	IBP1	IGFBP1	Insulin-like growth factor-binding protein 1	0.0004	0.0047	0.09	-	-	-/-
Q16270	IBP7	IGFBP7	Insulin-like growth factor-binding protein 7	0.0010	-	+/-	-	-	-/-
P05155	IC1	SERPING1	Plasma protease C1 inhibitor	0.0434	0.0204	2.13	0.0276	0.0214	1.29
P05362	ICAM1	ICAM1	Intercellular adhesion molecule 1	0.0078	0.0038	2.07	0.0274	0.0131	2.09
P32942	ICAM3	ICAM3	Intercellular adhesion molecule 3	0.0010	-	+/-	-	-	-/-
P54105	ICLN	CLNS1A	Methylosome subunit pICln	0.0047	0.0028	1.66	-	-	-/-
Q14197	ICT1	MRPL58	Peptidyl-tRNA hydrolase ICT1, mitochondrial	0.0006	0.0004	1.46	-	-	-/-
P14735	IDE	IDE	Insulin-degrading enzyme	0.0003	-	+/-	0.0059	0.0043	1.38
P50213	IDH3A	IDH3A	Isocitrate dehydrogenase [NAD] subunit alpha, mitochondrial	0.0251	0.0155	1.62	0.0182	0.0211	0.86
O43837	IDH3B	IDH3B	Isocitrate dehydrogenase [NAD] subunit beta, mitochondrial	0.0056	0.0034	1.66	0.0066	0.0044	1.49
P51553	IDH3G	IDH3G	Isocitrate dehydrogenase [NAD] subunit gamma, mitochondrial	0.0024	0.0011	2.24	0.0073	0.0049	1.49
O75874	IDHC	IDH1	Isocitrate dehydrogenase [NADP] cytoplasmic	0.1166	0.1340	0.87	0.1526	0.1880	0.81

P48735	IDHP	IDH2	Isocitrate dehydrogenase [NADP], mitochondrial	0.1008	0.1032	0.98	0.1149	0.1431	0.80
Q13907	IDI1	IDI1	Isopentenyl-diphosphate Delta-isomerase 1	0.0040	0.0072	0.56	0.0107	0.0201	0.53
P22304	IDS	IDS	Iduronate 2-sulfatase	0.0160	0.0887	0.18	-	-	-/-
P35475	IDUA	IDUA	Alpha-L-iduronidase	0.0001	-	+/-	0.0035	-	+/-
Q16666	IF16	IFI16	Gamma-interferon-inducible protein 16	0.0016	-	+/-	0.0033	-	+/-
P47813	IF1AX	EIF1AX	Eukaryotic translation initiation factor 1A, X-chromosomal	0.0013	-	+/-	-	-	-/-
O14602	IF1AY	EIF1AY	Eukaryotic translation initiation factor 1A, Y-chromosomal	0.0049	0.0082	0.59	-	0.0077	-/+
P05198	IF2A	EIF2S1	Eukaryotic translation initiation factor 2 subunit 1	0.0271	0.0233	1.16	0.0259	0.0117	2.21
P20042	IF2B	EIF2S2	Eukaryotic translation initiation factor 2 subunit 2	0.0029	0.0020	1.47	0.0101	0.0049	2.05
Q9Y6M1	IF2B2	IGF2BP2	Insulin-like growth factor 2 mRNA-binding protein 2	0.0003	-	+/-	-	-	-/-
P41091	IF2G	EIF2S3	Eukaryotic translation initiation factor 2 subunit 3	0.0122	0.0077	1.58	0.0266	0.0151	1.76
P46199	IF2M	MTIF2	Translation initiation factor IF-2, mitochondrial	0.0002	-	+/-	0.0018	-	+/-
O60841	IF2P	EIF5B	Eukaryotic translation initiation factor 5B	0.0000	-	+/-	0.0020	0.0013	1.63
Q9H2K0	IF3M	MTIF3	Translation initiation factor IF-3, mitochondrial	0.0005	0.0002	2.07	-	-	-/-

P60842-2	IF4A1	EIF4A1	Isoform 2 of Eukaryotic initiation factor 4A-I	0.0156	-	+/-	-	-	-/-
P60842	IF4A1	EIF4A1	Eukaryotic initiation factor 4A-I	0.0884	0.0476	1.85	0.0774	0.0449	1.72
Q14240	IF4A2	EIF4A2	Eukaryotic initiation factor 4A-II	0.0288	0.0245	1.18	0.0270	0.0247	1.09
P38919	IF4A3	EIF4A3	Eukaryotic initiation factor 4A-III	0.0118	0.0043	2.78	0.0190	0.0096	1.98
P23588	IF4B	EIF4B	Eukaryotic translation initiation factor 4B	0.0005	-	+/-	-	-	-/-
P06730	IF4E	EIF4E	Eukaryotic translation initiation factor 4E	0.0024	0.0041	0.58	0.0069	0.0071	0.97
Q04637	IF4G1	EIF4G1	Eukaryotic translation initiation factor 4 gamma 1	0.0010	0.0008	1.28	0.0068	0.0068	0.99
P78344	IF4G2	EIF4G2	Eukaryotic translation initiation factor 4 gamma 2	-	-	-/-	0.0034	-	+/-
Q15056	IF4H	EIF4H	Eukaryotic translation initiation factor 4H	0.0090	0.0121	0.74	0.0211	0.0082	2.56
P55010	IF5	EIF5	Eukaryotic translation initiation factor 5	0.0016	0.0029	0.55	0.0090	0.0060	1.50
P63241	IF5A1	EIF5A	Eukaryotic translation initiation factor 5A-1	0.2170	0.2893	0.75	0.2457	0.2568	0.96
Q9GZV4	IF5A2	EIF5A2	Eukaryotic translation initiation factor 5A-2	0.0012	-	+/-	-	-	-/-
P56537	IF6	EIF6	Eukaryotic translation initiation factor 6	0.0354	0.0408	0.87	0.0386	0.0835	0.46
P09914	IFIT1	IFIT1	Interferon-induced protein with tetratricopeptide repeats 1	0.0009	-	+/-	0.0028	-	+/-
P09913	IFIT2	IFIT2	Interferon-induced protein with tetratricopeptide repeats 2	-	0.0002	-/+	-	-	-/-
O14879	IFIT3	IFIT3	Interferon-induced protein with tetratricopeptide repeats 3	0.0008	0.0023	0.34	-	-	-/-
P13164	IFM1	IFITM1	Interferon-induced transmembrane protein 1	-	-	-/-	0.0233	-	+/-

Q9Y547	IFT25	HSPB11	Intraflagellar transport protein 25 homolog	0.0082	0.0125	0.65	-	-	-/-
Q9BW83	IFT27	IFT27	Intraflagellar transport protein 27 homolog	0.0029	0.0068	0.43	0.0098	-	+/-
P0DOX2	IGA2		Immunoglobulin alpha-2 heavy chain	0.0045	0.0024	1.82	-	0.0161	-/+
P0DOX3	IGD		Immunoglobulin delta heavy chain	0.0003	-	+/-	-	-	-/-
P0DOX5	IGG1		Immunoglobulin gamma-1 heavy chain	0.0098	0.0014	6.80	0.1556	0.1303	1.19
P01876	IGHA1	IGHA1	Immunoglobulin heavy constant alpha 1	0.2083	0.1232	1.69	0.1267	0.1000	1.27
P01877	IGHA2	IGHA2	Immunoglobulin heavy constant alpha 2	0.0098	0.0084	1.16	0.0088	0.0091	0.97
P01880	IGHD	IGHD	Immunoglobulin heavy constant delta	0.0003	0.0003	0.99	-	0.0052	-/+
P01857	IGHG1	IGHG1	Immunoglobulin heavy constant gamma 1	0.3831	0.3677	1.04	0.3064	0.3147	0.97
P01859	IGHG2	IGHG2	Immunoglobulin heavy constant gamma 2	0.2250	0.1763	1.28	0.2944	0.2875	1.02
P01860	IGHG3	IGHG3	Immunoglobulin heavy constant gamma 3	0.0838	0.0887	0.94	0.1441	0.1186	1.21
P01861	IGHG4	IGHG4	Immunoglobulin heavy constant gamma 4	0.0799	0.0573	1.39	0.1918	0.1538	1.25
P01871	IGHM	IGHM	Immunoglobulin heavy constant mu	0.0980	0.0423	2.31	0.0534	0.0466	1.15
P01591	IGJ	JCHAIN	Immunoglobulin J chain	0.0096	0.0063	1.51	0.0190	-	+/-
P0DOX7	IGK		Immunoglobulin kappa light chain	0.0380	0.0352	1.08	0.0983	0.1144	0.86
P01834	IGKC	IGKC	Immunoglobulin kappa constant	0.6928	0.5633	1.23	0.2130	0.2709	0.79
P0DOX8	IGL1		Immunoglobulin lambda-1 light chain	0.0548	0.0183	3.00	-	0.0321	-/+
P0CG04	IGLC1	IGLC1	Immunoglobulin lambda constant 1	0.0622	0.0572	1.09	0.1172	0.1013	1.16
P0DOY2	IGLC2	IGLC2	Immunoglobulin lambda constant 2	0.1741	0.0881	1.98	0.1400	0.1122	1.25
P0CF74	IGLC6	IGLC6	Immunoglobulin lambda constant 6	0.0356	0.0246	1.45	-	-	-/-

A0M8Q6	IGLC7	IGLC7	Immunoglobulin lambda constant 7	0.0568	0.0470	1.21	0.0317	0.0454	0.70
P0DOX6	IGM		Immunoglobulin mu heavy chain	0.0033	-	+/-	-	-	-/-
Q8N9C0	IGS22	IGSF22	Immunoglobulin superfamily member 22	-	0.0001	-/+	-	-	-/-
Q969P0	IGSF8	IGSF8	Immunoglobulin superfamily member 8	0.0005	-	+/-	-	0.0037	-/+
P25963	IKBA	NFKBIA	NF-kappa-B inhibitor alpha	0.0008	0.0022	0.35	-	-	-/-
Q15653	IKBB	NFKBIB	NF-kappa-B inhibitor beta	0.0005	0.0014	0.38	-	-	-/-
Q70UQ0	IKIP	IKBIP	Inhibitor of nuclear factor kappa-B kinase-interacting protein	0.0017	-	+/-	-	-	-/-
Q70UQ0-4	IKIP	IKBIP	Isoform 4 of Inhibitor of nuclear factor kappa-B kinase-interacting protein	0.0025	0.0008	3.33	-	-	-/-
Q14005	IL16	IL16	Pro-interleukin-16	0.0003	0.0003	0.88	-	-	-/-
Q14116	IL18	IL18	Interleukin-18	0.0024	0.0016	1.53	-	-	-/-
Q9NPH3	IL1AP	IL1RAP	Interleukin-1 receptor accessory protein	0.0005	-	+/-	-	-	-/-
P18510	IL1RA	IL1RN	Interleukin-1 receptor antagonist protein	0.0025	0.0034	0.72	-	-	-/-
P24001	IL32	IL32	Interleukin-32	-	-	-/-	0.0100	-	+/-
O95760	IL33	IL33	Interleukin-33	-	0.0007	-/+	-	-	-/-
P30740	ILEU	SERPINB1	Leukocyte elastase inhibitor	0.1025	0.0230	4.46	0.1529	0.0292	5.23
Q12905	ILF2	ILF2	Interleukin enhancer-binding factor 2	0.0180	0.0125	1.44	0.0396	0.0229	1.73
Q12906	ILF3	ILF3	Interleukin enhancer-binding factor 3	0.0122	0.0040	3.08	0.0175	0.0153	1.14
Q13418	ILK	ILK	Integrin-linked protein kinase	0.0050	0.0076	0.66	0.0366	0.0265	1.38

Q9H0C8	ILKAP	ILKAP	Integrin-linked kinase-associated serine/threonine phosphatase 2C	0.0003	0.0003	0.80	-	-	-/-
A1L0T0	ILVBL	ILVBL	Acetolactate synthase-like protein	0.0018	0.0028	0.65	0.0270	0.0367	0.74
P52292	IMA1	KPNA2	Importin subunit alpha-1	0.0001	-	+/-	-	-	-/-
O00629	IMA3	KPNA4	Importin subunit alpha-3	0.0002	0.0009	0.19	0.0129	0.0076	1.71
O00505	IMA4	KPNA3	Importin subunit alpha-4	-	0.0007	-/+	0.0069	0.0047	1.49
P52294	IMA5	KPNA1	Importin subunit alpha-5	0.0012	0.0012	0.99	0.0076	0.0037	2.02
O15131	IMA6	KPNA5	Importin subunit alpha-6	0.0006	0.0004	1.37	-	-	-/-
O60684	IMA7	KPNA6	Importin subunit alpha-7	0.0011	0.0015	0.74	0.0074	-	+/-
Q14974	IMB1	KPNB1	Importin subunit beta-1	0.0077	0.0047	1.62	0.0292	0.0223	1.31
P20839	IMDH1	IMPDH1	Inosine-5'-monophosphate dehydrogenase 1	0.0004	-	+/-	-	-	-/-
P12268	IMDH2	IMPDH2	Inosine-5'-monophosphate dehydrogenase 2	0.0080	0.0012	6.63	0.0263	0.0109	2.42
Q9NV31	IMP3	IMP3	U3 small nucleolar ribonucleoprotein protein IMP3	0.0004	0.0005	0.92	-	-	-/-
P29218	IMPA1	IMPA1	Inositol monophosphatase 1	0.0111	0.0077	1.43	0.0310	-	+/-
Q9NX62	IMPA3	IMPAD1	Inositol monophosphatase 3	0.0012	-	+/-	-	-	-/-
Q9P2X3	IMPCT	IMPACT	Protein IMPACT	-	-	-/-	0.0049	-	+/-
P80217	IN35	IFI35	Interferon-induced 35 kDa protein	0.0033	0.0028	1.18	0.0103	0.0068	1.51
P17181	INAR1	IFNAR1	Interferon alpha/beta receptor 1	0.0001	-	+/-	-	-	-/-
Q27J81	INF2	INF2	Inverted formin-2	-	-	-/-	0.0054	0.0039	1.39
P55103	INHBC	INHBC	Inhibin beta C chain	-	0.0010	-/+	-	-	-/-

P58166	INHBE	INHBE	Inhibin beta E chain	0.0038	0.0032	1.17	0.0150	0.0135	1.11
Q9NPH2	INO1	ISYNA1	Inositol-3-phosphate synthase 1	0.0011	0.0020	0.54	0.0086	0.0114	0.75
Q96PE3	INP4A	INPP4A	Inositol polyphosphate-4-phosphatase type I A	-	-	-/-	0.0017	-	+/-
Q9BT40	INP5K	INPP5K	Inositol polyphosphate 5-phosphatase K	0.0004	-	+/-	-	-	-/-
P49441	INPP	INPP1	Inositol polyphosphate 1-phosphatase	0.0007	0.0018	0.38	-	0.0049	-/+
P06213	INSR	INSR	Insulin receptor	0.0001	-	+/-	0.0020	-	+/-
Q8N201	INT1	INTS1	Integrator complex subunit 1	0.0000	-	+/-	-	-	-/-
Q9UL03	INT6	INTS6	Integrator complex subunit 6	0.0002	-	+/-	-	-	-/-
Q8TEX9	IPO4	IPO4	Importin-4	-	-	-/-	0.0021	0.0022	0.97
O00410	IPO5	IPO5	Importin-5	0.0007	0.0002	3.19	0.0145	0.0095	1.53
O95373	IPO7	IPO7	Importin-7	-	-	-/-	0.0069	0.0034	2.01
Q96P70	IPO9	IPO9	Importin-9	0.0001	0.0001	0.67	0.0052	-	+/-
P05154	IPSP	SERPINA5	Plasma serine protease inhibitor	0.0070	-	+/-	0.0147	0.0070	2.10
Q15181	IPYR	PPA1	Inorganic pyrophosphatase	0.0580	0.0309	1.88	0.0711	0.0441	1.61
Q9H2U2	IPYR2	PPA2	Inorganic pyrophosphatase 2, mitochondrial	0.0152	0.0262	0.58	0.0383	0.0467	0.82
P46940	IQGA1	IQGAP1	Ras GTPase-activating-like protein IQGAP1	0.0128	0.0071	1.81	0.0283	0.0180	1.57
Q13576	IQGA2	IQGAP2	Ras GTPase-activating-like protein IQGAP2	0.0294	0.0234	1.25	0.0321	0.0416	0.77
Q9Y5U9	IR3IP	IER3IP1	Immediate early response 3-interacting protein 1	-	-	-/-	0.0371	0.0384	0.97
Q14653	IRF3	IRF3	Interferon regulatory factor 3	-	0.0012	-/+	-	-	-/-

Q8WZA9	IRGQ	IRGQ	Immunity-related GTPase family Q protein	0.0019	0.0052	0.37	0.0043	-	+/-
Q9BUE6	ISCA1	ISCA1	Iron-sulfur cluster assembly 1 homolog, mitochondrial	0.0003	0.0020	0.15	-	-	-/-
Q86U28	ISCA2	ISCA2	Iron-sulfur cluster assembly 2 homolog, mitochondrial	0.0122	0.0140	0.87	-	-	-/-
Q9H1K1	ISCU	ISCU	Iron-sulfur cluster assembly enzyme ISCU, mitochondrial	0.0002	0.0008	0.23	-	-	-/-
P05161	ISG15	ISG15	Ubiquitin-like protein ISG15	0.0043	-	+/-	-	-	-/-
Q96AZ6	ISG20	ISG20	Interferon-stimulated gene 20 kDa protein	0.0012	0.0012	1.02	-	-	-/-
O14498	ISLR	ISLR	Immunoglobulin superfamily containing leucine-rich repeat protein	0.0007	-	+/-	-	-	-/-
Q96CN7	ISOC1	ISOC1	Isochorismatase domain-containing protein 1	0.0274	0.0415	0.66	0.0212	0.0318	0.67
Q96AB3	ISOC2	ISOC2	Isochorismatase domain-containing protein 2	0.0278	0.0826	0.34	0.0546	0.1391	0.39
Q9ULR0	ISY1	ISY1	Pre-mRNA-splicing factor ISY1 homolog	-	0.0003	-/+	-	-	-/-
P56199	ITA1	ITGA1	Integrin alpha-1	0.0083	0.0042	1.98	0.0090	0.0073	1.23
P17301	ITA2	ITGA2	Integrin alpha-2	0.0011	-	+/-	-	-	-/-
P08514	ITA2B	ITGA2B	Integrin alpha-IIb	0.0064	0.0023	2.76	-	-	-/-
P13612	ITA4	ITGA4	Integrin alpha-4	0.0008	-	+/-	-	-	-/-
P08648	ITA5	ITGA5	Integrin alpha-5	0.0021	0.0018	1.21	-	-	-/-
P23229	ITA6	ITGA6	Integrin alpha-6	0.0032	0.0004	8.37	0.0015	-	+/-
Q13683-13	ITA7	ITGA7	Isoform 2 of Integrin alpha-7	0.0003	-	+/-	-	-	-/-

Q13797	ITA9	ITGA9	Integrin alpha-9	-	0.0001	-/+	-	-	-/-
P20701	ITAL	ITGAL	Integrin alpha-L	0.0009	-	+/-	0.0020	-	+/-
P11215	ITAM	ITGAM	Integrin alpha-M	0.0123	-	+/-	0.0266	0.0018	14.55
P06756	ITAV	ITGAV	Integrin alpha-V	0.0097	0.0025	3.86	0.0048	-	+/-
P20702	ITAX	ITGAX	Integrin alpha-X	0.0002	-	+/-	0.0052	-	+/-
P05556	ITB1	ITGB1	Integrin beta-1	0.0252	0.0120	2.09	0.0270	0.0215	1.26
P05107	ITB2	ITGB2	Integrin beta-2	0.0083	0.0006	14.89	0.0177	0.0035	5.02
P05106	ITB3	ITGB3	Integrin beta-3	0.0004	-	+/-	-	-	-/-
P18084	ITB5	ITGB5	Integrin beta-5	0.0011	-	+/-	0.0038	-	+/-
P18564	ITB6	ITGB6	Integrin beta-6	-	0.0002	-/+	-	-	-/-
P19827	ITIH1	ITIH1	Inter-alpha-trypsin inhibitor heavy chain H1	0.0138	0.0051	2.68	0.0150	0.0109	1.37
P19823	ITIH2	ITIH2	Inter-alpha-trypsin inhibitor heavy chain H2	0.0228	0.0036	6.32	0.0202	0.0073	2.78
Q06033	ITIH3	ITIH3	Inter-alpha-trypsin inhibitor heavy chain H3	0.0034	0.0004	8.05	0.0054	0.0033	1.65
Q14624-2	ITIH4	ITIH4	Isoform 2 of Inter-alpha-trypsin inhibitor heavy chain H4	0.0004	0.0015	0.26	-	-	-/-
Q14624	ITIH4	ITIH4	Inter-alpha-trypsin inhibitor heavy chain H4	0.0261	0.0080	3.28	0.0182	0.0172	1.05
Q9Y287	ITM2B	ITM2B	Integral membrane protein 2B	0.0010	-	+/-	-	-	-/-
Q9BY32	ITPA	ITPA	Inosine triphosphate pyrophosphatase	0.0032	0.0048	0.67	0.0098	-	+/-
P28290	ITPI2	ITPRID2	Protein ITPRID2	0.0004	0.0003	1.26	-	0.0015	-/+
Q14643	ITPR1	ITPR1	Inositol 1,4,5-trisphosphate receptor type 1	-	-	-/-	0.0018	-	+/-

Q14571	ITPR2	ITPR2	Inositol 1,4,5-trisphosphate receptor type 2	0.0001	0.0000	1.80	0.0038	0.0040	0.97
Q15811	ITSN1	ITSN1	Intersectin-1	0.0001	-	+/-	-	-	-/-
P26440	IVD	IVD	Isovaleryl-CoA dehydrogenase, mitochondrial	0.0218	0.0431	0.50	0.0447	0.0855	0.52
Q6PHW0	IYD1	IYD	Iodotyrosine deiodinase 1	-	-	-/-	-	0.0080	-/+
Q9Y624	JAM1	F11R	Junctional adhesion molecule A	0.0055	0.0017	3.16	0.0103	0.0107	0.97
Q9UPT6	JIP3	MAPK8IP3	C-Jun-amino-terminal kinase-interacting protein 3	0.0000	-	+/-	-	-	-/-
O60271	JIP4	SPAG9	C-Jun-amino-terminal kinase-interacting protein 4	0.0000	-	+/-	-	-	-/-
Q9H910	JUPI2	JPT2	Jupiter microtubule associated homolog 2	-	0.0005	-/+	-	-	-/-
Q9ULL0	K1210	KIAA1210	Acrosomal protein KIAA1210	-	-	-/-	0.0009	-	+/-
P13645	K1C10	KRT10	Keratin, type I cytoskeletal 10	2.8289	2.5023	1.13	0.1317	0.0680	1.94
Q99456	K1C12	KRT12	Keratin, type I cytoskeletal 12	0.0001	0.0006	0.17	-	-	-/-
P13646	K1C13	KRT13	Keratin, type I cytoskeletal 13	0.0253	0.1554	0.16	-	-	-/-
P02533	K1C14	KRT14	Keratin, type I cytoskeletal 14	0.4036	0.6963	0.58	0.0093	0.0075	1.23
P19012	K1C15	KRT15	Keratin, type I cytoskeletal 15	0.0007	0.0014	0.49	-	-	-/-
P08779	K1C16	KRT16	Keratin, type I cytoskeletal 16	0.3418	0.6810	0.50	-	-	-/-
Q04695	K1C17	KRT17	Keratin, type I cytoskeletal 17	0.0654	0.2225	0.29	-	-	-/-
P05783	K1C18	KRT18	Keratin, type I cytoskeletal 18	0.3719	0.2361	1.58	0.1885	0.1688	1.12
P08727	K1C19	KRT19	Keratin, type I cytoskeletal 19	0.0505	0.0543	0.93	0.0140	0.0162	0.86
Q2M2I5	K1C24	KRT24	Keratin, type I cytoskeletal 24	0.0071	0.0004	17.33	-	-	-/-

Q7Z3Y8	K1C27	KRT27	Keratin, type I cytoskeletal 27	0.0088	0.0040	2.22	0.0136	-	+/-
P35527	K1C9	KRT9	Keratin, type I cytoskeletal 9	1.6294	1.8249	0.89	0.0538	0.0193	2.79
Q15323	K1H1	KRT31	Keratin, type I cuticular Ha1	0.0050	0.0349	0.14	-	-	-/-
Q14532	K1H2	KRT32	Keratin, type I cuticular Ha2	-	0.0003	-/+	-	-	-/-
Q8IYS2	K2013	KIAA2013	Uncharacterized protein KIAA2013	-	-	-/-	0.0035	-	+/-
P35908	K22E	KRT2	Keratin, type II cytoskeletal 2 epidermal	1.3021	1.4212	0.92	0.0427	0.0298	1.43
Q01546	K22O	KRT76	Keratin, type II cytoskeletal 2 oral	-	0.0034	-/+	-	-	-/-
P04264	K2C1	KRT1	Keratin, type II cytoskeletal 1	2.6501	2.4565	1.08	0.0985	0.0636	1.55
Q7Z794	K2C1B	KRT77	Keratin, type II cytoskeletal 1b	0.0042	0.0142	0.30	-	-	-/-
P12035	K2C3	KRT3	Keratin, type II cytoskeletal 3	0.0069	0.0316	0.22	-	-	-/-
P19013	K2C4	KRT4	Keratin, type II cytoskeletal 4	0.0010	0.0651	0.02	-	-	-/-
P13647	K2C5	KRT5	Keratin, type II cytoskeletal 5	0.3911	0.5506	0.71	0.0059	0.0045	1.31
P02538	K2C6A	KRT6A	Keratin, type II cytoskeletal 6A	0.3238	0.5387	0.60	0.0091	-	+/-
P04259	K2C6B	KRT6B	Keratin, type II cytoskeletal 6B	0.1255	0.3831	0.33	-	-	-/-
P48668	K2C6C	KRT6C	Keratin, type II cytoskeletal 6C	-	0.0333	-/+	-	-	-/-
P08729	K2C7	KRT7	Keratin, type II cytoskeletal 7	0.0710	0.0435	1.63	0.0239	0.0160	1.50
Q3SY84	K2C71	KRT71	Keratin, type II cytoskeletal 71	-	0.0007	-/+	-	-	-/-
Q14CN4	K2C72	KRT72	Keratin, type II cytoskeletal 72	0.0069	0.0216	0.32	-	-	-/-
Q14CN4-2	K2C72	KRT72	Isoform 2 of Keratin, type II cytoskeletal 72	-	0.0003	-/+	-	-	-/-
Q86Y46	K2C73	KRT73	Keratin, type II cytoskeletal 73	0.0007	0.0012	0.58	-	-	-/-

Q7RTS7	K2C74	KRT74	Keratin, type II cytoskeletal 74	0.0003	0.0026	0.10	-	-	-/-
O95678	K2C75	KRT75	Keratin, type II cytoskeletal 75	0.0346	0.0216	1.60	0.0043	-	+/-
Q8N1N4	K2C78	KRT78	Keratin, type II cytoskeletal 78	0.0011	0.0350	0.03	-	-	-/-
Q5XKE5	K2C79	KRT79	Keratin, type II cytoskeletal 79	0.0035	0.0048	0.73	-	-	-/-
P05787	K2C8	KRT8	Keratin, type II cytoskeletal 8	0.6346	0.4554	1.39	0.2142	0.1569	1.37
Q6KB66	K2C80	KRT80	Keratin, type II cytoskeletal 80	0.0007	0.0047	0.15	-	-	-/-
P00568	KAD1	AK1	Adenylate kinase isoenzyme 1	0.0183	0.0213	0.86	0.0272	0.0273	1.00
P54819-2	KAD2	AK2	Isoform 2 of Adenylate kinase 2, mitochondrial	0.0080	-	+/-	-	0.0044	-/+
P54819	KAD2	AK2	Adenylate kinase 2, mitochondrial	0.0694	0.1516	0.46	0.0554	0.0781	0.71
Q9UIJ7-2	KAD3	AK3	Isoform 2 of GTP:AMP phosphotransferase AK3, mitochondrial	0.0009	-	+/-	-	-	-/-
Q9UIJ7	KAD3	AK3	GTP:AMP phosphotransferase AK3, mitochondrial	0.0463	0.0935	0.49	0.0510	0.1008	0.51
P27144	KAD4	AK4	Adenylate kinase 4, mitochondrial	0.0416	0.0609	0.68	0.0211	0.0674	0.31
Q5TCS8	KAD9	AK9	Adenylate kinase 9	0.0000	-	+/-	-	-	-/-
P29622	KAIN	SERPINA4	Kallistatin	0.0021	0.0002	8.90	0.0155	0.0161	0.97
Q14678	KANK1	KANK1	KN motif and ankyrin repeat domain-containing protein 1	0.0002	-	+/-	-	-	-/-
Q63ZY3	KANK2	KANK2	KN motif and ankyrin repeat domain-containing protein 2	0.0012	0.0024	0.52	0.0046	0.0035	1.31
Q7Z3B3	KANL1	KANSL1	KAT8 regulatory NSL complex subunit 1	-	0.0001	-/+	-	-	-/-

P10644-2	KAP0	PRKAR1A	Isoform 2 of cAMP-dependent protein kinase type I-alpha regulatory subunit	-	0.0003	-/+	-	-	-/-
P10644	KAP0	PRKAR1A	cAMP-dependent protein kinase type I-alpha regulatory subunit	0.0283	0.0165	1.71	0.0214	0.0083	2.56
P31321	KAP1	PRKAR1B	cAMP-dependent protein kinase type I-beta regulatory subunit	-	0.0006	-/+	-	-	-/-
P13861	KAP2	PRKAR2A	cAMP-dependent protein kinase type II-alpha regulatory subunit	0.0393	0.0243	1.62	0.0546	0.0390	1.40
P31323	KAP3	PRKAR2B	cAMP-dependent protein kinase type II-beta regulatory subunit	0.0010	0.0041	0.24	-	-	-/-
P17612	KAPCA	PRKACA	cAMP-dependent protein kinase catalytic subunit alpha	0.0036	0.0032	1.14	0.0263	0.0272	0.97
P22694	KAPCB	PRKACB	cAMP-dependent protein kinase catalytic subunit beta	0.0004	-	+/-	0.0079	0.0060	1.32
Q16773	KAT1	KYAT1	Kynurenine--oxoglutarate transaminase 1	-	0.0010	-/+	-	-	-/-
Q6YP21	KAT3	KYAT3	Kynurenine--oxoglutarate transaminase 3	0.0001	0.0009	0.13	0.0054	0.0056	0.97
Q8IYT4	KATL2	KATNAL2	Katanin p60 ATPase-containing subunit A-like 2	0.0001	-	+/-	-	-	-/-
O75600	KBL	GCAT	2-amino-3-ketobutyrate coenzyme A ligase, mitochondrial	0.0005	0.0054	0.09	0.0043	0.0178	0.24
P48729	KC1A	CSNK1A1	Casein kinase I isoform alpha	0.0011	0.0019	0.60	0.0101	0.0039	2.59
P48730	KC1D	CSNK1D	Casein kinase I isoform delta	0.0002	0.0002	1.02	0.0031	-	+/-

Q13303	KCAB2	KCNAB2	Voltage-gated potassium channel subunit beta-2	0.0004	-	+/-	-	-	-/-
Q14012	KCC1A	CAMK1	Calcium/calmodulin-dependent protein kinase type 1	0.0005	0.0023	0.21	-	-	-/-
Q8IU85	KCC1D	CAMK1D	Calcium/calmodulin-dependent protein kinase type 1D	-	0.0002	-/+	-	-	-/-
Q13554	KCC2B	CAMK2B	Calcium/calmodulin-dependent protein kinase type II subunit beta	-	-	-/-	-	0.0045	-/+
Q13557	KCC2D	CAMK2D	Calcium/calmodulin-dependent protein kinase type II subunit delta	0.0004	0.0005	0.80	0.0130	0.0092	1.41
Q13555	KCC2G	CAMK2G	Calcium/calmodulin-dependent protein kinase type II subunit gamma	-	-	-/-	0.0077	0.0046	1.67
Q96CX2	KCD12	KCTD12	BTB/POZ domain-containing protein KCTD12	0.0207	0.0126	1.65	0.0257	0.0151	1.71
Q4G0X4	KCD21	KCTD21	BTB/POZ domain-containing protein KCTD21	0.0008	0.0037	0.23	-	-	-/-
P12277	KCRB	CKB	Creatine kinase B-type	0.0025	0.0061	0.41	0.0075	0.0078	0.97
P06732	KCRM	CKM	Creatine kinase M-type	0.0015	-	+/-	-	-	-/-
P30085	KCY	CMPK1	UMP-CMP kinase	0.0723	0.0824	0.88	0.0266	0.0416	0.64
O60341	KDM1A	KDM1A	Lysine-specific histone demethylase 1A	0.0001	-	+/-	-	-	-/-
Q06136	KDSR	KDSR	3-ketodihydrosphingosine reductase	-	-	-/-	0.0107	0.0111	0.97
Q14145	KEAP1	KEAP1	Kelch-like ECH-associated protein 1	0.0035	-	+/-	0.0093	-	+/-
Q63HM1	KFA	AFMID	Kynurenine formamidase	0.0055	0.0101	0.54	-	0.0168	-/+

P82909	KGD4	MRPS36	Alpha-ketoglutarate dehydrogenase component 4	-	0.0020	-/+	-	-	-/-
Q13976	KGP1	PRKG1	cGMP-dependent protein kinase 1	-	-	-/-	0.0031	-	+/-
Q16774	KGUA	GUK1	Guanylate kinase	0.0206	0.0087	2.37	-	-	-/-
Q16774-2	KGUA	GUK1	Isoform 2 of Guanylate kinase	0.0013	0.0036	0.37	-	-	-/-
Q07666	KHDR1	KHDRBS1	KH domain-containing, RNA-binding, signal transduction-associated protein 1	0.0033	0.0010	3.28	0.0070	-	+/-
O75525	KHDR3	KHDRBS3	KH domain-containing, RNA-binding, signal transduction-associated protein 3	0.0007	-	+/-	-	-	-/-
P50053-2	KHK	KHK	Isoform A of Ketohexokinase	0.0025	0.0043	0.58	0.0128	0.0476	0.27
P50053	KHK	KHK	Ketohexokinase	0.0234	0.0438	0.54	0.0166	0.0892	0.19
Q9NQT8	KI13B	KIF13B	Kinesin-like protein KIF13B	0.0000	-	+/-	0.0017	0.0018	0.97
Q96L93	KI16B	KIF16B	Kinesin-like protein KIF16B	0.0001	-	+/-	-	-	-/-
Q12756	KIF1A	KIF1A	Kinesin-like protein KIF1A	-	-	-/-	0.0002	-	+/-
O43896	KIF1C	KIF1C	Kinesin-like protein KIF1C	-	-	-/-	0.0013	-	+/-
O60282	KIF5C	KIF5C	Kinesin heavy chain isoform 5C	0.0000	-	+/-	-	-	-/-
Q03426	KIME	MVK	Mevalonate kinase	0.0008	0.0027	0.29	-	0.0245	-/+
P33176	KINH	KIF5B	Kinesin-1 heavy chain	0.0012	0.0003	3.49	0.0053	-	+/-
Q8TBQ9	KISHA	TMEM167A	Protein kish-A	-	-	-/-	0.0310	-	+/-
O00142	KITM	TK2	Thymidine kinase 2, mitochondrial	-	0.0023	-/+	0.0051	0.0053	0.97
Q2M2Z5	KIZ	KIZ	Centrosomal protein kizuna	-	0.0001	-/+	-	-	-/-

Q07866	KLC1	KLC1	Kinesin light chain 1	-	0.0001	-/+	-	-	-/-
Q9NSK0	KLC4	KLC4	Kinesin light chain 4	0.0011	0.0010	1.13	-	-	-/-
P03952	KLKB1	KLKB1	Plasma kallikrein	0.0020	0.0002	12.38	0.0029	0.0030	0.97
O15229	KMO	KMO	Kynurenine 3-monooxygenase	0.0021	0.0051	0.42	0.0249	0.0598	0.42
Q9UMN6	KMT2B	KMT2B	Histone-lysine N-methyltransferase 2B	-	0.0000	-/+	-	-	-/-
Q8NEZ4	KMT2C	KMT2C	Histone-lysine N-methyltransferase 2C	-	-	-/-	0.0002	-	+/-
Q4FZB7	KMT5B	KMT5B	Histone-lysine N-methyltransferase KMT5B	-	-	-/-	0.0003	-	+/-
P01042-2	KNG1	KNG1	Isoform LMW of Kininogen-1	0.0151	0.0095	1.58	0.0106	-	+/-
P01042	KNG1	KNG1	Kininogen-1	0.0235	0.0097	2.42	0.0111	0.0058	1.91
P50748	KNTC1	KNTC1	Kinetochore-associated protein 1	0.0001	-	+/-	-	-	-/-
P46019	KPB2	PHKA2	Phosphorylase b kinase regulatory subunit alpha, liver isoform	-	0.0002	-/+	-	0.0097	-/+
Q93100	KPBB	PHKB	Phosphorylase b kinase regulatory subunit beta	0.0003	0.0002	1.24	-	0.0043	-/+
Q93100-3	KPBB	PHKB	Isoform 3 of Phosphorylase b kinase regulatory subunit beta	-	-	-/-	0.0024	0.0060	0.40
P17252	KPCA	PRKCA	Protein kinase C alpha type	-	-	-/-	0.0033	0.0022	1.47
P05771	KPCB	PRKCB	Protein kinase C beta type	-	-	-/-	0.0043	-	+/-
Q05655	KPCD	PRKCD	Protein kinase C delta type	-	-	-/-	0.0031	-	+/-
Q02156	KPCE	PRKCE	Protein kinase C epsilon type	-	0.0001	-/+	-	-	-/-

Q14558	KPRA	PRPSAP1	Phosphoribosyl pyrophosphate synthase-associated protein 1	0.0014	0.0116	0.12	0.0330	0.0429	0.77
O60256	KPRB	PRPSAP2	Phosphoribosyl pyrophosphate synthase-associated protein 2	0.0008	0.0018	0.41	0.0093	0.0123	0.75
P14618-2	KPYM	PKM	Isoform M1 of Pyruvate kinase PKM	0.0003	-	+/-	-	-	-/-
P14618	KPYM	PKM	Pyruvate kinase PKM	0.1431	0.0424	3.38	0.0925	0.0500	1.85
P30613-2	KPYR	PKLR	Isoform L-type of Pyruvate kinase PKLR	-	0.0049	-/+	-	0.0762	-/+
P30613	KPYR	PKLR	Pyruvate kinase PKLR	0.0416	0.0710	0.59	0.0542	0.0805	0.67
A6NCN2	KR87P	KRT87P	Putative keratin-87 protein	-	0.0018	-/+	-	-	-/-
O76011	KRT34	KRT34	Keratin, type I cuticular Ha4	0.0022	0.0144	0.16	-	-	-/-
Q92764	KRT35	KRT35	Keratin, type I cuticular Ha5	-	0.0022	-/+	-	-	-/-
O76013	KRT36	KRT36	Keratin, type I cuticular Ha6	0.0002	0.0037	0.05	-	-	-/-
Q9NSB4	KRT82	KRT82	Keratin, type II cuticular Hb2	0.0001	0.0006	0.14	-	-	-/-
P78385	KRT83	KRT83	Keratin, type II cuticular Hb3	0.0002	0.0028	0.07	-	-	-/-
Q9NSB2	KRT84	KRT84	Keratin, type II cuticular Hb4	0.0005	0.0028	0.18	-	-	-/-
P78386	KRT85	KRT85	Keratin, type II cuticular Hb5	0.0007	0.0082	0.08	-	-	-/-
O43790	KRT86	KRT86	Keratin, type II cuticular Hb6	0.0019	0.0149	0.13	-	-	-/-
Q15418	KS6A1	RPS6KA1	Ribosomal protein S6 kinase alpha-1	-	-	-/-	0.0033	0.0034	0.97
P51812	KS6A3	RPS6KA3	Ribosomal protein S6 kinase alpha-3	0.0002	-	+/-	0.0069	0.0034	2.01
Q14525	KT33B	KRT33B	Keratin, type I cuticular Ha3-II	0.0026	0.0266	0.10	-	-	-/-
Q9HA64	KT3K	FN3KRP	Ketosamine-3-kinase	0.0065	0.0023	2.79	-	-	-/-

P23919	KTHY	DTYMK	Thymidylate kinase	0.0125	0.0114	1.09	0.0069	-	+/-
Q86UP2	KTN1	KTN1	Kinectin	0.0004	0.0001	6.46	-	-	-/-
P01602	KV105	IGKV1-5	Immunoglobulin kappa variable 1-5	0.0127	0.0041	3.10	-	-	-/-
A0A0C4DH72	KV106	IGKV1-6	Immunoglobulin kappa variable 1-6	-	0.0015	-/+	-	-	-/-
A0A0C4DH67	KV108	IGKV1-8	Immunoglobulin kappa variable 1-8	0.0007	-	+/-	-	-	-/-
P01599	KV117	IGKV1-17	Immunoglobulin kappa variable 1-17	0.0027	0.0049	0.56	-	-	-/-
A0A075B6S5	KV127	IGKV1-27	Immunoglobulin kappa variable 1-27	0.0013	0.0026	0.50	-	-	-/-
P01597	KV139	IGKV1-39	Immunoglobulin kappa variable 1-39	0.0095	0.0139	0.68	-	-	-/-
A0A0C4DH68	KV224	IGKV2-24	Immunoglobulin kappa variable 2-24	0.0020	0.0015	1.35	-	-	-/-
P04433	KV311	IGKV3-11	Immunoglobulin kappa variable 3-11	0.0169	0.0042	4.03	-	-	-/-
P01624	KV315	IGKV3-15	Immunoglobulin kappa variable 3-15	0.0041	-	+/-	-	-	-/-
P01619	KV320	IGKV3-20	Immunoglobulin kappa variable 3-20	0.1165	0.0770	1.51	0.0371	0.0384	0.97
A0A075B6H7	KV37	IGKV3-7	Probable non-functional immunoglobulin kappa variable 3-7	0.0021	-	+/-	-	-	-/-
P06312	KV401	IGKV4-1	Immunoglobulin kappa variable 4-1	0.0385	0.0101	3.81	0.0371	0.0384	0.97
A0A0C4DH24	KV621	IGKV6-21	Immunoglobulin kappa variable 6-21	0.0019	-	+/-	-	-	-/-
A0A0C4DH55	KVD07	IGKV3D-7	Immunoglobulin kappa variable 3D-7	-	0.0026	-/+	-	-	-/-
A0A087WSY6	KVD15	IGKV3D-15	Immunoglobulin kappa variable 3D-15	0.0070	0.0052	1.35	-	-	-/-
P01601	KVD16	IGKV1D-16	Immunoglobulin kappa variable 1D-16	0.0036	0.0035	1.03	-	-	-/-
A0A0C4DH25	KVD20	IGKV3D-20	Immunoglobulin kappa variable 3D-20	0.0181	0.0346	0.52	-	-	-/-

P01615	KVD28	IGKV2D-28	Immunoglobulin kappa variable 2D-28	0.0010	0.0021	0.50	-	-	-/-
A0A075B6S2	KVD29	IGKV2D-29	Immunoglobulin kappa variable 2D-29	0.0058	-	+/-	-	-	-/-
A0A075B6S6	KVD30	IGKV2D-30	Immunoglobulin kappa variable 2D-30	0.0039	0.0045	0.85	-	-	-/-
P01614	KVD40	IGKV2D-40	Immunoglobulin kappa variable 2D-40	0.0045	0.0073	0.63	-	-	-/-
Q9BQD3	KXDL1	KXD1	KxDL motif-containing protein 1	-	0.0008	-/+	-	-	-/-
Q16719	KYNU	KYNU	Kynureninase	0.0019	0.0014	1.39	0.0263	0.0184	1.43
Q9H9P8	L2HDH	L2HGDH	L-2-hydroxyglutarate dehydrogenase, mitochondrial	0.0007	0.0049	0.14	0.0086	0.0198	0.44
P05455	LA	SSB	Lupus La protein	0.0042	0.0021	2.01	0.0137	0.0080	1.71
Q53H82	LACB2	LACTB2	Endoribonuclease LACTB2	0.0301	0.0106	2.82	0.0289	0.0161	1.80
P83111	LACTB	LACTB	Serine beta-lactamase-like protein LACTB, mitochondrial	0.0090	0.0069	1.31	0.0183	0.0176	1.04
O00515	LAD1	LAD1	Ladinin-1	0.0017	0.0004	3.99	0.0031	-	+/-
Q14657	LAGE3	LAGE3	EKC/KEOPS complex subunit LAGE3	-	0.0007	-/+	-	-	-/-
P24043	LAMA2	LAMA2	Laminin subunit alpha-2	0.0000	-	+/-	-	-	-/-
Q16787	LAMA3	LAMA3	Laminin subunit alpha-3	0.0003	0.0000	8.54	-	-	-/-
Q16363	LAMA4	LAMA4	Laminin subunit alpha-4	0.0014	-	+/-	0.0015	-	+/-
O15230	LAMA5	LAMA5	Laminin subunit alpha-5	0.0020	0.0001	15.55	0.0037	0.0029	1.29
P07942	LAMB1	LAMB1	Laminin subunit beta-1	0.0038	0.0006	6.37	0.0023	-	+/-
P55268	LAMB2	LAMB2	Laminin subunit beta-2	0.0034	0.0004	7.77	0.0063	0.0030	2.14
P11047	LAMC1	LAMC1	Laminin subunit gamma-1	0.0121	0.0036	3.33	0.0095	0.0030	3.20

P11279	LAMP1	LAMP1	Lysosome-associated membrane glycoprotein 1	0.0115	0.0053	2.15	0.0167	0.0120	1.39
P13473	LAMP2	LAMP2	Lysosome-associated membrane glycoprotein 2	0.0228	0.0083	2.73	0.0196	0.0149	1.31
Q9UJQ1	LAMP5	LAMP5	Lysosome-associated membrane glycoprotein 5	0.0085	-	+/-	-	-	-/-
O43813	LANC1	LANCL1	Glutathione S-transferase LANCL1	0.0006	0.0006	1.07	0.0249	0.0181	1.38
Q9NS86	LANC2	LANCL2	LanC-like protein 2	0.0001	-	+/-	0.0036	-	+/-
P42166	LAP2A	TMPO	Lamina-associated polypeptide 2, isoform alpha	0.0008	0.0004	1.76	-	-	-/-
P42167	LAP2B	TMPO	Lamina-associated polypeptide 2, isoforms beta/gamma	-	0.0006	-/+	0.0048	-	+/-
Q71RC2	LARP4	LARP4	La-related protein 4	0.0001	-	+/-	-	-	-/-
Q14847	LASP1	LASP1	LIM and SH3 domain protein 1	0.0378	0.0117	3.23	0.0266	-	+/-
P18428	LBP	LBP	Lipopolysaccharide-binding protein	0.0021	0.0006	3.78	0.0160	-	+/-
Q14739	LBR	LBR	Delta(14)-sterol reductase LBR	0.0009	0.0001	6.64	0.0069	0.0056	1.23
Q9Y383	LC7L2	LUC7L2	Putative RNA-binding protein Luc7-like 2	0.0006	-	+/-	0.0037	-	+/-
Q9UIC8	LCMT1	LCMT1	Leucine carboxyl methyltransferase 1	0.0001	-	+/-	-	-	-/-
P31025	LCN1	LCN1	Lipocalin-1	0.0028	0.0095	0.30	-	-	-/-
Q13094	LCP2	LCP2	Lymphocyte cytosolic protein 2	0.0009	0.0004	2.51	0.0034	-	+/-
Q9H6V9	LDAH	LDAH	Lipid droplet-associated hydrolase	-	0.0007	-/+	-	-	-/-
Q6ZMR3	LDH6A	LDHAL6A	L-lactate dehydrogenase A-like 6A	0.0024	0.0003	8.54	-	-	-/-

Q9BYZ2	LDH6B	LDHAL6B	L-lactate dehydrogenase A-like 6B	0.0008	-	+/-	-	-	-/-
P00338-2	LDHA	LDHA	Isoform 2 of L-lactate dehydrogenase A chain	-	0.0005	-/+	-	-	-/-
P00338-3	LDHA	LDHA	Isoform 3 of L-lactate dehydrogenase A chain	0.0003	0.0005	0.60	-	-	-/-
P00338	LDHA	LDHA	L-lactate dehydrogenase A chain	0.1344	0.2151	0.62	0.0749	0.1314	0.57
P07195	LDHB	LDHB	L-lactate dehydrogenase B chain	0.0607	0.0283	2.14	0.0690	0.0412	1.67
Q86WU2	LDHD	LDHD	Probable D-lactate dehydrogenase, mitochondrial	0.0102	0.0159	0.64	0.0191	0.0386	0.50
P09382	LEG1	LGALS1	Galectin-1	0.2622	0.3118	0.84	0.1819	0.1513	1.20
Q05315	LEG10	CLC	Galectin-10	0.0010	0.0009	1.11	-	-	-/-
P17931	LEG3	LGALS3	Galectin-3	0.0320	0.0145	2.21	0.0502	-	+/-
P56470	LEG4	LGALS4	Galectin-4	0.0732	0.0830	0.88	0.0768	0.0780	0.98
P47929	LEG7	LGALS7	Galectin-7	0.0019	0.0224	0.09	-	-	-/-
O00214	LEG8	LGALS8	Galectin-8	0.0022	0.0005	4.67	0.0082	0.0055	1.50
O00182	LEG9	LGALS9	Galectin-9	0.0018	0.0004	4.03	0.0121	-	+/-
Q3ZCW2	LEGL	LGALSL	Galectin-related protein	0.0037	0.0087	0.43	-	-	-/-
Q8NC56	LEMD2	LEMD2	LEM domain-containing protein 2	-	-	-/-	0.0105	-	+/-
P41159	LEP	LEP	Leptin	-	0.0057	-/+	-	-	-/-
O95202	LETM1	LETM1	Mitochondrial proton/calcium exchanger protein	0.0011	0.0014	0.81	0.0040	0.0027	1.45
Q08380	LG3BP	LGALS3BP	Galectin-3-binding protein	0.0093	0.0026	3.58	0.0146	0.0065	2.24

Q92604	LGAT1	LPGAT1	Acyl-CoA:lysophosphatidylglycerol acyltransferase 1	0.0028	-	+/-	0.0398	0.0134	2.98
Q99538	LGMN	LGMN	Legumain	0.0036	0.0011	3.27	0.0103	-	+/-
Q04760	LGUL	GLO1	Lactoylglutathione lyase	0.0273	0.0463	0.59	0.0160	0.0255	0.63
Q9H008	LHPP	LHPP	Phospholysine phosphohistidine inorganic pyrophosphate phosphatase	0.0117	0.0216	0.54	0.0222	0.0389	0.57
P38571	LICH	LIPA	Lysosomal acid lipase/cholesteryl ester hydrolase	-	0.0003	-/+	0.0093	-	+/-
Q9UHB6	LIMA1	LIMA1	LIM domain and actin-binding protein 1	0.0028	-	+/-	0.0094	-	+/-
Q9UGP4	LIMD1	LIMD1	LIM domain-containing protein 1	0.0016	0.0004	4.01	-	-	-/-
P48059	LIMS1	LIMS1	LIM and senescent cell antigen-like-containing domain protein 1	0.0008	0.0019	0.42	0.0098	-	+/-
P48059-2	LIMS1	LIMS1	Isoform 2 of LIM and senescent cell antigen-like-containing domain protein 1	-	-	-/-	0.0126	0.0101	1.24
Q7Z4I7	LIMS2	LIMS2	LIM and senescent cell antigen-like-containing domain protein 2	-	0.0014	-/+	0.0095	-	+/-
Q7Z4I7-2	LIMS2	LIMS2	Isoform 2 of LIM and senescent cell antigen-like-containing domain protein 2	0.0005	-	+/-	-	-	-/-
O14910	LIN7A	LIN7A	Protein lin-7 homolog A	0.0035	0.0035	0.98	-	-	-/-
Q9NUP9	LIN7C	LIN7C	Protein lin-7 homolog C	0.0019	0.0020	0.96	-	-	-/-
Q86W92	LIPB1	PPFIBP1	Liprin-beta-1	0.0001	-	+/-	0.0014	-	+/-
P11150	LIPC	LIPC	Hepatic triacylglycerol lipase	-	-	-/-	-	0.0060	-/+

P43034	LIS1	PAFAH1B1	Platelet-activating factor acetylhydrolase IB subunit alpha	0.0058	0.0028	2.10	0.0166	0.0123	1.35
P09960	LKHA4	LTA4H	Leukotriene A-4 hydrolase	0.0152	0.0116	1.32	0.0335	0.0310	1.08
Q9H0V9	LMA2L	LMAN2L	VIP36-like protein	-	-	-/-	-	0.0055	-/+
P49257	LMAN1	LMAN1	Protein ERGIC-53	0.0241	0.0161	1.50	0.0434	0.0456	0.95
Q12907	LMAN2	LMAN2	Vesicular integral-membrane protein VIP36	0.0643	0.0290	2.22	0.0885	0.0689	1.29
Q9NZU5	LMCD1	LMCD1	LIM and cysteine-rich domains protein 1	0.0003	0.0002	1.37	-	-	-/-
Q9BU23	LMF2	LMF2	Lipase maturation factor 2	-	-	-/-	0.0084	0.0050	1.68
P02545-2	LMNA	LMNA	Isoform C of Prelamin-A/C	0.0509	0.0154	3.30	0.1277	0.0518	2.46
P02545	LMNA	LMNA	Prelamin-A/C	0.2014	0.0477	4.22	0.0975	0.0520	1.87
P20700	LMNB1	LMNB1	Lamin-B1	0.0557	0.0176	3.17	0.0329	0.0122	2.70
Q03252	LMNB2	LMNB2	Lamin-B2	0.0191	0.0071	2.68	0.0150	0.0094	1.60
Q8WWI1	LMO7	LMO7	LIM domain only protein 7	0.0004	0.0001	3.24	0.0011	-	+/-
Q8WWI1-3	LMO7	LMO7	Isoform 3 of LIM domain only protein 7	0.0003	-	+/-	-	-	-/-
Q9C0E8	LNP	LNPK	Endoplasmic reticulum junction formation protein lunapark	-	-	-/-	0.0066	0.0044	1.49
P36776	LONM	LONP1	Lon protease homolog, mitochondrial	0.0115	0.0075	1.52	0.0228	0.0257	0.89
Q86WA8	LONP2	LONP2	Lon protease homolog 2, peroxisomal	-	-	-/-	-	0.0019	-/+
P09917	LOX5	ALOX5	Polyunsaturated fatty acid 5-lipoxygenase	-	-	-/-	0.0083	-	+/-
Q08397	LOXL1	LOXL1	Lysyl oxidase homolog 1	0.0001	-	+/-	0.0106	0.0109	0.97
Q9Y4K0	LOXL2	LOXL2	Lysyl oxidase homolog 2	0.0002	-	+/-	-	-	-/-

Q93052	LPP	LPP	Lipoma-preferred partner	0.0038	0.0104	0.37	0.0111	0.0089	1.24
Q86U10	LPP60	ASPG	60 kDa lysophospholipase	-	0.0018	-/+	-	0.0127	-/+
P42704	LPPRC	LRPPRC	Leucine-rich PPR motif-containing protein, mitochondrial	0.0102	0.0094	1.07	0.0275	0.0278	0.99
O60711	LPXN	LPXN	Leupaxin	0.0016	0.0006	2.83	-	-	-/-
Q0VAA2	LR74A	LRRC74A	Leucine-rich repeat-containing protein 74A	0.0001	-	+/-	-	-	-/-
P50851	LRBA	LRBA	Lipopolysaccharide-responsive and beige-like anchor protein	-	-	-/-	0.0037	0.0030	1.22
Q9H9A6	LRC40	LRRC40	Leucine-rich repeat-containing protein 40	-	0.0009	-/+	0.0070	0.0042	1.67
Q96CN5	LRC45	LRRC45	Leucine-rich repeat-containing protein 45	0.0001	0.0005	0.13	-	-	-/-
Q8N1G4	LRC47	LRRC47	Leucine-rich repeat-containing protein 47	0.0037	0.0031	1.17	0.0076	0.0093	0.82
Q8N9N7	LRC57	LRRC57	Leucine-rich repeat-containing protein 57	0.0009	0.0021	0.43	-	-	-/-
Q96AG4	LRC59	LRRC59	Leucine-rich repeat-containing protein 59	0.0019	0.0017	1.15	0.0183	0.0091	2.02
Q96JM4	LRIQ1	LRRIQ1	Leucine-rich repeat and IQ domain-containing protein 1	-	-	-/-	-	0.0008	-/+
Q9H2I8	LRMDA	LRMDA	Leucine-rich melanocyte differentiation-associated protein	0.0011	-	+/-	-	-	-/-
Q07954	LRP1	LRP1	Prolow-density lipoprotein receptor-related protein 1	0.0069	0.0018	3.73	0.0105	0.0058	1.79
Q9NZR2	LRP1B	LRP1B	Low-density lipoprotein receptor-related protein 1B	-	-	-/-	-	0.0006	-/+
Q9BTT6	LRRC1	LRRC1	Leucine-rich repeat-containing protein 1	0.0011	-	+/-	-	-	-/-

Q32MZ4	LRRF1	LRRFIP1	Leucine-rich repeat flightless-interacting protein 1	0.0001	0.0001	1.04	-	-	-/-
Q5S007	LRRK2	LRRK2	Leucine-rich repeat serine/threonine-protein kinase 2	0.0024	0.0004	6.13	-	-	-/-
Q6UWE0	LRSM1	LRSAM1	E3 ubiquitin-protein ligase LRSAM1	0.0001	-	+/-	-	-	-/-
Q9UFC0	LRWD1	LRWD1	Leucine-rich repeat and WD repeat-containing protein 1	-	0.0002	-/+	-	-	-/-
Q8ND56	LS14A	LSM14A	Protein LSM14 homolog A	0.0005	-	+/-	-	-	-/-
O15116	LSM1	LSM1	U6 snRNA-associated Sm-like protein LSm1	0.0018	0.0047	0.39	-	-	-/-
Q3MHD2	LSM12	LSM12	Protein LSM12 homolog	0.0020	0.0026	0.77	-	-	-/-
Q9Y333	LSM2	LSM2	U6 snRNA-associated Sm-like protein LSm2	0.0129	0.0107	1.21	-	-	-/-
P62310	LSM3	LSM3	U6 snRNA-associated Sm-like protein LSm3	0.0025	0.0072	0.35	-	-	-/-
Q9Y4Z0	LSM4	LSM4	U6 snRNA-associated Sm-like protein LSm4	-	0.0007	-/+	-	-	-/-
Q9Y4Y9	LSM5	LSM5	U6 snRNA-associated Sm-like protein LSm5	0.0019	0.0157	0.12	-	-	-/-
P62312	LSM6	LSM6	U6 snRNA-associated Sm-like protein LSm6	-	0.0037	-/+	-	-	-/-
Q9UK45	LSM7	LSM7	U6 snRNA-associated Sm-like protein LSm7	0.0017	0.0090	0.19	-	-	-/-
O95777	LSM8	LSM8	U6 snRNA-associated Sm-like protein LSm8	0.0106	0.0227	0.47	-	-	-/-
Q9BRA0	LSMD1	NAA38	N-alpha-acetyltransferase 38, NatC auxiliary subunit	-	0.0011	-/+	-	-	-/-
P33241	LSP1	LSP1	Lymphocyte-specific protein 1	0.0051	0.0011	4.82	0.0056	-	+/-
Q86X29	LSR	LSR	Lipolysis-stimulated lipoprotein receptor	-	-	-/-	0.0026	-	+/-

Q9BVC4	LST8	MLST8	Target of rapamycin complex subunit LST8	0.0014	0.0005	2.93	-	-	-/-
Q14766	LTBP1	LTBP1	Latent-transforming growth factor beta-binding protein 1	-	-	-/-	0.0014	-	+/-
Q14767	LTBP2	LTBP2	Latent-transforming growth factor beta-binding protein 2	0.0001	-	+/-	0.0038	-	+/-
Q8N2S1	LTBP4	LTBP4	Latent-transforming growth factor beta-binding protein 4	0.0018	0.0003	5.33	0.0037	-	+/-
Q6IAA8	LTOR1	LAMTOR1	Ragulator complex protein LAMTOR1	0.0189	0.0289	0.65	-	-	-/-
Q9Y2Q5	LTOR2	LAMTOR2	Ragulator complex protein LAMTOR2	0.0140	0.0288	0.49	0.0603	0.0623	0.97
Q9UHA4	LTOR3	LAMTOR3	Ragulator complex protein LAMTOR3	0.0114	0.0102	1.12	0.0169	-	+/-
Q0VGL1	LTOR4	LAMTOR4	Ragulator complex protein LAMTOR4	0.0060	0.0023	2.68	-	-	-/-
O43504	LTOR5	LAMTOR5	Ragulator complex protein LAMTOR5	0.0081	0.0049	1.66	-	-	-/-
Q96GA3	LTV1	LTV1	Protein LTV1 homolog	0.0012	-	+/-	-	-	-/-
P51884	LUM	LUM	Lumican	0.1169	0.0477	2.45	0.0691	0.0462	1.49
Q86V48	LUZP1	LUZP1	Leucine zipper protein 1	0.0001	-	+/-	-	-	-/-
P01703	LV140	IGLV1-40	Immunoglobulin lambda variable 1-40	0.0033	-	+/-	-	-	-/-
P01700	LV147	IGLV1-47	Immunoglobulin lambda variable 1-47	0.0346	0.0132	2.62	-	-	-/-
P01701	LV151	IGLV1-51	Immunoglobulin lambda variable 1-51	0.0077	-	+/-	-	-	-/-
P01709	LV208	IGLV2-8	Immunoglobulin lambda variable 2-8	0.0054	-	+/-	-	-	-/-
P01704	LV214	IGLV2-14	Immunoglobulin lambda variable 2-14	0.0087	-	+/-	-	-	-/-
A0A075B6K4	LV310	IGLV3-10	Immunoglobulin lambda variable 3-10	0.0070	-	+/-	-	-	-/-

P01714	LV319	IGLV3-19	Immunoglobulin lambda variable 3-19	0.0150	-	+/-	-	-	-/-
P80748	LV321	IGLV3-21	Immunoglobulin lambda variable 3-21	0.0971	0.0041	23.49	0.0603	-	+/-
P01717	LV325	IGLV3-25	Immunoglobulin lambda variable 3-25	0.0038	-	+/-	-	-	-/-
P01721	LV657	IGLV6-57	Immunoglobulin lambda variable 6-57	0.0076	0.0049	1.55	-	-	-/-
A0A075B6I9	LV746	IGLV7-46	Immunoglobulin lambda variable 7-46	0.0038	-	+/-	-	-	-/-
A0A075B6I0	LV861	IGLV8-61	Immunoglobulin lambda variable 8-61	0.0075	-	+/-	-	-	-/-
Q9BS40	LXN	LXN	Latexin	0.0092	0.0015	5.97	0.0147	0.0096	1.53
P10253	LYAG	GAA	Lysosomal alpha-glucosidase	0.0156	0.0155	1.01	0.0278	0.0247	1.12
P07948	LYN	LYN	Tyrosine-protein kinase Lyn	0.0010	-	+/-	0.0161	0.0029	5.56
P07948-2	LYN	LYN	Isoform 2 of Tyrosine-protein kinase Lyn	-	-	-/-	0.0194	-	+/-
O75608	LYPA1	LYPLA1	Acyl-protein thioesterase 1	0.0221	0.0254	0.87	0.0287	0.0517	0.55
O95372	LYPA2	LYPLA2	Acyl-protein thioesterase 2	0.0088	0.0148	0.60	0.0103	-	+/-
Q5VWZ2	LYPL1	LYPLAL1	Lysophospholipase-like protein 1	0.0184	0.0097	1.89	0.0405	0.0419	0.97
Q86UE4	LYRIC	MTDH	Protein LYRIC	0.0004	0.0004	0.96	0.0096	0.0069	1.38
Q9HD34	LYRM4	LYRM4	LYR motif-containing protein 4	-	0.0005	-/+	-	-	-/-
Q5U5X0	LYRM7	LYRM7	Complex III assembly factor LYRM7	-	0.0005	-/+	-	-	-/-
P61626	LYSC	LYZ	Lysozyme C	0.0144	0.0057	2.51	0.0986	0.0184	5.37
Q8IV50	LYSM2	LYSMD2	LysM and putative peptidoglycan-binding domain-containing protein 2	0.0002	-	+/-	-	-	-/-
Q8WZA0	LZIC	LZIC	Protein LZIC	0.0020	0.0094	0.22	-	-	-/-

Q9NQ48	LZTL1	LZTFL1	Leucine zipper transcription factor-like protein 1	0.0012	0.0037	0.32	-	-	-/-
Q9NPA3	M1IP1	MID1IP1	Mid1-interacting protein 1	0.0008	-	+/-	-	-	-/-
Q9UI17	M2GD	DMGDH	Dimethylglycine dehydrogenase, mitochondrial	0.0090	0.0295	0.30	0.0020	0.0765	0.03
Q02978	M2OM	SLC25A11	Mitochondrial 2-oxoglutarate/malate carrier protein	0.0178	0.0328	0.54	0.0437	0.0379	1.15
Q9NYL2	M3K20	MAP3K20	Mitogen-activated protein kinase kinase kinase 20	0.0001	-	+/-	-	-	-/-
Q12851	M4K2	MAP4K2	Mitogen-activated protein kinase kinase kinase kinase 2	0.0002	-	+/-	-	-	-/-
O95819	M4K4	MAP4K4	Mitogen-activated protein kinase kinase kinase kinase 4	-	-	-/-	0.0011	-	+/-
P33908	MA1A1	MAN1A1	Mannosyl-oligosaccharide 1,2-alpha-mannosidase IA	-	-	-/-	0.0055	-	+/-
O60476	MA1A2	MAN1A2	Mannosyl-oligosaccharide 1,2-alpha-mannosidase IB	-	-	-/-	0.0037	-	+/-
Q16706	MA2A1	MAN2A1	Alpha-mannosidase 2	0.0006	0.0001	4.88	0.0154	0.0050	3.06
O00754	MA2B1	MAN2B1	Lysosomal alpha-mannosidase	0.0016	0.0003	4.72	0.0094	-	+/-
Q9Y2E5	MA2B2	MAN2B2	Epididymis-specific alpha-mannosidase	0.0001	-	+/-	0.0024	-	+/-
Q9NTJ4	MA2C1	MAN2C1	Alpha-mannosidase 2C1	-	-	-/-	0.0022	0.0035	0.64
Q96T17	MA7D2	MAP7D2	MAP7 domain-containing protein 2	0.0001	-	+/-	-	-	-/-
O43708	MAAI	GSTZ1	Maleylacetoacetate isomerase	0.0234	0.0661	0.35	0.0088	0.0546	0.16

Q9BQ69	MACD1	MACROD1	ADP-ribose glycohydrolase MACROD1	0.0021	0.0025	0.86	-	0.0042	-/+
Q9UPN3	MACF1	MACF1	Microtubule-actin cross-linking factor 1, isoforms 1/2/3/5	0.0002	0.0000	4.03	0.0009	0.0004	2.19
O15525	MAFG	MAFG	Transcription factor MafG	0.0002	-	+/-	-	-	-/-
Q9H0U3	MAGT1	MAGT1	Magnesium transporter protein 1	0.0021	-	+/-	0.0120	0.0090	1.33
Q8WWC4	MAIP1	MAIP1	m-AAA protease-interacting protein 1, mitochondrial	0.0008	0.0006	1.27	0.0090	0.0060	1.50
Q9BXY0	MAK16	MAK16	Protein MAK16 homolog	-	-	-/-	0.0034	-	+/-
P55145	MANF	MANF	Mesencephalic astrocyte-derived neurotrophic factor	0.0065	0.0087	0.74	0.0207	0.0096	2.16
P23368	MAOM	ME2	NAD-dependent malic enzyme, mitochondrial	0.0035	0.0006	5.58	0.0155	0.0083	1.88
P48163	MAOX	ME1	NADP-dependent malic enzyme	0.0026	0.0016	1.59	0.0083	0.0125	0.66
P53582	MAP11	METAP1	Methionine aminopeptidase 1	0.0001	0.0012	0.12	-	-	-/-
Q6UB28	MAP12	METAP1D	Methionine aminopeptidase 1D, mitochondrial	0.0007	0.0004	1.77	-	-	-/-
P46821	MAP1B	MAP1B	Microtubule-associated protein 1B	0.0002	-	+/-	-	-	-/-
Q66K74	MAP1S	MAP1S	Microtubule-associated protein 1S	0.0006	-	+/-	-	-	-/-
P50579	MAP2	METAP2	Methionine aminopeptidase 2	0.0008	0.0011	0.79	0.0043	-	+/-
P27816	MAP4	MAP4	Microtubule-associated protein 4	0.0006	0.0002	3.78	0.0047	-	+/-
Q5VT66	MARC1	MARC1	Mitochondrial amidoxime-reducing component 1	0.0013	0.0010	1.28	-	0.0110	-/+

Q969Z3	MARC2	MARC2	Mitochondrial amidoxime reducing component 2	0.0022	0.0068	0.33	0.0152	0.0219	0.69
Q9UEW3	MARCO	MARCO	Macrophage receptor MARCO	0.0001	-	+/-	-	0.0037	-/+
P29966	MARCS	MARCKS	Myristoylated alanine-rich C-kinase substrate	0.0024	-	+/-	0.0043	-	+/-
Q15691	MARE1	MAPRE1	Microtubule-associated protein RP/EB family member 1	0.0178	0.0142	1.25	0.0194	0.0111	1.75
Q15555	MARE2	MAPRE2	Microtubule-associated protein RP/EB family member 2	0.0023	0.0022	1.02	0.0056	-	+/-
Q9UPY8	MARE3	MAPRE3	Microtubule-associated protein RP/EB family member 3	-	0.0029	-/+	0.0096	-	+/-
Q9NX47	MARH5	MARCHF5	E3 ubiquitin-protein ligase MARCHF5	-	-	-/-	0.0064	0.0066	0.97
O00187	MASP2	MASP2	Mannan-binding lectin serine protease 2	0.0013	0.0005	2.51	0.0073	-	+/-
Q9NZL9	MAT2B	MAT2B	Methionine adenosyltransferase 2 subunit beta	0.0071	0.0164	0.43	0.0116	0.0193	0.60
O00339	MATN2	MATN2	Matrilin-2	0.0004	-	+/-	-	-	-/-
P43243	MATR3	MATR3	Matrin-3	0.0051	0.0029	1.72	0.0077	0.0072	1.07
Q7Z434	MAVS	MAVS	Mitochondrial antiviral-signaling protein	0.0002	0.0007	0.22	0.0054	0.0120	0.45
Q9Y586	MB212	MAB21L2	Protein mab-21-like 2	-	-	-/-	-	0.0008	-/+
Q9BQG0	MBB1A	MYBBP1A	Myb-binding protein 1A	-	-	-/-	0.0022	0.0011	1.96
P11226	MBL2	MBL2	Mannose-binding protein C	0.0213	0.0157	1.36	0.0169	0.0080	2.12
A4D2B0	MBLC1	MBLAC1	Metallo-beta-lactamase domain-containing protein 1	-	0.0008	-/+	-	-	-/-

Q68D91	MBLC2	MBLAC2	Metallo-beta-lactamase domain-containing protein 2	0.0039	0.0010	3.79	-	-	-/-
Q9NR56	MBNL1	MBNL1	Muscleblind-like protein 1	0.0002	0.0003	0.54	-	-	-/-
Q5VZF2	MBNL2	MBNL2	Muscleblind-like protein 2	0.0009	-	+/-	-	-	-/-
Q6P1A2	MBOA5	LPCAT3	Lysophospholipid acyltransferase 5	0.0016	0.0008	2.14	0.0185	0.0114	1.62
O43324	MCA3	EEF1E1	Eukaryotic translation elongation factor 1 epsilon-1	0.0118	0.0099	1.19	0.0332	0.0113	2.94
O43772	MCAT	SLC25A20	Mitochondrial carnitine/acylcarnitine carrier protein	0.0329	0.0497	0.66	0.0718	0.1652	0.43
Q96RQ3	MCCA	MCCC1	Methylcrotonoyl-CoA carboxylase subunit alpha, mitochondrial	0.0060	0.0129	0.46	0.0158	0.0320	0.49
Q9HCC0	MCCB	MCCC2	Methylcrotonoyl-CoA carboxylase beta chain, mitochondrial	0.0155	0.0140	1.11	0.0458	0.0634	0.72
Q96PE7	MCEE	MCEE	Methylmalonyl-CoA epimerase, mitochondrial	0.0060	0.0186	0.32	-	-	-/-
Q8NI22	MCFD2	MCFD2	Multiple coagulation factor deficiency protein 2	-	-	-/-	-	0.0055	-/+
P25205	MCM3	MCM3	DNA replication licensing factor MCM3	0.0002	-	+/-	-	-	-/-
Q9BTE3	MCMBP	MCMBP	Mini-chromosome maintenance complex-binding protein	0.0004	-	+/-	-	-	-/-
Q9BUT9	MCRI2	MCRIP2	MAPK regulated corepressor interacting protein 2	0.0010	-	+/-	-	-	-/-
Q9ULC4	MCTS1	MCTS1	Malignant T-cell-amplified sequence 1	0.0069	0.0112	0.62	0.0196	0.0091	2.15

Q8NE86	MCU	MCU	Calcium uniporter protein, mitochondrial	0.0004	-	+/-	0.0093	-	+/-
Q96AQ8	MCUR1	MCUR1	Mitochondrial calcium uniporter regulator 1	-	0.0004	-/+	-	-	-/-
Q9Y6D9	MD1L1	MAD1L1	Mitotic spindle assembly checkpoint protein MAD1	0.0003	0.0001	1.88	-	-	-/-
P40925	MDHC	MDH1	Malate dehydrogenase, cytoplasmic	0.0549	0.0602	0.91	0.0590	0.0744	0.79
P40926	MDHM	MDH2	Malate dehydrogenase, mitochondrial	0.1383	0.1950	0.71	0.1356	0.1427	0.95
P08183	MDR1	ABCB1	ATP-dependent translocase ABCB1	0.0002	0.0001	3.37	0.0034	-	+/-
P21439	MDR3	ABCB4	Phosphatidylcholine translocator ABCB4	0.0001	-	+/-	0.0021	0.0029	0.72
P51608	MECP2	MECP2	Methyl-CpG-binding protein 2	0.0001	-	+/-	0.0031	-	+/-
Q9BV79	MECR	MECR	Enoyl-[acyl-carrier-protein] reductase, mitochondrial	0.0008	0.0011	0.72	-	0.0056	-/+
Q9H944	MED20	MED20	Mediator of RNA polymerase II transcription subunit 20	0.0004	-	+/-	-	-	-/-
O75095	MEGF6	MEGF6	Multiple epidermal growth factor-like domains protein 6	0.0002	-	+/-	-	-	-/-
Q9Y316	MEMO1	MEMO1	Protein MEMO1	0.0036	0.0017	2.11	0.0155	0.0263	0.59
Q9BQA1	MEP50	WDR77	Methylosome protein 50	0.0029	0.0054	0.53	-	-	-/-
Q14696	MESD	MESD	LRP chaperone MESD	0.0064	0.0069	0.92	0.0133	0.0050	2.64
Q8N4P3	MESH1	HDDC3	Guanosine-3',5'-bis(diphosphate) 3'-pyrophosphohydrolase MESH1	0.0066	0.0089	0.74	0.0077	0.0080	0.97
Q9H7H0	MET17	METTL17	Methyltransferase-like protein 17, mitochondrial	0.0002	-	+/-	-	-	-/-

Q9H8H3	MET7A	METTL7A	Methyltransferase-like protein 7A	0.0575	0.0622	0.92	0.0674	0.0727	0.93
Q6UX53	MET7B	METTL7B	Methyltransferase-like protein 7B	0.0138	0.0237	0.58	0.0792	0.0783	1.01
Q00266	METK1	MAT1A	S-adenosylmethionine synthase isoform type-1	0.0505	0.0794	0.64	0.0290	0.0729	0.40
P31153	METK2	MAT2A	S-adenosylmethionine synthase isoform type-2	0.0121	0.0091	1.32	0.0202	0.0228	0.88
Q14CX5	MF13A	MFSD13A	Transmembrane protein 180	-	0.0004	-/+	-	-	-/-
P55081	MFAP1	MFAP1	Microfibrillar-associated protein 1	-	0.0002	-/+	-	-	-/-
P55001	MFAP2	MFAP2	Microfibrillar-associated protein 2	0.0028	-	+/-	0.0254	-	+/-
P55083	MFAP4	MFAP4	Microfibril-associated glycoprotein 4	0.0154	0.0062	2.47	0.0375	0.0227	1.66
Q13361	MFAP5	MFAP5	Microfibrillar-associated protein 5	0.0017	-	+/-	-	-	-/-
Q9GZY8	MFF	MFF	Mitochondrial fission factor	-	-	-/-	0.0049	0.0050	0.97
O95140	MFN2	MFN2	Mitofusin-2	0.0002	-	+/-	-	-	-/-
Q9H019	MFR1L	MTFR1L	Mitochondrial fission regulator 1-like	0.0002	0.0011	0.17	-	-	-/-
P26572	MGAT1	MGAT1	Alpha-1,3-mannosyl-glycoprotein 2-beta-N-acetylglucosaminyltransferase	0.0009	-	+/-	0.0097	0.0057	1.68
Q10469	MGAT2	MGAT2	Alpha-1,6-mannosyl-glycoprotein 2-beta-N-acetylglucosaminyltransferase	-	-	-/-	0.0053	-	+/-
Q86V88	MGDP1	MDP1	Magnesium-dependent phosphatase 1	0.0018	0.0009	2.07	-	-	-/-
Q99685	MGLL	MGLL	Monoglyceride lipase	0.0208	0.0355	0.59	0.0169	0.0354	0.48
P16455	MGMT	MGMT	Methylated-DNA--protein-cysteine methyltransferase	0.0032	0.0063	0.51	-	0.0181	-/+

P61326	MGN	MAGOH	Protein mago nashi homolog	0.0180	0.0162	1.11	0.0164	-	+/-
P10620	MGST1	MGST1	Microsomal glutathione S-transferase 1	0.2010	0.1534	1.31	0.3426	0.3024	1.13
Q99735	MGST2	MGST2	Microsomal glutathione S-transferase 2	-	-	-/-	0.0215	0.0138	1.57
O14880	MGST3	MGST3	Microsomal glutathione S-transferase 3	0.0114	0.0119	0.96	0.1661	0.1615	1.03
Q96PC5	MIA2	MIA2	Melanoma inhibitory activity protein 2	0.0004	-	+/-	0.0019	0.0020	0.97
Q5TGZ0	MIC10	MICOS10	MICOS complex subunit MIC10	-	0.0024	-/+	-	-	-/-
Q5XKP0	MIC13	MICOS13	MICOS complex subunit MIC13	0.0016	0.0034	0.47	-	-	-/-
Q9NX63	MIC19	CHCHD3	MICOS complex subunit MIC19	0.0092	0.0096	0.96	0.0148	0.0070	2.11
Q9BRQ6	MIC25	CHCHD6	MICOS complex subunit MIC25	0.0017	0.0033	0.50	-	-	-/-
Q9BUR5	MIC26	APOO	MICOS complex subunit MIC26	0.0042	0.0080	0.53	0.0098	0.0161	0.61
Q6UXV4	MIC27	APOOL	MICOS complex subunit MIC27	0.0057	0.0042	1.37	0.0071	0.0060	1.19
Q16891-2	MIC60	IMMT	Isoform 2 of MICOS complex subunit MIC60	0.0128	0.0054	2.38	-	-	-/-
Q16891-4	MIC60	IMMT	Isoform 4 of MICOS complex subunit MIC60	0.0117	0.0029	4.03	-	-	-/-
Q16891	MIC60	IMMT	MICOS complex subunit MIC60	0.0230	0.0047	4.95	0.0139	0.0200	0.70
Q8TDZ2	MICA1	MICAL1	[F-actin]-monooxygenase MICAL1	0.0001	-	+/-	0.0017	-	+/-
Q9BRT3	MIEN1	MIEN1	Migration and invasion enhancer 1	0.0036	0.0068	0.53	-	-	-/-
P14174	MIF	MIF	Macrophage migration inhibitory factor	0.0643	0.0709	0.91	-	0.0083	-/+
Q7Z6M3	MILR1	MILR1	Allergin-1	0.0022	-	+/-	-	-	-/-
P20774	MIME	OGN	Mimecan	0.0550	0.0220	2.50	0.0463	0.0267	1.74

Q8N5J2	MINY1	MINDY1	Ubiquitin carboxyl-terminal hydrolase MINDY-1	0.0005	0.0013	0.40	0.0060	-	+/-
Q8N5J2-2	MINY1	MINDY1	Isoform 2 of Ubiquitin carboxyl-terminal hydrolase MINDY-1	-	-	-/-	0.0084	-	+/-
Q99797	MIPEP	MIPEP	Mitochondrial intermediate peptidase	-	0.0001	-/+	-	-	-/-
Q8IXI2	MIRO1	RHOT1	Mitochondrial Rho GTPase 1	0.0010	-	+/-	-	-	-/-
Q8IXI1	MIRO2	RHOT2	Mitochondrial Rho GTPase 2	0.0015	0.0002	8.10	0.0041	0.0035	1.19
P28482	MK01	MAPK1	Mitogen-activated protein kinase 1	0.0070	0.0063	1.11	0.0341	0.0165	2.06
P27361	MK03	MAPK3	Mitogen-activated protein kinase 3	0.0023	0.0039	0.58	0.0181	0.0047	3.89
P45983	MK08	MAPK8	Mitogen-activated protein kinase 8	-	0.0004	-/+	-	-	-/-
Q16539	MK14	MAPK14	Mitogen-activated protein kinase 14	0.0044	0.0035	1.25	0.0116	-	+/-
Q9HBH9	MKNK2	MKNK2	MAP kinase-interacting serine/threonine-protein kinase 2	-	-	-/-	0.0009	-	+/-
P19105	ML12A	MYL12A	Myosin regulatory light chain 12A	0.0295	0.0220	1.34	-	-	-/-
O14950	ML12B	MYL12B	Myosin regulatory light chain 12B	0.1522	0.0772	1.97	0.1250	0.0553	2.26
Q14165	MLEC	MLEC	Malectin	0.0274	0.0143	1.91	0.0412	0.0380	1.09
Q15773	MLF2	MLF2	Myeloid leukemia factor 2	0.0069	0.0068	1.02	-	-	-/-
Q9GZQ8	MLP3B	MAP1LC3B	Microtubule-associated proteins 1A/1B light chain 3B	0.0004	-	+/-	-	-	-/-
Q96A32	MLRS	MYLPF	Myosin regulatory light chain 2, skeletal muscle isoform	0.0002	-	+/-	-	-	-/-
Q9UH92	MLX	MLX	Max-like protein X	-	0.0003	-/+	-	-	-/-

Q8IVH4	MMAA	MMAA	Methylmalonic aciduria type A protein, mitochondrial	0.0026	0.0070	0.37	0.0095	0.0098	0.97
Q96EY8	MMAB	MMAB	Corrinoid adenosyltransferase	0.0054	0.0084	0.64	-	0.0099	-/+
Q9Y4U1	MMAC	MMACHC	Cyanocobalamin reductase / alkylcobalamin dealkylase	-	0.0004	-/+	-	-	-/-
P22894	MMP8	MMP8	Neutrophil collagenase	0.0067	-	+/-	0.0147	-	+/-
P14780	MMP9	MMP9	Matrix metalloproteinase-9	0.0165	0.0020	8.11	0.0395	-	+/-
Q9H8L6	MMRN2	MMRN2	Multimerin-2	0.0006	-	+/-	-	-	-/-
Q02252	MMSA	ALDH6A1	Methylmalonate-semialdehyde dehydrogenase [acylating], mitochondrial	0.0951	0.1735	0.55	0.0897	0.2659	0.34
P41218	MNDA	MNDA	Myeloid cell nuclear differentiation antigen	0.0119	0.0003	41.75	0.0160	-	+/-
Q7L9L4	MOB1B	MOB1B	MOB kinase activator 1B	0.0005	0.0012	0.44	-	-	-/-
Q96BX8	MOB3A	MOB3A	MOB kinase activator 3A	-	0.0006	-/+	-	-	-/-
Q13875	MOBP	MOBP	Myelin-associated oligodendrocyte basic protein	0.0004	-	+/-	-	-	-/-
O96033	MOC2A	MOCS2	Molybdopterin synthase sulfur carrier subunit	0.0122	0.0121	1.00	-	-	-/-
O96007	MOC2B	MOCS2	Molybdopterin synthase catalytic subunit	0.0061	0.0032	1.88	-	-	-/-
Q96EN8	MOCOS	MOCOS	Molybdenum cofactor sulfurase	-	-	-/-	-	0.0025	-/+
Q9NZB8	MOCS1	MOCS1	Molybdenum cofactor biosynthesis protein 1	-	0.0001	-/+	-	-	-/-
O95396	MOCS3	MOCS3	Adenylyltransferase and sulfurtransferase MOCS3	0.0010	0.0004	2.64	-	-	-/-
P26038	MOES	MSN	Moesin	0.0520	0.0224	2.33	0.0636	0.0441	1.44

Q9HD47	MOG1	RANGRF	Ran guanine nucleotide release factor	-	0.0011	-/+	-	-	-/-
Q13724	MOGS	MOGS	Mannosyl-oligosaccharide glucosidase	0.0113	0.0093	1.21	0.0302	0.0380	0.80
P36021	MOT8	SLC16A2	Monocarboxylate transporter 8	-	0.0003	-/+	-	-	-/-
Q9HCE1	MOV10	MOV10	Helicase MOV-10	0.0002	-	+/-	0.0038	0.0031	1.22
Q6UVY6	MOXD1	MOXD1	DBH-like monooxygenase protein 1	0.0016	-	+/-	-	-	-/-
Q02750	MP2K1	MAP2K1	Dual specificity mitogen-activated protein kinase kinase 1	0.0054	0.0058	0.94	0.0133	0.0138	0.97
P36507	MP2K2	MAP2K2	Dual specificity mitogen-activated protein kinase kinase 2	0.0079	0.0051	1.55	-	-	-/-
P46734	MP2K3	MAP2K3	Dual specificity mitogen-activated protein kinase kinase 3	0.0002	-	+/-	0.0068	0.0070	0.97
P45985	MP2K4	MAP2K4	Dual specificity mitogen-activated protein kinase kinase 4	-	-	-/-	0.0088	0.0067	1.32
Q9Y5U8	MPC1	MPC1	Mitochondrial pyruvate carrier 1	0.0026	0.0012	2.13	0.0240	0.0507	0.47
O95563	MPC2	MPC2	Mitochondrial pyruvate carrier 2	0.0175	0.0463	0.38	0.0392	0.1047	0.37
Q00325-2	MPCP	SLC25A3	Isoform B of Phosphate carrier protein, mitochondrial	0.0049	-	+/-	0.0515	0.0640	0.80
Q00325	MPCP	SLC25A3	Phosphate carrier protein, mitochondrial	0.0223	0.0068	3.26	0.0310	0.0538	0.58
O75970	MPDZ	MPDZ	Multiple PDZ domain protein	0.0005	0.0007	0.73	-	0.0011	-/+
Q99547	MPH6	MPHOSPH6	M-phase phosphoprotein 6	-	0.0007	-/+	-	-	-/-
P34949	MPI	MPI	Mannose-6-phosphate isomerase	0.0025	0.0059	0.42	0.0128	0.0096	1.34
Q9NZW5	MPP6	MPP6	MAGUK p55 subfamily member 6	0.0005	0.0002	2.92	0.0091	0.0029	3.11

Q10713	MPPA	PMPCA	Mitochondrial-processing peptidase subunit alpha	0.0119	0.0073	1.64	0.0097	0.0197	0.49
O75439	MPPB	PMPCB	Mitochondrial-processing peptidase subunit beta	0.0088	0.0079	1.13	0.0054	0.0143	0.38
P20645	MPRD	M6PR	Cation-dependent mannose-6-phosphate receptor	0.0023	0.0004	6.06	0.0112	-	+/-
P11717	MPRI	IGF2R	Cation-independent mannose-6-phosphate receptor	0.0008	0.0003	2.52	0.0012	0.0012	0.97
Q6WCQ1	MPRIP	MPRIP	Myosin phosphatase Rho-interacting protein	0.0002	-	+/-	0.0012	-	+/-
O75352	MPU1	MPDU1	Mannose-P-dolichol utilization defect 1 protein	0.0045	0.0014	3.22	0.0133	-	+/-
P22897	MRC1	MRC1	Macrophage mannose receptor 1	0.0001	0.0001	1.29	0.0026	0.0034	0.77
Q9UBG0	MRC2	MRC2	C-type mannose receptor 2	0.0001	-	+/-	0.0023	-	+/-
Q5VT25	MRCKA	CDC42BPA	Serine/threonine-protein kinase MRCK alpha	-	-	-/-	-	0.0008	-/+
P49959	MRE11	MRE11	Double-strand break repair protein MRE11	0.0009	0.0004	2.01	0.0020	-	+/-
Q9P0P8	MRES1	MTRES1	Mitochondrial transcription rescue factor 1	0.0002	0.0006	0.38	-	-	-/-
Q9HC36	MRM3	MRM3	rRNA methyltransferase 3, mitochondrial	0.0002	-	+/-	-	-	-/-
Q8NDA8	MROH1	MROH1	Maestro heat-like repeat-containing protein family member 1	-	-	-/-	0.0011	-	+/-
P49006	MRP	MARCKSL1	MARCKS-related protein	0.0012	-	+/-	-	-	-/-
P33527	MRP1	ABCC1	Multidrug resistance-associated protein 1	0.0001	-	+/-	-	-	-/-

Q92887	MRP2	ABCC2	Canalicular multispecific organic anion transporter 1	0.0006	0.0001	6.14	0.0036	0.0044	0.82
O15438	MRP3	ABCC3	Canalicular multispecific organic anion transporter 2	0.0007	0.0002	3.41	0.0054	0.0031	1.74
O95255	MRP6	ABCC6	Multidrug resistance-associated protein 6	0.0002	0.0001	1.66	0.0043	0.0045	0.97
Q9UKD2	MRT4	MRTO4	mRNA turnover protein 4 homolog	-	-	-/-	0.0049	-	+/-
Q96DH6	MSI2H	MSI2	RNA-binding protein Musashi homolog 2	0.0011	-	+/-	0.0060	-	+/-
Q9UJ68	MSRA	MSRA	Mitochondrial peptide methionine sulfoxide reductase	0.0141	0.0181	0.78	0.0140	0.0268	0.52
Q9Y3D2	MSRB2	MSRB2	Methionine-R-sulfoxide reductase B2, mitochondrial	0.0012	0.0026	0.45	-	-	-/-
P47224	MSS4	RABIF	Guanine nucleotide exchange factor MSS4	0.0102	0.0049	2.11	-	-	-/-
P13640	MT1G	MT1G	Metallothionein-1G	0.0013	-	+/-	-	-	-/-
P13640-2	MT1G	MT1G	Isoform 2 of Metallothionein-1G	0.0007	-	+/-	-	-	-/-
O94776	MTA2	MTA2	Metastasis-associated protein MTA2	0.0012	-	+/-	0.0043	-	+/-
Q86U44	MTA70	METTL3	N6-adenosine-methyltransferase catalytic subunit	-	0.0002	-/+	-	-	-/-
Q13126	MTAP	MTAP	S-methyl-5'-thioadenosine phosphorylase	0.0042	0.0053	0.79	0.0100	0.0143	0.70
P11137-4	MTAP2	MAP2	Isoform 4 of Microtubule-associated protein 2	0.0009	-	+/-	-	-	-/-
P11137	MTAP2	MAP2	Microtubule-associated protein 2	0.0000	-	+/-	-	-	-/-
Q9NZJ7	MTCH1	MTCH1	Mitochondrial carrier homolog 1	-	-	-/-	0.0053	0.0085	0.62
Q9Y6C9	MTCH2	MTCH2	Mitochondrial carrier homolog 2	0.0515	0.0659	0.78	0.0793	0.0828	0.96

Q9UDX5	MTFP1	MTFP1	Mitochondrial fission process protein 1	0.0026	0.0028	0.94	0.0116	0.0120	0.97
P49914	MTHFS	MTHFS	5-formyltetrahydrofolate cyclo-ligase	0.0053	0.0108	0.49	0.0066	0.0148	0.45
Q96S19	MTL26	METTL26	Methyltransferase-like 26	0.0010	0.0047	0.22	-	-	-/-
Q9NXD2	MTMRA	MTMR10	Myotubularin-related protein 10	-	-	-/-	-	0.0022	-/+
Q9BV20	MTNA	MRI1	Methylthioribose-1-phosphate isomerase	0.0054	0.0046	1.19	0.0058	0.0093	0.62
Q96GX9	MTNB	APIP	Methylthioribulose-1-phosphate dehydratase	0.0022	0.0023	0.95	0.0062	-	+/-
Q9BV57	MTND	ADI1	1,2-dihydroxy-3-keto-5-methylthiopentene dioxygenase	0.0251	0.0313	0.80	0.0294	0.0486	0.61
P42345	MTOR	MTOR	Serine/threonine-protein kinase mTOR	-	-	-/-	0.0017	0.0010	1.62
P55157	MTP	MTTP	Microsomal triglyceride transfer protein large subunit	0.0418	0.0619	0.68	0.0423	0.0751	0.56
P58546	MTPN	MTPN	Myotrophin	0.0320	0.0408	0.79	0.0254	0.0161	1.58
P42285	MTREX	MTREX	Exosome RNA helicase MTR4	0.0000	-	+/-	0.0036	0.0022	1.64
Q13505	MTX1	MTX1	Metaxin-1	0.0004	0.0002	1.74	0.0041	0.0035	1.19
O75431	MTX2	MTX2	Metaxin-2	0.0140	0.0094	1.49	0.0095	0.0080	1.19
P43121	MUC18	MCAM	Cell surface glycoprotein MUC18	0.0008	-	+/-	-	-	-/-
P22033	MUTA	MMUT	Methylmalonyl-CoA mutase, mitochondrial	0.0156	0.0389	0.40	0.0169	0.0417	0.40
P53602	MVD1	MVD	Diphosphomevalonate decarboxylase	0.0004	0.0055	0.06	-	0.0080	-/+
Q14764	MVP	MVP	Major vault protein	0.0581	0.0060	9.67	0.0407	0.0084	4.86
P20591	MX1	MX1	Interferon-induced GTP-binding protein Mx1	0.0001	-	+/-	-	-	-/-
Q92614	MY18A	MYO18A	Unconventional myosin-XVIIIa	0.0002	0.0006	0.27	0.0024	0.0016	1.56

Q96S97	MYADM	MYADM	Myeloid-associated differentiation marker	-	-	-/-	0.0186	-	+/-
Q99836	MYD88	MYD88	Myeloid differentiation primary response protein MyD88	0.0002	0.0011	0.22	-	-	-/-
Q969H8	MYDGF	MYDGF	Myeloid-derived growth factor	0.0819	0.0678	1.21	0.0411	0.0289	1.42
Q9P2K5	MYEF2	MYEF2	Myelin expression factor 2	0.0076	-	+/-	-	-	-/-
P02144	MYG	MB	Myoglobin	0.0016	-	+/-	-	-	-/-
Q9HB07	MYG1	C12orf10	UPF0160 protein MYG1, mitochondrial	0.0046	0.0060	0.76	0.0133	0.0078	1.71
P12882	MYH1	MYH1	Myosin-1	0.0003	-	+/-	-	-	-/-
P35580	MYH10	MYH10	Myosin-10	0.0079	0.0036	2.22	0.0191	0.0165	1.16
P35749	MYH11	MYH11	Myosin-11	0.0072	0.0015	4.90	0.0102	0.0085	1.19
Q7Z406	MYH14	MYH14	Myosin-14	0.0032	0.0021	1.52	0.0095	0.0004	24.48
Q7Z406-2	MYH14	MYH14	Isoform 2 of Myosin-14	-	-	-/-	0.0089	0.0078	1.14
Q9UKX2	MYH2	MYH2	Myosin-2	0.0004	-	+/-	-	-	-/-
P11055	MYH3	MYH3	Myosin-3	0.0001	-	+/-	-	-	-/-
Q9Y623	MYH4	MYH4	Myosin-4	0.0002	0.0001	2.46	-	-	-/-
P12883	MYH7	MYH7	Myosin-7	0.0001	-	+/-	-	-	-/-
P13535	MYH8	MYH8	Myosin-8	0.0002	-	+/-	-	-	-/-
P35579-2	MYH9	MYH9	Isoform 2 of Myosin-9	0.0020	-	+/-	-	-	-/-
P35579	MYH9	MYH9	Myosin-9	0.0564	0.0268	2.11	0.0839	0.0540	1.55
P05976	MYL1	MYL1	Myosin light chain 1/3, skeletal muscle isoform	0.0017	0.0018	0.98	-	-	-/-

Q9BUA6	MYL10	MYL10	Myosin regulatory light chain 10	0.0004	-	+/-	-	-	-/-
P60660-2	MYL6	MYL6	Isoform Smooth muscle of Myosin light polypeptide 6	0.1139	0.1058	1.08	-	-	-/-
P60660	MYL6	MYL6	Myosin light polypeptide 6	0.2604	0.2410	1.08	0.2570	0.1506	1.71
P14649	MYL6B	MYL6B	Myosin light chain 6B	0.0010	0.0015	0.63	-	-	-/-
P24844	MYL9	MYL9	Myosin regulatory light polypeptide 9	0.0741	0.0417	1.78	0.0646	-	+/-
Q15746	MYLK	MYLK	Myosin light chain kinase, smooth muscle	0.0011	0.0006	1.94	0.0040	0.0032	1.26
A0A1B0GTQ4	MYMX	MYMX	Protein myomixer	0.0013	-	+/-	-	-	-/-
O43795	MYO1B	MYO1B	Unconventional myosin-Ib	0.0057	0.0038	1.51	0.0018	0.0216	0.08
O43795-2	MYO1B	MYO1B	Isoform 2 of Unconventional myosin-Ib	-	-	-/-	0.0255	-	+/-
O00159-3	MYO1C	MYO1C	Isoform 3 of Unconventional myosin-Ic	0.0003	-	+/-	-	-	-/-
O00159	MYO1C	MYO1C	Unconventional myosin-Ic	0.0031	0.0036	0.86	0.0229	0.0170	1.35
O94832	MYO1D	MYO1D	Unconventional myosin-Id	0.0000	-	+/-	0.0059	0.0020	3.04
Q12965	MYO1E	MYO1E	Unconventional myosin-Ie	0.0002	-	+/-	0.0048	0.0014	3.53
O00160	MYO1F	MYO1F	Unconventional myosin-If	0.0007	-	+/-	0.0082	0.0020	4.02
B0I1T2	MYO1G	MYO1G	Unconventional myosin-Ig	0.0000	-	+/-	0.0022	-	+/-
Q8NEV4	MYO3A	MYO3A	Myosin-IIIa	0.0004	-	+/-	-	-	-/-
Q9Y4I1	MYO5A	MYO5A	Unconventional myosin-Va	-	-	-/-	0.0007	-	+/-
Q9ULV0	MYO5B	MYO5B	Unconventional myosin-Vb	0.0001	-	+/-	-	-	-/-
Q9UM54-6	MYO6	MYO6	Isoform 6 of Unconventional myosin-VI	0.0001	-	+/-	0.0024	-	+/-

Q9UM54	MYO6	MYO6	Unconventional myosin-VI	0.0006	-	+/-	0.0044	0.0010	4.53
Q9NZM1	MYOF	MYOF	Myoferlin	0.0008	-	+/-	0.0072	0.0012	6.22
Q5VU43	MYOME	PDE4DIP	Myomegalin	-	0.0000	-/+	-	-	-/-
P02689	MYP2	PMP2	Myelin P2 protein	0.0003	-	+/-	-	-	-/-
P60201	MYPR	PLP1	Myelin proteolipid protein	0.0004	-	+/-	-	-	-/-
O14974	MYPT1	PPP1R12A	Protein phosphatase 1 regulatory subunit 12A	0.0002	0.0002	1.43	-	-	-/-
Q8WU39	MZB1	MZB1	Marginal zone B- and B1-cell-specific protein	0.0054	0.0093	0.58	-	-	-/-
P41227	NAA10	NAA10	N-alpha-acetyltransferase 10	0.0020	0.0037	0.53	-	-	-/-
P61599	NAA20	NAA20	N-alpha-acetyltransferase 20	0.0006	0.0019	0.33	-	-	-/-
Q9GZZ1	NAA50	NAA50	N-alpha-acetyltransferase 50	0.0024	0.0047	0.50	0.0127	-	+/-
Q02083-2	NAAA	NAAA	Isoform 2 of N-acylethanolamine-hydrolyzing acid amidase	-	0.0004	-/+	-	-	-/-
Q02083	NAAA	NAAA	N-acylethanolamine-hydrolyzing acid amidase	0.0010	0.0010	1.03	0.0082	0.0085	0.97
E9PAV3	NACAM	NACA	Nascent polypeptide-associated complex subunit alpha, muscle-specific form	0.0056	0.0024	2.33	0.0017	0.0016	1.09
Q9BZK3	NACP4	NACA4P	Putative nascent polypeptide-associated complex subunit alpha-like protein	0.0039	0.0011	3.56	-	-	-/-
Q15274	NADC	QPRT	Nicotinate-nucleotide pyrophosphorylase [carboxylating]	0.0419	0.0301	1.39	0.0437	0.0599	0.73
Q6IA69	NADE	NADSYN1	Glutamine-dependent NAD(+) synthetase	-	-	-/-	-	0.0028	-/+

Q58DX5	NADL2	NAALADL2	Inactive N-acetylated-alpha-linked acidic dipeptidase-like protein 2	-	0.0003	-/+	-	-	-/-
Q9Y303	NAGA	AMDHD2	N-acetylglucosamine-6-phosphate deacetylase	-	0.0010	-/+	-	-	-/-
P17050	NAGAB	NAGA	Alpha-N-acetylgalactosaminidase	0.0055	0.0028	1.92	0.0138	-	+/-
Q9UJ70	NAGK	NAGK	N-acetyl-D-glucosamine kinase	0.0269	0.0193	1.39	0.0460	0.0260	1.77
Q8N159	NAGS	NAGS	N-acetylglutamate synthase, mitochondrial	0.0001	0.0002	0.50	-	0.0098	-/+
Q4G0N4	NAKD2	NADK2	NAD kinase 2, mitochondrial	0.0322	0.0714	0.45	0.0259	0.0483	0.54
Q86W24	NAL14	NLRP14	NACHT, LRR and PYD domains-containing protein 14	-	-	-/-	0.0016	-	+/-
Q8IZF0	NALCN	NALCN	Sodium leak channel non-selective protein	0.0008	-	+/-	-	-	-/-
Q9NX02	NALP2	NLRP2	NACHT, LRR and PYD domains-containing protein 2	0.0001	-	+/-	-	-	-/-
Q86W28	NALP8	NLRP8	NACHT, LRR and PYD domains-containing protein 8	0.0001	0.0001	0.62	-	-	-/-
P43490	NAMPT	NAMPT	Nicotinamide phosphoribosyltransferase	0.0220	0.0105	2.09	0.0684	0.0336	2.03
P49321	NASP	NASP	Nuclear autoantigenic sperm protein	0.0005	0.0016	0.33	-	-	-/-
Q9H0A0	NAT10	NAT10	RNA cytidine acetyltransferase	0.0001	-	+/-	0.0022	-	+/-
Q9UHE5	NAT8	NAT8	N-acetyltransferase 8	0.0003	-	+/-	0.0084	0.0211	0.40
Q9UHQ9	NB5R1	CYB5R1	NADH-cytochrome b5 reductase 1	0.0254	0.0177	1.44	0.0492	0.0187	2.64
Q6BCY4	NB5R2	CYB5R2	NADH-cytochrome b5 reductase 2	0.0005	0.0006	0.72	-	-	-/-
P00387	NB5R3	CYB5R3	NADH-cytochrome b5 reductase 3	0.0674	0.0590	1.14	0.0976	0.0708	1.38

A2RRP1	NBAS	NBAS	Neuroblastoma-amplified sequence	0.0003	-	+/-	0.0019	0.0008	2.45
O60934	NBN	NBN	Nibrin	0.0001	-	+/-	-	-	-/-
Q14596	NBR1	NBR1	Next to BRCA1 gene 1 protein	0.0011	-	+/-	-	-	-/-
P61601	NCALD	NCALD	Neurocalcin-delta	0.0062	0.0032	1.96	-	-	-/-
Q09161	NCBP1	NCBP1	Nuclear cap-binding protein subunit 1	-	-	-/-	0.0021	-	+/-
P52298	NCBP2	NCBP2	Nuclear cap-binding protein subunit 2	0.0009	0.0010	0.83	-	-	-/-
Q6PIU2	NCEH1	NCEH1	Neutral cholesterol ester hydrolase 1	0.0014	-	+/-	0.0186	0.0056	3.31
P14598	NCF1	NCF1	Neutrophil cytosol factor 1	0.0030	-	+/-	0.0054	-	+/-
P19878	NCF2	NCF2	Neutrophil cytosol factor 2	0.0011	-	+/-	0.0110	-	+/-
P19878-2	NCF2	NCF2	Isoform 2 of Neutrophil cytosol factor 2	0.0002	-	+/-	-	-	-/-
Q15080	NCF4	NCF4	Neutrophil cytosol factor 4	0.0019	-	+/-	0.0108	-	+/-
P16333	NCK1	NCK1	Cytoplasmic protein NCK1	0.0010	0.0006	1.77	-	-	-/-
O43639	NCK2	NCK2	Cytoplasmic protein NCK2	0.0001	-	+/-	-	-	-/-
Q9Y2A7	NCKP1	NCKAP1	Nck-associated protein 1	-	-	-/-	0.0042	0.0017	2.49
P55160	NCKPL	NCKAP1L	Nck-associated protein 1-like	0.0002	-	+/-	0.0025	-	+/-
Q969V3	NCLN	NCLN	Nicalin	0.0026	0.0006	4.36	0.0193	0.0096	2.01
Q9HCD5	NCOA5	NCOA5	Nuclear receptor coactivator 5	0.0001	-	+/-	0.0021	-	+/-
P16435	NCPR	POR	NADPH--cytochrome P450 reductase	0.0974	0.0646	1.51	0.0923	0.0978	0.94
P62166	NCS1	NCS1	Neuronal calcium sensor 1	0.0017	-	+/-	-	-	-/-
Q13232	NDK3	NME3	Nucleoside diphosphate kinase 3	0.0064	0.0064	1.01	0.0219	0.0161	1.37

O75414	NDK6	NME6	Nucleoside diphosphate kinase 6	0.0013	-	+/-	-	-	-/-
Q9Y5B8	NDK7	NME7	Nucleoside diphosphate kinase 7	-	0.0006	-/+	-	-	-/-
P15531-2	NDKA	NME1	Isoform 2 of Nucleoside diphosphate kinase A	0.0030	-	+/-	-	-	-/-
P15531	NDKA	NME1	Nucleoside diphosphate kinase A	0.1837	0.1467	1.25	0.1398	0.1092	1.28
P22392	NDKB	NME2	Nucleoside diphosphate kinase B	0.0522	0.0939	0.56	0.1428	0.1046	1.37
Q92597	NDRG1	NDRG1	Protein NDRG1	0.0267	0.0267	1.00	0.0116	0.0120	0.97
Q9UN36-2	NDRG2	NDRG2	Isoform 2 of Protein NDRG2	0.0007	0.0017	0.41	-	-	-/-
Q9UN36	NDRG2	NDRG2	Protein NDRG2	0.0580	0.1586	0.37	0.0262	0.0672	0.39
Q9UGV2	NDRG3	NDRG3	Protein NDRG3	0.0024	0.0023	1.03	-	-	-/-
O15239	NDUA1	NDUFA1	NADH dehydrogenase [ubiquinone] 1 alpha subcomplex subunit 1	-	0.0013	-/+	-	-	-/-
O43678	NDUA2	NDUFA2	NADH dehydrogenase [ubiquinone] 1 alpha subcomplex subunit 2	0.0015	0.0050	0.31	-	0.0138	-/+
O95167	NDUA3	NDUFA3	NADH dehydrogenase [ubiquinone] 1 alpha subcomplex subunit 3	0.0033	0.0045	0.73	-	-	-/-
O00483	NDUA4	NDUFA4	Cytochrome c oxidase subunit NDUFA4	0.0088	0.0141	0.62	0.0396	0.0634	0.62
Q16718	NDUA5	NDUFA5	NADH dehydrogenase [ubiquinone] 1 alpha subcomplex subunit 5	0.0096	0.0123	0.78	0.0143	0.0120	1.19
P56556	NDUA6	NDUFA6	NADH dehydrogenase [ubiquinone] 1 alpha subcomplex subunit 6	0.0059	0.0125	0.47	0.0088	0.0144	0.61

P51970	NDUA8	NDUFA8	NADH dehydrogenase [ubiquinone] 1 alpha subcomplex subunit 8	0.0064	0.0201	0.32	-	-	-/-
Q16795	NDUA9	NDUFA9	NADH dehydrogenase [ubiquinone] 1 alpha subcomplex subunit 9, mitochondrial	0.0089	0.0202	0.44	0.0142	0.0311	0.45
O95299	NDUAA	NDUFA10	NADH dehydrogenase [ubiquinone] 1 alpha subcomplex subunit 10, mitochondrial	0.0072	0.0137	0.52	0.0145	0.0246	0.59
Q86Y39	NDUAB	NDUFA11	NADH dehydrogenase [ubiquinone] 1 alpha subcomplex subunit 11	0.0064	0.0030	2.12	-	0.0321	-/+
Q9UI09	NDUAC	NDUFA12	NADH dehydrogenase [ubiquinone] 1 alpha subcomplex subunit 12	0.0096	0.0117	0.82	0.0164	0.0511	0.32
Q9P0J0	NDUAD	NDUFA13	NADH dehydrogenase [ubiquinone] 1 alpha subcomplex subunit 13	0.0245	0.0583	0.42	0.0134	0.0321	0.42
O75438	NDUB1	NDUFB1	NADH dehydrogenase [ubiquinone] 1 beta subcomplex subunit 1	-	0.0033	-/+	-	-	-/-
O43676	NDUB3	NDUFB3	NADH dehydrogenase [ubiquinone] 1 beta subcomplex subunit 3	0.0008	0.0056	0.15	-	-	-/-
O95168	NDUB4	NDUFB4	NADH dehydrogenase [ubiquinone] 1 beta subcomplex subunit 4	0.0010	0.0008	1.29	-	-	-/-
O43674	NDUB5	NDUFB5	NADH dehydrogenase [ubiquinone] 1 beta subcomplex subunit 5, mitochondrial	0.0018	0.0017	1.01	0.0074	0.0077	0.97
O95139	NDUB6	NDUFB6	NADH dehydrogenase [ubiquinone] 1 beta subcomplex subunit 6	0.0016	0.0015	1.08	-	-	-/-
P17568	NDUB7	NDUFB7	NADH dehydrogenase [ubiquinone] 1 beta subcomplex subunit 7	0.0004	0.0004	1.08	-	-	-/-

O95169	NDUB8	NDUFB8	NADH dehydrogenase [ubiquinone] 1 beta subcomplex subunit 8, mitochondrial	-	0.0005	-/+	0.0098	0.0161	0.61
Q9Y6M9	NDUB9	NDUFB9	NADH dehydrogenase [ubiquinone] 1 beta subcomplex subunit 9	0.0027	0.0048	0.57	-	-	-/-
O96000	NDUBA	NDUFB10	NADH dehydrogenase [ubiquinone] 1 beta subcomplex subunit 10	0.0106	0.0221	0.48	-	-	-/-
Q9NX14	NDUBB	NDUFB11	NADH dehydrogenase [ubiquinone] 1 beta subcomplex subunit 11, mitochondrial	0.0010	0.0015	0.65	0.0109	0.0113	0.97
O95298	NDUC2	NDUFC2	NADH dehydrogenase [ubiquinone] 1 subunit C2	0.0003	0.0038	0.08	-	-	-/-
E9PQ53	NDUCR	NDUFC2-KCTD14	NADH dehydrogenase [ubiquinone] 1 subunit C2, isoform 2	0.0009	-	+/-	-	-	-/-
Q8N183	NDUF2	NDUFAF2	NADH dehydrogenase [ubiquinone] 1 alpha subcomplex assembly factor 2	0.0026	0.0009	2.88	-	-	-/-
Q9BU61	NDUF3	NDUFAF3	NADH dehydrogenase [ubiquinone] 1 alpha subcomplex assembly factor 3	0.0163	0.0033	4.95	-	-	-/-
Q9P032	NDUF4	NDUFAF4	NADH dehydrogenase [ubiquinone] 1 alpha subcomplex assembly factor 4	0.0003	0.0013	0.25	-	-	-/-
Q5TEU4	NDUF5	NDUFAF5	Arginine-hydroxylase NDUFAF5, mitochondrial	0.0001	0.0006	0.23	-	-	-/-
Q330K2	NDUF6	NDUFAF6	NADH dehydrogenase (ubiquinone) complex I, assembly factor 6	0.0001	-	+/-	-	-	-/-
Q7L592	NDUF7	NDUFAF7	Protein arginine methyltransferase NDUFAF7, mitochondrial	0.0004	0.0002	1.70	0.0038	-	+/-

P28331	NDUS1	NDUFS1	NADH-ubiquinone oxidoreductase 75 kDa subunit, mitochondrial	0.0268	0.0218	1.23	0.0287	0.0396	0.73
O75306	NDUS2	NDUFS2	NADH dehydrogenase [ubiquinone] iron-sulfur protein 2, mitochondrial	0.0062	0.0118	0.53	0.0205	0.0220	0.93
O75489	NDUS3	NDUFS3	NADH dehydrogenase [ubiquinone] iron-sulfur protein 3, mitochondrial	0.0441	0.0384	1.15	0.0409	0.0500	0.82
O43181	NDUS4	NDUFS4	NADH dehydrogenase [ubiquinone] iron-sulfur protein 4, mitochondrial	0.0027	0.0015	1.80	-	-	-/-
O43920	NDUS5	NDUFS5	NADH dehydrogenase [ubiquinone] iron-sulfur protein 5	0.0008	0.0016	0.49	0.0155	0.0227	0.68
O75380	NDUS6	NDUFS6	NADH dehydrogenase [ubiquinone] iron-sulfur protein 6, mitochondrial	0.0034	0.0053	0.64	-	-	-/-
O75251	NDUS7	NDUFS7	NADH dehydrogenase [ubiquinone] iron-sulfur protein 7, mitochondrial	0.0060	0.0016	3.80	-	0.0017	-/+
O00217	NDUS8	NDUFS8	NADH dehydrogenase [ubiquinone] iron-sulfur protein 8, mitochondrial	0.0169	0.0238	0.71	-	0.0248	-/+
P49821	NDUV1	NDUFV1	NADH dehydrogenase [ubiquinone] flavoprotein 1, mitochondrial	0.0098	0.0110	0.89	0.0203	0.0464	0.44
P19404	NDUV2	NDUFV2	NADH dehydrogenase [ubiquinone] flavoprotein 2, mitochondrial	0.0084	0.0134	0.63	-	0.0020	-/+
P56181-2	NDUV3	NDUFV3	Isoform 2 of NADH dehydrogenase [ubiquinone] flavoprotein 3, mitochondrial	0.0001	-	+/-	-	-	-/-
P56181	NDUV3	NDUFV3	NADH dehydrogenase [ubiquinone] flavoprotein 3, mitochondrial	-	0.0007	-/+	-	-	-/-

Q96SB3	NEB2	PPP1R9B	Neurabin-2	0.0006	0.0003	2.23	-	-	-/-
Q8NC96	NECP1	NECAP1	Adaptin ear-binding coat-associated protein 1	0.0011	-	+/-	-	-	-/-
Q9NVZ3	NECP2	NECAP2	Adaptin ear-binding coat-associated protein 2	0.0037	0.0039	0.95	0.0074	-	+/-
Q92692	NECT2	NECTIN2	Nectin-2	0.0026	0.0003	8.23	-	-	-/-
P46934-4	NEDD4	NEDD4	Isoform 4 of E3 ubiquitin-protein ligase NEDD4	-	-	-/-	0.0038	0.0029	1.30
Q15843	NEDD8	NEDD8	NEDD8	0.0035	0.0083	0.43	0.0143	-	+/-
Q8TDX7	NEK7	NEK7	Serine/threonine-protein kinase Nek7	0.0012	0.0008	1.57	-	-	-/-
Q8TD19	NEK9	NEK9	Serine/threonine-protein kinase Nek9	0.0001	-	+/-	-	0.0021	-/+
Q8WX92	NELFB	NELFB	Negative elongation factor B	-	-	-/-	0.0028	-	+/-
Q9UMX5	NENF	NENF	Neudesin	0.0035	0.0064	0.54	-	-	-/-
Q92979	NEP1	EMG1	Ribosomal RNA small subunit methyltransferase NEP1	0.0002	-	+/-	-	-	-/-
P48681	NEST	NES	Nestin	0.0007	0.0005	1.46	0.0012	-	+/-
Q8NFW8	NEUA	CMAS	N-acylneuraminate cytidylyltransferase	0.0031	0.0033	0.92	0.0130	0.0072	1.80
Q9BYT8	NEUL	NLN	Neurolysin, mitochondrial	0.0016	0.0017	0.93	0.0164	0.0097	1.68
Q99519	NEUR1	NEU1	Sialidase-1	0.0076	0.0013	5.96	0.0150	-	+/-
Q0ZGT2	NEXN	NEXN	Nexilin	0.0002	0.0006	0.27	-	-	-/-
Q12857	NFIA	NFIA	Nuclear factor 1 A-type	-	-	-/-	-	0.0041	-/+
P19838	NFKB1	NFKB1	Nuclear factor NF-kappa-B p105 subunit	0.0003	0.0001	2.94	0.0032	-	+/-
Q00653	NFKB2	NFKB2	Nuclear factor NF-kappa-B p100 subunit	0.0002	-	+/-	0.0021	-	+/-

P07196	NFL	NEFL	Neurofilament light polypeptide	-	0.0004	-/+	-	-	-/-
P07197	NFM	NEFM	Neurofilament medium polypeptide	-	0.0001	-/+	-	-	-/-
Q9Y697	NFS1	NFS1	Cysteine desulfurase, mitochondrial	0.0009	0.0018	0.52	0.0036	-	+/-
Q9UMS0	NFU1	NFU1	NFU1 iron-sulfur cluster scaffold homolog, mitochondrial	0.0025	0.0050	0.50	-	-	-/-
P25208	NFYB	NFYB	Nuclear transcription factor Y subunit beta	0.0003	-	+/-	-	-	-/-
Q13952	NFYC	NFYC	Nuclear transcription factor Y subunit gamma	0.0012	-	+/-	-	-	-/-
P80188	NGAL	LCN2	Neutrophil gelatinase-associated lipocalin	0.0653	0.0048	13.56	0.1230	0.0107	11.49
Q8N5V2	NGEF	NGEF	Ephexin-1	-	-	-/-	0.0019	-	+/-
P55769	NH2L1	SNU13	NHP2-like protein 1	0.0090	0.0124	0.73	0.0265	0.0350	0.76
Q8NBF2	NHLC2	NHLRC2	NHL repeat-containing protein 2	-	-	-/-	-	0.0030	-/+
Q5JS37	NHLC3	NHLRC3	NHL repeat-containing protein 3	0.0014	0.0007	1.97	0.0066	0.0068	0.97
Q9NX24	NHP2	NHP2	H/ACA ribonucleoprotein complex subunit 2	0.0012	0.0015	0.76	-	-	-/-
O14745	NHRF1	SLC9A3R1	Na (+)/H (+) exchange regulatory cofactor NHE-RF1	0.0234	0.0146	1.60	0.0089	0.0096	0.93
Q15599-2	NHRF2	SLC9A3R2	Isoform 2 of Na(+)/H(+) exchange regulatory cofactor NHE-RF2	0.0019	-	+/-	-	-	-/-
Q15599	NHRF2	SLC9A3R2	Na (+)/H (+) exchange regulatory cofactor NHE-RF1	0.0056	0.0047	1.18	0.0084	0.0064	1.32
Q5T2W1	NHRF3	PDZK1	Na (+)/H (+) exchange regulatory cofactor NHE-RF1	0.0030	0.0140	0.21	-	0.0081	-/+
Q6T4R5	NHS	NHS	Nance-Horan syndrome protein	0.0000	-	+/-	-	-	-/-

Q9BZQ8	NIBA1	NIBAN1	Protein Niban 1	0.0001	-	+/-	0.0033	-	+/-
Q96TA1	NIBA2	NIBAN2	Protein Niban 2	0.0005	0.0004	1.13	0.0048	-	+/-
Q92542	NICA	NCSTN	Nicastrin	0.0045	-	+/-	0.0088	0.0034	2.57
P14543	NID1	NID1	Nidogen-1	0.0168	0.0044	3.79	0.0159	0.0089	1.78
Q14112	NID2	NID2	Nidogen-2	0.0114	0.0021	5.46	0.0051	-	+/-
Q9GZT8	NIF3L	NIF3L1	NIF3-like protein 1	0.0023	0.0047	0.50	0.0056	0.0090	0.62
Q9Y221	NIP7	NIP7	60S ribosome subunit biogenesis protein NIP7 homolog	0.0005	-	+/-	-	-	-/-
Q6KC79	NIPBL	NIPBL	Nipped-B-like protein	0.0000	-	+/-	-	-	-/-
Q9BPW8	NIPS1	NIPSNAP1	Protein NipSnap homolog 1	0.0327	0.0566	0.58	0.0836	0.1143	0.73
O75323	NIPS2	NIPSNAP2	Protein NipSnap homolog 2	0.0004	0.0013	0.33	0.0103	-	+/-
Q9Y2I1	NISCH	NISCH	Nischarin	-	0.0001	-/+	-	-	-/-
Q86X76	NIT1	NIT1	Deaminated glutathione amidase	0.0219	0.0135	1.62	0.0312	0.0366	0.85
Q9NQR4	NIT2	NIT2	Omega-amidase NIT2	0.0496	0.0674	0.74	0.0648	0.1300	0.50
O15226	NKRF	NKRF	NF-kappa-B-repressing factor	0.0001	-	+/-	-	-	-/-
Q9NPP4	NLRC4	NLRC4	NLR family CARD domain-containing protein 4	-	0.0001	-/+	-	-	-/-
P22307-3	NLTP	SCP2	Isoform 3 of Non-specific lipid-transfer protein	0.0002	0.0050	0.04	-	-	-/-
P22307-2	NLTP	SCP2	Isoform SCP2 of Non-specific lipid-transfer protein	0.0005	0.0007	0.65	-	-	-/-

P22307	NLTP	SCP2	Non-specific lipid-transfer protein	0.0616	0.1075	0.57	0.0910	0.1421	0.64
Q96D46	NMD3	NMD3	60S ribosomal export protein NMD3	0.0001	-	+/-	-	-	-/-
Q13287	NMI	NMI	N-myc-interactor	0.0017	0.0033	0.52	0.0073	-	+/-
Q9HAN9	NMNA1	NMNAT1	Nicotinamide/nicotinic acid mononucleotide adenylyltransferase 1	0.0001	0.0009	0.14	-	0.0048	-/+
Q96T66	NMNA3	NMNAT3	Nicotinamide/nicotinic acid mononucleotide adenylyltransferase 3	0.0002	0.0007	0.25	-	-	-/-
Q9HBL8	NMRL1	NMRAL1	NmrA-like family domain-containing protein 1	0.0013	0.0019	0.67	-	-	-/-
P30419	NMT1	NMT1	Glycylpeptide N-tetradecanoyltransferase 1	0.0006	-	+/-	0.0143	0.0087	1.64
O60551	NMT2	NMT2	Glycylpeptide N-tetradecanoyltransferase 2	-	-	-/-	0.0114	0.0031	3.71
P40261	NNMT	NNMT	Nicotinamide N-methyltransferase	0.0420	0.0430	0.98	0.0476	0.0600	0.79
Q8IW45	NNRD	NAXD	ATP-dependent (S)-NAD(P)H-hydrate dehydratase	0.0056	0.0096	0.58	-	0.0064	-/+
Q8NCW5	NNRE	NAXE	NAD(P)H-hydrate epimerase	0.0097	0.0093	1.05	0.0266	0.0219	1.21
Q13423	NNTM	NNT	NAD(P) transhydrogenase, mitochondrial	0.0716	0.0502	1.43	0.1001	0.1113	0.90
Q9BZE4	NOG1	GTPBP4	Nucleolar GTP-binding protein 1	0.0007	-	+/-	0.0016	-	+/-
Q14978	NOLC1	NOLC1	Nucleolar and coiled-body phosphoprotein 1	-	-	-/-	-	0.0004	-/+
Q15155	NOMO1	NOMO1	Nodal modulator 1	0.0115	0.0027	4.22	-	-	-/-
Q5JPE7	NOMO2	NOMO2	Nodal modulator 2	0.0032	0.0011	2.88	0.0170	-	+/-
P69849	NOMO3	NOMO3	Nodal modulator 3	0.0021	0.0002	9.11	0.0214	0.0110	1.94

Q15233	NONO	NONO	Non-POU domain-containing octamer-binding protein	0.0134	0.0121	1.11	0.0239	0.0184	1.30
O00567	NOP56	NOP56	Nucleolar protein 56	0.0041	0.0006	6.71	0.0091	0.0054	1.69
Q9Y2X3	NOP58	NOP58	Nucleolar protein 58	0.0029	0.0010	2.95	0.0093	0.0063	1.47
Q9Y314	NOSIP	NOSIP	Nitric oxide synthase-interacting protein	0.0005	0.0004	1.45	-	-	-/-
P55209	NP1L1	NAP1L1	Nucleosome assembly protein 1-like 1	0.0055	0.0052	1.06	0.0009	0.0061	0.15
Q9ULW6	NP1L2	NAP1L2	Nucleosome assembly protein 1-like 2	-	0.0003	-/+	-	-	-/-
Q99733	NP1L4	NAP1L4	Nucleosome assembly protein 1-like 4	0.0047	0.0073	0.65	0.0041	0.0065	0.63
Q14207	NPAT	NPAT	Protein NPAT	0.0000	0.0001	0.67	-	-	-/-
P61916	NPC2	NPC2	NPC intracellular cholesterol transporter 2	0.0183	0.0130	1.41	0.0254	0.0263	0.97
Q9BXD5	NPL	NPL	N-acetylneuraminate lyase	0.0013	0.0015	0.86	0.0100	-	+/-
Q8TAT6	NPL4	NPLOC4	Nuclear protein localization protein 4 homolog	0.0012	0.0013	0.92	0.0145	0.0084	1.72
P06748	NPM	NPM1	Nucleophosmin	0.0577	0.0433	1.33	0.0284	0.0245	1.16
O75607	NPM3	NPM3	Nucleoplasmin-3	-	0.0010	-/+	-	-	-/-
Q6UXI9	NPNT	NPNT	Nephronectin	-	-	-/-	0.0050	0.0033	1.48
Q8WTW4	NPRL2	NPRL2	GATOR complex protein NPRL2	0.0076	-	+/-	-	-	-/-
Q9UFN0	NPS3A	NIPSNAP3A	Protein NipSnap homolog 3A	0.0147	0.0336	0.44	0.0371	0.0526	0.71
Q9Y639	NPTN	NPTN	Neuroplastin	0.0004	-	+/-	0.0044	-	+/-
P15559	NQO1	NQO1	NAD(P)H dehydrogenase [quinone] 1	0.0197	-	+/-	0.0699	-	+/-

P16083	NQO2	NQO2	Ribosyldihydronicotinamide dehydrogenase [quinone]	0.0243	0.0444	0.55	0.0489	0.0750	0.65
Q86WQ0	NR2CA	NR2C2AP	Nuclear receptor 2C2-associated protein	0.0009	0.0008	1.17	-	-	-/-
Q86VF7	NRAP	NRAP	Nebulin-related-anchoring protein	0.0000	-	+/-	-	-	-/-
Q96F24	NRBF2	NRBF2	Nuclear receptor-binding factor 2	0.0001	-	+/-	-	-	-/-
Q9UHY1	NRBP	NRBP1	Nuclear receptor-binding protein	-	0.0006	-/+	-	-	-/-
O43847	NRDC	NRDC	Nardilysin	0.0001	-	+/-	0.0025	0.0017	1.47
Q9H7Z3	NRDE2	NRDE2	Nuclear exosome regulator NRDE2	0.0001	-	+/-	-	-	-/-
Q9BQI9	NRIP2	NRIP2	Nuclear receptor-interacting protein 2	0.0002	-	+/-	-	-	-/-
Q7Z2Y5	NRK	NRK	Nik-related protein kinase	-	0.0001	-/+	-	-	-/-
Q9NWW6	NRK1	NMRK1	Nicotinamide riboside kinase 1	0.0013	0.0078	0.17	-	-	-/-
O14786	NRP1	NRP1	Neuropilin-1	0.0012	-	+/-	0.0044	-	+/-
Q15738	NSDHL	NSDHL	Sterol-4-alpha-carboxylate 3-dehydrogenase, decarboxylating	0.0019	0.0045	0.42	0.0257	0.0225	1.14
P46459	NSF	NSF	Vesicle-fusing ATPase	0.0030	0.0018	1.64	0.0199	0.0080	2.49
Q9UNZ2	NSF1C	NSFL1C	NSFL1 cofactor p47	0.0217	0.0220	0.98	0.0103	0.0050	2.05
Q08J23	NSUN2	NSUN2	RNA cytosine C(5)-methyltransferase NSUN2	-	-	-/-	0.0051	0.0042	1.22
Q8TEA1	NSUN6	NSUN6	Putative methyltransferase NSUN6	-	0.0003	-/+	-	-	-/-
Q8TCD5	NT5C	NT5C	5'(3')-deoxyribonucleotidase, cytosolic type	0.0049	0.0101	0.49	-	0.0015	-/+
Q5TFE4	NT5D1	NT5DC1	5'-nucleotidase domain-containing protein 1	0.0001	0.0017	0.08	0.0041	0.0114	0.36

Q96AB6	NTAN1	NTAN1	Protein N-terminal asparagine amidohydrolase	0.0023	0.0005	4.24	-	-	-/-
P61970	NTF2	NUTF2	Nuclear transport factor 2	0.0694	0.1775	0.39	0.0547	0.0565	0.97
Q9BV86	NTM1A	NTMT1	N-terminal Xaa-Pro-Lys N-methyltransferase 1	-	0.0004	-/+	-	-	-/-
Q9BSD7	NTPCR	NTPCR	Cancer-related nucleoside-triphosphatase	0.0049	0.0096	0.51	0.0294	0.0083	3.53
Q8WUM0	NU133	NUP133	Nuclear pore complex protein Nup133	0.0005	-	+/-	0.0027	-	+/-
O75694	NU155	NUP155	Nuclear pore complex protein Nup155	0.0004	-	+/-	0.0041	-	+/-
Q12769	NU160	NUP160	Nuclear pore complex protein Nup160	-	-	-/-	0.0032	0.0016	1.97
Q92621	NU205	NUP205	Nuclear pore complex protein Nup205	0.0001	-	+/-	0.0029	-	+/-
P35658	NU214	NUP214	Nuclear pore complex protein Nup214	0.0001	-	+/-	-	-	-/-
P03905	NU4M	MT-ND4	NADH-ubiquinone oxidoreductase chain 4	0.0004	-	+/-	-	-	-/-
P03915	NU5M	MT-ND5	NADH-ubiquinone oxidoreductase chain 5	-	0.0007	-/+	0.0103	0.0107	0.97
Q9Y5A7	NUB1	NUB1	NEDD8 ultimate buster 1	0.0001	-	+/-	-	-	-/-
P53384	NUBP1	NUBP1	Cytosolic Fe-S cluster assembly factor NUBP1	0.0013	0.0052	0.25	-	-	-/-
Q9Y5Y2	NUBP2	NUBP2	Cytosolic Fe-S cluster assembly factor NUBP2	0.0012	0.0073	0.17	-	0.0203	-/+
Q8TB37	NUBPL	NUBPL	Iron-sulfur protein NUBPL	0.0019	0.0023	0.84	-	-	-/-
Q02818	NUCB1	NUCB1	Nucleobindin-1	0.0012	0.0023	0.49	0.0028	-	+/-
P80303	NUCB2	NUCB2	Nucleobindin-2	0.0003	0.0007	0.44	-	-	-/-

Q14249	NUCG	ENDOG	Endonuclease G, mitochondrial	0.0005	0.0031	0.17	-	-	-/-
Q9H1E3	NUCKS	NUCKS1	Nuclear ubiquitous casein and cyclin-dependent kinase substrate 1	0.0005	0.0002	2.60	-	-	-/-
P19338	NUCL	NCL	Nucleolin	0.0561	0.0188	2.98	0.0195	0.0162	1.21
O95848	NUD14	NUDT14	Uridine diphosphate glucose pyrophosphatase NUDT14	0.0007	0.0015	0.50	-	-	-/-
Q9NV35	NUD15	NUDT15	Nucleotide triphosphate diphosphatase NUDT15	0.0011	0.0015	0.76	-	-	-/-
Q96DE0	NUD16	NUDT16	U8 snoRNA-decapping enzyme	0.0165	0.0159	1.03	0.0196	0.0203	0.97
A8MXV4	NUD19	NUDT19	Nucleoside diphosphate-linked moiety X motif 19	0.0003	0.0004	0.72	-	-	-/-
Q9Y266	NUDC	NUDC	Nuclear migration protein nudC	0.0191	0.0082	2.32	0.0103	0.0056	1.83
Q8WVJ2	NUDC2	NUDCD2	NudC domain-containing protein 2	0.0071	0.0088	0.81	-	-	-/-
Q8IVD9	NUDC3	NUDCD3	NudC domain-containing protein 3	-	0.0012	-/+	-	-	-/-
O95989	NUDT3	NUDT3	Diphosphoinositol polyphosphate phosphohydrolase 1	0.0045	0.0086	0.52	-	-	-/-
Q9NZJ9	NUDT4	NUDT4	Diphosphoinositol polyphosphate phosphohydrolase 2	0.0011	0.0008	1.39	-	-	-/-
Q9UKK9	NUDT5	NUDT5	ADP-sugar pyrophosphatase	0.0406	0.0588	0.69	0.0164	0.0241	0.68
P53370	NUDT6	NUDT6	Nucleoside diphosphate-linked moiety X motif 6	0.0011	0.0040	0.29	-	-	-/-
Q8WV74	NUDT8	NUDT8	Nucleoside diphosphate-linked moiety X motif 8	0.0021	0.0053	0.40	-	-	-/-

Q9BW91	NUDT9	NUDT9	ADP-ribose pyrophosphatase, mitochondrial	0.0015	0.0010	1.40	-	-	-/-
Q14980	NUMA1	NUMA1	Nuclear mitotic apparatus protein 1	0.0006	-	+/-	0.0006	0.0006	0.97
P49757	NUMB	NUMB	Protein numb homolog	0.0001	-	+/-	0.0041	-	+/-
Q8NFH5	NUP35	NUP35	Nucleoporin NUP35	0.0015	0.0011	1.43	-	-	-/-
Q8NFH4	NUP37	NUP37	Nucleoporin Nup37	0.0041	0.0014	2.95	0.0077	-	+/-
Q8NFH3	NUP43	NUP43	Nucleoporin Nup43	0.0053	0.0018	2.88	-	-	-/-
Q9UKX7	NUP50	NUP50	Nuclear pore complex protein Nup50	0.0002	-	+/-	-	-	-/-
Q7Z3B4	NUP54	NUP54	Nucleoporin p54	0.0016	0.0002	7.60	-	-	-/-
Q7Z3B4-3	NUP54	NUP54	Isoform 3 of Nucleoporin p54	-	-	-/-	0.0041	-	+/-
P37198	NUP62	NUP62	Nuclear pore glycoprotein p62	0.0029	0.0005	6.45	-	-	-/-
Q9BW27	NUP85	NUP85	Nuclear pore complex protein Nup85	-	-	-/-	0.0043	-	+/-
Q99567	NUP88	NUP88	Nuclear pore complex protein Nup88	0.0003	-	+/-	-	-	-/-
Q8N1F7	NUP93	NUP93	Nuclear pore complex protein Nup93	0.0019	-	+/-	0.0100	0.0032	3.13
P52948	NUP98	NUP98	Nuclear pore complex protein Nup98-Nup96	0.0002	-	+/-	0.0020	0.0013	1.46
Q6DKJ4	NXN	NXN	Nucleoredoxin	-	0.0007	-/+	-	-	-/-
Q8IWE2	NXP20	FAM114A1	Protein NOXP20	0.0017	0.0073	0.23	-	-	-/-
Q9Y530	OARD1	OARD1	ADP-ribose glycohydrolase OARD1	0.0006	0.0064	0.09	0.0093	0.0096	0.97
Q9Y6K5	OAS3	OAS3	2'-5'-oligoadenylate synthase 3	-	-	-/-	0.0036	-	+/-
P04181	OAT	OAT	Ornithine aminotransferase, mitochondrial	0.0014	0.0037	0.37	-	0.0042	-/+
Q9NX40	OCAD1	OCIAD1	OCIA domain-containing protein 1	0.0050	0.0104	0.47	0.0087	0.0185	0.47

Q56VL3	OCAD2	OCIAD2	OCIA domain-containing protein 2	0.0079	0.0123	0.64	0.0103	0.0241	0.43
Q16625	OCLN	OCLN	Occludin	-	0.0002	-/+	-	-	-/-
Q9UKG9	OCTC	CROT	Peroxisomal carnitine O-octanoyltransferase	0.0002	-	+/-	0.0174	0.0028	6.20
P11182	ODB2	DBT	Lipoamide acyltransferase component of branched-chain alpha-keto acid dehydrogenase complex, mitochondrial	0.0085	0.0189	0.45	0.0169	0.0442	0.38
P12694	ODBA	BCKDHA	2-oxoisovalerate dehydrogenase subunit alpha, mitochondrial	0.0091	0.0251	0.36	0.0059	0.0347	0.17
P21953	ODBB	BCKDHB	2-oxoisovalerate dehydrogenase subunit beta, mitochondrial	0.0015	0.0078	0.19	0.0051	0.0402	0.13
Q02218	ODO1	OGDH	2-oxoglutarate dehydrogenase, mitochondrial	0.0045	0.0063	0.71	0.0120	0.0196	0.61
P36957	ODO2	DLST	Dihydrolipoyllysine-residue succinyltransferase component of 2-oxoglutarate dehydrogenase complex, mitochondrial	0.0430	0.0233	1.84	0.0269	0.0226	1.19
P10515	ODP2	DLAT	Dihydrolipoyllysine-residue acetyltransferase component of pyruvate dehydrogenase complex, mitochondrial	0.0136	0.0064	2.12	0.0085	0.0189	0.45
P08559	ODPA	PDHA1	Pyruvate dehydrogenase E1 component subunit alpha, somatic form, mitochondrial	0.0288	0.0172	1.68	0.0092	0.0117	0.79
P11177	ODPB	PDHB	Pyruvate dehydrogenase E1 component subunit beta, mitochondrial	0.0513	0.0384	1.34	0.0270	0.0484	0.56
O00330	ODPX	PDHX	Pyruvate dehydrogenase protein X component, mitochondrial	0.0022	0.0019	1.16	-	-	-/-

Q5SWX8	ODR4	ODR4	Protein odr-4 homolog	-	-	-/-	0.0106	0.0062	1.69
Q9H488	OFUT1	POFUT1	GDP-fucose protein O-fucosyltransferase 1	0.0057	0.0059	0.96	0.0225	0.0233	0.97
Q9Y2G5	OFUT2	POFUT2	GDP-fucose protein O-fucosyltransferase 2	-	0.0002	-/+	-	-	-/-
O60502	OGA	OGA	Protein O-GlcNAcase	-	-	-/-	0.0021	0.0022	0.97
Q9ULD0	OGDHL	OGDHL	2-oxoglutarate dehydrogenase-like, mitochondrial	0.0009	0.0033	0.27	0.0073	0.0140	0.52
Q6N063	OGFD2	OGFOD2	2-oxoglutarate and iron-dependent oxygenase domain-containing protein 2	-	0.0003	-/+	-	-	-/-
Q6PK18	OGFD3	OGFOD3	2-oxoglutarate and iron-dependent oxygenase domain-containing protein 3	0.0010	-	+/-	-	-	-/-
Q9NZT2	OGFR	OGFR	Opioid growth factor receptor	-	0.0002	-/+	0.0056	-	+/-
Q8WWZ8	OIT3	OIT3	Oncoprotein-induced transcript 3 protein	-	0.0007	-/+	-	-	-/-
Q9NTK5-2	OLA1	OLA1	Isoform 2 of Obg-like ATPase 1	0.0008	-	+/-	-	-	-/-
Q9NTK5	OLA1	OLA1	Obg-like ATPase 1	0.0111	0.0056	1.99	0.0121	0.0059	2.07
Q6UWY5	OLFL1	OLFML1	Olfactomedin-like protein 1	0.0009	-	+/-	0.0044	0.0045	0.97
Q9NRN5	OLFL3	OLFML3	Olfactomedin-like protein 3	0.0003	-	+/-	-	-	-/-
Q6UX06	OLFM4	OLFM4	Olfactomedin-4	0.0117	-	+/-	0.0165	-	+/-
Q96E52	OMA1	OMA1	Metalloendopeptidase OMA1, mitochondrial	-	-	-/-	0.0047	-	+/-
O60313-10	OPA1	OPA1	Isoform 4 of Dynamin-like 120 kDa protein, mitochondrial	0.0012	-	+/-	-	-	-/-
O60313	OPA1	OPA1	Dynamin-like 120 kDa protein, mitochondrial	0.0013	0.0016	0.80	0.0033	0.0055	0.59

Q9H6K4	OPA3	OPA3	Optic atrophy 3 protein	0.0005	-	+/-	-	-	-/-
O14841	OPLA	OPLAH	5-oxoprolinase	0.0057	0.0047	1.21	0.0247	0.0227	1.09
Q96CV9	OPTN	OPTN	Optineurin	0.0005	0.0015	0.34	-	-	-/-
Q6IF42	OR2A2	OR2A2	Olfactory receptor 2A2	0.0118	-	+/-	-	-	-/-
Q8NH73	OR4S2	OR4S2	Olfactory receptor 4S2	-	0.0005	-/+	-	-	-/-
Q9Y3B8	ORN	REXO2	Oligoribonuclease, mitochondrial	0.0011	0.0008	1.45	-	-	-/-
Q9Y619	ORNT1	SLC25A15	Mitochondrial ornithine transporter 1	0.0052	0.0132	0.39	0.0179	0.0318	0.56
Q13438	OS9	OS9	Protein OS-9	0.0089	0.0108	0.82	0.0007	-	+/-
Q13438-2	OS9	OS9	Isoform 2 of Protein OS-9	-	0.0027	-/+	-	-	-/-
Q9H4L5	OSBL3	OSBPL3	Oxysterol-binding protein-related protein 3	0.0001	-	+/-	-	-	-/-
Q9BZF1	OSBL8	OSBPL8	Oxysterol-binding protein-related protein 8	0.0001	-	+/-	0.0042	0.0017	2.49
P22059	OSBP1	OSBP	Oxysterol-binding protein 1	0.0019	0.0005	3.96	0.0138	0.0082	1.67
Q9NPF4	OSGEP	OSGEP	Probable tRNA N6-adenosine threonylcarbamoyltransferase	0.0016	0.0029	0.56	0.0062	-	+/-
P39656	OST48	DDOST	Dolichyl-diphosphooligosaccharide--protein glycosyltransferase 48 kDa subunit	0.0734	0.0459	1.60	0.0446	0.0366	1.22
Q92882	OSTF1	OSTF1	Osteoclast-stimulating factor 1	0.0172	0.0069	2.48	0.0186	-	+/-
P00480	OTC	OTC	Ornithine carbamoyltransferase, mitochondrial	0.0794	0.1819	0.44	0.0830	0.1493	0.56
Q8N6M0	OTU6B	OTUD6B	Deubiquitinase OTUD6B	0.0003	-	+/-	-	-	-/-
Q96FW1	OTUB1	OTUB1	Ubiquitin thioesterase OTUB1	0.0308	0.0207	1.49	0.0280	0.0289	0.97

Q8WZ82	OVCA2	OVCA2	Esterase OVCA2	0.0004	0.0023	0.19	-	-	-/-
P14920	OXDA	DAO	D-amino-acid oxidase	0.0022	0.0071	0.32	0.0060	0.0308	0.19
Q99489	OXDD	DDO	D-aspartate oxidase	0.0002	0.0008	0.20	-	-	-/-
Q96RQ9	OXLA	IL4I1	L-amino-acid oxidase	0.0010	-	+/-	0.0032	-	+/-
Q96HP4	OXND1	OXNAD1	Oxidoreductase NAD-binding domain-containing protein 1	-	0.0010	-/+	-	-	-/-
Q8N573	OXR1	OXR1	Oxidation resistance protein 1	0.0004	0.0001	3.31	-	-	-/-
Q9NWU1	OXSM	OXSM	3-oxoacyl-[acyl-carrier-protein] synthase, mitochondrial	0.0003	0.0027	0.11	0.0069	0.0255	0.27
O95747	OXSR1	OXSR1	Serine/threonine-protein kinase OSR1	0.0010	0.0008	1.16	0.0029	0.0046	0.63
O75747	P3C2G	PIK3C2G	Phosphatidylinositol 4-phosphate 3-kinase C2 domain-containing subunit gamma	0.0000	-	+/-	-	-	-/-
Q32P28	P3H1	P3H1	Prolyl 3-hydroxylase 1	0.0048	0.0022	2.14	0.0026	-	+/-
P13674-2	P4HA1	P4HA1	Isoform 2 of Prolyl 4-hydroxylase subunit alpha-1	0.0005	-	+/-	-	-	-/-
P13674	P4HA1	P4HA1	Prolyl 4-hydroxylase subunit alpha-1	0.0075	0.0016	4.54	0.0124	0.0060	2.07
O15460	P4HA2	P4HA2	Prolyl 4-hydroxylase subunit alpha-2	0.0009	-	+/-	-	-	-/-
Q9BTU6	P4K2A	PI4K2A	Phosphatidylinositol 4-kinase type 2-alpha	-	-	-/-	0.0032	-	+/-
P32322	P5CR1	PYCR1	Pyrroline-5-carboxylate reductase 1, mitochondrial	0.0140	-	+/-	0.0033	-	+/-
Q96C36	P5CR2	PYCR2	Pyrroline-5-carboxylate reductase 2	0.0023	-	+/-	-	-	-/-
Q53H96	P5CR3	PYCR3	Pyrroline-5-carboxylate reductase 3	0.0083	0.0060	1.39	-	-	-/-

P54886	P5CS	ALDH18A1	Delta-1-pyrroline-5-carboxylate synthase	0.0059	0.0009	6.23	0.0189	0.0060	3.16
P27986	P85A	PIK3R1	Phosphatidylinositol 3-kinase regulatory subunit alpha	0.0002	-	+/-	-	-	-/-
P68402	PA1B2	PAFAH1B2	Platelet-activating factor acetylhydrolase IB subunit beta	0.0115	0.0086	1.34	-	-	-/-
Q15102	PA1B3	PAFAH1B3	Platelet-activating factor acetylhydrolase IB subunit gamma	0.0032	0.0071	0.45	-	-	-/-
Q9UQ80	PA2G4	PA2G4	Proliferation-associated protein 2G4	0.0192	0.0104	1.85	0.0281	0.0164	1.71
P14555	PA2GA	PLA2G2A	Phospholipase A2, membrane associated	-	-	-/-	0.0081	-	+/-
Q9BRP4	PAAF1	PAAF1	Proteasomal ATPase-associated factor 1	0.0001	0.0004	0.32	-	-	-/-
P11940	PABP1	PABPC1	Polyadenylate-binding protein 1	0.0333	0.0255	1.31	0.0318	0.0267	1.19
Q86U42	PABP2	PABPN1	Polyadenylate-binding protein 2	0.0058	0.0026	2.22	0.0054	0.0056	0.97
Q13310-3	PABP4	PABPC4	Isoform 3 of Polyadenylate-binding protein 4	0.0013	-	+/-	-	-	-/-
Q13310	PABP4	PABPC4	Polyadenylate-binding protein 4	0.0098	0.0092	1.06	0.0182	0.0170	1.07
Q9UNF0	PACN2	PACSIN2	Protein kinase C and casein kinase substrate in neurons protein 2	0.0026	0.0049	0.53	0.0107	0.0076	1.40
Q9UKS6	PACN3	PACSIN3	Protein kinase C and casein kinase substrate in neurons protein 3	0.0004	0.0019	0.23	-	0.0122	-/+
Q9Y2J8	PADI2	PADI2	Protein-arginine deiminase type-2	0.0001	-	+/-	0.0054	-	+/-
Q9UM07	PADI4	PADI4	Protein-arginine deiminase type-4	0.0014	-	+/-	0.0177	-	+/-
Q99487	PAFA2	PAFAH2	Platelet-activating factor acetylhydrolase 2, cytoplasmic	0.0010	0.0004	2.18	0.0051	0.0053	0.97

O14832	PAHX	PHYH	Phytanoyl-CoA dioxygenase, peroxisomal	0.0096	0.0258	0.37	0.0106	0.0442	0.24
P05121	PAI1	SERPINE1	Plasminogen activator inhibitor 1	0.0054	-	+/-	-	-	-/-
Q9H074	PAIP1	PAIP1	Polyadenylate-binding protein-interacting protein 1	-	-	-/-	0.0043	-	+/-
Q8NC51	PAIRB	SERBP1	Plasminogen activator inhibitor 1 RNA-binding protein	0.0028	0.0002	18.90	0.0044	-	+/-
Q13153	PAK1	PAK1	Serine/threonine-protein kinase PAK 1	-	0.0003	-/+	-	-	-/-
Q13177	PAK2	PAK2	Serine/threonine-protein kinase PAK 2	0.0018	0.0019	0.98	0.0143	0.0050	2.85
O75914	PAK3	PAK3	Serine/threonine-protein kinase PAK 3	-	-	-/-	0.0063	-	+/-
P0DN37	PAL4G	PPIAL4G	Peptidyl-prolyl cis-trans isomerase A-like 4G	0.0002	-	+/-	-	-	-/-
Q8WX93	PALLD	PALLD	Palladin	0.0014	-	+/-	0.0053	-	+/-
O75781	PALM	PALM	Paralemmin-1	0.0002	0.0003	0.63	-	-	-/-
A6NDB9	PALM3	PALM3	Paralemmin-3	0.0006	0.0006	1.14	-	0.0033	-/+
Q9NP74	PALMD	PALMD	Palmdelphin	0.0011	-	+/-	-	-	-/-
O43252	PAPS1	PAPSS1	Bifunctional 3'-phosphoadenosine 5'-phosphosulfate synthase 1	-	-	-/-	0.0038	-	+/-
O95340	PAPS2	PAPSS2	Bifunctional 3'-phosphoadenosine 5'-phosphosulfate synthase 2	0.0047	0.0024	1.98	0.0148	0.0147	1.00
Q53GL7	PAR10	PARP10	Protein mono-ADP-ribosyltransferase PARP10	0.0003	-	+/-	-	-	-/-
Q460N5	PAR14	PARP14	Protein mono-ADP-ribosyltransferase PARP14	-	-	-/-	0.0021	-	+/-

Q86W56	PARG	PARG	Poly(ADP-ribose) glycohydrolase	0.0007	-	+/-	-	-	-/-
Q99497	PARK7	PARK7	Protein/nucleic acid deglycase DJ-1	0.1772	0.2536	0.70	0.0661	0.1316	0.50
P09874	PARP1	PARP1	Poly [ADP-ribose] polymerase 1	0.0050	0.0015	3.46	0.0226	0.0059	3.80
Q9UKK3	PARP4	PARP4	Protein mono-ADP-ribosyltransferase PARP4	0.0002	-	+/-	0.0067	-	+/-
Q8IXQ6	PARP9	PARP9	Protein mono-ADP-ribosyltransferase PARP9	0.0002	-	+/-	0.0057	0.0021	2.73
Q9NVD7	PARVA	PARVA	Alpha-parvin	0.0011	0.0027	0.41	0.0113	0.0117	0.97
P49023	PAXI	PXN	Paxillin	0.0001	0.0003	0.32	-	-	-/-
Q9BUH6	PAXX	PAXX	Protein PAXX	0.0239	0.0125	1.91	0.0088	0.0091	0.97
Q9BVG4	PBDC1	PBDC1	Protein PBDC1	0.0141	0.0100	1.41	0.0133	0.0064	2.08
Q96AQ6	PBIP1	PBXIP1	Pre-B-cell leukemia transcription factor-interacting protein 1	0.0001	-	+/-	0.0053	-	+/-
P30039	PBLD	PBLD	Phenazine biosynthesis-like domain-containing protein	0.0452	0.1005	0.45	0.0327	0.1655	0.20
Q8NF37	PCAT1	LPCAT1	Lysophosphatidylcholine acyltransferase 1	0.0003	-	+/-	-	-	-/-
Q7L5N7	PCAT2	LPCAT2	Lysophosphatidylcholine acyltransferase 2	-	-	-/-	0.0036	-	+/-
Q15365	PCBP1	PCBP1	Poly(rC)-binding protein 1	0.0689	0.0558	1.23	0.1045	0.0874	1.20
Q15366-4	PCBP2	PCBP2	Isoform 4 of Poly(rC)-binding protein 2	-	-	-/-	0.0050	-	+/-
Q15366	PCBP2	PCBP2	Poly(rC)-binding protein 2	0.0289	0.0322	0.90	0.0410	0.0412	1.00
Q15366-2	PCBP2	PCBP2	Isoform 2 of Poly(rC)-binding protein 2	-	-	-/-	0.0416	0.0366	1.14
P57721	PCBP3	PCBP3	Poly(rC)-binding protein 3	0.0004	0.0003	1.20	0.0224	0.0185	1.21

P05165	PCCA	PCCA	Propionyl-CoA carboxylase alpha chain, mitochondrial	0.0133	0.0217	0.61	0.0213	0.0492	0.43
P05166	PCCB	PCCB	Propionyl-CoA carboxylase beta chain, mitochondrial	0.0153	0.0194	0.79	0.0392	0.0682	0.57
P35558	PCKGC	PCK1	Phosphoenolpyruvate carboxykinase, cytosolic [GTP]	0.0036	0.0260	0.14	0.0096	0.0840	0.11
Q16822-2	PCKGM	PCK2	Isoform 2 of Phosphoenolpyruvate carboxykinase [GTP], mitochondrial	0.0007	-	+/-	-	-	-/-
Q16822	PCKGM	PCK2	Phosphoenolpyruvate carboxykinase [GTP], mitochondrial	0.1040	0.2327	0.45	0.0875	0.2260	0.39
Q7Z2X4	PCLI1	PID1	PTB-containing, cubilin and LRP1-interacting protein	-	0.0004	-/+	-	-	-/-
Q96MG8	PCMD1	PCMTD1	Protein-L-isoaspartate O-methyltransferase domain-containing protein 1	0.0006	0.0005	1.15	-	-	-/-
P12004	PCNA	PCNA	Proliferating cell nuclear antigen	0.0195	0.0181	1.08	0.0127	0.0083	1.52
Q8WW12	PCNP	PCNP	PEST proteolytic signal-containing nuclear protein	0.0015	-	+/-	-	-	-/-
O95613	PCNT	PCNT	Pericentrin	-	-	-/-	-	0.0004	-/+
Q15113	PCOC1	PCOLCE	Procollagen C-endopeptidase enhancer 1	0.0006	-	+/-	-	-	-/-
P42785	PCP	PRCP	Lysosomal Pro-X carboxypeptidase	0.0025	0.0004	5.68	0.0137	0.0110	1.25
P49585	PCY1A	PCYT1A	Choline-phosphate cytidylyltransferase A	0.0021	0.0018	1.16	0.0073	0.0036	2.02
Q99447	PCY2	PCYT2	Ethanolamine-phosphate cytidylyltransferase	0.0086	0.0177	0.49	0.0076	0.0236	0.32
Q9UHG3	PCYOX	PCYOX1	Prenylcysteine oxidase 1	0.0211	0.0176	1.20	0.0441	0.0605	0.73

Q9BUL8	PDC10	PDCD10	Programmed cell death protein 10	0.0052	0.0052	1.01	0.0096	0.0064	1.50
Q8WUM4-2	PDC6I	PDCD6IP	Isoform 2 of Programmed cell death 6-interacting protein	0.0001	-	+/-	-	-	-/-
Q8WUM4	PDC6I	PDCD6IP	Programmed cell death 6-interacting protein	0.0155	0.0093	1.67	0.0266	0.0228	1.17
Q53EL6	PDCD4	PDCD4	Programmed cell death protein 4	0.0009	0.0013	0.67	0.0086	0.0070	1.23
O14737	PDCD5	PDCD5	Programmed cell death protein 5	0.0013	0.0027	0.49	0.0098	-	+/-
O75340	PDCD6	PDCD6	Programmed cell death protein 6	0.0289	0.0215	1.35	0.0397	0.0321	1.24
O75340-2	PDCD6	PDCD6	Isoform 2 of Programmed cell death protein 6	-	-	-/-	0.0233	-	+/-
Q9H2J4	PDCL3	PDCL3	Phosducin-like protein 3	0.0032	0.0038	0.85	-	-	-/-
Q6L8Q7	PDE12	PDE12	2',5'-phosphodiesterase 12	0.0001	0.0012	0.09	0.0065	-	+/-
Q01064	PDE1B	PDE1B	Calcium/calmodulin-dependent 3',5'-cyclic nucleotide phosphodiesterase 1B	0.0001	-	+/-	-	-	-/-
Q08493	PDE4C	PDE4C	cAMP-specific 3',5'-cyclic phosphodiesterase 4C	-	0.0002	-/+	-	-	-/-
O43924	PDE6D	PDE6D	Retinal rod rhodopsin-sensitive cGMP 3',5'-cyclic phosphodiesterase subunit delta	0.0008	0.0018	0.44	-	-	-/-
P07237	PDIA1	P4HB	Protein disulfide-isomerase	0.5231	0.2956	1.77	0.2009	0.1615	1.24
P30101	PDIA3	PDIA3	Protein disulfide-isomerase A3	0.3163	0.1200	2.64	0.1761	0.1552	1.13
P13667	PDIA4	PDIA4	Protein disulfide-isomerase A4	0.2127	0.0912	2.33	0.1764	0.1077	1.64
Q14554	PDIA5	PDIA5	Protein disulfide-isomerase A5	0.0105	0.0127	0.82	0.0180	0.0217	0.83
Q15084	PDIA6	PDIA6	Protein disulfide-isomerase A6	0.1466	0.0922	1.59	0.0849	0.0692	1.23

Q9Y2S7	PDIP2	POLDIP2	Polymerase delta-interacting protein 2	0.0042	0.0083	0.51	0.0095	0.0098	0.97
Q9BY77	PDIP3	POLDIP3	Polymerase delta-interacting protein 3	-	0.0006	-/+	-	-	-/-
Q15118	PDK1	PDK1	[Pyruvate dehydrogenase (acetyl-transferring)] kinase isozyme 1, mitochondrial	-	0.0002	-/+	-	-	-/-
Q15119	PDK2	PDK2	[Pyruvate dehydrogenase (acetyl-transferring)] kinase isozyme 2, mitochondrial	-	0.0011	-/+	-	-	-/-
Q16654	PDK4	PDK4	[Pyruvate dehydrogenase (acetyl-transferring)] kinase isozyme 4, mitochondrial	-	0.0022	-/+	-	0.0049	-/+
O00151	PDLI1	PDLIM1	PDZ and LIM domain protein 1	0.0132	0.0157	0.84	0.0124	0.0093	1.34
Q96JY6	PDLI2	PDLIM2	PDZ and LIM domain protein 2	0.0028	0.0058	0.48	-	0.0053	-/+
Q96JY6-3	PDLI2	PDLIM2	Isoform 3 of PDZ and LIM domain protein 2	-	-	-/-	-	0.0074	-/+
Q53GG5-2	PDLI3	PDLIM3	Isoform 2 of PDZ and LIM domain protein 3	0.0007	-	+/-	-	-	-/-
Q53GG5	PDLI3	PDLIM3	PDZ and LIM domain protein 3	0.0008	0.0003	2.43	-	-	-/-
Q96HC4-4	PDLI5	PDLIM5	Isoform 4 of PDZ and LIM domain protein 5	-	0.0002	-/+	-	-	-/-
Q96HC4	PDLI5	PDLIM5	PDZ and LIM domain protein 5	0.0062	0.0087	0.71	0.0287	0.0476	0.60
Q9NR12	PDLI7	PDLIM7	PDZ and LIM domain protein 7	0.0010	-	+/-	0.0108	0.0042	2.60
Q9P0J1	PDP1	PDP1	[Pyruvate dehydrogenase [acetyl-transferring]]-phosphatase 1, mitochondrial	0.0008	-	+/-	-	-	-/-
Q8NCN5	PDPR	PDPR	Pyruvate dehydrogenase phosphatase regulatory subunit, mitochondrial	-	-	-/-	0.0046	-	+/-
Q9NUG6	PDRG1	PDRG1	p53 and DNA damage-regulated protein 1	0.0002	-	+/-	-	-	-/-

Q29RF7	PDS5A	PDS5A	Sister chromatid cohesion protein PDS5 homolog A	0.0001	-	+/-	0.0017	0.0011	1.46
P52945	PDX1	PDX1	Pancreas/duodenum homeobox protein 1	-	0.0005	-/+	-	-	-/-
Q6P996-3	PDXD1	PDXDC1	Isoform 3 of Pyridoxal-dependent decarboxylase domain-containing protein 1	0.0017	-	+/-	-	-	-/-
Q6P996	PDXD1	PDXDC1	Pyridoxal-dependent decarboxylase domain-containing protein 1	0.0058	0.0054	1.09	0.0063	0.0025	2.53
O00764	PDXK	PDXK	Pyridoxal kinase	0.0168	0.0170	0.99	0.0111	0.0179	0.62
Q5EBL8	PDZ11	PDZD11	PDZ domain-containing protein 11	0.0026	0.0077	0.33	-	-	-/-
Q15121	PEA15	PEA15	Astrocytic phosphoprotein PEA-15	0.0172	0.0102	1.69	-	-	-/-
Q13951	PEBB	CBFB	Core-binding factor subunit beta	-	0.0017	-/+	-	-	-/-
P30086	PEBP1	PEBP1	Phosphatidylethanolamine-binding protein 1	0.1038	0.1120	0.93	0.1231	0.1899	0.65
P16284	PECA1	PECAM1	Platelet endothelial cell adhesion molecule	0.0025	-	+/-	0.0024	-	+/-
Q9BY49	PECR	PECR	Peroxisomal trans-2-enoyl-CoA reductase	0.0420	0.0612	0.69	0.0569	0.0944	0.60
P36955	PEDF	SERPINF1	Pigment epithelium-derived factor	0.0286	0.0036	8.05	0.0327	0.0123	2.65
Q9UBV8	PEF1	PEF1	Peflin	0.0075	0.0080	0.93	-	0.0120	-/+
Q9BRX2	PELO	PELO	Protein pelota homolog	-	0.0002	-/+	-	-	-/-
Q8IZL8	PELP1	PELP1	Proline-, glutamic acid- and leucine-rich protein 1	-	-	-/-	0.0031	-	+/-
P12955	PEPD	PEPD	Xaa-Pro dipeptidase	0.0125	0.0108	1.16	0.0167	0.0244	0.69
O60437	PEPL	PPL	Periplakin	0.0014	0.0019	0.72	-	0.0014	-/+

Q8NDH3	PEPL1	NPEPL1	Probable aminopeptidase NPEPL1	-	-	-/-	0.0040	-	+/-
P11678	PERE	EPX	Eosinophil peroxidase	0.0110	0.0004	25.37	0.0100	0.0059	1.69
P05164	PERM	MPO	Myeloperoxidase	0.0444	0.0023	19.12	0.1171	0.0114	10.26
O43933	PEX1	PEX1	Peroxisome biogenesis factor 1	0.0002	-	+/-	-	-	-/-
O75381	PEX14	PEX14	Peroxisomal membrane protein PEX14	-	0.0012	-/+	-	-	-/-
Q9Y5Y5	PEX16	PEX16	Peroxisomal membrane protein PEX16	-	-	-/-	0.0060	-	+/-
P40855	PEX19	PEX19	Peroxisomal biogenesis factor 19	0.0197	0.0124	1.58	0.0045	-	+/-
P56589	PEX3	PEX3	Peroxisomal biogenesis factor 3	-	-	-/-	0.0065	-	+/-
P50542	PEX5	PEX5	Peroxisomal targeting signal 1 receptor	-	0.0011	-/+	-	-	-/-
O60925	PFD1	PFDN1	Prefoldin subunit 1	0.0012	-	+/-	-	-	-/-
Q9UHV9	PFD2	PFDN2	Prefoldin subunit 2	0.0193	0.0052	3.70	0.0088	0.0091	0.97
P61758	PFD3	VBP1	Prefoldin subunit 3	0.0031	0.0010	2.97	-	-	-/-
Q9NQP4	PFD4	PFDN4	Prefoldin subunit 4	-	0.0014	-/+	-	-	-/-
Q99471	PFD5	PFDN5	Prefoldin subunit 5	0.0080	0.0027	2.96	-	-	-/-
P17858	PFKAL	PFKL	ATP-dependent 6-phosphofructokinase, liver type	0.0020	0.0035	0.56	0.0189	0.0378	0.50
P08237	PFKAM	PFKM	ATP-dependent 6-phosphofructokinase, muscle type	0.0001	-	+/-	-	-	-/-
Q01813	PFKAP	PFKP	ATP-dependent 6-phosphofructokinase, platelet type	0.0001	-	+/-	0.0043	-	+/-
Q9BZM1	PG12A	PLA2G12A	Group XIIA secretory phospholipase A2	0.0003	-	+/-	-	-	-/-

Q9BX93	PG12B	PLA2G12B	Group XIIB secretory phospholipase A2-like protein	0.0067	0.0189	0.35	-	0.0257	-/+
P18669	PGAM1	PGAM1	Phosphoglycerate mutase 1	0.1081	0.0994	1.09	0.1026	0.1866	0.55
P15259	PGAM2	PGAM2	Phosphoglycerate mutase 2	0.0015	0.0009	1.64	-	-	-/-
Q8N0Y7	PGAM4	PGAM4	Probable phosphoglycerate mutase 4	0.0013	-	+/-	-	-	-/-
Q96HS1	PGAM5	PGAM5	Serine/threonine-protein phosphatase PGAM5, mitochondrial	0.0122	0.0175	0.69	0.0090	0.0207	0.43
P98160	PGBM	HSPG2	Basement membrane-specific heparan sulfate proteoglycan core protein	0.0224	0.0053	4.23	0.0325	0.0244	1.33
P15428	PGDH	HPGD	15-hydroxyprostaglandin dehydrogenase [NAD(+)]	0.0199	0.0270	0.74	0.0245	0.0138	1.78
Q9H7Z7	PGES2	PTGES2	Prostaglandin E synthase 2	0.0107	0.0100	1.06	0.0129	0.0096	1.35
P00558	PGK1	PGK1	Phosphoglycerate kinase 1	0.1635	0.1433	1.14	0.2158	0.1406	1.53
P36871	PGM1	PGM1	Phosphoglucomutase-1	0.0662	0.1538	0.43	0.0712	0.1924	0.37
Q96G03	PGM2	PGM2	Phosphoglucomutase-2	0.0059	0.0058	1.03	0.0433	0.0310	1.40
Q6PCE3	PGM2L	PGM2L1	Glucose 1,6-bisphosphate synthase	-	0.0001	-/+	-	-	-/-
Q15124	PGM5	PGM5	Phosphoglucomutase-like protein 5	-	-	-/-	0.0032	0.0033	0.97
A6NDG6	PGP	PGP	Glycerol-3-phosphate phosphatase	0.0011	0.0017	0.66	-	-	-/-
Q9NXJ5	PGPI	PGPEP1	Pyroglutamyl-peptidase 1	-	0.0039	-/+	-	-	-/-
O00264	PGRC1	PGRMC1	Membrane-associated progesterone receptor component 1	0.1222	0.0781	1.57	0.0968	0.0964	1.00

O15173	PGRC2	PGRMC2	Membrane-associated progesterone receptor component 2	0.0380	0.0253	1.50	0.0484	0.0430	1.13
O75594	PGRP1	PGLYRP1	Peptidoglycan recognition protein 1	0.0028	-	+/-	0.0287	-	+/-
Q96PD5	PGRP2	PGLYRP2	N-acetylmuramoyl-L-alanine amidase	0.0004	0.0004	1.00	0.0041	0.0042	0.97
P21810	PGS1	BGN	Biglycan	0.0208	0.0236	0.88	0.0610	0.0529	1.15
P07585	PGS2	DCN	Decorin	0.0164	0.0208	0.79	0.0487	0.0232	2.10
Q92696	PGTA	RABGGTA	Geranylgeranyl transferase type-2 subunit alpha	-	0.0007	-/+	0.0032	-	+/-
P53609	PGTB1	PGGT1B	Geranylgeranyl transferase type-1 subunit beta	0.0002	-	+/-	-	-	-/-
P00439	PH4H	PAH	Phenylalanine-4-hydroxylase	0.0117	0.0450	0.26	0.0106	0.0822	0.13
P35232	PHB	PHB	Prohibitin	0.3277	0.1329	2.47	0.1094	0.1190	0.92
Q99623	PHB2	PHB2	Prohibitin-2	0.0714	0.1023	0.70	0.0783	0.0933	0.84
O75151	PHF2	PHF2	Lysine-specific demethylase PHF2	0.0001	-	+/-	-	-	-/-
P15735	PHKG2	PHKG2	Phosphorylase b kinase gamma catalytic chain, liver/testis isoform	-	0.0006	-/+	-	0.0065	-/+
P80108	PHLD	GPLD1	Phosphatidylinositol-glycan-specific phospholipase D	0.0005	-	+/-	-	-	-/-
Q9Y3A3	PHOCN	MOB4	MOB-like protein phocein	0.0005	0.0008	0.70	-	-	-/-
Q9NRX4	PHP14	PHPT1	14 kDa phosphohistidine phosphatase	0.0220	0.0186	1.19	0.0143	0.0148	0.97
P61457	PHS	PCBD1	Pterin-4-alpha-carbinolamine dehydratase	0.0357	0.0397	0.90	0.0411	0.0573	0.72
Q9H0N5	PHS2	PCBD2	Pterin-4-alpha-carbinolamine dehydratase 2	0.0023	0.0042	0.55	-	-	-/-

Q5SRE7	PHYD1	PHYHD1	Phytanoyl-CoA dioxygenase domain-containing protein 1	0.0076	0.0154	0.50	-	0.0150	-/+
O43692	PI15	PI15	Peptidase inhibitor 15	0.0006	-	+/-	-	-	-/-
P48426	PI42A	PIP4K2A	Phosphatidylinositol 5-phosphate 4-kinase type-2 alpha	0.0007	-	+/-	0.0053	-	+/-
Q8TBX8	PI42C	PIP4K2C	Phosphatidylinositol 5-phosphate 4-kinase type-2 gamma	0.0003	-	+/-	0.0057	-	+/-
Q13492	PICAL	PICALM	Phosphatidylinositol-binding clathrin assembly protein	0.0021	0.0017	1.22	0.0149	0.0091	1.65
Q9NRD5	PICK1	PICK1	PRKCA-binding protein	0.0002	-	+/-	-	-	-/-
P01833	PIGR	PIGR	Polymeric immunoglobulin receptor	0.0298	0.0003	104.61	0.0425	-	+/-
Q9NWS0	PIHD1	PIH1D1	PIH1 domain-containing protein 1	0.0040	0.0033	1.20	-	-	-/-
P22061	PIMT	PCMT1	Protein-L-isoaspartate(D-aspartate) O-methyltransferase	0.0413	0.0559	0.74	-	0.0214	-/+
Q13526	PIN1	PIN1	Peptidyl-prolyl cis-trans isomerase NIMA-interacting 1	0.0083	0.0110	0.76	-	-	-/-
Q9Y237	PIN4	PIN4	Peptidyl-prolyl cis-trans isomerase NIMA-interacting 4	0.0003	0.0008	0.42	-	-	-/-
Q9H307	PININ	PNN	Pinin	0.0001	-	+/-	-	-	-/-
A0A0B4J2F0	PIOS1	PIGBOS1	Protein PIGBOS1	-	0.0018	-/+	-	-	-/-
P12273	PIP	PIP	Prolactin-inducible protein	0.0198	0.0273	0.73	-	-	-/-
Q00169	PIPNA	PITPNA	Phosphatidylinositol transfer protein alpha isoform	0.0061	0.0063	0.97	0.0103	0.0107	0.97

P48739	PIPNB	PITPNB	Phosphatidylinositol transfer protein beta isoform	0.0154	0.0156	0.98	0.0169	0.0211	0.80
O00625	PIR	PIR	Pirin	0.0044	0.0031	1.43	0.0060	-	+/-
Q9UG56	PISD	PISD	Phosphatidylserine decarboxylase proenzyme, mitochondrial	-	0.0002	-/+	-	-	-/-
Q9GZP4	PITH1	PITHD1	PITH domain-containing protein 1	0.0031	0.0073	0.43	0.0127	-	+/-
Q9HB21	PKHA1	PLEKHA1	Pleckstrin homology domain-containing family A member 1	-	0.0002	-/+	-	-	-/-
Q9Y2H5	PKHA6	PLEKHA6	Pleckstrin homology domain-containing family A member 6	0.0000	-	+/-	-	-	-/-
Q9H8W4	PKHF2	PLEKHF2	Pleckstrin homology domain-containing family F member 2	0.0006	-	+/-	-	-	-/-
Q8TD55	PKHO2	PLEKHO2	Pleckstrin homology domain-containing family O member 2	0.0004	0.0009	0.45	-	-	-/-
Q13835	PKP1	PKP1	Plakophilin-1	0.0002	0.0006	0.31	-	-	-/-
Q99959	PKP2	PKP2	Plakophilin-2	0.0006	0.0006	0.98	-	0.0070	-/+
P14923	PLAK	JUP	Junction plakoglobin	0.0109	0.0713	0.15	0.0240	0.0299	0.80
Q9Y263	PLAP	PLAA	Phospholipase A-2-activating protein	-	-	-/-	0.0066	0.0040	1.66
P53816	PLAT3	PLAAT3	Phospholipase A and acyltransferase 3	-	0.0006	-/+	-	-	-/-
Q6P4A8	PLBL1	PLBD1	Phospholipase B-like 1	0.0041	0.0005	8.02	0.0226	-	+/-
Q8NHP8	PLBL2	PLBD2	Putative phospholipase B-like 2	0.0008	0.0006	1.50	0.0086	-	+/-

O15120	PLCB	AGPAT2	1-acyl-sn-glycerol-3-phosphate acyltransferase beta	0.0002	-	+/-	-	-	-/-
Q01970	PLCB3	PLCB3	1-phosphatidylinositol 4,5-bisphosphate phosphodiesterase beta-3	0.0000	-	+/-	-	-	-/-
P51178	PLCD1	PLCD1	1-phosphatidylinositol 4,5-bisphosphate phosphodiesterase delta-1	0.0002	0.0001	1.60	-	-	-/-
P16885	PLCG2	PLCG2	1-phosphatidylinositol 4,5-bisphosphate phosphodiesterase gamma-2	-	-	-/-	0.0040	0.0023	1.73
Q4KWH8	PLCH1	PLCH1	1-phosphatidylinositol 4,5-bisphosphate phosphodiesterase eta-1	0.0002	-	+/-	-	-	-/-
Q63HM9	PLCX3	PLCXD3	PI-PLC X domain-containing protein 3	0.0005	0.0004	1.40	-	-	-/-
Q13393	PLD1	PLD1	Phospholipase D1	0.0001	-	+/-	-	-	-/-
Q8IV08	PLD3	PLD3	Phospholipase D3	0.0048	0.0011	4.24	0.0280	0.0110	2.55
Q15149-3	PLEC	PLEC	Isoform 3 of Plectin	0.0030	0.0011	2.80	0.0211	-	+/-
Q15149	PLEC	PLEC	Plectin	0.0172	0.0023	7.51	0.0048	0.0152	0.31
Q15149-8	PLEC	PLEC	Isoform 8 of Plectin	-	-	-/-	-	0.0122	-/+
P08567	PLEK	PLEK	Pleckstrin	0.0001	0.0005	0.25	0.0076	-	+/-
Q6UW63	PLGT2	POGLUT2	Protein O-glucosyltransferase 2	0.0001	-	+/-	-	-	-/-
Q7Z4H8	PLGT3	POGLUT3	Protein O-glucosyltransferase 3	0.0002	0.0004	0.57	0.0093	0.0062	1.50
O60240	PLIN1	PLIN1	Perilipin-1	0.0001	-	+/-	-	-	-/-
Q99541	PLIN2	PLIN2	Perilipin-2	0.0068	0.0020	3.43	0.0116	-	+/-
O60664	PLIN3	PLIN3	Perilipin-3	0.0260	0.0123	2.13	0.0268	0.0130	2.06

Q96Q06	PLIN4	PLIN4	Perilipin-4	-	0.0001	-/+	-	0.0026	-/+
Q00G26	PLIN5	PLIN5	Perilipin-5	0.0008	0.0033	0.25	-	-	-/-
Q9H4B4	PLK3	PLK3	Serine/threonine-protein kinase PLK3	0.0002	-	+/-	-	-	-/-
P00747	PLMN	PLG	Plasminogen	0.0219	0.0045	4.84	0.0260	0.0248	1.05
Q02809	PLOD1	PLOD1	Procollagen-lysine,2-oxoglutarate 5-dioxygenase 1	0.0009	0.0001	6.53	0.0102	-	+/-
O00469	PLOD2	PLOD2	Procollagen-lysine,2-oxoglutarate 5-dioxygenase 2	0.0004	-	+/-	-	-	-/-
O60568	PLOD3	PLOD3	Multifunctional procollagen lysine hydroxylase and glycosyltransferase LH3	0.0071	0.0007	10.16	0.0207	0.0039	5.36
O94903	PLPHP	PLPBP	Pyridoxal phosphate homeostasis protein	0.0111	0.0235	0.47	0.0233	0.0350	0.67
Q8IY17	PLPL6	PNPLA6	Neuropathy target esterase	0.0005	-	+/-	0.0025	0.0013	1.96
Q96GD0	PLPP	PDXP	Pyridoxal phosphate phosphatase	0.0040	0.0073	0.55	0.0128	0.0360	0.36
O14495	PLPP3	PLPP3	Phospholipid phosphatase 3	-	-	-/-	-	0.0062	-/+
O43660	PLRG1	PLRG1	Pleiotropic regulator 1	0.0001	-	+/-	-	-	-/-
Q9HBL7	PLRKT	PLGRKT	Plasminogen receptor (KT)	0.0022	-	+/-	-	-	-/-
O15162	PLS1	PLSCR1	Phospholipid scramblase 1	-	-	-/-	0.0175	-	+/-
Q14651	PLSI	PLS1	Plastin-1	0.0012	0.0021	0.54	0.0108	0.0112	0.97
P13796	PLSL	LCP1	Plastin-2	0.1058	0.0517	2.05	0.2106	0.1167	1.80
P13797	PLST	PLS3	Plastin-3	0.0500	0.0373	1.34	0.1182	0.0752	1.57
O43157	PLXB1	PLXNB1	Plexin-B1	0.0001	-	+/-	-	-	-/-

O15031	PLXB2	PLXNB2	Plexin-B2	0.0025	0.0003	7.39	0.0031	0.0010	2.97
O43808	PM34	SLC25A17	Peroxisomal membrane protein PMP34	-	-	-/-	0.0051	-	+/-
P07738	PMGE	BPGM	Bisphosphoglycerate mutase	0.0100	0.0045	2.23	0.0058	0.0060	0.97
Q8WZA1	PMGT1	POMGNT1	Protein O-linked-mannose beta-1,2-N-acetylglucosaminyltransferase 1	-	-	-/-	0.0023	-	+/-
P29590	PML	PML	Protein PML	0.0041	0.0016	2.63	0.0097	0.0032	3.04
P29590-10	PML	PML	Isoform PML-7 of Protein PML	-	-	-/-	-	0.0089	-/+
Q92871	PMM1	PMM1	Phosphomannomutase 1	-	0.0003	-/+	-	-	-/-
O15305	PMM2	PMM2	Phosphomannomutase 2	0.0092	0.0122	0.75	0.0054	0.0087	0.62
Q15126	PMVK	PMVK	Phosphomevalonate kinase	0.0302	0.0178	1.70	0.0212	0.0219	0.97
Q6XQN6	PNCB	NAPRT	Nicotinate phosphoribosyltransferase	0.0070	0.0307	0.23	0.0233	0.0585	0.40
Q8N490	PNKD	PNKD	Probable hydrolase PNKD	0.0013	-	+/-	0.0058	-	+/-
Q9NRX1	PNO1	PNO1	RNA-binding protein PNO1	0.0012	0.0007	1.66	-	-	-/-
P00491	PNPH	PNP	Purine nucleoside phosphorylase	0.0851	0.0825	1.03	0.1048	0.1487	0.70
Q9NVS9	PNPO	PNPO	Pyridoxine-5'-phosphate oxidase	0.0198	0.0378	0.53	0.0133	0.0219	0.61
Q8TCS8	PNPT1	PNPT1	Polyribonucleotide nucleotidyltransferase 1, mitochondrial	-	0.0002	-/+	0.0032	-	+/-
Q8TEM1	PO210	NUP210	Nuclear pore membrane glycoprotein 210	0.0019	-	+/-	0.0040	0.0013	3.00
Q8WVV4	POF1B	POF1B	Protein POF1B	-	0.0002	-/+	-	-	-/-
Q9Y244	POMP	POMP	Proteasome maturation protein	-	0.0011	-/+	-	-	-/-
P27169	PON1	PON1	Serum paraoxonase/arylesterase 1	0.2764	0.0682	4.05	0.0836	0.0926	0.90

Q15165	PON2	PON2	Serum paraoxonase/arylesterase 2	0.2433	0.0172	14.14	0.0783	0.0380	2.06
Q15166	PON3	PON3	Serum paraoxonase/lactonase 3	0.1020	0.0298	3.42	0.0559	0.0942	0.59
Q99575	POP1	POP1	Ribonucleases P/MRP protein subunit POP1	-	-	-/-	0.0013	-	+/-
O75817	POP7	POP7	Ribonuclease P protein subunit p20	0.0009	0.0006	1.60	-	-	-/-
Q15063-2	POSTN	POSTN	Isoform 2 of Periostin	0.0043	-	+/-	0.0040	-	+/-
Q15063	POSTN	POSTN	Periostin	0.0176	-	+/-	0.0309	-	+/-
Q15063-3	POSTN	POSTN	Isoform 3 of Periostin	0.0106	-	+/-	0.0453	-	+/-
Q6S8J3	POTEE	POTEE	POTE ankyrin domain family member E	0.0003	-	+/-	-	-	-/-
A5A3E0	POTEF	POTEF	POTE ankyrin domain family member F	0.0009	0.0003	3.46	0.0080	0.0036	2.24
P0CG38	POTEI	POTEI	POTE ankyrin domain family member I	0.0063	0.0010	6.16	-	-	-/-
P0CG39	POTEJ	POTEJ	POTE ankyrin domain family member J	0.0003	-	+/-	0.0014	-	+/-
B7ZBB8	PP13G	PPP1R3G	Protein phosphatase 1 regulatory subunit 3G	-	0.0004	-/+	-	-	-/-
P62136-2	PP1A	PPP1CA	Isoform 2 of Serine/threonine-protein phosphatase PP1-alpha catalytic subunit	-	0.0004	-/+	-	-	-/-
P62136	PP1A	PPP1CA	Serine/threonine-protein phosphatase PP1-alpha catalytic subunit	0.0211	0.0199	1.06	0.0642	0.0664	0.97
P62140	PP1B	PPP1CB	Serine/threonine-protein phosphatase PP1-beta catalytic subunit	0.0150	0.0180	0.84	0.0460	0.0488	0.94
P36873	PP1G	PPP1CC	Serine/threonine-protein phosphatase PP1-gamma catalytic subunit	0.0157	0.0079	1.99	0.0594	0.0535	1.11
Q15435	PP1R7	PPP1R7	Protein phosphatase 1 regulatory subunit 7	0.0117	0.0250	0.47	0.0136	0.0114	1.19

Q12972	PP1R8	PPP1R8	Nuclear inhibitor of protein phosphatase 1	0.0003	-	+/-	-	-	-/-
P67775	PP2AA	PPP2CA	Serine/threonine-protein phosphatase 2A catalytic subunit alpha isoform	-	-	-/-	0.0251	0.0172	1.46
P62714	PP2AB	PPP2CB	Serine/threonine-protein phosphatase 2A catalytic subunit beta isoform	0.0141	0.0124	1.14	0.0481	0.0270	1.78
P16298	PP2BB	PPP3CB	Serine/threonine-protein phosphatase 2B catalytic subunit beta isoform	-	-	-/-	0.0035	-	+/-
P60510	PP4C	PPP4C	Serine/threonine-protein phosphatase 4 catalytic subunit	0.0002	-	+/-	-	-	-/-
Q6NUP7	PP4R4	PPP4R4	Serine/threonine-protein phosphatase 4 regulatory subunit 4	-	0.0002	-/+	-	-	-/-
O75170	PP6R2	PPP6R2	Serine/threonine-protein phosphatase 6 regulatory subunit 2	-	0.0002	-/+	0.0022	0.0048	0.47
P13686	PPA5	ACP5	Tartrate-resistant acid phosphatase type 5	0.0005	-	+/-	0.0060	-	+/-
P24666-2	PPAC	ACP1	Isoform 2 of Low molecular weight phosphotyrosine protein phosphatase	0.0056	-	+/-	-	-	-/-
P24666	PPAC	ACP1	Low molecular weight phosphotyrosine protein phosphatase	0.0195	0.0172	1.13	0.0219	0.0301	0.73
P11117	PPAL	ACP2	Lysosomal acid phosphatase	0.0005	0.0005	1.01	0.0103	0.0050	2.05
P15309	PPAP	ACPP	Prostatic acid phosphatase	-	0.0027	-/+	-	-	-/-
P05186	PPBT	ALPL	Alkaline phosphatase, tissue-nonspecific isozyme	0.0054	0.0004	12.80	0.0040	-	+/-
P48147	PPCE	PREP	Prolyl endopeptidase	0.0007	0.0035	0.19	0.0116	0.0138	0.85

Q9HAB8	PPCS	PPCS	Phosphopantothenate--cysteine ligase	0.0089	0.0055	1.63	0.0103	0.0107	0.97
Q9UKL6	PPCT	PCTP	Phosphatidylcholine transfer protein	0.0008	0.0025	0.34	-	-	-/-
P10619	PPGB	CTSA	Lysosomal protective protein	0.0053	0.0042	1.24	0.0365	-	+/-
P62937	PPIA	PPIA	Peptidyl-prolyl cis-trans isomerase A	0.3200	0.3453	0.93	0.4109	0.2758	1.49
P23284	PPIB	PPIB	Peptidyl-prolyl cis-trans isomerase B	0.1150	0.1839	0.63	0.1529	0.1475	1.04
P45877	PPIC	PPIC	Peptidyl-prolyl cis-trans isomerase C	0.0023	0.0020	1.15	-	-	-/-
Q08752	PPID	PPID	Peptidyl-prolyl cis-trans isomerase D	0.0021	0.0025	0.82	0.0039	-	+/-
Q9UNP9	PPIE	PPIE	Peptidyl-prolyl cis-trans isomerase E	0.0015	0.0024	0.61	-	-	-/-
P30405	PPIF	PPIF	Peptidyl-prolyl cis-trans isomerase F, mitochondrial	0.0076	0.0221	0.34	0.0121	0.0289	0.42
Q13427	PPIG	PPIG	Peptidyl-prolyl cis-trans isomerase G	-	-	-/-	0.0013	-	+/-
O43447	PPIH	PPIH	Peptidyl-prolyl cis-trans isomerase H	0.0053	0.0114	0.46	-	-	-/-
Q9Y3C6	PPIL1	PPIL1	Peptidyl-prolyl cis-trans isomerase-like 1	0.0088	0.0146	0.60	-	-	-/-
Q9H2H8	PPIL3	PPIL3	Peptidyl-prolyl cis-trans isomerase-like 3	0.0127	0.0084	1.52	-	-	-/-
Q8WUA2	PPIL4	PPIL4	Peptidyl-prolyl cis-trans isomerase-like 4	0.0003	0.0001	2.36	-	-	-/-
Q9H939	PPIP2	PSTPIP2	Proline-serine-threonine phosphatase-interacting protein 2	0.0001	0.0002	0.50	-	-	-/-
P35813	PPM1A	PPM1A	Protein phosphatase 1A	0.0036	0.0126	0.28	0.0047	0.0234	0.20
O75688	PPM1B	PPM1B	Protein phosphatase 1B	0.0008	0.0018	0.44	0.0060	-	+/-
P49593	PPM1F	PPM1F	Protein phosphatase 1F	0.0009	0.0045	0.21	0.0068	-	+/-
O15355	PPM1G	PPM1G	Protein phosphatase 1G	0.0003	0.0003	1.29	-	-	-/-

Q8N3J5	PPM1K	PPM1K	Protein phosphatase 1K, mitochondrial	-	0.0003	-/+	-	-	-/-
Q9Y570	PPME1	PPME1	Protein phosphatase methylesterase 1	0.0007	0.0007	0.96	-	-	-/-
O95428	PPN	PAPLN	Papilin	0.0003	-	+/-	-	-	-/-
P50336	PPOX	PPOX	Protoporphyrinogen oxidase	0.0038	0.0010	4.01	0.0077	0.0052	1.49
P53041	PPP5	PPP5C	Serine/threonine-protein phosphatase 5	0.0002	0.0002	1.23	0.0049	-	+/-
O00743	PPP6	PPP6C	Serine/threonine-protein phosphatase 6 catalytic subunit	0.0021	0.0033	0.65	0.0128	0.0172	0.75
Q6NYC8-2	PPR18	PPP1R18	Isoform 2 of Phostensin	0.0001	-	+/-	-	-	-/-
Q86XI6	PPR3B	PPP1R3B	Protein phosphatase 1 regulatory subunit 3B	0.0006	0.0008	0.80	-	-	-/-
P50897	PPT1	PPT1	Palmitoyl-protein thioesterase 1	0.0137	0.0099	1.38	0.0155	0.0210	0.74
O60828-2	PQBP1	PQBP1	Isoform 2 of Polyglutamine-binding protein 1	0.0002	-	+/-	-	-	-/-
O75400	PR40A	PRPF40A	Pre-mRNA-processing factor 40 homolog A	-	-	-/-	0.0025	-	+/-
O75915	PRAF3	ARL6IP5	PRA1 family protein 3	0.0009	0.0005	1.86	0.0296	0.0214	1.38
Q8NCQ7	PRCA1	PROCA1	Protein PROCA1	0.0002	-	+/-	-	-	-/-
Q06830	PRDX1	PRDX1	Peroxiredoxin-1	0.3800	0.5225	0.73	0.4434	0.3430	1.29
P32119	PRDX2	PRDX2	Peroxiredoxin-2	0.2873	0.1839	1.56	0.1315	0.1548	0.85
P30048	PRDX3	PRDX3	Thioredoxin-dependent peroxide reductase, mitochondrial	0.2967	0.2391	1.24	0.1117	0.1804	0.62
Q13162	PRDX4	PRDX4	Peroxiredoxin-4	0.1056	0.0835	1.26	0.0838	0.1115	0.75
P30044	PRDX5	PRDX5	Peroxiredoxin-5, mitochondrial	0.0909	0.1104	0.82	0.1000	0.0922	1.08
P30041	PRDX6	PRDX6	Peroxiredoxin-6	0.2692	0.3078	0.87	0.1441	0.2156	0.67

Q9HCU5	PREB	PREB	Prolactin regulatory element-binding protein	0.0006	-	+/-	0.0071	-	+/-
P51888	PRELP	PRELP	Prolargin	0.0060	0.0025	2.41	0.0380	0.0184	2.06
Q5JRX3	PREP	PITRM1	Presequence protease, mitochondrial	0.0011	0.0019	0.60	0.0097	0.0089	1.09
P13727	PRG2	PRG2	Bone marrow proteoglycan	0.0046	-	+/-	0.0140	0.0125	1.12
Q92954	PRG4	PRG4	Proteoglycan 4	0.0002	0.0002	0.87	0.0035	-	+/-
Q92954-2	PRG4	PRG4	Isoform B of Proteoglycan 4	-	-	-/-	0.0033	-	+/-
P78527	PRKDC	PRKDC	DNA-dependent protein kinase catalytic subunit	0.0009	0.0001	7.79	0.0163	0.0064	2.55
O75569	PRKRA	PRKRA	Interferon-inducible double-stranded RNA-dependent protein kinase activator A	0.0013	0.0015	0.88	-	-	-/-
P04070	PROC	PROC	Vitamin K-dependent protein C	0.0005	0.0004	1.15	-	-	-/-
P07737	PROF1	PFN1	Profilin-1	0.4044	0.4226	0.96	0.3328	0.2106	1.58
P35080-2	PROF2	PFN2	Isoform IIb of Profilin-2	0.0057	0.0106	0.53	-	-	-/-
P35080	PROF2	PFN2	Profilin-2	0.0019	0.0066	0.29	-	-	-/-
Q16378	PROL4	PRR4	Proline-rich protein 4	-	0.0018	-/+	-	-	-/-
P07225	PROS	PROS1	Vitamin K-dependent protein S	0.0034	0.0004	9.53	0.0108	0.0029	3.70
Q9UMS4	PRP19	PRPF19	Pre-mRNA-processing factor 19	0.0048	0.0034	1.39	0.0119	0.0096	1.24
Q8WWY3	PRP31	PRPF31	U4/U6 small nuclear ribonucleoprotein Prp31	0.0002	0.0002	0.90	0.0059	-	+/-
O43172	PRP4	PRPF4	U4/U6 small nuclear ribonucleoprotein Prp4	0.0001	0.0004	0.19	-	-	-/-
Q13523	PRP4B	PRPF4B	Serine/threonine-protein kinase PRP4 homolog	-	-	-/-	0.0008	-	+/-

O94906	PRP6	PRPF6	Pre-mRNA-processing factor 6	0.0001	-	+/-	0.0033	-	+/-
Q6P2Q9	PRP8	PRPF8	Pre-mRNA-processing-splicing factor 8	0.0003	-	+/-	0.0061	0.0017	3.67
Q96S44	PRPK	TP53RK	EKC/KEOPS complex subunit TP53RK	0.0007	0.0017	0.40	-	-	-/-
P60891	PRPS1	PRPS1	Ribose-phosphate pyrophosphokinase 1	0.0073	0.0126	0.57	0.0146	0.0310	0.47
P11908	PRPS2	PRPS2	Ribose-phosphate pyrophosphokinase 2	0.0078	0.0249	0.31	0.0155	0.0286	0.54
P21108	PRPS3	PRPS1L1	Ribose-phosphate pyrophosphokinase 3	0.0012	0.0021	0.56	-	-	-/-
Q96M27	PRRC1	PRRC1	Protein PRRC1	0.0129	0.0031	4.19	0.0254	0.0115	2.20
P62333	PRS10	PSMC6	26S proteasome regulatory subunit 10B	0.0109	0.0131	0.83	0.0303	0.0231	1.31
P62191	PRS4	PSMC1	26S proteasome regulatory subunit 4	0.0116	0.0098	1.18	0.0169	0.0196	0.86
P17980	PRS6A	PSMC3	26S proteasome regulatory subunit 6A	0.0245	0.0218	1.13	0.0220	0.0226	0.97
P43686	PRS6B	PSMC4	26S proteasome regulatory subunit 6B	0.0238	0.0249	0.96	0.0215	0.0147	1.46
P35998	PRS7	PSMC2	26S proteasome regulatory subunit 7	0.0198	0.0116	1.71	0.0258	0.0148	1.74
P62195	PRS8	PSMC5	26S proteasome regulatory subunit 8	0.0127	0.0161	0.79	0.0317	0.0246	1.29
P24158	PRTN3	PRTN3	Myeloblastin	0.0340	0.0053	6.42	0.0960	0.0157	6.10
Q86TP1	PRUN1	PRUNE1	Exopolyphosphatase PRUNE1	-	-	-/-	0.0049	-	+/-
P20472	PRVA	PVALB	Parvalbumin alpha	-	0.0006	-/+	-	-	-/-
P55786	PSA	NPEPPS	Puromycin-sensitive aminopeptidase	0.0038	0.0017	2.25	0.0289	0.0185	1.56
P25786	PSA1	PSMA1	Proteasome subunit alpha type-1	0.0690	0.0465	1.48	0.0483	0.0500	0.97
P25787	PSA2	PSMA2	Proteasome subunit alpha type-2	0.0327	0.0379	0.86	0.0547	0.0614	0.89
P25788	PSA3	PSMA3	Proteasome subunit alpha type-3	0.0551	0.0285	1.94	0.0224	0.0281	0.80

P25789	PSA4	PSMA4	Proteasome subunit alpha type-4	0.0162	0.0133	1.22	0.0269	0.0284	0.95
P28066	PSA5	PSMA5	Proteasome subunit alpha type-5	0.0811	0.1106	0.73	0.0397	0.0577	0.69
P60900	PSA6	PSMA6	Proteasome subunit alpha type-6	0.0417	0.0427	0.98	0.0416	0.0366	1.14
O14818	PSA7	PSMA7	Proteasome subunit alpha type-7	0.0248	0.0398	0.62	0.0251	0.0241	1.04
P20618	PSB1	PSMB1	Proteasome subunit beta type-1	0.0275	0.0593	0.46	0.0635	0.0689	0.92
P40306	PSB10	PSMB10	Proteasome subunit beta type-10	0.0063	0.0050	1.27	0.0077	0.0080	0.97
P49721	PSB2	PSMB2	Proteasome subunit beta type-2	0.0591	0.0473	1.25	0.0747	0.0565	1.32
P49720	PSB3	PSMB3	Proteasome subunit beta type-3	0.0318	0.0725	0.44	0.0314	0.0405	0.77
P28070	PSB4	PSMB4	Proteasome subunit beta type-4	0.0271	0.0374	0.72	0.0453	0.0532	0.85
P28074	PSB5	PSMB5	Proteasome subunit beta type-5	0.0136	0.0358	0.38	0.0342	0.0289	1.18
P28072	PSB6	PSMB6	Proteasome subunit beta type-6	0.0160	0.0205	0.78	-	0.0120	-/+
Q99436	PSB7	PSMB7	Proteasome subunit beta type-7	0.0139	0.0098	1.42	-	0.0107	-/+
P28062	PSB8	PSMB8	Proteasome subunit beta type-8	0.0294	0.0302	0.97	0.0537	0.0476	1.13
P28065-2	PSB9	PSMB9	Isoform LMP2.S of Proteasome subunit beta type-9	0.0043	0.0265	0.16	-	-	-/-
P28065	PSB9	PSMB9	Proteasome subunit beta type-9	0.0415	0.0303	1.37	0.0200	0.0207	0.97
O75832	PSD10	PSMD10	26S proteasome non-ATPase regulatory subunit 10	0.0077	0.0088	0.87	0.0133	-	+/-
O00231	PSD11	PSMD11	26S proteasome non-ATPase regulatory subunit 11	0.0113	0.0145	0.78	0.0203	0.0263	0.77

O00232	PSD12	PSMD12	26S proteasome non-ATPase regulatory subunit 12	0.0024	0.0018	1.35	0.0237	0.0183	1.30
Q9UNM6	PSD13	PSMD13	26S proteasome non-ATPase regulatory subunit 13	0.0168	0.0128	1.31	0.0376	0.0300	1.25
O00487	PSDE	PSMD14	26S proteasome non-ATPase regulatory subunit 14	0.0160	0.0107	1.50	0.0194	0.0201	0.97
Q99460	PSMD1	PSMD1	26S proteasome non-ATPase regulatory subunit 1	0.0014	0.0006	2.26	0.0224	0.0202	1.11
Q13200-2	PSMD2	PSMD2	Isoform 2 of 26S proteasome non-ATPase regulatory subunit 2	-	0.0002	-/+	-	-	-/-
Q13200	PSMD2	PSMD2	26S proteasome non-ATPase regulatory subunit 2	0.0077	0.0041	1.87	0.0195	0.0229	0.85
O43242	PSMD3	PSMD3	26S proteasome non-ATPase regulatory subunit 3	0.0036	0.0032	1.12	0.0233	0.0202	1.15
P55036	PSMD4	PSMD4	26S proteasome non-ATPase regulatory subunit 4	0.0065	0.0136	0.48	0.0077	-	+/-
Q16401	PSMD5	PSMD5	26S proteasome non-ATPase regulatory subunit 5	0.0022	0.0059	0.36	0.0210	0.0188	1.12
Q15008	PSMD6	PSMD6	26S proteasome non-ATPase regulatory subunit 6	0.0008	0.0018	0.45	0.0212	0.0168	1.26
P51665	PSMD7	PSMD7	26S proteasome non-ATPase regulatory subunit 7	0.0086	0.0097	0.89	0.0130	0.0062	2.08
P48556	PSMD8	PSMD8	26S proteasome non-ATPase regulatory subunit 8	0.0056	0.0042	1.33	0.0079	0.0104	0.76

O00233	PSMD9	PSMD9	26S proteasome non-ATPase regulatory subunit 9	0.0038	0.0017	2.25	-	-	-/-
Q06323	PSME1	PSME1	Proteasome activator complex subunit 1	0.0244	0.0143	1.71	0.0374	0.0342	1.09
Q9UL46	PSME2	PSME2	Proteasome activator complex subunit 2	0.0784	0.0411	1.91	0.0632	0.0531	1.19
P61289	PSME3	PSME3	Proteasome activator complex subunit 3	0.0039	0.0016	2.39	0.0056	0.0058	0.97
Q14997	PSME4	PSME4	Proteasome activator complex subunit 4	-	-	-/-	0.0010	-	+/-
Q92530	PSMF1	PSMF1	Proteasome inhibitor PI31 subunit	0.0037	0.0080	0.46	-	0.0083	-/+
Q9BT73	PSMG3	PSMG3	Proteasome assembly chaperone 3	0.0006	0.0059	0.11	-	-	-/-
Q5JS54	PSMG4	PSMG4	Proteasome assembly chaperone 4	-	0.0018	-/+	-	-	-/-
Q8WXF1	PSPC1	PSPC1	Paraspeckle component 1	0.0010	0.0002	4.72	0.0040	-	+/-
P26599	PTBP1	PTBP1	Polypyrimidine tract-binding protein 1	0.0259	0.0533	0.49	0.0471	0.0346	1.36
O95758	PTBP3	PTBP3	Polypyrimidine tract-binding protein 3	0.0006	0.0006	0.98	0.0059	-	+/-
Q96EY7	PTCD3	PTCD3	Pentatricopeptide repeat domain-containing protein 3, mitochondrial	0.0002	0.0002	1.29	0.0023	-	+/-
Q96BW5	PTER	PTER	Phosphotriesterase-related protein	0.0017	0.0050	0.34	-	0.0055	-/+
Q16647	PTGIS	PTGIS	Prostacyclin synthase	-	-	-/-	0.0130	-	+/-
Q14914	PTGR1	PTGR1	Prostaglandin reductase 1	0.0932	0.1300	0.72	0.1155	0.1241	0.93
Q8N8N7	PTGR2	PTGR2	Prostaglandin reductase 2	0.0047	0.0117	0.40	0.0066	0.0193	0.34
Q8N4Q0	PTGR3	ZADH2	Prostaglandin reductase 3	0.0004	0.0011	0.32	-	0.0093	-/+
Q9Y3E5	PTH2	PTRH2	Peptidyl-tRNA hydrolase 2, mitochondrial	0.0047	0.0097	0.49	0.0065	-	+/-
P06454	PTMA	PTMA	Prothymosin alpha	0.0024	-	+/-	0.0051	-	+/-

P20962	PTMS	PTMS	Parathymosin	-	0.0006	-/+	-	0.0148	-/+
P18031	PTN1	PTPN1	Tyrosine-protein phosphatase non-receptor type 1	0.0017	0.0016	1.03	0.0054	-	+/-
Q06124-1	PTN11	PTPN11	Isoform 2 of Tyrosine-protein phosphatase non-receptor type 11	0.0004	-	+/-	-	-	-/-
Q06124	PTN11	PTPN11	Tyrosine-protein phosphatase non-receptor type 11	0.0008	0.0006	1.52	0.0036	0.0024	1.47
Q05209	PTN12	PTPN12	Tyrosine-protein phosphatase non-receptor type 12	-	-	-/-	0.0046	0.0023	1.99
Q9H3S7	PTN23	PTPN23	Tyrosine-protein phosphatase non-receptor type 23	-	-	-/-	-	0.0013	-/+
P29350	PTN6	PTPN6	Tyrosine-protein phosphatase non-receptor type 6	0.0044	0.0001	33.07	0.0178	0.0099	1.80
Q15257	PTPA	PTPA	Serine/threonine-protein phosphatase 2A activator	0.0023	0.0033	0.69	0.0106	0.0110	0.97
Q8WUK0	PTPM1	PTPMT1	Phosphatidylglycerophosphatase and protein-tyrosine phosphatase 1	0.0008	0.0006	1.30	-	-	-/-
P18433	PTPRA	PTPRA	Receptor-type tyrosine-protein phosphatase alpha	-	-	-/-	0.0022	-	+/-
P08575	PTPRC	PTPRC	Receptor-type tyrosine-protein phosphatase C	0.0025	0.0002	14.94	0.0182	0.0022	8.30
P23469	PTPRE	PTPRE	Receptor-type tyrosine-protein phosphatase epsilon	-	-	-/-	0.0037	-	+/-
P10586	PTPRF	PTPRF	Receptor-type tyrosine-protein phosphatase F	0.0002	0.0001	2.06	-	-	-/-

Q15262	PTPRK	PTPRK	Receptor-type tyrosine-protein phosphatase kappa	0.0001	0.0002	0.51	-	-	-/-
P28827	PTPRM	PTPRM	Receptor-type tyrosine-protein phosphatase mu	0.0000	0.0002	0.20	-	-	-/-
Q03393	PTPS	PTS	6-pyruvoyl tetrahydrobiopterin synthase	-	0.0007	-/+	-	-	-/-
Q6GMV3	PTRD1	PTRHD1	Putative peptidyl-tRNA hydrolase PTRHD1	0.0008	0.0014	0.59	-	-	-/-
P48651	PTSS1	PTDSS1	Phosphatidylserine synthase 1	0.0003	-	+/-	0.0122	0.0047	2.63
P26022	PTX3	PTX3	Pentraxin-related protein PTX3	0.0002	0.0114	0.02	-	-	-/-
Q9UHX1	PUF60	PUF60	Poly(U)-binding-splicing factor PUF60	0.0019	0.0008	2.49	0.0186	0.0042	4.49
Q15397	PUM3	PUM3	Pumilio homolog 3	-	0.0001	-/+	-	-	-/-
P22102	PUR2	GART	Trifunctional purine biosynthetic protein adenosine-3	0.0001	0.0005	0.25	0.0061	0.0081	0.76
O15067	PUR4	PFAS	Phosphoribosylformylglycinamidine synthase	0.0004	0.0010	0.44	0.0044	0.0017	2.49
P22234	PUR6	PAICS	Multifunctional protein ADE2	0.0050	0.0097	0.52	0.0389	0.0446	0.87
P30566	PUR8	ADSL	Adenylosuccinate lyase	0.0005	-	+/-	0.0084	0.0087	0.97
P31939	PUR9	ATIC	Bifunctional purine biosynthesis protein PURH	0.0146	0.0095	1.54	0.0351	0.0350	1.00
Q00577	PURA	PURA	Transcriptional activator protein Pur-alpha	0.0065	0.0055	1.18	0.0047	0.0049	0.97
Q8N142	PURA1	ADSS1	Adenylosuccinate synthetase isozyme 1	0.0020	0.0081	0.25	-	0.0164	-/+
P30520	PURA2	ADSS	Adenylosuccinate synthetase isozyme 2	0.0015	0.0011	1.34	0.0108	0.0042	2.60
Q96QR8	PURB	PURB	Transcriptional activator protein Pur-beta	0.0007	0.0008	0.84	-	-	-/-

Q8N0Z8	PUSL1	PUSL1	tRNA pseudouridine synthase-like 1	-	0.0003	-/+	-	-	-/-
O96011	PX11B	PEX11B	Peroxisomal membrane protein 11B	0.0005	-	+/-	0.0225	-	+/-
Q96HA9	PX11C	PEX11G	Peroxisomal membrane protein 11C	0.0007	-	+/-	0.0081	0.0101	0.80
Q5TGL8	PXDC1	PXDC1	PX domain-containing protein 1	0.0002	0.0018	0.12	-	-	-/-
Q92626	PXDN	PXDN	Peroxidasin homolog	0.0008	-	+/-	0.0020	-	+/-
Q9BRX8	PXL2A	PRXL2A	Peroxiredoxin-like 2A	0.0257	0.0354	0.73	0.0333	0.0367	0.91
Q8TBF2	PXL2B	PRXL2B	Prostamide/prostaglandin F synthase	0.0004	0.0027	0.16	-	-	-/-
Q9NR77	PXMP2	PXMP2	Peroxisomal membrane protein 2	0.0007	0.0033	0.21	0.0057	0.0213	0.27
P11498	PYC	PC	Pyruvate carboxylase, mitochondrial	0.0529	0.0729	0.73	0.0608	0.1140	0.53
P11216	PYGB	PYGB	Glycogen phosphorylase, brain form	0.0258	0.0094	2.76	0.0551	0.0382	1.44
P06737	PYGL	PYGL	Glycogen phosphorylase, liver form	0.0512	0.0950	0.54	0.0640	0.1339	0.48
P11217	PYGM	PYGM	Glycogen phosphorylase, muscle form	0.0004	-	+/-	0.0116	0.0120	0.97
Q9BRP8	PYM1	PYM1	Partner of Y14 and mago	0.0007	0.0004	1.58	-	-	-/-
P27708	PYR1	CAD	CAD protein	0.0008	0.0001	11.80	0.0027	0.0025	1.10
Q02127	PYRD	DHODH	Dihydroorotate dehydrogenase (quinone), mitochondrial	0.0037	0.0023	1.58	0.0062	0.0064	0.97
P17812	PYRG1	CTPS1	CTP synthase 1	0.0006	0.0002	3.46	0.0044	0.0030	1.48
Q9NRF8	PYRG2	CTPS2	CTP synthase 2	-	-	-/-	0.0030	0.0047	0.63
P31930	QCR1	UQCRC1	Cytochrome b-c1 complex subunit 1, mitochondrial	0.0933	0.0404	2.31	0.0817	0.0924	0.88
O14957	QCR10	UQCR11	Cytochrome b-c1 complex subunit 10	-	0.0028	-/+	0.0266	-	+/-

P22695	QCR2	UQCRC2	Cytochrome b-c1 complex subunit 2, mitochondrial	0.1044	0.0653	1.60	0.0704	0.0835	0.84
P07919	QCR6	UQCRH	Cytochrome b-c1 complex subunit 6, mitochondrial	0.0028	0.0060	0.47	-	0.0049	-/+
P14927	QCR7	UQCRB	Cytochrome b-c1 complex subunit 7	0.0064	0.0073	0.87	0.0160	0.0144	1.11
O14949	QCR8	UQCRQ	Cytochrome b-c1 complex subunit 8	0.0124	0.0038	3.30	-	-	-/-
Q9UDW1	QCR9	UQCR10	Cytochrome b-c1 complex subunit 9	-	0.0014	-/+	-	-	-/-
Q96PU8	QKI	QKI	Protein quaking	0.0007	0.0006	1.03	-	-	-/-
Q08257	QOR	CRYZ	Quinone oxidoreductase	0.0736	0.1287	0.57	0.0703	0.1254	0.56
O95825	QORL1	CRYZL1	Quinone oxidoreductase-like protein 1	0.0004	0.0009	0.46	-	-	-/-
Q53FA7	QORX	TP53I3	Quinone oxidoreductase PIG3	0.0039	0.0035	1.12	0.0310	-	+/-
Q16769	QPCT	QPCT	Glutaminyl-peptide cyclotransferase	0.0004	-	+/-	-	-	-/-
O00391	QSOX1	QSOX1	Sulfhydryl oxidase 1	0.0002	-	+/-	0.0023	-	+/-
Q5T6V5	QSPP	C9orf64	Queuosine salvage protein	0.0011	0.0058	0.19	0.0095	0.0127	0.75
Q32P51	RA1L2	HNRNPA1L2	Heterogeneous nuclear ribonucleoprotein A1-like 2	-	0.0079	-/+	-	-	-/-
P61026	RAB10	RAB10	Ras-related protein Rab-10	0.0143	0.0072	1.98	0.0429	0.0149	2.87
Q6IQ22	RAB12	RAB12	Ras-related protein Rab-12	0.0003	-	+/-	-	-	-/-
P51153	RAB13	RAB13	Ras-related protein Rab-13	0.0061	0.0071	0.86	0.0124	-	+/-
P61106	RAB14	RAB14	Ras-related protein Rab-14	0.0816	0.0620	1.32	0.1772	0.0978	1.81
P59190	RAB15	RAB15	Ras-related protein Rab-15	-	0.0008	-/+	-	-	-/-

Q9H0T7	RAB17	RAB17	Ras-related protein Rab-17	-	-	-/-	0.0074	-	+/-
Q9NP72	RAB18	RAB18	Ras-related protein Rab-18	0.0133	0.0136	0.97	0.0283	0.0337	0.84
P62820-2	RAB1A	RAB1A	Isoform 2 of Ras-related protein Rab-1A	-	-	-/-	0.0062	-	+/-
P62820	RAB1A	RAB1A	Ras-related protein Rab-1A	0.0671	0.0365	1.84	0.0890	0.0179	4.98
Q9H0U4	RAB1B	RAB1B	Ras-related protein Rab-1B	0.0355	0.0347	1.02	0.0686	0.0449	1.53
Q9UL25	RAB21	RAB21	Ras-related protein Rab-21	0.0021	0.0019	1.09	0.0248	0.0193	1.29
Q9ULC3	RAB23	RAB23	Ras-related protein Rab-23	0.0034	-	+/-	-	-	-/-
P61019	RAB2A	RAB2A	Ras-related protein Rab-2A	0.0466	0.0332	1.40	0.0594	0.0496	1.20
Q13636	RAB31	RAB31	Ras-related protein Rab-31	0.0008	-	+/-	0.0140	-	+/-
Q13637	RAB32	RAB32	Ras-related protein Rab-32	0.0010	-	+/-	-	0.0077	-/+
Q15286	RAB35	RAB35	Ras-related protein Rab-35	0.0003	0.0012	0.28	0.0138	-	+/-
O95716	RAB3D	RAB3D	Ras-related protein Rab-3D	0.0036	-	+/-	-	-	-/-
Q5JT25	RAB41	RAB41	Ras-related protein Rab-41	0.0007	-	+/-	-	-	-/-
Q86YS6	RAB43	RAB43	Ras-related protein Rab-43	0.0023	0.0027	0.86	0.0133	-	+/-
Q7Z6P3	RAB44	RAB44	Ras-related protein Rab-44	0.0001	-	+/-	-	-	-/-
P20338	RAB4A	RAB4A	Ras-related protein Rab-4A	0.0037	0.0009	4.18	0.0162	-	+/-
P61018	RAB4B	RAB4B	Ras-related protein Rab-4B	-	-	-/-	0.0127	0.0019	6.69
P20339	RAB5A	RAB5A	Ras-related protein Rab-5A	0.0104	0.0083	1.26	0.0382	0.0395	0.97
P61020	RAB5B	RAB5B	Ras-related protein Rab-5B	0.0056	0.0068	0.83	0.0382	0.0395	0.97
P51148	RAB5C	RAB5C	Ras-related protein Rab-5C	0.0392	0.0233	1.68	0.0854	0.0604	1.41

P20340	RAB6A	RAB6A	Ras-related protein Rab-6A	0.0162	0.0095	1.70	0.0224	0.0115	1.94
P51149	RAB7A	RAB7A	Ras-related protein Rab-7a	0.0680	0.0401	1.70	0.0730	0.0558	1.31
O14966	RAB7L	RAB29	Ras-related protein Rab-7L1	-	-	-/-	0.0081	-	+/-
P61006	RAB8A	RAB8A	Ras-related protein Rab-8A	0.0152	0.0130	1.17	0.0292	0.0166	1.76
Q92930	RAB8B	RAB8B	Ras-related protein Rab-8B	0.0058	0.0017	3.32	0.0200	0.0179	1.12
P51151	RAB9A	RAB9A	Ras-related protein Rab-9A	0.0031	0.0010	3.06	-	-	-/-
Q15276	RABE1	RABEP1	Rab GTPase-binding effector protein 1	0.0002	0.0011	0.17	-	-	-/-
Q9H5N1	RABE2	RABEP2	Rab GTPase-binding effector protein 2	-	0.0014	-/+	-	-	-/-
Q7Z6M1	RABEK	RABEPK	Rab9 effector protein with kelch motifs	0.0003	0.0006	0.50	0.0069	-	+/-
Q5HYI8	RABL3	RABL3	Rab-like protein 3	0.0062	0.0027	2.29	-	-	-/-
P63000	RAC1	RAC1	Ras-related C3 botulinum toxin substrate 1	0.0115	0.0168	0.68	0.0361	0.0449	0.80
P15153	RAC2	RAC2	Ras-related C3 botulinum toxin substrate 2	0.0284	0.0200	1.42	0.0429	0.0284	1.51
P60763	RAC3	RAC3	Ras-related C3 botulinum toxin substrate 3	0.0009	-	+/-	-	-	-/-
P63244	RACK1	RACK1	Receptor of activated protein C kinase 1	0.0969	0.0717	1.35	0.1974	0.1459	1.35
O60216	RAD21	RAD21	Double-strand-break repair protein rad21 homolog	0.0001	-	+/-	-	-	-/-
Q92878	RAD50	RAD50	DNA repair protein RAD50	0.0001	-	+/-	0.0008	0.0008	0.97
P35241	RADI	RDX	Radixin	0.0073	0.0067	1.09	0.0278	0.0229	1.21
P78406	RAE1L	RAE1	mRNA export factor	0.0010	0.0018	0.57	0.0087	-	+/-
P46060	RAGP1	RANGAP1	Ran GTPase-activating protein 1	0.0045	0.0030	1.50	0.0067	0.0033	2.01

Q9P0K7	RAI14	RAI14	Ankycorbin	0.0005	-	+/-	-	-	-/-
P11233	RALA	RALA	Ras-related protein Ral-A	0.0044	0.0007	6.77	0.0084	0.0056	1.50
P11234	RALB	RALB	Ras-related protein Ral-B	0.0035	-	+/-	-	-	-/-
Q9UKM9	RALY	RALY	RNA-binding protein Raly	0.0115	0.0076	1.52	0.0103	0.0107	0.97
P62826	RAN	RAN	GTP-binding nuclear protein Ran	0.0601	0.0318	1.89	0.0516	0.0292	1.77
Q9H6Z4	RANB3	RANBP3	Ran-binding protein 3	-	0.0012	-/+	-	-	-/-
P43487	RANG	RANBP1	Ran-specific GTPase-activating protein	0.0032	0.0027	1.20	-	-	-/-
P62834	RAP1A	RAP1A	Ras-related protein Rap-1A	0.0127	0.0286	0.44	0.0568	0.0425	1.34
P61224	RAP1B	RAP1B	Ras-related protein Rap-1b	0.0406	0.0337	1.20	0.0428	0.0201	2.13
P10114	RAP2A	RAP2A	Ras-related protein Rap-2a	0.0120	0.0105	1.14	-	-	-/-
P61225	RAP2B	RAP2B	Ras-related protein Rap-2b	0.0186	0.0165	1.12	0.0862	0.0170	5.07
Q9Y3L5	RAP2C	RAP2C	Ras-related protein Rap-2c	0.0162	0.0340	0.48	0.0371	0.0301	1.23
Q70E73	RAPH1	RAPH1	Ras-associated and pleckstrin homology domains-containing protein 1	-	-	-/-	-	0.0016	-/+
Q99969	RARR2	RARRES2	Retinoic acid receptor responder protein 2	0.0005	0.0013	0.39	0.0081	-	+/-
P01112	RASH	HRAS	GTPase HRas	-	0.0044	-/+	-	-	-/-
P01116	RASK	KRAS	GTPase KRas	-	0.0024	-/+	-	-	-/-
P01116-2	RASK	KRAS	Isoform 2B of GTPase KRas	0.0006	0.0045	0.13	0.0096	0.0223	0.43
Q86YV0	RASL3	RASAL3	RAS protein activator like-3	-	-	-/-	0.0031	-	+/-
P01111	RASN	NRAS	GTPase NRas	0.0266	0.0268	0.99	0.0088	0.0203	0.44

Q8IY67	RAVR1	RAVER1	Ribonucleoprotein PTB-binding 1	0.0004	-	+/-	-	-	-/-
P62491	RB11A	RAB11A	Ras-related protein Rab-11A	0.0734	0.0113	6.51	0.0018	0.0025	0.71
Q15907	RB11B	RAB11B	Ras-related protein Rab-11B	0.0281	0.0243	1.15	0.0561	0.0337	1.67
Q8IXT5	RB12B	RBM12B	RNA-binding protein 12B	-	-	-/-	0.0020	-	+/-
P51159	RB27A	RAB27A	Ras-related protein Rab-27A	0.0065	0.0024	2.69	0.0222	0.0125	1.77
O00194	RB27B	RAB27B	Ras-related protein Rab-27B	0.0043	-	+/-	-	-	-/-
Q9H082	RB33B	RAB33B	Ras-related protein Rab-33B	-	0.0011	-/+	-	-	-/-
Q15042	RB3GP	RAB3GAP1	Rab3 GTPase-activating protein catalytic subunit	0.0002	-	+/-	-	-	-/-
Q8IUD2	RB6I2	ERC1	ELKS/Rab6-interacting/CAST family member 1	0.0002	-	+/-	-	-	-/-
Q09028	RBBP4	RBBP4	Histone-binding protein RBBP4	0.0195	0.0095	2.06	0.0096	0.0099	0.97
Q7Z6E9	RBBP6	RBBP6	E3 ubiquitin-protein ligase RBBP6	-	-	-/-	-	0.0006	-/+
Q16576	RBBP7	RBBP7	Histone-binding protein RBBP7	0.0077	0.0062	1.24	-	-	-/-
O75884	RBBP9	RBBP9	Putative hydrolase RBBP9	0.0158	0.0165	0.96	0.0249	0.0441	0.56
Q9H2M9	RBGPR	RAB3GAP2	Rab3 GTPase-activating protein non-catalytic subunit	0.0000	-	+/-	-	-	-/-
Q9UBK7-2	RBL2A	RABL2A	Isoform 2 of Rab-like protein 2A	0.0003	0.0008	0.31	-	-	-/-
Q9UBK7	RBL2A	RABL2A	Rab-like protein 2A	-	0.0006	-/+	-	-	-/-
P98175	RBM10	RBM10	RNA-binding protein 10	0.0003	-	+/-	-	-	-/-
Q9NTZ6	RBM12	RBM12	RNA-binding protein 12	0.0002	-	+/-	0.0027	-	+/-

Q96PK6	RBM14	RBM14	RNA-binding protein 14	0.0080	0.0043	1.86	0.0109	0.0092	1.18
Q96T37	RBM15	RBM15	RNA-binding protein 15	0.0001	-	+/-	-	-	-/-
Q9NW64	RBM22	RBM22	Pre-mRNA-splicing factor RBM22	0.0004	0.0005	0.71	-	-	-/-
P49756	RBM25	RBM25	RNA-binding protein 25	-	-	-/-	0.0010	-	+/-
P98179	RBM3	RBM3	RNA-binding protein 3	0.0062	0.0028	2.23	0.0098	-	+/-
Q14498	RBM39	RBM39	RNA-binding protein 39	0.0017	-	+/-	0.0065	0.0055	1.18
Q9BWF3	RBM4	RBM4	RNA-binding protein 4	0.0014	0.0012	1.15	-	-	-/-
A0AV96	RBM47	RBM47	RNA-binding protein 47	0.0001	-	+/-	0.0066	-	+/-
Q9BQ04	RBM4B	RBM4B	RNA-binding protein 4B	0.0025	0.0010	2.43	-	-	-/-
P52756	RBM5	RBM5	RNA-binding protein 5	0.0000	-	+/-	-	-	-/-
Q9Y5S9	RBM8A	RBM8A	RNA-binding protein 8A	0.0193	0.0091	2.11	0.0064	-	+/-
P38159	RBMX	RBMX	RNA-binding motif protein, X chromosome	0.0050	0.0028	1.80	0.0057	0.0028	2.00
P49792	RBP2	RANBP2	E3 SUMO-protein ligase RanBP2	0.0001	-	+/-	0.0028	-	+/-
Q92804	RBP56	TAF15	TATA-binding protein-associated factor 2N	0.0006	-	+/-	0.0043	-	+/-
Q93062	RBPMS	RBPMS	RNA-binding protein with multiple splicing	0.0005	-	+/-	-	-	-/-
Q9H477	RBSK	RBKS	Ribokinase	0.0035	0.0073	0.48	-	0.0083	-/+
P62877	RBX1	RBX1	E3 ubiquitin-protein ligase RBX1	0.0052	0.0049	1.06	-	-	-/-
Q9UBF6-4	RBX2	RNF7	Isoform 4 of RING-box protein 2	-	0.0015	-/+	-	-	-/-
P18754	RCC1	RCC1	Regulator of chromosome condensation	0.0052	0.0019	2.79	0.0176	0.0096	1.84
Q9P258	RCC2	RCC2	Protein RCC2	0.0006	0.0002	3.34	0.0077	-	+/-

Q9Y2P8	RCL1	RCL1	RNA 3'-terminal phosphate cyclase-like protein	0.0002	-	+/-	-	-	-/-
Q15293	RCN1	RCN1	Reticulocalbin-1	0.0197	0.0166	1.19	0.0040	0.0020	1.99
Q14257	RCN2	RCN2	Reticulocalbin-2	0.0029	0.0024	1.21	-	-	-/-
Q96D15	RCN3	RCN3	Reticulocalbin-3	0.0015	0.0011	1.29	0.0023	-	+/-
P54725	RD23A	RAD23A	UV excision repair protein RAD23 homolog A	0.0016	0.0105	0.15	-	-	-/-
P54727-2	RD23B	RAD23B	Isoform 2 of UV excision repair protein RAD23 homolog B	0.0021	-	+/-	-	-	-/-
P54727	RD23B	RAD23B	UV excision repair protein RAD23 homolog B	0.0493	0.0275	1.79	0.0224	0.0337	0.66
Q8IZV5	RDH10	RDH10	Retinol dehydrogenase 10	-	-	-/-	0.0087	0.0161	0.54
Q8TC12	RDH11	RDH11	Retinol dehydrogenase 11	0.0187	0.0139	1.35	0.0293	0.0227	1.29
Q8NBN7	RDH13	RDH13	Retinol dehydrogenase 13	0.0005	0.0032	0.17	-	-	-/-
Q9HBH5	RDH14	RDH14	Retinol dehydrogenase 14	0.0014	0.0007	2.06	0.0103	0.0078	1.33
O75452	RDH16	RDH16	Retinol dehydrogenase 16	0.0619	0.0734	0.84	0.0768	0.1168	0.66
Q92781	RDH5	RDH5	Retinol dehydrogenase 5	-	0.0009	-/+	-	0.0143	-/+
P46063	RECQ1	RECQL	ATP-dependent DNA helicase Q1	0.0014	-	+/-	0.0103	0.0050	2.05
O94761	RECQ4	RECQL4	ATP-dependent DNA helicase Q4	0.0001	-	+/-	-	-	-/-
Q13123	RED	IK	Protein Red	0.0001	-	+/-	-	-	-/-
Q00765	REEP5	REEP5	Receptor expression-enhancing protein 5	0.0054	0.0010	5.65	0.0301	0.0268	1.12

Q96HR9-2	REEP6	REEP6	Isoform 2 of Receptor expression-enhancing protein 6	0.0015	0.0086	0.18	-	-	-/-
Q96HR9	REEP6	REEP6	Receptor expression-enhancing protein 6	0.0189	0.0141	1.34	0.0283	0.0422	0.67
P51606	RENBP	RENBP	N-acylglucosamine 2-epimerase	0.0001	-	+/-	0.0063	-	+/-
O75787	RENR	ATP6AP2	Renin receptor	0.0005	-	+/-	-	-	-/-
Q92900	RENT1	UPF1	Regulator of nonsense transcripts 1	0.0023	0.0005	4.76	0.0097	0.0047	2.04
Q96D71	REPS1	REPS1	RalBP1-associated Eps domain-containing protein 1	0.0001	-	+/-	-	-	-/-
O15258	RER1	RER1	Protein RER1	0.0022	0.0005	4.67	0.0405	0.0091	4.43
P09455-2	RET1	RBP1	Isoform 2 of Retinol-binding protein 1	0.0013	0.0013	0.98	-	-	-/-
P09455	RET1	RBP1	Retinol-binding protein 1	0.1216	0.1984	0.61	0.0175	0.1175	0.15
P02753	RET4	RBP4	Retinol-binding protein 4	0.1673	0.0398	4.20	0.1087	0.0608	1.79
P82980	RET5	RBP5	Retinol-binding protein 5	0.0079	0.0319	0.25	-	0.0207	-/+
Q96R05	RET7	RBP7	Retinoid-binding protein 7	0.0003	0.0006	0.50	0.0103	-	+/-
Q9HD89	RETN	RETN	Resistin	0.0177	0.0029	6.07	0.0233	-	+/-
Q86VR2	RETR3	RETREG3	Reticulophagy regulator 3	-	-	-/-	0.0077	0.0080	0.97
Q6NUM9	RETST	RETSAT	All-trans-retinol 13,14-reductase	0.0009	0.0029	0.30	0.0132	0.0148	0.89
P27694	RFA1	RPA1	Replication protein A 70 kDa DNA-binding subunit	0.0010	0.0001	7.03	0.0059	0.0094	0.62
P15927	RFA2	RPA2	Replication protein A 32 kDa subunit	0.0051	0.0046	1.10	-	-	-/-
P35244	RFA3	RPA3	Replication protein A 14 kDa subunit	0.0432	0.0362	1.19	0.0207	-	+/-

P35251	RFC1	RFC1	Replication factor C subunit 1	0.0000	-	+/-	-	-	-/-
P35250	RFC2	RFC2	Replication factor C subunit 2	-	0.0003	-/+	-	-	-/-
P35249	RFC4	RFC4	Replication factor C subunit 4	0.0004	0.0004	1.02	-	-	-/-
O75154	RFIP3	RAB11FIP3	Rab11 family-interacting protein 3	0.0002	-	+/-	-	-	-/-
Q14699	RFTN1	RFTN1	Raftlin	0.0003	0.0002	1.29	-	-	-/-
Q15493	RGN	RGN	Regucalcin	0.0129	0.0383	0.34	0.0097	0.1354	0.07
Q13972	RGRF1	RASGRF1	Ras-specific guanine nucleotide-releasing factor 1	0.0001	-	+/-	-	-	-/-
O14827	RGRF2	RASGRF2	Ras-specific guanine nucleotide-releasing factor 2	0.0001	-	+/-	-	-	-/-
O43665	RGS10	RGS10	Regulator of G-protein signaling 10	0.0005	-	+/-	-	-	-/-
Q15382	RHEB	RHEB	GTP-binding protein Rheb	0.0030	-	+/-	0.0103	-	+/-
Q07960	RHG01	ARHGAP1	Rho GTPase-activating protein 1	0.0194	0.0057	3.44	0.0688	0.0341	2.02
Q96QB1	RHG07	DLC1	Rho GTPase-activating protein 7	0.0005	-	+/-	-	-	-/-
Q68EM7	RHG17	ARHGAP17	Rho GTPase-activating protein 17	0.0002	-	+/-	0.0023	-	+/-
P42331	RHG25	ARHGAP25	Rho GTPase-activating protein 25	0.0002	-	+/-	0.0032	-	+/-
Q9NRY4	RHG35	ARHGAP35	Rho GTPase-activating protein 35	-	-	-/-	0.0011	0.0023	0.48
P61586	RHOA	RHOA	Transforming protein RhoA	0.0403	0.0232	1.74	0.0842	0.0476	1.77
P62745	RHOB	RHOB	Rho-related GTP-binding protein RhoB	0.0021	0.0030	0.69	0.0121	0.0125	0.97
P08134	RHOC	RHOC	Rho-related GTP-binding protein RhoC	0.0158	0.0150	1.05	0.0526	0.0350	1.50
P84095	RHOG	RHOG	Rho-related GTP-binding protein RhoG	0.0066	0.0044	1.50	0.0274	0.0152	1.80

P17081	RHOQ	RHOQ	Rho-related GTP-binding protein RhoQ	0.0006	-	+/-	-	-	-/-
Q9NPQ8	RIC8A	RIC8A	Synembryn-A	-	-	-/-	0.0069	-	+/-
P52758	RIDA	RIDA	2-iminobutanoate/2-iminopropanoate deaminase	0.0923	0.3308	0.28	0.1412	0.2035	0.69
Q969G6	RIFK	RFK	Riboflavin kinase	-	0.0009	-/+	-	-	-/-
Q06587	RING1	RING1	E3 ubiquitin-protein ligase RING1	-	0.0010	-/+	-	-	-/-
P13489	RINI	RNH1	Ribonuclease inhibitor	0.0353	0.0337	1.05	0.0863	0.0798	1.08
Q9H6W3	RIOX1	RIOX1	Ribosomal oxygenase 1	0.0012	-	+/-	0.0029	-	+/-
Q13546	RIPK1	RIPK1	Receptor-interacting serine/threonine-protein kinase 1	0.0002	-	+/-	-	-	-/-
Q7LG56	RIR2B	RRM2B	Ribonucleoside-diphosphate reductase subunit M2 B	-	-	-/-	0.0049	-	+/-
Q9HB40	RISC	SCPEP1	Retinoid-inducible serine carboxypeptidase	0.0057	0.0035	1.64	0.0098	-	+/-
P27635	RL10	RPL10	60S ribosomal protein L10	0.0049	0.0034	1.45	0.0304	0.0221	1.38
P62906	RL10A	RPL10A	60S ribosomal protein L10a	0.0172	0.0188	0.92	0.0425	0.0293	1.45
P62913	RL11	RPL11	60S ribosomal protein L11	0.0172	0.0226	0.76	0.0307	0.0159	1.92
P30050	RL12	RPL12	60S ribosomal protein L12	0.0231	0.0264	0.87	0.0363	0.0356	1.02
P26373	RL13	RPL13	60S ribosomal protein L13	0.0052	0.0023	2.20	0.0268	0.0248	1.08
P40429	RL13A	RPL13A	60S ribosomal protein L13a	0.0003	0.0007	0.39	0.0165	0.0140	1.18
P50914	RL14	RPL14	60S ribosomal protein L14	0.0056	0.0025	2.18	0.0152	0.0147	1.03
P61313	RL15	RPL15	60S ribosomal protein L15	0.0019	0.0005	3.71	0.0192	0.0148	1.30

P18621	RL17	RPL17	60S ribosomal protein L17	0.0040	0.0013	2.97	0.0133	0.0107	1.25
Q07020	RL18	RPL18	60S ribosomal protein L18	0.0091	0.0045	2.04	0.0091	0.0097	0.94
Q02543	RL18A	RPL18A	60S ribosomal protein L18a	0.0039	0.0003	13.67	0.0186	0.0120	1.55
P84098	RL19	RPL19	60S ribosomal protein L19	0.0004	-	+/-	0.0058	0.0056	1.04
O76021	RL1D1	RSL1D1	Ribosomal L1 domain-containing protein 1	0.0001	0.0001	0.67	0.0046	0.0038	1.22
P46778	RL21	RPL21	60S ribosomal protein L21	0.0019	0.0028	0.69	0.0141	0.0125	1.13
P35268	RL22	RPL22	60S ribosomal protein L22	0.0214	0.0263	0.81	0.0309	0.0175	1.76
Q6P5R6	RL22L	RPL22L1	60S ribosomal protein L22-like 1	0.0011	0.0004	2.55	0.0081	-	+/-
P62829	RL23	RPL23	60S ribosomal protein L23	0.0147	0.0191	0.77	0.0504	0.0307	1.64
P62750	RL23A	RPL23A	60S ribosomal protein L23a	0.0049	0.0035	1.39	0.0209	0.0115	1.82
P83731	RL24	RPL24	60S ribosomal protein L24	0.0012	0.0002	5.13	0.0154	0.0171	0.90
P61254	RL26	RPL26	60S ribosomal protein L26	0.0042	0.0029	1.41	0.0171	0.0076	2.26
P61353	RL27	RPL27	60S ribosomal protein L27	0.0125	0.0056	2.23	0.0342	0.0252	1.36
P46776	RL27A	RPL27A	60S ribosomal protein L27a	0.0031	0.0006	4.93	0.0252	0.0210	1.20
P46779	RL28	RPL28	60S ribosomal protein L28	0.0024	0.0009	2.77	0.0064	0.0066	0.97
P47914	RL29	RPL29	60S ribosomal protein L29	-	-	-/-	0.0044	-	+/-
P39023	RL3	RPL3	60S ribosomal protein L3	0.0041	0.0026	1.61	0.0339	0.0310	1.09
P62888	RL30	RPL30	60S ribosomal protein L30	0.0209	0.0155	1.35	0.0736	0.0769	0.96
P62899	RL31	RPL31	60S ribosomal protein L31	0.0027	0.0024	1.12	0.0114	-	+/-
P62910	RL32	RPL32	60S ribosomal protein L32	0.0007	-	+/-	0.0222	0.0186	1.19

P49207	RL34	RPL34	60S ribosomal protein L34	0.0008	0.0003	2.37	0.0079	-	+/-
P42766	RL35	RPL35	60S ribosomal protein L35	0.0006	0.0003	2.53	0.0098	0.0050	1.95
P18077	RL35A	RPL35A	60S ribosomal protein L35a	0.0040	0.0054	0.74	0.0133	-	+/-
Q9Y3U8	RL36	RPL36	60S ribosomal protein L36	0.0005	-	+/-	0.0142	0.0062	2.30
P61513	RL37A	RPL37A	60S ribosomal protein L37a	0.0017	-	+/-	0.0140	0.0144	0.97
P63173	RL38	RPL38	60S ribosomal protein L38	0.0054	0.0044	1.24	0.0219	0.0161	1.37
P62891	RL39	RPL39	60S ribosomal protein L39	-	-	-/-	0.0116	-	+/-
Q96EH5	RL39L	RPL39L	60S ribosomal protein L39-like	0.0064	-	+/-	-	-	-/-
P36578	RL4	RPL4	60S ribosomal protein L4	0.0026	0.0029	0.89	0.0264	0.0262	1.01
P46777	RL5	RPL5	60S ribosomal protein L5	0.0104	0.0068	1.54	0.0276	0.0277	1.00
Q02878	RL6	RPL6	60S ribosomal protein L6	0.0063	0.0039	1.64	0.0196	0.0157	1.25
P18124	RL7	RPL7	60S ribosomal protein L7	0.0059	0.0034	1.72	0.0328	0.0288	1.14
P62424	RL7A	RPL7A	60S ribosomal protein L7a	0.0056	0.0082	0.68	0.0337	0.0340	0.99
P62917	RL8	RPL8	60S ribosomal protein L8	0.0044	0.0011	4.10	0.0204	0.0134	1.52
P32969	RL9	RPL9	60S ribosomal protein L9	0.0223	0.0352	0.63	0.0251	0.0260	0.97
P05388	RLA0	RPLP0	60S acidic ribosomal protein P0	0.1039	0.0697	1.49	0.0611	0.0517	1.18
P05386	RLA1	RPLP1	60S acidic ribosomal protein P1	0.0057	0.0127	0.45	0.0241	0.0073	3.30
P05387	RLA2	RPLP2	60S acidic ribosomal protein P2	0.0096	0.0177	0.54	0.0234	0.0040	5.87
Q13129	RLF	RLF	Zinc finger protein Rlf	-	0.0000	-/+	-	-	-/-
Q9BYD6	RM01	MRPL1	39S ribosomal protein L1, mitochondrial	0.0015	0.0013	1.13	0.0057	-	+/-

Q5T653	RM02	MRPL2	39S ribosomal protein L2, mitochondrial	-	-	-/-	0.0066	-	+/-
P09001	RM03	MRPL3	39S ribosomal protein L3, mitochondrial	0.0005	-	+/-	-	-	-/-
Q9BYD3	RM04	MRPL4	39S ribosomal protein L4, mitochondrial	0.0005	0.0003	1.73	-	-	-/-
Q9BYD2	RM09	MRPL9	39S ribosomal protein L9, mitochondrial	0.0013	-	+/-	0.0044	-	+/-
Q7Z7H8	RM10	MRPL10	39S ribosomal protein L10, mitochondrial	0.0002	0.0003	0.54	-	-	-/-
Q9Y3B7	RM11	MRPL11	39S ribosomal protein L11, mitochondrial	0.0048	0.0092	0.53	0.0066	-	+/-
P52815	RM12	MRPL12	39S ribosomal protein L12, mitochondrial	0.0120	0.0031	3.90	-	-	-/-
Q9BYD1	RM13	MRPL13	39S ribosomal protein L13, mitochondrial	0.0079	0.0032	2.48	0.0088	-	+/-
Q9P015	RM15	MRPL15	39S ribosomal protein L15, mitochondrial	0.0006	-	+/-	0.0065	-	+/-
Q9NX20	RM16	MRPL16	39S ribosomal protein L16, mitochondrial	0.0021	0.0005	4.22	-	-	-/-
Q9NRX2	RM17	MRPL17	39S ribosomal protein L17, mitochondrial	0.0011	0.0006	1.96	-	-	-/-
Q9H0U6	RM18	MRPL18	39S ribosomal protein L18, mitochondrial	0.0029	0.0033	0.89	-	-	-/-
P49406	RM19	MRPL19	39S ribosomal protein L19, mitochondrial	0.0021	0.0039	0.53	0.0073	-	+/-
Q7Z2W9	RM21	MRPL21	39S ribosomal protein L21, mitochondrial	0.0020	-	+/-	-	-	-/-
Q9NWU5	RM22	MRPL22	39S ribosomal protein L22, mitochondrial	0.0020	0.0034	0.60	0.0087	-	+/-
Q16540	RM23	MRPL23	39S ribosomal protein L23, mitochondrial	0.0009	0.0010	0.93	-	-	-/-
Q96A35	RM24	MRPL24	39S ribosomal protein L24, mitochondrial	0.0039	0.0022	1.77	0.0058	-	+/-
Q13084	RM28	MRPL28	39S ribosomal protein L28, mitochondrial	0.0007	0.0004	1.78	-	-	-/-
Q9BZE1	RM37	MRPL37	39S ribosomal protein L37, mitochondrial	0.0015	-	+/-	-	-	-/-
Q96DV4	RM38	MRPL38	39S ribosomal protein L38, mitochondrial	0.0003	0.0006	0.48	-	-	-/-

Q9NYK5	RM39	MRPL39	39S ribosomal protein L39, mitochondrial	0.0016	0.0041	0.39	0.0043	-	+/-
Q9NQ50	RM40	MRPL40	39S ribosomal protein L40, mitochondrial	0.0004	0.0006	0.67	-	-	-/-
Q8IXM3	RM41	MRPL41	39S ribosomal protein L41, mitochondrial	0.0010	-	+/-	-	-	-/-
Q8N983	RM43	MRPL43	39S ribosomal protein L43, mitochondrial	0.0034	0.0029	1.16	-	-	-/-
Q9H9J2	RM44	MRPL44	39S ribosomal protein L44, mitochondrial	0.0028	0.0016	1.73	-	-	-/-
Q9BRJ2	RM45	MRPL45	39S ribosomal protein L45, mitochondrial	0.0011	0.0002	4.64	-	-	-/-
Q9H2W6	RM46	MRPL46	39S ribosomal protein L46, mitochondrial	0.0041	0.0044	0.94	-	-	-/-
Q96GC5	RM48	MRPL48	39S ribosomal protein L48, mitochondrial	0.0053	0.0025	2.16	-	-	-/-
Q13405	RM49	MRPL49	39S ribosomal protein L49, mitochondrial	0.0031	0.0054	0.57	-	-	-/-
Q8N5N7	RM50	MRPL50	39S ribosomal protein L50, mitochondrial	0.0006	-	+/-	-	-	-/-
Q96EL3	RM53	MRPL53	39S ribosomal protein L53, mitochondrial	0.0011	0.0015	0.73	-	-	-/-
Q96DB5	RMD1	RMDN1	Regulator of microtubule dynamics protein 1	0.0058	0.0066	0.88	0.0069	0.0072	0.97
Q96LZ7	RMD2	RMDN2	Regulator of microtubule dynamics protein 2	0.0031	0.0041	0.75	0.0159	0.0219	0.73
Q96TC7	RMD3	RMDN3	Regulator of microtubule dynamics protein 3	0.0128	0.0060	2.13	0.0105	0.0070	1.51
Q9NWS8	RMND1	RMND1	Required for meiotic nuclear division protein 1 homolog	-	-	-/-	-	0.0033	-/+
Q96E39	RMXL1	RBMXL1	RNA binding motif protein, X-linked-like-1	0.0014	0.0021	0.66	-	-	-/-
O75526	RMXL2	RBMXL2	RNA-binding motif protein, X-linked-like-2	-	0.0004	-/+	-	-	-/-
Q9Y508	RN114	RNF114	E3 ubiquitin-protein ligase RNF114	0.0005	0.0006	0.90	-	-	-/-
Q9P0P0	RN181	RNF181	E3 ubiquitin-protein ligase RNF181	-	0.0015	-/+	-	-	-/-

Q63HN8	RN213	RNF213	E3 ubiquitin-protein ligase RNF213	-	0.0000	-/+	0.0027	0.0008	3.25
P10153	RNAS2	RNASE2	Non-secretory ribonuclease	0.0006	0.0018	0.33	-	-	-/-
Q92730	RND1	RND1	Rho-related GTP-binding protein Rho6	0.0032	-	+/-	-	-	-/-
O75792	RNH2A	RNASEH2A	Ribonuclease H2 subunit A	0.0002	0.0011	0.14	-	-	-/-
Q8TDP1	RNH2C	RNASEH2C	Ribonuclease H2 subunit C	0.0010	0.0031	0.31	-	-	-/-
Q15287	RNPS1	RNPS1	RNA-binding protein with serine-rich domain 1	0.0001	-	+/-	-	-	-/-
O00584	RNT2	RNASET2	Ribonuclease T2	0.0011	0.0028	0.41	0.0062	-	+/-
Q9BQ52	RNZ2	ELAC2	Zinc phosphodiesterase ELAC protein 2	-	-	-/-	-	0.0021	-/+
P19474	RO52	TRIM21	E3 ubiquitin-protein ligase TRIM21	0.0010	0.0009	1.13	0.0052	-	+/-
P10155	RO60	RO60	60 kDa SS-A/Ro ribonucleoprotein	0.0034	0.0007	4.73	0.0137	0.0074	1.86
Q13151	ROA0	HNRNPA0	Heterogeneous nuclear ribonucleoprotein A0	0.0084	0.0069	1.22	0.0124	0.0093	1.34
P09651-2	ROA1	HNRNPA1	Isoform A1-A of Heterogeneous nuclear ribonucleoprotein A1	0.0001	-	+/-	-	-	-/-
P09651	ROA1	HNRNPA1	Heterogeneous nuclear ribonucleoprotein A1	0.0952	0.0603	1.58	0.1328	0.0617	2.15
P22626	ROA2	HNRNPA2B1	Heterogeneous nuclear ribonucleoproteins A2/B1	0.1610	0.1169	1.38	0.1216	0.0815	1.49
P51991-2	ROA3	HNRNPA3	Isoform 2 of Heterogeneous nuclear ribonucleoprotein A3	0.0052	0.0064	0.81	-	-	-/-
P51991	ROA3	HNRNPA3	Heterogeneous nuclear ribonucleoprotein A3	0.0492	0.0356	1.38	0.0718	0.0440	1.63
Q99729	ROAA	HNRNPAB	Heterogeneous nuclear ribonucleoprotein A/B	0.0043	0.0010	4.09	-	-	-/-

Q99729-2	ROAA	HNRNPAB	Isoform 2 of Heterogeneous nuclear ribonucleoprotein A/B	0.0152	0.0093	1.64	0.0173	0.0101	1.71
Q13464	ROCK1	ROCK1	Rho-associated protein kinase 1	-	-	-/-	-	0.0014	-/+
O75116	ROCK2	ROCK2	Rho-associated protein kinase 2	0.0000	-	+/-	0.0009	0.0014	0.64
Q8N5L8	RP25L	RPP25L	Ribonuclease P protein subunit p25-like protein	0.0003	-	+/-	-	-	-/-
P19388	RPAB1	POLR2E	DNA-directed RNA polymerases I, II, and III subunit RPABC1	0.0069	0.0026	2.64	-	-	-/-
P52434	RPAB3	POLR2H	DNA-directed RNA polymerases I, II, and III subunit RPABC3	0.0126	0.0055	2.28	-	-	-/-
P62875	RPAB5	POLR2L	DNA-directed RNA polymerases I, II, and III subunit RPABC5	-	0.0015	-/+	-	-	-/-
O15160	RPAC1	POLR1C	DNA-directed RNA polymerases I and III subunit RPAC1	0.0004	0.0013	0.29	-	-	-/-
P0DPB6	RPAC2	POLR1D	DNA-directed RNA polymerases I and III subunit RPAC2	0.0003	0.0007	0.50	-	-	-/-
P52435	RPB11	POLR2J	DNA-directed RNA polymerase II subunit RPB11-a	0.0009	0.0013	0.72	-	-	-/-
P30876	RPB2	POLR2B	DNA-directed RNA polymerase II subunit RPB2	0.0001	-	+/-	0.0013	-	+/-
P19387	RPB3	POLR2C	DNA-directed RNA polymerase II subunit RPB3	0.0149	0.0078	1.91	-	-	-/-
O15514	RPB4	POLR2D	DNA-directed RNA polymerase II subunit RPB4	0.0032	0.0016	1.99	-	-	-/-

P62487	RPB7	POLR2G	DNA-directed RNA polymerase II subunit RPB7	0.0012	0.0042	0.28	-	-	-/-
P36954	RPB9	POLR2I	DNA-directed RNA polymerase II subunit RPB9	-	0.0107	-/+	-	-	-/-
Q9Y535	RPC8	POLR3H	DNA-directed RNA polymerase III subunit RPC8	0.0003	-	+/-	-	-	-/-
Q9H7B2	RPF2	RPF2	Ribosome production factor 2 homolog	-	-	-/-	0.0047	-	+/-
P49247	RPIA	RPIA	Ribose-5-phosphate isomerase	0.0006	0.0005	1.11	0.0060	-	+/-
P04843	RPN1	RPN1	Dolichyl-diphosphooligosaccharide--protein glycosyltransferase subunit 1	0.0911	0.0399	2.28	0.0850	0.0807	1.05
P04844	RPN2	RPN2	Dolichyl-diphosphooligosaccharide--protein glycosyltransferase subunit 2	0.0613	0.0225	2.72	0.0986	0.0688	1.43
P78346	RPP30	RPP30	Ribonuclease P protein subunit p30	-	0.0015	-/+	-	-	-/-
P78345	RPP38	RPP38	Ribonuclease P protein subunit p38	0.0001	-	+/-	-	-	-/-
Q9NQG5	RPR1B	RPRD1B	Regulation of nuclear pre-mRNA domain-containing protein 1B	0.0036	0.0047	0.77	-	0.0042	-/+
Q7L523	RRAGA	RRAGA	Ras-related GTP-binding protein A	-	-	-/-	0.0049	-	+/-
P10301	RRAS	RRAS	Ras-related protein R-Ras	0.0072	0.0057	1.26	0.0178	0.0184	0.97
P62070	RRAS2	RRAS2	Ras-related protein R-Ras2	0.0021	0.0020	1.05	-	-	-/-
Q9P2E9	RRBP1	RRBP1	Ribosome-binding protein 1	0.0056	0.0041	1.35	0.0147	0.0202	0.73
Q969S9	RRF2M	GFM2	Ribosome-releasing factor 2, mitochondrial	-	0.0002	-/+	-	-	-/-
Q96E11	RRFM	MRRF	Ribosome-recycling factor, mitochondrial	0.0008	0.0018	0.44	-	-	-/-

Q9Y2L1	RRP44	DIS3	Exosome complex exonuclease RRP44	0.0002	-	+/-	0.0030	-	+/-
P46783	RS10	RPS10	40S ribosomal protein S10	0.0078	0.0064	1.23	0.0229	0.0162	1.41
P62280	RS11	RPS11	40S ribosomal protein S11	0.0145	0.0017	8.40	0.0296	0.0286	1.04
P25398	RS12	RPS12	40S ribosomal protein S12	0.0321	0.0326	0.99	0.0613	0.0488	1.26
P62277	RS13	RPS13	40S ribosomal protein S13	0.0026	0.0029	0.90	0.0188	0.0096	1.96
P62263	RS14	RPS14	40S ribosomal protein S14	0.0266	0.0295	0.90	0.0480	0.0383	1.25
P62841	RS15	RPS15	40S ribosomal protein S15	0.0026	0.0063	0.41	0.0161	0.0038	4.23
P62244	RS15A	RPS15A	40S ribosomal protein S15a	0.0382	0.0586	0.65	0.0370	0.0488	0.76
P62249	RS16	RPS16	40S ribosomal protein S16	0.0213	0.0335	0.63	0.0329	0.0338	0.97
P08708	RS17	RPS17	40S ribosomal protein S17	0.0179	0.0414	0.43	0.0655	0.0496	1.32
P62269	RS18	RPS18	40S ribosomal protein S18	0.0167	0.0195	0.85	0.0314	0.0267	1.18
P39019	RS19	RPS19	40S ribosomal protein S19	0.0057	0.0013	4.31	0.0239	0.0197	1.21
P15880	RS2	RPS2	40S ribosomal protein S2	0.0370	0.0216	1.72	0.0397	0.0388	1.02
P60866	RS20	RPS20	40S ribosomal protein S20	0.0098	0.0143	0.69	0.0173	0.0138	1.26
P63220	RS21	RPS21	40S ribosomal protein S21	0.0272	0.0536	0.51	0.0280	0.0289	0.97
P62266	RS23	RPS23	40S ribosomal protein S23	0.0041	0.0015	2.76	0.0215	0.0223	0.97
P62847	RS24	RPS24	40S ribosomal protein S24	0.0019	0.0062	0.31	0.0199	0.0120	1.66
P62851	RS25	RPS25	40S ribosomal protein S25	0.0058	0.0043	1.36	0.0090	0.0093	0.97
P62854	RS26	RPS26	40S ribosomal protein S26	0.0012	0.0023	0.53	0.0116	0.0120	0.97
P42677	RS27	RPS27	40S ribosomal protein S27	0.0028	0.0099	0.28	-	-	-/-

P62979	RS27A	RPS27A	Ubiquitin-40S ribosomal protein S27a	0.0003	-	+/-	0.0357	0.0245	1.46
Q71UM5	RS27L	RPS27L	40S ribosomal protein S27-like	0.0034	0.0129	0.26	0.0215	0.0321	0.67
P62857	RS28	RPS28	40S ribosomal protein S28	0.0149	0.0013	11.20	0.0155	-	+/-
P23396	RS3	RPS3	40S ribosomal protein S3	0.1182	0.1137	1.04	0.1079	0.1063	1.01
P62861	RS30	FAU	40S ribosomal protein S30	0.0014	-	+/-	-	-	-/-
P61247	RS3A	RPS3A	40S ribosomal protein S3a	0.0256	0.0195	1.32	0.0544	0.0365	1.49
P62701	RS4X	RPS4X	40S ribosomal protein S4, X isoform	0.0279	0.0246	1.14	0.0478	0.0418	1.15
P22090	RS4Y1	RPS4Y1	40S ribosomal protein S4, Y isoform 1	0.0052	0.0036	1.47	0.0252	0.0261	0.97
P46782	RS5	RPS5	40S ribosomal protein S5	0.0114	0.0020	5.63	0.0464	0.0223	2.08
P62753	RS6	RPS6	40S ribosomal protein S6	0.0039	0.0021	1.86	0.0167	0.0190	0.88
P62081	RS7	RPS7	40S ribosomal protein S7	0.0257	0.0358	0.72	0.0465	0.0436	1.07
P62241	RS8	RPS8	40S ribosomal protein S8	0.0065	0.0030	2.16	0.0329	0.0269	1.22
P46781	RS9	RPS9	40S ribosomal protein S9	0.0220	0.0113	1.95	0.0265	0.0121	2.19
Q96T23	RSF1	RSF1	Remodeling and spacing factor 1	0.0074	0.0001	90.21	-	-	-/-
P14678	RSMB	SNRPB	Small nuclear ribonucleoprotein-associated proteins B and B'	0.0044	0.0069	0.63	0.0169	0.0175	0.97
P08865	RSSA	RPSA	40S ribosomal protein SA	0.1970	0.1244	1.58	0.1003	0.0890	1.13
Q15404	RSU1	RSU1	Ras suppressor protein 1	0.0071	0.0143	0.49	0.0096	0.0099	0.97
Q9Y399	RT02	MRPS2	28S ribosomal protein S2, mitochondrial	0.0009	0.0007	1.31	-	-	-/-
P82932	RT06	MRPS6	28S ribosomal protein S6, mitochondrial	-	0.0006	-/+	-	-	-/-

Q9Y2R9	RT07	MRPS7	28S ribosomal protein S7, mitochondrial	0.0014	0.0005	2.82	-	-	-/-
P82933	RT09	MRPS9	28S ribosomal protein S9, mitochondrial	0.0003	0.0010	0.30	0.0081	0.0032	2.56
P82664	RT10	MRPS10	28S ribosomal protein S10, mitochondrial	0.0031	0.0015	2.04	-	-	-/-
P82912	RT11	MRPS11	28S ribosomal protein S11, mitochondrial	0.0006	0.0006	0.93	-	-	-/-
Q9Y3D3	RT16	MRPS16	28S ribosomal protein S16, mitochondrial	0.0003	0.0006	0.54	-	-	-/-
Q9Y2R5	RT17	MRPS17	28S ribosomal protein S17, mitochondrial	0.0009	0.0022	0.43	-	-	-/-
Q9Y676	RT18B	MRPS18B	28S ribosomal protein S18b, mitochondrial	0.0007	0.0005	1.53	-	-	-/-
P82921	RT21	MRPS21	28S ribosomal protein S21, mitochondrial	-	-	-/-	0.0103	-	+/-
P82650	RT22	MRPS22	28S ribosomal protein S22, mitochondrial	0.0095	0.0081	1.17	0.0086	-	+/-
Q9Y3D9	RT23	MRPS23	28S ribosomal protein S23, mitochondrial	0.0046	0.0042	1.10	0.0155	-	+/-
Q96EL2	RT24	MRPS24	28S ribosomal protein S24, mitochondrial	0.0003	-	+/-	-	-	-/-
P82663	RT25	MRPS25	28S ribosomal protein S25, mitochondrial	0.0043	0.0046	0.94	0.0071	-	+/-
Q9BYN8	RT26	MRPS26	28S ribosomal protein S26, mitochondrial	0.0004	-	+/-	-	-	-/-
Q92552	RT27	MRPS27	28S ribosomal protein S27, mitochondrial	0.0010	0.0011	0.91	0.0039	-	+/-
Q9Y2Q9	RT28	MRPS28	28S ribosomal protein S28, mitochondrial	0.0044	0.0049	0.89	-	-	-/-
P51398	RT29	DAP3	28S ribosomal protein S29, mitochondrial	0.0009	0.0006	1.42	0.0059	0.0040	1.48
Q92665	RT31	MRPS31	28S ribosomal protein S31, mitochondrial	0.0008	0.0002	3.59	-	-	-/-
P82930	RT34	MRPS34	28S ribosomal protein S34, mitochondrial	0.0009	0.0006	1.68	-	-	-/-
P82673	RT35	MRPS35	28S ribosomal protein S35, mitochondrial	0.0003	-	+/-	-	-	-/-

Q8WWV3	RT4I1	RTN4IP1	Reticulon-4-interacting protein 1, mitochondrial	0.0003	0.0008	0.44	-	-	-/-
O00442	RTCA	RTCA	RNA 3'-terminal phosphate cyclase	0.0007	0.0009	0.83	0.0106	0.0110	0.97
Q9Y3I0	RTCB	RTCB	tRNA-splicing ligase RtcB homolog	0.0128	0.0032	3.96	0.0296	0.0226	1.31
Q9BY42	RTF2	RTF2	Replication termination factor 2	0.0002	-	+/-	-	-	-/-
A6ZKI3	RTL8C	RTL8C	Retrotransposon Gag-like protein 8C	0.0005	-	+/-	-	-	-/-
Q16799	RTN1	RTN1	Reticulon-1	-	-	-/-	0.0031	-	+/-
O95197	RTN3	RTN3	Reticulon-3	0.0001	-	+/-	0.0030	0.0020	1.47
O95197-3	RTN3	RTN3	Isoform 3 of Reticulon-3	-	-	-/-	0.0093	-	+/-
Q9NQC3-2	RTN4	RTN4	Isoform B of Reticulon-4	0.0020	-	+/-	0.0233	0.0293	0.79
Q9NQC3	RTN4	RTN4	Reticulon-4	0.0027	0.0018	1.50	0.0019	0.0076	0.25
Q9NQC3-3	RTN4	RTN4	Isoform C of Reticulon-4	-	-	-/-	0.0178	0.0304	0.58
Q9Y224	RTRAF	RTRAF	RNA transcription, translation and transport factor protein	0.0037	0.0021	1.76	-	-	-/-
P08621	RU17	SNRNP70	U1 small nuclear ribonucleoprotein 70 kDa	0.0007	-	+/-	0.0025	-	+/-
P09661	RU2A	SNRPA1	U2 small nuclear ribonucleoprotein A'	0.0064	0.0058	1.11	0.0073	0.0049	1.49
P08579	RU2B	SNRPB2	U2 small nuclear ribonucleoprotein B''	0.0026	0.0015	1.70	0.0064	-	+/-
Q96T51	RUFY1	RUFY1	RUN and FYVE domain-containing protein 1	-	-	-/-	0.0020	-	+/-
Q8WXA3	RUFY2	RUFY2	RUN and FYVE domain-containing protein 2	0.0001	-	+/-	-	-	-/-
Q9Y265	RUVB1	RUVBL1	RuvB-like 1	0.0093	0.0039	2.39	0.0175	0.0072	2.42
Q9Y230	RUVB2	RUVBL2	RuvB-like 2	0.0173	0.0057	3.05	0.0143	0.0087	1.64

P62304	RUXE	SNRPE	Small nuclear ribonucleoprotein E	0.0029	0.0132	0.22	-	0.0161	-/+
P62306	RUXF	SNRPF	Small nuclear ribonucleoprotein F	0.0835	0.1265	0.66	0.0671	-	+/-
P62308	RUXG	SNRPG	Small nuclear ribonucleoprotein G	0.0028	0.0165	0.17	-	-	-/-
Q8N488	RYBP	RYBP	RING1 and YY1-binding protein	-	0.0003	-/+	-	-	-/-
P25815	S100P	S100P	Protein S100-P	0.0145	0.0011	13.43	0.0085	-	+/-
P26447	S10A4	S100A4	Protein S100-A4	0.0174	0.0110	1.59	0.0420	0.0138	3.05
P06703	S10A6	S100A6	Protein S100-A6	0.0069	0.0099	0.70	0.0069	0.0045	1.51
P31151	S10A7	S100A7	Protein S100-A7	0.0039	0.0176	0.22	-	-	-/-
P05109	S10A8	S100A8	Protein S100-A8	0.1907	0.0266	7.16	0.2348	0.0422	5.57
P06702	S10A9	S100A9	Protein S100-A9	0.1822	0.0534	3.41	0.3005	0.0155	19.36
P60903	S10AA	S100A10	Protein S100-A10	0.0721	0.1198	0.60	0.0155	0.0161	0.97
P31949	S10AB	S100A11	Protein S100-A11	0.0264	0.0128	2.07	0.0317	0.0041	7.63
P80511	S10AC	S100A12	Protein S100-A12	0.0257	-	+/-	0.0263	-	+/-
Q99584	S10AD	S100A13	Protein S100-A13	0.0033	0.0010	3.35	-	-	-/-
Q9HCY8	S10AE	S100A14	Protein S100-A14	-	0.0021	-/+	-	-	-/-
Q96FQ6	S10AG	S100A16	Protein S100-A16	0.0030	0.0008	3.67	-	-	-/-
P55011	S12A2	SLC12A2	Solute carrier family 12 member 2	-	-	-/-	0.0019	-	+/-
Q9BXP2	S12A9	SLC12A9	Solute carrier family 12 member 9	-	-	-/-	0.0067	-	+/-
Q86YT5	S13A5	SLC13A5	Solute carrier family 13 member 5	-	-	-/-	0.0069	-	+/-
O76054	S14L2	SEC14L2	SEC14-like protein 2	0.0223	0.0498	0.45	0.0244	0.0735	0.33

Q9UDX3	S14L4	SEC14L4	SEC14-like protein 4	0.0009	0.0006	1.43	-	-	-/-
O15245	S22A1	SLC22A1	Solute carrier family 22 member 1	-	0.0005	-/+	-	0.0078	-/+
Q9Y694	S22A7	SLC22A7	Solute carrier family 22 member 7	-	0.0002	-/+	-	-	-/-
Q8IVM8	S22A9	SLC22A9	Solute carrier family 22 member 9	-	-	-/-	-	0.0070	-/+
Q96BI1	S22AI	SLC22A18	Solute carrier family 22 member 18	0.0023	-	+/-	0.0071	-	+/-
Q9Y6Y8	S23IP	SEC23IP	SEC23-interacting protein	0.0012	0.0018	0.65	0.0092	0.0045	2.04
Q8TBP6	S2540	SLC25A40	Solute carrier family 25 member 40	-	-	-/-	0.0051	-	+/-
Q86VD7	S2542	SLC25A42	Mitochondrial coenzyme A transporter SLC25A42	-	0.0016	-/+	0.0047	0.0165	0.29
Q6PCB7	S27A1	SLC27A1	Long-chain fatty acid transport protein 1	0.0009	-	+/-	0.0057	-	+/-
O14975	S27A2	SLC27A2	Very long-chain acyl-CoA synthetase	0.0113	0.0089	1.27	0.0473	0.0656	0.72
Q5K4L6	S27A3	SLC27A3	Solute carrier family 27 member 3	0.0004	0.0009	0.49	0.0128	0.0154	0.83
Q6P1M0	S27A4	SLC27A4	Long-chain fatty acid transport protein 4	0.0005	-	+/-	0.0066	-	+/-
Q9Y2P5	S27A5	SLC27A5	Bile acyl-CoA synthetase	0.0258	0.0466	0.56	0.0630	0.1252	0.50
B4DYI2	S31C2	SPATA31C2	Putative spermatogenesis-associated protein 31C2	-	0.0001	-/+	-	-	-/-
Q8TB61	S35B2	SLC35B2	Adenosine 3'-phospho 5'-phosphosulfate transporter 1	0.0002	-	+/-	0.0053	-	+/-
Q9NTN3	S35D1	SLC35D1	UDP-glucuronic acid/UDP-N-acetylgalactosamine transporter	-	0.0003	-/+	-	-	-/-
L0R6Q1	S35U4	SLC35A4	SLC35A4 upstream open reading frame protein	0.0030	0.0047	0.63	-	-	-/-

Q99624	S38A3	SLC38A3	Sodium-coupled neutral amino acid transporter 3	-	0.0008	-/+	-	0.0096	-/+
Q15043	S39AE	SLC39A14	Zinc transporter ZIP14	0.0023	-	+/-	0.0091	0.0154	0.59
Q7Z3Q1	S46A3	SLC46A3	Solute carrier family 46 member 3	-	-	-/-	0.0071	-	+/-
P61619	S61A1	SEC61A1	Protein transport protein Sec61 subunit alpha isoform 1	0.0054	0.0003	18.55	0.0385	0.0136	2.82
P0DJI8	SAA1	SAA1	Serum amyloid A-1 protein	0.0195	0.0122	1.60	0.0673	-	+/-
P0DJI9	SAA2	SAA2	Serum amyloid A-2 protein	0.0019	0.0038	0.51	-	-	-/-
P35542	SAA4	SAA4	Serum amyloid A-4 protein	0.0052	0.0007	7.86	-	-	-/-
Q9NTJ5	SAC1	SACM1L	Phosphatidylinositide phosphatase SAC1	0.0016	0.0003	4.56	0.0171	0.0091	1.89
Q9NZJ4	SACS	SACS	Sacsin	-	-	-/-	0.0004	-	+/-
Q9UBE0	SAE1	SAE1	SUMO-activating enzyme subunit 1	0.0024	0.0040	0.59	-	-	-/-
Q9UBT2	SAE2	UBA2	SUMO-activating enzyme subunit 2	0.0005	0.0009	0.54	0.0037	0.0038	0.97
Q15424	SAFB1	SAFB	Scaffold attachment factor B1	0.0002	-	+/-	-	-	-/-
Q14151	SAFB2	SAFB2	Scaffold attachment factor B2	0.0001	-	+/-	0.0011	0.0003	4.25
P23526	SAHH	AHCY	Adenosylhomocysteinase	0.0657	0.0700	0.94	0.0730	0.0917	0.80
O43865	SAHH2	AHCYL1	S-adenosylhomocysteine hydrolase-like protein 1	0.0008	-	+/-	0.0118	0.0122	0.97
Q96HN2	SAHH3	AHCYL2	Adenosylhomocysteinase 3	0.0004	-	+/-	0.0090	-	+/-
Q9Y512	SAM50	SAMM50	Sorting and assembly machinery component 50 homolog	0.0025	0.0002	11.19	0.0201	0.0223	0.90

Q9Y3Z3	SAMH1	SAMHD1	Deoxynucleoside triphosphate triphosphohydrolase SAMHD1	0.0107	0.0065	1.65	0.0312	0.0143	2.18
P02743	SAMP	APCS	Serum amyloid P-component	0.1230	0.0786	1.57	0.0585	0.0805	0.73
P07602	SAP	PSAP	Prosaposin	0.0022	0.0028	0.77	0.0244	0.0102	2.38
O00422	SAP18	SAP18	Histone deacetylase complex subunit SAP18	0.0047	0.0030	1.55	-	-	-/-
P17900	SAP3	GM2A	Ganglioside GM2 activator	0.0047	0.0074	0.63	-	-	-/-
Q9NR31	SAR1A	SAR1A	GTP-binding protein SAR1a	0.0400	0.0318	1.26	0.0243	0.0418	0.58
Q9Y6B6	SAR1B	SAR1B	GTP-binding protein SAR1b	0.0608	0.0881	0.69	0.0846	0.1842	0.46
Q96BY9	SARAF	SARAF	Store-operated calcium entry-associated regulatory factor	0.0032	-	+/-	-	-	-/-
Q9UL12	SARDH	SARDH	Sarcosine dehydrogenase, mitochondrial	0.0210	0.0428	0.49	0.0272	0.0866	0.31
P82979	SARNP	SARNP	SAP domain-containing ribonucleoprotein	0.0038	0.0008	4.94	-	-	-/-
Q15020	SART3	SART3	Squamous cell carcinoma antigen recognized by T-cells 3	0.0003	-	+/-	0.0044	0.0015	3.00
Q9NQZ2	SAS10	UTP3	Something about silencing protein 10	-	-	-/-	-	0.0023	-/+
Q96F10	SAT2	SAT2	Diamine acetyltransferase 2	0.0027	0.0078	0.34	0.0207	0.0284	0.73
Q9Y3A5	SBDS	SBDS	Ribosome maturation protein SBDS	0.0075	0.0046	1.62	0.0116	0.0068	1.70
Q13228	SBP1	SELENBP1	Methanethiol oxidase	0.1005	0.1614	0.62	0.1123	0.2332	0.48
Q6UWP8	SBSN	SBSN	Suprabasin	0.0009	-	+/-	-	-	-/-
P67812	SC11A	SEC11A	Signal peptidase complex catalytic subunit SEC11A	0.0024	0.0047	0.52	0.0178	0.0131	1.35

Q9BY50	SC11C	SEC11C	Signal peptidase complex catalytic subunit SEC11C	0.0032	0.0086	0.37	0.0116	-	+/-
O15027	SC16A	SEC16A	Protein transport protein Sec16A	0.0010	0.0005	2.13	-	-	-/-
Q96JE7	SC16B	SEC16B	Protein transport protein Sec16B	0.0016	0.0003	6.38	0.0046	0.0035	1.31
O75396	SC22B	SEC22B	Vesicle-trafficking protein SEC22b	0.0494	0.0146	3.37	0.0914	0.0948	0.96
Q15436	SC23A	SEC23A	Protein transport protein Sec23A	0.0163	0.0080	2.05	0.0646	0.0547	1.18
Q15437	SC23B	SEC23B	Protein transport protein Sec23B	0.0005	-	+/-	0.0217	0.0108	2.00
O95486	SC24A	SEC24A	Protein transport protein Sec24A	0.0020	-	+/-	0.0153	0.0128	1.19
O95487	SC24B	SEC24B	Protein transport protein Sec24B	0.0006	-	+/-	0.0135	0.0038	3.58
P53992	SC24C	SEC24C	Protein transport protein Sec24C	0.0053	0.0011	4.92	0.0161	0.0112	1.44
O94855	SC24D	SEC24D	Protein transport protein Sec24D	0.0017	0.0007	2.27	0.0199	0.0151	1.32
O94979	SC31A	SEC31A	Protein transport protein Sec31A	0.0129	0.0065	1.99	0.0235	0.0203	1.15
P60468	SC61B	SEC61B	Protein transport protein Sec61 subunit beta	0.0203	0.0029	6.93	0.0209	0.0175	1.19
P60059	SC61G	SEC61G	Protein transport protein Sec61 subunit gamma	0.0017	0.0012	1.39	0.0352	-	+/-
O15127	SCAM2	SCAMP2	Secretory carrier-associated membrane protein 2	0.0021	-	+/-	0.0116	-	+/-
O14828	SCAM3	SCAMP3	Secretory carrier-associated membrane protein 3	0.0042	-	+/-	0.0303	-	+/-
Q9BY12	SCAPE	SCAPER	S phase cyclin A-associated protein in the endoplasmic reticulum	-	-	-/-	0.0010	-	+/-
O95171-2	SCEL	SCEL	Isoform 2 of Sciellin	-	0.0001	-/+	-	-	-/-

Q8WVM8	SCFD1	SCFD1	Sec1 family domain-containing protein 1	0.0044	0.0021	2.10	0.0229	0.0140	1.64
Q8WU76	SCFD2	SCFD2	Sec1 family domain-containing protein 2	-	-	-/-	0.0034	-	+/-
Q96I15	SCLY	SCLY	Selenocysteine lyase	0.0056	0.0090	0.63	0.0476	0.0352	1.35
Q6NUK1	SCMC1	SLC25A24	Calcium-binding mitochondrial carrier protein SCaMC-1	0.0062	0.0004	15.83	-	-	-/-
Q99250	SCN2A	SCN2A	Sodium channel protein type 2 subunit alpha	0.0004	-	+/-	-	-	-/-
Q6R2W3	SCND3	ZBED9	SCAN domain-containing protein 3	-	-	-/-	0.0011	-	+/-
O75880	SCO1	SCO1	Protein SCO1 homolog, mitochondrial	0.0035	0.0056	0.62	-	0.0087	-/+
O43819	SCO2	SCO2	Protein SCO2 homolog, mitochondrial	0.0090	0.0022	4.17	-	-	-/-
P55809	SCOT1	OXCT1	Succinyl-CoA:3-ketoacid coenzyme A transferase 1, mitochondrial	0.0003	0.0004	0.75	-	-	-/-
Q8NBX0	SCPDL	SCCPDH	Saccharopine dehydrogenase-like oxidoreductase	0.0381	0.0219	1.74	0.0896	0.0662	1.35
Q8WTV0	SCRB1	SCARB1	Scavenger receptor class B member 1	0.0041	0.0008	4.88	0.0147	0.0123	1.19
Q14108	SCRB2	SCARB2	Lysosome membrane protein 2	0.0123	0.0061	2.01	0.0241	0.0214	1.13
Q14160	SCRIB	SCRIB	Protein scribble homolog	0.0020	0.0004	4.84	0.0011	-	+/-
Q12765	SCRN1	SCRN1	Secernin-1	0.0007	0.0016	0.46	-	-	-/-
Q96FV2	SCRN2	SCRN2	Secernin-2	0.0046	0.0109	0.43	0.0058	0.0207	0.28
Q0VDG4	SCRN3	SCRN3	Secernin-3	-	0.0009	-/+	-	-	-/-
Q96KG9	SCYL1	SCYL1	N-terminal kinase-like protein	0.0001	0.0004	0.35	0.0076	0.0055	1.39
Q6P3W7	SCYL2	SCYL2	SCY1-like protein 2	-	-	-/-	0.0021	-	+/-

P18827	SDC1	SDC1	Syndecan-1	0.0003	-	+/-	0.0175	0.0181	0.97
O00560	SDCB1	SDCBP	Syntenin-1	0.0018	0.0032	0.55	0.0069	-	+/-
O00560-2	SDCB1	SDCBP	Isoform 2 of Syntenin-1	0.0014	0.0014	0.99	-	-	-/-
O00560-3	SDCB1	SDCBP	Isoform 3 of Syntenin-1	0.0010	0.0011	0.91	-	-	-/-
Q9H190	SDCB2	SDCBP2	Syntenin-2	0.0002	0.0003	0.54	-	-	-/-
P48061	SDF1	CXCL12	Stromal cell-derived factor 1	-	-	-/-	0.0133	0.0321	0.41
Q99470	SDF2	SDF2	Stromal cell-derived factor 2	0.0012	0.0015	0.78	-	-	-/-
Q9HCN8	SDF2L	SDF2L1	Stromal cell-derived factor 2-like protein 1	0.0234	0.0130	1.81	0.0186	-	+/-
P31040	SDHA	SDHA	Succinate dehydrogenase [ubiquinone] flavoprotein subunit, mitochondrial	0.0505	0.0520	0.97	0.0316	0.0706	0.45
P21912	SDHB	SDHB	Succinate dehydrogenase [ubiquinone] iron-sulfur subunit, mitochondrial	0.0161	0.0325	0.50	0.0261	0.0504	0.52
P20132	SDHL	SDS	L-serine dehydratase/L-threonine deaminase	0.0030	0.0024	1.29	0.0143	0.0107	1.34
Q96GA7	SDSL	SDSL	Serine dehydratase-like	0.0072	0.0129	0.56	0.0254	0.0321	0.79
Q9UBV2	SE1L1	SEL1L	Protein sel-1 homolog 1	0.0032	0.0004	8.33	0.0163	0.0058	2.82
P55735-4	SEC13	SEC13	Isoform 4 of Protein SEC13 homolog	-	0.0005	-/+	-	-	-/-
P55735	SEC13	SEC13	Protein SEC13 homolog	0.0282	0.0275	1.03	0.0475	0.0507	0.94
Q12981	SEC20	BNIP1	Vesicle transport protein SEC20	-	-	-/-	0.0056	-	+/-
Q9UGP8	SEC63	SEC63	Translocation protein SEC63 homolog	0.0003	0.0009	0.34	0.0051	0.0075	0.67
O76038	SEGN	SCGN	Secretagogin	0.0002	0.0028	0.07	-	-	-/-
Q96EE3	SEH1	SEH1L	Nucleoporin SEH1	0.0002	0.0003	0.61	-	-	-/-

P57772	SELB	EEFSEC	Selenocysteine-specific elongation factor	0.0002	-	+/-	-	0.0043	-/+
Q8IZQ5	SELH	SELENOH	Selenoprotein H	0.0007	0.0046	0.16	-	-	-/-
Q9BVL4	SELO	SELENOO	Protein adenylyltransferase SelO, mitochondrial	0.0008	0.0030	0.27	0.0076	0.0114	0.67
Q9H4L4	SENP3	SENP3	Sentrin-specific protease 3	0.0002	-	+/-	-	-	-/-
Q96LD8	SENP8	SENP8	Sentrin-specific protease 8	-	0.0006	-/+	-	-	-/-
Q9P0V9	SEP10	SEPTIN10	Septin-10	0.0005	0.0007	0.84	0.0030	0.0048	0.63
Q9NVA2	SEP11	SEPTIN11	Septin-11	0.0112	0.0032	3.49	0.0133	0.0064	2.08
O60613	SEP15	SELENOF	Selenoprotein F	0.0220	0.0138	1.59	0.0124	0.0128	0.97
P49908	SEPP1	SELENOP	Selenoprotein P	0.0006	-	+/-	-	-	-/-
Q12884	SEPR	FAP	Prolyl endopeptidase FAP	-	-	-/-	0.0040	-	+/-
Q8WYJ6	SEPT1	SEPTIN1	Septin-1	0.0003	0.0007	0.47	-	-	-/-
Q15019	SEPT2	SEPTIN2	Septin-2	0.0249	0.0217	1.15	0.0298	0.0251	1.18
O43236	SEPT4	SEPTIN4	Septin-4	-	0.0002	-/+	0.0045	-	+/-
Q99719	SEPT5	SEPTIN5	Septin-5	0.0002	0.0002	1.11	-	-	-/-
Q14141	SEPT6	SEPTIN6	Septin-6	0.0050	0.0014	3.47	0.0155	0.0048	3.25
Q16181	SEPT7	SEPTIN7	Septin-7	0.0125	0.0110	1.14	0.0200	0.0143	1.39
Q92599	SEPT8	SEPTIN8	Septin-8	0.0033	0.0012	2.62	0.0060	-	+/-
Q9UHD8	SEPT9	SEPTIN9	Septin-9	0.0098	0.0051	1.91	0.0273	0.0128	2.13
O43175	SERA	PHGDH	D-3-phosphoglycerate dehydrogenase	0.0223	0.0567	0.39	0.0156	0.0966	0.16

P78330	SERB	PSPH	Phosphoserine phosphatase	0.0036	0.0031	1.16	-	-	-/-
Q9Y617	SERC	PSAT1	Phosphoserine aminotransferase	0.0352	0.0683	0.52	0.0591	0.0879	0.67
P50454	SERPH	SERPINH1	Serpin H1	0.0629	0.0135	4.65	0.0518	0.0100	5.18
Q01105	SET	SET	Protein SET	0.0153	0.0093	1.65	0.0014	-	+/-
Q8WTS6	SETD7	SETD7	Histone-lysine N-methyltransferase SETD7	0.0034	0.0072	0.47	-	-	-/-
P0DME0	SETLP	SETSIP	Protein SETSIP	0.0137	0.0006	21.09	0.0060	-	+/-
Q7Z333	SETX	SETX	Probable helicase senataxin	-	0.0000	-/+	-	-	-/-
Q15637	SF01	SF1	Splicing factor 1	0.0005	-	+/-	-	-	-/-
Q15459	SF3A1	SF3A1	Splicing factor 3A subunit 1	0.0049	0.0014	3.56	0.0069	0.0072	0.97
Q15428	SF3A2	SF3A2	Splicing factor 3A subunit 2	0.0010	0.0003	3.61	-	-	-/-
Q12874	SF3A3	SF3A3	Splicing factor 3A subunit 3	0.0025	0.0011	2.22	0.0086	0.0042	2.03
O75533	SF3B1	SF3B1	Splicing factor 3B subunit 1	0.0003	0.0001	2.68	0.0084	0.0025	3.36
Q13435	SF3B2	SF3B2	Splicing factor 3B subunit 2	0.0002	-	+/-	-	-	-/-
Q15393	SF3B3	SF3B3	Splicing factor 3B subunit 3	0.0032	0.0003	11.78	0.0130	0.0072	1.80
Q15427	SF3B4	SF3B4	Splicing factor 3B subunit 4	0.0027	0.0013	2.08	0.0084	-	+/-
Q9BWJ5	SF3B5	SF3B5	Splicing factor 3B subunit 5	0.0054	0.0040	1.34	-	-	-/-
Q9Y3B4	SF3B6	SF3B6	Splicing factor 3B subunit 6	0.0014	0.0027	0.54	0.0155	-	+/-
P23246	SFPQ	SFPQ	Splicing factor, proline- and glutamine-rich	0.0124	0.0121	1.02	0.0185	0.0141	1.31
Q9H9B4	SFXN1	SFXN1	Sideroflexin-1	0.0274	0.0520	0.53	0.0428	0.0914	0.47
Q96NB2	SFXN2	SFXN2	Sideroflexin-2	-	0.0018	-/+	0.0054	0.0120	0.45

Q9BWM7	SFXN3	SFXN3	Sideroflexin-3	0.0006	-	+/-	0.0186	-	+/-
Q8TD22	SFXN5	SFXN5	Sideroflexin-5	0.0023	0.0039	0.59	-	-	-/-
O95969	SG1D2	SCGB1D2	Secretoglobin family 1D member 2	-	0.0013	-/+	-	-	-/-
Q96BR1	SGK3	SGK3	Serine/threonine-protein kinase Sgk3	-	-	-/-	0.0032	-	+/-
Q5BJF2	SGMR2	TMEM97	Sigma intracellular receptor 2	-	-	-/-	0.0023	-	+/-
O95470	SGPL1	SGPL1	Sphingosine-1-phosphate lyase 1	0.0010	-	+/-	0.0105	0.0091	1.15
Q9Y2Z0-2	SGT1	SUGT1	Isoform 2 of Protein SGT1 homolog	0.0018	0.0062	0.29	-	-	-/-
Q9Y2Z0	SGT1	SUGT1	Protein SGT1 homolog	0.0082	0.0144	0.57	0.0046	0.0048	0.97
O43765	SGTA	SGTA	Small glutamine-rich tetratricopeptide repeat-containing protein alpha	0.0051	0.0047	1.10	-	-	-/-
Q99961	SH3G1	SH3GL1	Endophilin-A2	0.0007	0.0014	0.51	-	-	-/-
Q96B97	SH3K1	SH3KBP1	SH3 domain-containing kinase-binding protein 1	0.0002	-	+/-	0.0033	-	+/-
O75368	SH3L1	SH3BGRL	SH3 domain-binding glutamic acid-rich-like protein	0.0823	0.0356	2.32	0.0371	-	+/-
Q9UJC5	SH3L2	SH3BGRL2	SH3 domain-binding glutamic acid-rich-like protein 2	0.0101	0.0130	0.78	-	-	-/-
Q9H299	SH3L3	SH3BGRL3	SH3 domain-binding glutamic acid-rich-like protein 3	0.0366	0.0294	1.24	0.0207	-	+/-
P29353	SHC1	SHC1	SHC-transforming protein 1	-	-	-/-	0.0036	-	+/-
Q92835	SHIP1	INPP5D	Phosphatidylinositol 3,4,5-trisphosphate 5-phosphatase 1	-	-	-/-	0.0028	-	+/-

Q9Y371	SHLB1	SH3GLB1	Endophilin-B1	0.0043	0.0022	1.98	0.0093	0.0045	2.04
Q9NR46	SHLB2	SH3GLB2	Endophilin-B2	0.0016	0.0012	1.32	-	-	-/-
A0MZ66	SHOT1	SHTN1	Shootin-1	0.0029	0.0026	1.09	-	-	-/-
Q9UHJ6	SHPK	SHPK	Sedoheptulokinase	0.0011	0.0042	0.26	-	0.0236	-/+
Q8TF72	SHRM3	SHROOM3	Protein Shroom3	0.0000	-	+/-	-	-	-/-
Q9HAT2	SIAE	SIAE	Sialate O-acetylesterase	0.0028	0.0018	1.58	0.0192	0.0104	1.85
Q9NR45	SIAS	NANS	Sialic acid synthase	0.0070	0.0187	0.38	0.0122	0.0265	0.46
P15907	SIAT1	ST6GAL1	Beta-galactoside alpha-2,6-sialyltransferase 1	0.0057	-	+/-	0.0330	0.0068	4.84
Q9H173	SIL1	SIL1	Nucleotide exchange factor SIL1	0.0002	-	+/-	-	-	-/-
Q96EX1	SIM12	SMIM12	Small integral membrane protein 12	0.0004	0.0010	0.41	-	-	-/-
Q8IXJ6	SIR2	SIRT2	NAD-dependent protein deacetylase sirtuin-2	0.0006	0.0028	0.20	-	-	-/-
Q9NTG7	SIR3	SIRT3	NAD-dependent protein deacetylase sirtuin-3, mitochondrial	0.0018	0.0026	0.67	-	-	-/-
Q9NXA8	SIR5	SIRT5	NAD-dependent protein deacylase sirtuin-5, mitochondrial	0.0061	0.0062	0.98	0.0093	0.0096	0.97
O75563	SKAP2	SKAP2	Src kinase-associated phosphoprotein 2	0.0013	0.0002	5.47	-	-	-/-
Q15477	SKIV2	SKIV2L	Helicase SKI2W	0.0000	0.0002	0.17	0.0081	0.0024	3.44
P63208	SKP1	SKP1	S-phase kinase-associated protein 1	0.0255	0.0237	1.07	0.0257	0.0134	1.91
Q5T5P2	SKT	KIAA1217	Sickle tail protein homolog	0.0001	-	+/-	-	-	-/-
Q68CJ6	SLIP	NUGGC	Nuclear GTPase SLIP-GC	-	0.0001	-/+	-	-	-/-

Q9GZT3	SLIRP	SLIRP	SRA stem-loop-interacting RNA-binding protein, mitochondrial	0.0015	0.0010	1.44	0.0200	-	+/-
Q9H2G2	SLK	SLK	STE20-like serine/threonine-protein kinase	0.0002	-	+/-	0.0015	-	+/-
Q8WU79	SMAP2	SMAP2	Stromal membrane-associated protein 2	0.0019	0.0005	3.82	0.0069	-	+/-
Q14683	SMC1A	SMC1A	Structural maintenance of chromosomes protein 1A	0.0005	0.0001	8.23	0.0025	0.0008	2.96
Q9UQE7	SMC3	SMC3	Structural maintenance of chromosomes protein 3	0.0003	-	+/-	0.0022	0.0014	1.63
P51531	SMCA2	SMARCA2	Probable global transcription activator SNF2L2	0.0002	-	+/-	0.0008	0.0009	0.97
O60264	SMCA5	SMARCA5	SWI/SNF-related matrix-associated actin-dependent regulator of chromatin subfamily A member 5	0.0000	-	+/-	0.0011	-	+/-
Q969G3-2	SMCE1	SMARCE1	Isoform 2 of SWI/SNF-related matrix-associated actin-dependent regulator of chromatin subfamily E member 1	0.0001	-	+/-	-	-	-/-
Q969G3	SMCE1	SMARCE1	SWI/SNF-related matrix-associated actin-dependent regulator of chromatin subfamily E member 1	-	0.0003	-/+	-	-	-/-
P62314	SMD1	SNRPD1	Small nuclear ribonucleoprotein Sm D1	0.0045	0.0070	0.64	0.0139	0.0080	1.74
P62316	SMD2	SNRPD2	Small nuclear ribonucleoprotein Sm D2	0.0046	0.0057	0.81	0.0207	0.0214	0.97
P62318	SMD3	SNRPD3	Small nuclear ribonucleoprotein Sm D3	0.0058	0.0040	1.45	0.0147	0.0152	0.97
A6NHR9	SMHD1	SMCHD1	Structural maintenance of chromosomes flexible hinge domain-containing protein 1	-	-	-/-	0.0008	-	+/-

Q16637	SMN	SMN1	Survival motor neuron protein	0.0008	0.0005	1.70	-	-	-/-
Q8TAQ2	SMRC2	SMARCC2	SWI/SNF complex subunit SMARCC2	0.0001	-	+/-	-	-	-/-
Q2TAY7	SMU1	SMU1	WD40 repeat-containing protein SMU1	-	-	-/-	0.0033	-	+/-
P54920	SNAA	NAPA	Alpha-soluble NSF attachment protein	0.0894	0.0556	1.61	0.0636	0.0544	1.17
Q9H115	SNAB	NAPB	Beta-soluble NSF attachment protein	0.0010	-	+/-	-	-	-/-
Q99747	SNAG	NAPG	Gamma-soluble NSF attachment protein	0.0085	0.0066	1.28	-	-	-/-
Q7KZF4	SND1	SND1	Staphylococcal nuclease domain-containing protein 1	0.0453	0.0198	2.29	0.0599	0.0434	1.38
Q12824	SNF5	SMARCB1	SWI/SNF-related matrix-associated actin-dependent regulator of chromatin subfamily B member 1	0.0005	0.0002	2.41	-	-	-/-
Q96H20	SNF8	SNF8	Vacuolar-sorting protein SNF8	0.0007	0.0016	0.43	-	-	-/-
O43760	SNG2	SYNGR2	Synaptogyrin-2	0.0028	-	+/-	0.0036	-	+/-
O00161	SNP23	SNAP23	Synaptosomal-associated protein 23	0.0002	0.0014	0.14	-	-	-/-
O95721	SNP29	SNAP29	Synaptosomal-associated protein 29	0.0045	0.0041	1.09	-	-	-/-
Q8WVK2	SNR27	SNRNP27	U4/U6.U5 small nuclear ribonucleoprotein 27 kDa protein	0.0001	-	+/-	-	-	-/-
Q96DI7	SNR40	SNRNP40	U5 small nuclear ribonucleoprotein 40 kDa protein	0.0009	0.0024	0.39	-	-	-/-
P09012	SNRPA	SNRPA	U1 small nuclear ribonucleoprotein A	0.0038	0.0052	0.74	-	-	-/-
Q13884	SNTB1	SNTB1	Beta-1-syntrophin	0.0077	0.0071	1.09	0.0166	0.0120	1.38
Q13425	SNTB2	SNTB2	Beta-2-syntrophin	-	-	-/-	0.0069	-	+/-

O43290	SNUT1	SART1	U4/U6.U5 tri-snRNP-associated protein 1	0.0001	0.0002	0.24	-	-	-/-
Q53GS9	SNUT2	USP39	U4/U6.U5 tri-snRNP-associated protein 2	0.0002	-	+/-	0.0035	-	+/-
Q13573	SNW1	SNW1	SNW domain-containing protein 1	0.0003	0.0001	3.09	-	-	-/-
Q13596	SNX1	SNX1	Sorting nexin-1	0.0032	0.0033	0.96	0.0093	0.0062	1.50
Q9UMY4	SNX12	SNX12	Sorting nexin-12	0.0040	0.0053	0.75	0.0077	-	+/-
Q9NRS6	SNX15	SNX15	Sorting nexin-15	0.0002	0.0005	0.41	-	-	-/-
Q96RF0	SNX18	SNX18	Sorting nexin-18	0.0001	0.0004	0.30	-	-	-/-
O60749	SNX2	SNX2	Sorting nexin-2	0.0043	0.0072	0.61	0.0110	0.0096	1.15
Q9Y343	SNX24	SNX24	Sorting nexin-24	-	0.0006	-/+	-	-	-/-
Q96L92	SNX27	SNX27	Sorting nexin-27	0.0007	0.0007	1.05	0.0096	0.0064	1.50
O60493	SNX3	SNX3	Sorting nexin-3	0.0078	0.0044	1.76	0.0081	-	+/-
O60493-2	SNX3	SNX3	Isoform 2 of Sorting nexin-3	0.0013	0.0009	1.47	-	-	-/-
O95219	SNX4	SNX4	Sorting nexin-4	0.0011	0.0006	1.88	-	-	-/-
Q9Y5X3	SNX5	SNX5	Sorting nexin-5	0.0030	0.0054	0.55	0.0096	0.0118	0.81
Q9UNH7	SNX6	SNX6	Sorting nexin-6	0.0026	0.0021	1.22	0.0114	0.0047	2.43
Q9UNH6	SNX7	SNX7	Sorting nexin-7	-	0.0006	-/+	-	-	-/-
Q9Y5X1	SNX9	SNX9	Sorting nexin-9	0.0002	0.0013	0.13	0.0043	0.0045	0.97
Q9Y6L6	SO1B1	SLCO1B1	Solute carrier organic anion transporter family member 1B1	0.0008	0.0010	0.81	-	0.0124	-/+
Q9NPD5	SO1B3	SLCO1B3	Solute carrier organic anion transporter family member 1B3	0.0010	0.0017	0.59	-	0.0066	-/+

O94956	SO2B1	SLCO2B1	Solute carrier organic anion transporter family member 2B1	0.0003	0.0002	1.70	-	0.0055	-/+
P00441	SODC	SOD1	Superoxide dismutase [Cu-Zn]	0.0552	0.0579	0.95	0.0050	0.0360	0.14
P08294	SODE	SOD3	Extracellular superoxide dismutase [Cu-Zn]	0.0030	0.0031	0.95	-	-	-/-
P04179	SODM	SOD2	Superoxide dismutase [Mn], mitochondrial	0.1353	0.0822	1.65	0.1557	0.0778	2.00
P30626	SORCN	SRI	Sorcin	0.0334	0.0327	1.02	0.0386	0.0128	3.01
Q92673	SORL	SORL1	Sortilin-related receptor	0.0005	-	+/-	0.0009	-	+/-
Q99523	SORT	SORT1	Sortilin	0.0003	-	+/-	0.0025	-	+/-
Q9BQ15	SOSB1	NABP2	SOSS complex subunit B1	-	0.0005	-/+	-	-	-/-
Q9P0Z9	SOX	PIPOX	Peroxisomal sarcosine oxidase	0.0393	0.0418	0.94	0.1065	0.0623	1.71
P35716	SOX11	SOX11	Transcription factor SOX-11	0.0005	-	+/-	-	-	-/-
P23497	SP100	SP100	Nuclear autoantigen Sp-100	0.0007	-	+/-	-	-	-/-
Q9Y5B9	SP16H	SUPT16H	FACT complex subunit SPT16	0.0001	0.0001	2.00	0.0051	-	+/-
O75391	SPAG7	SPAG7	Sperm-associated antigen 7	0.0004	-	+/-	-	-	-/-
Q8N0X7	SPART	SPART	Spartin	0.0001	-	+/-	-	-	-/-
P48595	SPB10	SERPINB10	Serpin B10	0.0003	-	+/-	-	-	-/-
Q96P63	SPB12	SERPINB12	Serpin B12	0.0008	0.0272	0.03	-	-	-/-
P29508	SPB3	SERPINB3	Serpin B3	0.0021	0.0126	0.16	-	-	-/-
P48594	SPB4	SERPINB4	Serpin B4	-	0.0015	-/+	-	-	-/-
P35237	SPB6	SERPINB6	Serpin B6	0.0428	0.0301	1.42	0.0731	0.0384	1.90

P50452	SPB8	SERPINB8	Serpin B8	0.0033	0.0015	2.12	0.0106	-	+/-
P50453	SPB9	SERPINB9	Serpin B9	0.0204	0.0143	1.43	0.0352	0.0205	1.72
Q9HD40	SPCS	SEPSECS	O-phosphoseryl-tRNA(Sec) selenium transferase	-	0.0009	-/+	-	0.0067	-/+
Q9HD40-3	SPCS	SEPSECS	Isoform 3 of O-phosphoseryl-tRNA(Sec) selenium transferase	-	-	-/-	-	0.0075	-/+
Q9Y6A9	SPCS1	SPCS1	Signal peptidase complex subunit 1	0.0060	-	+/-	-	-	-/-
Q15005	SPCS2	SPCS2	Signal peptidase complex subunit 2	0.0093	0.0015	6.19	0.0354	0.0111	3.19
P61009	SPCS3	SPCS3	Signal peptidase complex subunit 3	0.0097	0.0018	5.29	0.0360	0.0241	1.49
Q9BSE5	SPEB	AGMAT	Agmatinase, mitochondrial	0.0308	0.0458	0.67	0.0347	0.0664	0.52
P19623	SPEE	SRM	Spermidine synthase	0.0130	0.0119	1.09	0.0162	0.0120	1.35
O75940	SPF30	SMNDC1	Survival of motor neuron-related-splicing factor 30	0.0002	-	+/-	-	-	-/-
Q96I25	SPF45	RBM17	Splicing factor 45	0.0003	0.0005	0.53	-	-	-/-
Q6Q759	SPG17	SPAG17	Sperm-associated antigen 17	-	0.0000	-/+	-	-	-/-
Q9NZD8	SPG21	SPG21	Maspardin	-	0.0013	-/+	-	-	-/-
P51688	SPHM	SGSH	N-sulphoglucosamine sulphohydrolase	-	-	-/-	0.0069	-	+/-
Q5VVC0	SPO16	SPO16	Protein SPO16 homolog	0.0044	0.0018	2.38	-	-	-/-
Q9HCB6	SPON1	SPON1	Spondin-1	0.0001	-	+/-	-	-	-/-
Q13103	SPP24	SPP2	Secreted phosphoprotein 24	0.0002	-	+/-	-	-	-/-
P09486	SPRC	SPARC	SPARC	0.0057	0.0166	0.35	-	-	-/-

P35270	SPRE	SPR	Sepiapterin reductase	0.0239	0.0458	0.52	0.0758	0.0803	0.94
Q8WW59	SPRY4	SPRYD4	SPRY domain-containing protein 4	0.1013	0.0671	1.51	0.0486	0.0420	1.16
P49903	SPS1	SEPHS1	Selenide, water dikinase 1	0.0035	0.0072	0.48	0.0128	-	+/-
Q99611	SPS2	SEPHS2	Selenide, water dikinase 2	0.0018	0.0057	0.31	0.0173	0.0179	0.97
Q9NUQ6	SPS2L	SPATS2L	SPATS2-like protein	0.0004	0.0002	2.53	-	0.0023	-/+
Q9NUQ6-2	SPS2L	SPATS2L	Isoform 2 of SPATS2-like protein	0.0001	-	+/-	-	-	-/-
P52788	SPSY	SMS	Spermine synthase	0.0024	0.0024	1.00	0.0075	-	+/-
O00267	SPT5H	SUPT5H	Transcription elongation factor SPT5	-	-	-/-	0.0016	-	+/-
Q7KZ85	SPT6H	SUPT6H	Transcription elongation factor SPT6	0.0004	-	+/-	-	0.0009	-/+
P02549	SPTA1	SPTA1	Spectrin alpha chain, erythrocytic 1	0.0040	0.0032	1.23	0.0010	0.0011	0.89
P11277	SPTB1	SPTB	Spectrin beta chain, erythrocytic	0.0019	0.0009	2.24	0.0034	0.0044	0.76
Q01082-3	SPTB2	SPTBN1	Isoform 2 of Spectrin beta chain, non-erythrocytic 1	0.0051	0.0048	1.06	0.0193	-	+/-
Q01082	SPTB2	SPTBN1	Spectrin beta chain, non-erythrocytic 1	0.0284	0.0117	2.43	0.0338	0.0333	1.01
O15269	SPTC1	SPTLC1	Serine palmitoyltransferase 1	0.0008	-	+/-	-	-	-/-
Q13813-2	SPTN1	SPTAN1	Isoform 2 of Spectrin alpha chain, non-erythrocytic 1	0.0099	0.0253	0.39	-	-	-/-
Q13813-3	SPTN1	SPTAN1	Isoform 3 of Spectrin alpha chain, non-erythrocytic 1	0.0223	0.0077	2.90	-	-	-/-
Q13813	SPTN1	SPTAN1	Spectrin alpha chain, non-erythrocytic 1	0.0424	0.0290	1.46	0.0344	0.0394	0.87
O15020	SPTN2	SPTBN2	Spectrin beta chain, non-erythrocytic 2	0.0007	0.0007	1.03	0.0012	0.0058	0.20

Q9H254	SPTN4	SPTBN4	Spectrin beta chain, non-erythrocytic 4	-	-	-/-	0.0002	-	+/-
P21549	SPYA	AGXT	Serine--pyruvate aminotransferase	0.2967	0.5521	0.54	0.4269	0.4804	0.89
Q9Y6N5	SQOR	SQOR	Sulfide:quinone oxidoreductase, mitochondrial	0.0349	0.0283	1.23	0.0712	0.0544	1.31
Q13501	SQSTM	SQSTM1	Sequestosome-1	0.2246	0.0024	94.21	0.2730	-	+/-
Q9HD15	SRA1	SRA1	Steroid receptor RNA activator 1	0.0002	-	+/-	-	-	-/-
Q9BX66-10	SRBS1	SORBS1	Isoform 10 of Sorbin and SH3 domain-containing protein 1	0.0005	-	+/-	-	-	-/-
Q9BX66	SRBS1	SORBS1	Sorbin and SH3 domain-containing protein 1	0.0012	0.0002	5.65	0.0013	0.0020	0.64
O94875	SRBS2	SORBS2	Sorbin and SH3 domain-containing protein 2	0.0024	0.0027	0.87	-	0.0020	-/+
O94875-11	SRBS2	SORBS2	Isoform 11 of Sorbin and SH3 domain-containing protein 2	0.0008	-	+/-	-	-	-/-
O94875-10	SRBS2	SORBS2	Isoform 10 of Sorbin and SH3 domain-containing protein 2	0.0021	0.0038	0.55	-	-	-/-
P12931	SRC	SRC	Proto-oncogene tyrosine-protein kinase Src	-	-	-/-	0.0050	-	+/-
Q14247	SRC8	CTTN	Src substrate cortactin	0.0099	0.0022	4.50	0.0056	0.0039	1.46
Q8WXA9-2	SREK1	SREK1	Isoform 2 of Splicing regulatory glutamine/lysine-rich protein 1	-	-	-/-	0.0025	-	+/-
Q8NEF9	SRFB1	SRFBP1	Serum response factor-binding protein 1	0.0016	-	+/-	-	-	-/-
P49458	SRP09	SRP9	Signal recognition particle 9 kDa protein	0.0078	0.0059	1.34	0.0143	-	+/-
P37108	SRP14	SRP14	Signal recognition particle 14 kDa protein	0.0063	0.0034	1.84	0.0169	0.0175	0.97
P09132	SRP19	SRP19	Signal recognition particle 19 kDa protein	0.0008	-	+/-	0.0071	0.0074	0.97

P61011	SRP54	SRP54	Signal recognition particle 54 kDa protein	0.0022	0.0027	0.82	0.0094	0.0080	1.18
Q9UHB9	SRP68	SRP68	Signal recognition particle subunit SRP68	0.0002	-	+/-	0.0111	0.0045	2.44
O76094	SRP72	SRP72	Signal recognition particle subunit SRP72	0.0011	0.0003	4.03	0.0040	0.0041	0.97
P08240	SRPRA	SRPRA	Signal recognition particle receptor subunit alpha	0.0028	0.0022	1.30	0.0155	0.0145	1.07
Q9Y5M8	SRPRB	SRPRB	Signal recognition particle receptor subunit beta	0.0051	0.0059	0.86	0.0327	0.0186	1.76
P78539	SRPX	SRPX	Sushi repeat-containing protein SRPX	-	0.0002	-/+	-	0.0033	-/+
Q9GZT4	SRR	SRR	Serine racemase	-	0.0004	-/+	-	-	-/-
Q8IYB3	SRRM1	SRRM1	Serine/arginine repetitive matrix protein 1	-	0.0001	-/+	-	-	-/-
Q9BXP5	SRRT	SRRT	Serrate RNA effector molecule homolog	0.0005	0.0001	4.66	0.0013	-	+/-
O75494	SRS10	SRSF10	Serine/arginine-rich splicing factor 10	0.0016	0.0016	0.99	0.0042	-	+/-
Q05519	SRS11	SRSF11	Serine/arginine-rich splicing factor 11	0.0002	-	+/-	-	-	-/-
Q07955	SRSF1	SRSF1	Serine/arginine-rich splicing factor 1	0.0188	0.0121	1.56	0.0121	0.0013	9.10
Q01130	SRSF2	SRSF2	Serine/arginine-rich splicing factor 2	0.0021	0.0008	2.44	-	-	-/-
P84103	SRSF3	SRSF3	Serine/arginine-rich splicing factor 3	0.0048	0.0040	1.20	0.0069	0.0047	1.49
Q08170	SRSF4	SRSF4	Serine/arginine-rich splicing factor 4	0.0003	-	+/-	-	-	-/-
Q13243	SRSF5	SRSF5	Serine/arginine-rich splicing factor 5	0.0009	0.0003	2.92	-	-	-/-
Q13247	SRSF6	SRSF6	Serine/arginine-rich splicing factor 6	0.0018	0.0010	1.72	-	-	-/-
Q16629	SRSF7	SRSF7	Serine/arginine-rich splicing factor 7	0.0038	0.0021	1.77	0.0030	-	+/-
Q13242	SRSF9	SRSF9	Serine/arginine-rich splicing factor 9	0.0014	-	+/-	-	-	-/-

Q9BYN0	SRXN1	SRXN1	Sulfiredoxin-1	0.0056	0.0010	5.72	-	-	-/-
Q04837	SSBP	SSBP1	Single-stranded DNA-binding protein, mitochondrial	0.0115	0.0278	0.41	0.0382	0.0284	1.35
P51649	SSDH	ALDH5A1	Succinate-semialdehyde dehydrogenase, mitochondrial	0.0194	0.0224	0.87	0.0332	0.0735	0.45
Q8TE77	SSH3	SSH3	Protein phosphatase Slingshot homolog 3	0.0008	0.0018	0.42	0.0052	-	+/-
P43307	SSRA	SSR1	Translocon-associated protein subunit alpha	0.0177	0.0091	1.95	0.0264	0.0161	1.64
P51571	SSRD	SSR4	Translocon-associated protein subunit delta	0.1161	0.0383	3.03	0.1070	0.0769	1.39
Q9UNL2	SSRG	SSR3	Translocon-associated protein subunit gamma	-	-	-/-	0.0084	0.0138	0.61
Q08945	SSRP1	SSRP1	FACT complex subunit SSRP1	0.0006	-	+/-	0.0050	0.0025	1.99
Q9NP77	SSU72	SSU72	RNA polymerase II subunit A C-terminal domain phosphatase SSU72	0.0007	0.0006	1.23	-	-	-/-
P50225	ST1A1	SULT1A1	Sulfotransferase 1A1	0.1001	0.1933	0.52	0.0724	0.1382	0.52
P50226	ST1A2	SULT1A2	Sulfotransferase 1A2	0.0112	0.0269	0.42	0.0322	0.0557	0.58
P0DMM9	ST1A3	SULT1A3	Sulfotransferase 1A3	0.0177	0.0148	1.19	0.0147	0.0039	3.77
O43704	ST1B1	SULT1B1	Sulfotransferase family cytosolic 1B member 1	0.0062	0.0171	0.37	0.0049	0.0138	0.35
O00338	ST1C2	SULT1C2	Sulfotransferase 1C2	0.0014	-	+/-	0.0050	-	+/-
O75897	ST1C4	SULT1C4	Sulfotransferase 1C4	0.0006	-	+/-	-	-	-/-
P49888	ST1E1	SULT1E1	Sulfotransferase 1E1	0.0016	0.0019	0.85	-	0.0050	-/+
Q06520	ST2A1	SULT2A1	Bile salt sulfotransferase	0.0953	0.2089	0.46	0.0660	0.1793	0.37

Q9Y365	STA10	STARD10	START domain-containing protein 10	0.0072	0.0163	0.44	0.0186	0.0321	0.58
P42229	STA5A	STAT5A	Signal transducer and activator of transcription 5A	0.0001	-	+/-	0.0050	0.0038	1.31
Q9NY15	STAB1	STAB1	Stabilin-1	0.0001	0.0002	0.76	0.0019	-	+/-
O95630	STABP	STAMBP	STAM-binding protein	0.0004	0.0012	0.29	-	-	-/-
Q92783	STAM1	STAM	Signal transducing adapter molecule 1	-	0.0021	-/+	-	-	-/-
O75886	STAM2	STAM2	Signal transducing adapter molecule 2	0.0012	0.0027	0.44	-	-	-/-
Q9NSY2	STAR5	STARD5	StAR-related lipid transfer protein 5	0.0033	0.0080	0.41	-	-	-/-
Q9NQZ5	STAR7	STARD7	StAR-related lipid transfer protein 7, mitochondrial	0.0001	0.0003	0.32	-	-	-/-
Q9P2P6	STAR9	STARD9	StAR-related lipid transfer protein 9	-	0.0000	-/+	-	-	-/-
P42224	STAT1	STAT1	Signal transducer and activator of transcription 1-alpha/beta	0.0063	0.0037	1.70	0.0413	0.0270	1.53
P52630	STAT2	STAT2	Signal transducer and activator of transcription 2	-	-	-/-	0.0043	0.0022	1.98
P40763	STAT3	STAT3	Signal transducer and activator of transcription 3	0.0022	0.0023	0.96	0.0348	0.0210	1.66
P42226-2	STAT6	STAT6	Isoform 2 of Signal transducer and activator of transcription 6	-	-	-/-	0.0032	-	+/-
P42226	STAT6	STAT6	Signal transducer and activator of transcription 6	-	-	-/-	0.0037	-	+/-
O95793	STAU1	STAU1	Double-stranded RNA-binding protein Staufen homolog 1	0.0022	0.0007	3.00	-	-	-/-

Q9NUL3	STAU2	STAU2	Double-stranded RNA-binding protein Staufen homolog 2	0.0004	-	+/-	-	-	-/-
O95210	STBD1	STBD1	Starch-binding domain-containing protein 1	0.0215	0.0138	1.56	0.0712	0.0448	1.59
Q8NFT2	STEA2	STEAP2	Metalloreductase STEAP2	-	-	-/-	0.0039	-	+/-
Q658P3	STEA3	STEAP3	Metalloreductase STEAP3	0.0017	0.0033	0.51	0.0062	0.0231	0.27
Q687X5	STEA4	STEAP4	Metalloreductase STEAP4	0.0024	0.0002	11.54	0.0108	0.0087	1.24
Q9H5V9	STEEP	STEEP1	STING ER exit protein	0.0004	-	+/-	-	-	-/-
Q13586	STIM1	STIM1	Stromal interaction molecule 1	0.0003	0.0004	0.67	0.0034	0.0035	0.97
Q86WV6	STING	TMEM173	Stimulator of interferon genes protein	0.0005	-	+/-	0.0093	-	+/-
P31948	STIP1	STIP1	Stress-induced-phosphoprotein 1	0.0055	0.0035	1.56	0.0050	0.0034	1.46
O94804	STK10	STK10	Serine/threonine-protein kinase 10	-	-	-/-	0.0018	0.0019	0.97
Q9Y6E0-2	STK24	STK24	Isoform A of Serine/threonine-protein kinase 24	-	0.0010	-/+	-	-	-/-
Q9Y6E0	STK24	STK24	Serine/threonine-protein kinase 24	-	-	-/-	0.0116	0.0037	3.17
O00506	STK25	STK25	Serine/threonine-protein kinase 25	0.0006	0.0006	0.97	0.0058	-	+/-
Q9P289	STK26	STK26	Serine/threonine-protein kinase 26	0.0001	-	+/-	0.0103	-	+/-
Q9P289-2	STK26	STK26	Isoform 2 of Serine/threonine-protein kinase 26	-	0.0006	-/+	-	-	-/-
Q13188	STK3	STK3	Serine/threonine-protein kinase 3	-	0.0007	-/+	-	-	-/-
Q15208	STK38	STK38	Serine/threonine-protein kinase 38	-	-	-/-	0.0029	-	+/-
Q13043	STK4	STK4	Serine/threonine-protein kinase 4	0.0005	0.0018	0.30	0.0069	-	+/-

Q9UJZ1	STML2	STOML2	Stomatin-like protein 2, mitochondrial	0.0604	0.0177	3.42	0.0341	0.0248	1.37
P16949	STMN1	STMN1	Stathmin	0.0064	-	+/-	-	-	-/-
P27105	STOM	STOM	Erythrocyte band 7 integral membrane protein	0.0685	0.0354	1.93	0.0858	0.0795	1.08
Q9Y3F4	STRAP	STRAP	Serine-threonine kinase receptor-associated protein	0.0356	0.0252	1.41	0.0138	0.0232	0.60
O43815	STRN	STRN	Striatin	0.0002	0.0007	0.28	-	-	-/-
Q13033	STRN3	STRN3	Striatin-3	-	0.0011	-/+	-	-	-/-
P46977	STT3A	STT3A	Dolichyl-diphosphooligosaccharide--protein glycosyltransferase subunit STT3A	0.0033	0.0012	2.75	0.0161	0.0132	1.23
Q8TCJ2	STT3B	STT3B	Dolichyl-diphosphooligosaccharide--protein glycosyltransferase subunit STT3B	0.0010	0.0004	2.77	0.0118	0.0064	1.85
Q86Y82	STX12	STX12	Syntaxin-12	0.0002	0.0004	0.41	-	-	-/-
O14662	STX16	STX16	Syntaxin-16	-	0.0008	-/+	-	-	-/-
P56962	STX17	STX17	Syntaxin-17	0.0003	0.0006	0.50	-	-	-/-
Q9P2W9	STX18	STX18	Syntaxin-18	-	-	-/-	0.0039	0.0041	0.97
Q12846	STX4	STX4	Syntaxin-4	0.0001	-	+/-	0.0043	-	+/-
Q13190	STX5	STX5	Syntaxin-5	-	-	-/-	0.0069	-	+/-
O15400	STX7	STX7	Syntaxin-7	0.0002	0.0011	0.16	0.0096	-	+/-
Q9UNK0	STX8	STX8	Syntaxin-8	-	-	-/-	0.0064	-	+/-
Q15833	STXB2	STXBP2	Syntaxin-binding protein 2	0.0008	0.0002	3.80	0.0065	0.0026	2.53
O00186	STXB3	STXBP3	Syntaxin-binding protein 3	0.0005	0.0003	1.51	0.0057	0.0034	1.66

Q8NFX7	STXB6	STXBP6	Syntaxin-binding protein 6	-	0.0004	-/+	-	-	-/-
P53597	SUCA	SUCLG1	Succinate--CoA ligase [ADP/GDP-forming] subunit alpha, mitochondrial	0.0222	0.0399	0.56	0.0129	0.0483	0.27
Q9P2R7	SUCB1	SUCLA2	Succinate--CoA ligase [ADP-forming] subunit beta, mitochondrial	0.0058	0.0055	1.06	0.0053	0.0096	0.56
Q96I99	SUCB2	SUCLG2	Succinate--CoA ligase [GDP-forming] subunit beta, mitochondrial	0.1049	0.1234	0.85	0.0484	0.1329	0.36
Q9HAC7	SUCHY	SUGCT	Succinate--hydroxymethylglutarate CoA-transferase	0.0003	0.0009	0.40	-	0.0045	-/+
Q8NBK3	SUMF1	SUMF1	Formylglycine-generating enzyme	0.0006	-	+/-	-	-	-/-
Q8NBJ7	SUMF2	SUMF2	Inactive C-alpha-formylglycine-generating enzyme 2	0.0121	0.0052	2.30	0.0120	0.0058	2.07
P63165	SUMO1	SUMO1	Small ubiquitin-related modifier 1	-	0.0025	-/+	-	-	-/-
P61956	SUMO2	SUMO2	Small ubiquitin-related modifier 2	0.0023	0.0009	2.64	-	-	-/-
P55854	SUMO3	SUMO3	Small ubiquitin-related modifier 3	0.0011	-	+/-	-	-	-/-
O94901	SUN1	SUN1	SUN domain-containing protein 1	0.0006	-	+/-	-	-	-/-
Q9UH99	SUN2	SUN2	SUN domain-containing protein 2	0.0041	0.0004	9.79	0.0148	0.0024	6.04
P51687	SUOX	SUOX	Sulfite oxidase, mitochondrial	0.0067	0.0109	0.62	0.0040	0.0202	0.20
Q15526	SURF1	SURF1	Surfeit locus protein 1	0.0016	0.0009	1.72	0.0087	0.0090	0.97
O15260-2	SURF4	SURF4	Isoform 2 of Surfeit locus protein 4	0.0006	0.0068	0.09	-	-	-/-
O15260	SURF4	SURF4	Surfeit locus protein 4	0.0068	-	+/-	0.0990	0.0471	2.10
Q9UGT4	SUSD2	SUSD2	Sushi domain-containing protein 2	0.0029	0.0002	12.21	0.0071	-	+/-

Q8IYB8	SUV3	SUPV3L1	ATP-dependent RNA helicase SUPV3L1, mitochondrial	-	-	-/-	0.0020	-	+/-
Q53LP3	SWAHC	SOWAHC	Ankyrin repeat domain-containing protein SOWAHC	0.0001	0.0004	0.25	-	-	-/-
Q9UH65	SWP70	SWAP70	Switch-associated protein 70	0.0003	-	+/-	0.0020	-	+/-
P49588	SYAC	AARS	Alanine--tRNA ligase, cytoplasmic	0.0077	0.0086	0.90	0.0244	0.0228	1.07
Q5JTZ9	SYAM	AARS2	Alanine--tRNA ligase, mitochondrial	0.0005	0.0011	0.45	0.0020	-	+/-
Q96A49	SYAP1	SYAP1	Synapse-associated protein 1	0.0038	0.0138	0.28	-	-	-/-
P49589	SYCC	CARS	Cysteine--tRNA ligase, cytoplasmic	0.0003	0.0007	0.43	0.0058	0.0060	0.97
Q9HA77	SYCM	CARS2	Probable cysteine--tRNA ligase, mitochondrial	0.0001	-	+/-	-	0.0031	-/+
P14868	SYDC	DARS	Aspartate--tRNA ligase, cytoplasmic	0.0238	0.0087	2.72	0.0397	0.0321	1.24
Q6PI48	SYDM	DARS2	Aspartate--tRNA ligase, mitochondrial	0.0020	-	+/-	0.0098	0.0055	1.78
Q5JPH6	SYEM	EARS2	Probable glutamate--tRNA ligase, mitochondrial	0.0012	0.0009	1.36	0.0065	0.0049	1.31
P07814	SYEP	EPRS	Bifunctional glutamate/proline--tRNA ligase	0.0059	0.0011	5.17	0.0198	0.0101	1.96
Q9Y285	SYFA	FARSA	Phenylalanine--tRNA ligase alpha subunit	0.0018	0.0013	1.37	0.0105	0.0070	1.51
Q9NSD9	SYFB	FARSB	Phenylalanine--tRNA ligase beta subunit	0.0027	0.0011	2.53	0.0158	0.0091	1.73
O95363	SYFM	FARS2	Phenylalanine--tRNA ligase, mitochondrial	-	-	-/-	0.0058	0.0039	1.48
P12081	SYHC	HARS	Histidine--tRNA ligase, cytoplasmic	0.0032	0.0027	1.19	0.0078	0.0052	1.50
P49590	SYHM	HARS2	Histidine--tRNA ligase, mitochondrial	0.0008	-	+/-	0.0057	-	+/-

P41252	SYIC	IARS	Isoleucine--tRNA ligase, cytoplasmic	0.0032	0.0008	4.05	0.0182	0.0103	1.76
Q9NSE4	SYIM	IARS2	Isoleucine--tRNA ligase, mitochondrial	0.0089	0.0066	1.34	0.0314	0.0267	1.18
P57105	SYJ2B	SYNJ2BP	Synaptojanin-2-binding protein	0.1106	0.0474	2.34	0.0155	0.0263	0.59
Q15046	SYK	KARS	Lysine--tRNA ligase	0.0068	0.0036	1.87	0.0125	0.0097	1.28
Q9P2J5	SYLC	LARS	Leucine--tRNA ligase, cytoplasmic	0.0013	0.0001	19.96	0.0155	0.0081	1.92
P56192	SYMC	MARS	Methionine--tRNA ligase, cytoplasmic	0.0011	0.0002	4.73	0.0237	0.0101	2.34
Q96GW9	SYMM	MARS2	Methionine--tRNA ligase, mitochondrial	-	-	-/-	0.0065	0.0049	1.31
Q92797	SYMPK	SYMPK	Symplekin	-	-	-/-	0.0013	-	+/-
O43776	SYNC	NARS1	Asparagine--tRNA ligase, cytoplasmic	0.0032	0.0003	11.31	0.0225	0.0174	1.30
Q8NF91	SYNE1	SYNE1	Nesprin-1	0.0000	-	+/-	0.0000	0.0002	0.22
Q8WXH0	SYNE2	SYNE2	Nesprin-2	0.0001	0.0001	0.83	0.0004	-	+/-
Q6ZMZ3	SYNE3	SYNE3	Nesprin-3	-	-	-/-	0.0015	-	+/-
Q9UMS6	SYNP2	SYNPO2	Synaptopodin-2	0.0001	0.0001	1.55	-	-	-/-
Q16563	SYPL1	SYPL1	Synaptophysin-like protein 1	0.0029	-	+/-	0.0109	-	+/-
P47897	SYQ	QARS	Glutamine--tRNA ligase	0.0030	0.0031	0.97	0.0153	0.0258	0.59
P54136	SYRC	RARS	Arginine--tRNA ligase, cytoplasmic	0.0063	0.0041	1.55	0.0363	0.0218	1.66
P49591	SYSC	SARS	Serine--tRNA ligase, cytoplasmic	0.0034	0.0032	1.04	0.0214	0.0208	1.03
Q9NP81	SYSM	SARS2	Serine--tRNA ligase, mitochondrial	0.0013	0.0004	3.83	0.0032	0.0070	0.46
P26639	SYTC	TARS1	Threonine--tRNA ligase 1, cytoplasmic	0.0031	0.0023	1.38	0.0218	0.0139	1.57
Q96C24	SYTL4	SYTL4	Synaptotagmin-like protein 4	-	-	-/-	0.0018	-	+/-

Q9BW92	SYTM	TARS2	Threonine--tRNA ligase, mitochondrial	0.0005	-	+/-	0.0094	0.0082	1.15
P37840	SYUA	SNCA	Alpha-synuclein	0.0064	0.0093	0.69	-	-	-/-
O76070	SYUG	SNCG	Gamma-synuclein	0.0013	0.0034	0.37	-	-	-/-
P26640	SYVC	VARS	Valine--tRNA ligase	0.0110	0.0015	7.32	0.0452	0.0155	2.91
Q5ST30	SYVM	VARS2	Valine--tRNA ligase, mitochondrial	0.0001	-	+/-	-	-	-/-
Q86TM6	SYVN1	SYVN1	E3 ubiquitin-protein ligase synoviolin	0.0002	-	+/-	0.0047	-	+/-
P23381	SYWC	WARS	Tryptophan--tRNA ligase, cytoplasmic	0.0104	0.0060	1.71	0.0235	0.0123	1.92
Q9UGM6	SYWM	WARS2	Tryptophan--tRNA ligase, mitochondrial	0.0003	0.0011	0.29	-	-	-/-
P54577	SYYC	YARS	Tyrosine--tRNA ligase, cytoplasmic	0.0080	0.0005	14.85	0.0165	0.0102	1.62
Q9Y2Z4	SYYM	YARS2	Tyrosine--tRNA ligase, mitochondrial	0.0009	-	+/-	0.0073	0.0036	2.02
Q9H061	T126A	TMEM126A	Transmembrane protein 126A	0.0029	0.0060	0.48	-	-	-/-
Q3YBM2	T176B	TMEM176B	Transmembrane protein 176B	0.0009	-	+/-	0.0098	-	+/-
P13984	T2FB	GTF2F2	General transcription factor IIF subunit 2	0.0009	0.0010	0.94	-	-	-/-
Q7RTR8	T2R42	TAS2R42	Taste receptor type 2 member 42	0.0033	-	+/-	-	-	-/-
Q96EM0	T3HPD	L3HYPDH	Trans-3-hydroxy-L-proline dehydratase	-	0.0010	-/+	-	-	-/-
Q15750	TAB1	TAB1	TGF-beta-activated kinase 1 and MAP3K7-binding protein 1	0.0004	0.0021	0.20	-	-	-/-
Q9BXJ8	TACAN	TMEM120A	Ion channel TACAN	-	-	-/-	0.0121	0.0082	1.47
Q9BSH4	TACO1	TACO1	Translational activator of cytochrome c oxidase 1	0.0037	0.0046	0.80	0.0046	0.0074	0.63
O75478	TAD2A	TADA2A	Transcriptional adapter 2-alpha	0.0001	-	+/-	-	-	-/-

Q13148	TADBP	TARDBP	TAR DNA-binding protein 43	0.0130	0.0050	2.62	0.0133	0.0107	1.25
Q15545	TAF7	TAF7	Transcription initiation factor TFIID subunit 7	-	0.0002	-/+	-	-	-/-
Q01995	TAGL	TAGLN	Transgelin	0.1000	0.0904	1.11	0.0731	0.0267	2.74
P37802	TAGL2	TAGLN2	Transgelin-2	0.1192	0.0508	2.35	0.1463	0.0973	1.50
P37837	TALDO	TALDO1	Transaldolase	0.0852	0.0411	2.07	0.0671	0.0519	1.29
Q96BW9	TAM41	TAMM41	Phosphatidate cytidylyltransferase, mitochondrial	0.0003	-	+/-	-	-	-/-
Q03518	TAP1	TAP1	Antigen peptide transporter 1	0.0007	-	+/-	0.0086	0.0033	2.57
Q03519	TAP2	TAP2	Antigen peptide transporter 2	0.0009	-	+/-	0.0091	-	+/-
Q9H2D6	TARA	TRIOBP	TRIO and F-actin-binding protein	0.0003	0.0001	4.86	-	-	-/-
Q7Z7G0	TARSH	ABI3BP	Target of Nesh-SH3	0.0001	-	+/-	-	-	-/-
Q9UK61	TASOR	TASOR	Protein TASOR	-	-	-/-	-	0.0010	-/+
Q6P1N9	TATD1	TATDN1	Putative deoxyribonuclease TATDN1	0.0018	0.0008	2.26	0.0050	-	+/-
Q17R31	TATD3	TATDN3	Putative deoxyribonuclease TATDN3	-	0.0005	-/+	-	-	-/-
Q86VP1	TAXB1	TAX1BP1	Tax1-binding protein 1	0.0002	-	+/-	-	-	-/-
Q9C0C2	TB182	TNKS1BP1	182 kDa tankyrase-1-binding protein	0.0011	0.0016	0.73	0.0033	-	+/-
Q71U36	TBA1A	TUBA1A	Tubulin alpha-1A chain	0.0028	-	+/-	-	-	-/-
P68363	TBA1B	TUBA1B	Tubulin alpha-1B chain	0.4057	0.2179	1.86	0.1382	0.1657	0.83
Q9BQE3	TBA1C	TUBA1C	Tubulin alpha-1C chain	0.2110	0.1085	1.94	0.3032	0.2402	1.26
P0DPH7	TBA3C	TUBA3C	Tubulin alpha-3C chain	0.1313	0.0497	2.64	0.1178	0.0819	1.44

P68366	TBA4A	TUBA4A	Tubulin alpha-4A chain	0.3685	0.1990	1.85	0.2621	0.2598	1.01
Q9NY65	TBA8	TUBA8	Tubulin alpha-8 chain	0.0011	0.0107	0.10	-	-	-/-
A6NHL2	TBAL3	TUBAL3	Tubulin alpha chain-like 3	0.0027	0.0002	11.30	-	-	-/-
Q9H4B7	TBB1	TUBB1	Tubulin beta-1 chain	0.0025	-	+/-	0.0411	-	+/-
Q13885	TBB2A	TUBB2A	Tubulin beta-2A chain	0.6437	0.2685	2.40	1.0665	0.6341	1.68
Q13509	TBB3	TUBB3	Tubulin beta-3 chain	0.1092	0.0018	62.28	0.1929	0.0704	2.74
P04350	TBB4A	TUBB4A	Tubulin beta-4A chain	0.1599	0.0963	1.66	0.5090	0.0945	5.39
P68371	TBB4B	TUBB4B	Tubulin beta-4B chain	0.9226	0.4431	2.08	1.2945	0.7899	1.64
P07437	TBB5	TUBB	Tubulin beta chain	1.1655	0.5637	2.07	1.1461	0.9043	1.27
Q9BUF5	TBB6	TUBB6	Tubulin beta-6 chain	0.0898	0.0408	2.20	0.2067	0.1167	1.77
Q3ZCM7	TBB8	TUBB8	Tubulin beta-8 chain	0.0252	-	+/-	-	0.0636	-/+
A6NNZ2	TBB8B	TUBB8B	Tubulin beta 8B	0.0590	0.0073	8.08	-	0.0726	-/+
Q8TC07	TBC15	TBC1D15	TBC1 domain family member 15	-	-	-/-	0.0025	-	+/-
Q9HA65	TBC17	TBC1D17	TBC1 domain family member 17	0.0006	0.0004	1.41	-	0.0034	-/+
Q9ULP9	TBC24	TBC1D24	TBC1 domain family member 24	-	-	-/-	0.0080	0.0031	2.56
Q66K14	TBC9B	TBC1D9B	TBC1 domain family member 9B	-	-	-/-	0.0029	0.0015	1.97
O75347	TBCA	TBCA	Tubulin-specific chaperone A	0.0236	0.0079	2.99	-	-	-/-
Q99426	TBCB	TBCB	Tubulin-folding cofactor B	0.0134	0.0054	2.46	-	-	-/-
Q15814	TBCC	TBCC	Tubulin-specific chaperone C	0.0003	0.0002	1.56	0.0060	-	+/-
Q9BTW9-4	TBCD	TBCD	Isoform 4 of Tubulin-specific chaperone D	-	-	-/-	0.0024	-	+/-

Q9BTW9	TBCD	TBCD	Tubulin-specific chaperone D	-	-	-/-	0.0024	-	+/-
Q92609	TBCD5	TBC1D5	TBC1 domain family member 5	-	-	-/-	0.0035	-	+/-
Q15813	TBCE	TBCE	Tubulin-specific chaperone E	0.0002	-	+/-	0.0100	-	+/-
Q5QJ74	TBCEL	TBCEL	Tubulin-specific chaperone cofactor E-like protein	0.0002	0.0014	0.11	-	-	-/-
Q9UPU7	TBD2B	TBC1D2B	TBC1 domain family member 2B	-	-	-/-	0.0005	-	+/-
P23258	TBG1	TUBG1	Tubulin gamma-1 chain	0.0021	0.0012	1.74	-	-	-/-
Q9UHD2	TBK1	TBK1	Serine/threonine-protein kinase TBK1	-	-	-/-	0.0022	-	+/-
Q9BZK7	TBL1R	TBL1XR1	F-box-like/WD repeat-containing protein TBL1XR1	0.0007	0.0003	2.43	-	-	-/-
Q9Y4P3	TBL2	TBL2	Transducin beta-like protein 2	0.0057	0.0022	2.62	0.0224	0.0113	1.98
P23193	TCEA1	TCEA1	Transcription elongation factor A protein 1	0.0016	0.0016	1.05	-	-	-/-
O75764	TCEA3	TCEA3	Transcription elongation factor A protein 3	0.0003	-	+/-	-	-	-/-
P53999	TCP4	SUB1	Activated RNA polymerase II transcriptional coactivator p15	0.0277	0.0081	3.41	0.0169	0.0125	1.35
P17987	TCPA	TCP1	T-complex protein 1 subunit alpha	0.0279	0.0133	2.10	0.0246	0.0308	0.80
P78371	TCPB	CCT2	T-complex protein 1 subunit beta	0.0359	0.0217	1.65	0.0454	0.0534	0.85
P50991	TCPD	CCT4	T-complex protein 1 subunit delta	0.0181	0.0145	1.25	0.0315	0.0308	1.02
P48643-2	TCPE	CCT5	Isoform 2 of T-complex protein 1 subunit epsilon	-	-	-/-	-	0.0020	-/+
P48643	TCPE	CCT5	T-complex protein 1 subunit epsilon	0.0193	0.0243	0.79	0.0184	0.0329	0.56

P49368	TCPG	CCT3	T-complex protein 1 subunit gamma	0.0206	0.0142	1.45	0.0341	0.0421	0.81
Q99832	TCPH	CCT7	T-complex protein 1 subunit eta	0.0160	0.0086	1.85	0.0338	0.0366	0.92
P50990	TCPQ	CCT8	T-complex protein 1 subunit theta	0.0393	0.0346	1.14	0.0473	0.0572	0.83
Q92526-3	TCPW	CCT6B	Isoform 3 of T-complex protein 1 subunit zeta-2	-	0.0005	-/+	-	-	-/-
P40227	TCPZ	CCT6A	T-complex protein 1 subunit zeta	0.0188	0.0114	1.65	0.0240	0.0189	1.27
O14776	TCRG1	TCERG1	Transcription elongation regulator 1	0.0002	0.0001	3.11	-	-	-/-
P13693	TCTP	TPT1	Translationally-controlled tumor protein	0.0347	0.0402	0.86	0.0453	0.0207	2.19
Q9Y2W6	TDRKH	TDRKH	Tudor and KH domain-containing protein	0.0006	0.0006	1.04	-	-	-/-
Q9NYB0	TE2IP	TERF2IP	Telomeric repeat-binding factor 2-interacting protein 1	-	0.0016	-/+	-	-	-/-
Q15185	TEBP	PTGES3	Prostaglandin E synthase 3	0.0224	0.0144	1.56	0.0215	0.0271	0.79
Q9NZ01	TECR	TECR	Very-long-chain enoyl-CoA reductase	0.0045	0.0023	1.94	0.0289	0.0161	1.80
P24821-2	TENA	TNC	Isoform 2 of Tenascin	0.0005	0.0014	0.33	-	-	-/-
P24821	TENA	TNC	Tenascin	0.0188	0.0033	5.69	0.0130	0.0042	3.05
Q9HBL0	TENS1	TNS1	Tensin-1	0.0011	0.0010	1.07	0.0026	0.0041	0.63
Q68CZ2	TENS3	TNS3	Tensin-3	0.0001	0.0002	0.35	0.0022	0.0022	0.97
P22105	TENX	TNXB	Tenascin-X	0.0086	0.0024	3.60	0.0049	0.0029	1.71
P55072	TERA	VCP	Transitional endoplasmic reticulum ATPase	0.0852	0.0501	1.70	0.0744	0.0623	1.20
Q15554	TERF2	TERF2	Telomeric repeat-binding factor 2	0.0001	-	+/-	-	-	-/-
Q9UGI8	TES	TES	Testin	0.0017	0.0006	2.86	-	-	-/-

P05452	TETN	CLEC3B	Tetranectin	0.0202	0.0071	2.84	0.0147	-	+/-
Q00403	TF2B	GTF2B	Transcription initiation factor IIB	0.0003	0.0011	0.32	-	-	-/-
Q13888	TF2H2	GTF2H2	General transcription factor IIH subunit 2	0.0002	-	+/-	-	-	-/-
Q04206	TF65	RELA	Transcription factor p65	-	0.0005	-/+	0.0040	-	+/-
Q00059	TFAM	TFAM	Transcription factor A, mitochondrial	0.0007	0.0009	0.82	-	-	-/-
Q8WVM0	TFB1M	TFB1M	Dimethyladenosine transferase 1, mitochondrial	-	0.0003	-/+	-	-	-/-
Q9H5Q4	TFB2M	TFB2M	Dimethyladenosine transferase 2, mitochondrial	-	-	-/-	0.0035	-	+/-
Q12800	TFCP2	TFCP2	Alpha-globin transcription factor CP2	-	0.0002	-/+	-	-	-/-
Q92734	TFG	TFG	Protein TFG	0.0808	0.0429	1.88	0.0556	0.0184	3.03
P02786	TFR1	TFRC	Transferrin receptor protein 1	0.0092	0.0001	77.86	0.0277	0.0048	5.73
Q9UP52	TFR2	TFR2	Transferrin receptor protein 2	0.0045	-	+/-	0.0207	0.0082	2.52
P22735	TGM1	TGM1	Protein-glutamine gamma-glutamyltransferase K	-	0.0006	-/+	-	-	-/-
P21980-2	TGM2	TGM2	Isoform 2 of Protein-glutamine gamma-glutamyltransferase 2	0.0002	-	+/-	-	-	-/-
P21980	TGM2	TGM2	Protein-glutamine gamma-glutamyltransferase 2	0.0714	0.0596	1.20	0.0604	0.0993	0.61
Q08188	TGM3	TGM3	Protein-glutamine gamma-glutamyltransferase E	-	0.0021	-/+	-	-	-/-

Q5JRA6	TGO1	MIA3	Transport and Golgi organization protein 1 homolog	0.0014	0.0002	6.01	0.0022	0.0018	1.21
Q5JRA6-2	TGO1	MIA3	Isoform 2 of Transport and Golgi organization protein 1 homolog	-	-	-/-	-	0.0019	-/+
Q8WY91	THAP4	THAP4	THAP domain-containing protein 4	-	0.0004	-/+	-	-	-/-
P24557	THAS	TBXAS1	Thromboxane-A synthase	-	-	-/-	0.0093	0.0055	1.68
P05543	THBG	SERPINA7	Thyroxine-binding globulin	0.0024	-	+/-	-	-	-/-
Q5T1C6	THEM4	THEM4	Acyl-coenzyme A thioesterase THEM4	0.0010	-	+/-	-	-	-/-
Q8WUY1	THEM6	THEM6	Protein THEM6	-	0.0005	-/+	0.0103	0.0068	1.51
Q9BWD1	THIC	ACAT2	Acetyl-CoA acetyltransferase, cytosolic	0.0104	0.0351	0.30	0.0111	0.0688	0.16
P09110	THIK	ACAA1	3-ketoacyl-CoA thiolase, peroxisomal	0.0606	0.1166	0.52	0.1387	0.1745	0.79
P24752	THIL	ACAT1	Acetyl-CoA acetyltransferase, mitochondrial	0.1985	0.2928	0.68	0.1919	0.3568	0.54
P42765	THIM	ACAA2	3-ketoacyl-CoA thiolase, mitochondrial	0.2173	0.5193	0.42	0.2647	0.8818	0.30
P10599	THIO	TXN	Thioredoxin	0.1244	0.0812	1.53	0.1707	0.0832	2.05
Q99757	THIOM	TXN2	Thioredoxin, mitochondrial	0.0117	0.0150	0.78	-	-	-/-
Q5TEJ8	THMS2	THEMIS2	Protein THEMIS2	-	-	-/-	0.0045	-	+/-
Q8IYQ7	THNS1	THNSL1	Threonine synthase-like 1	0.0013	0.0022	0.59	0.0072	0.0158	0.45
Q8NI27	THOC2	THOC2	THO complex subunit 2	-	-	-/-	0.0018	-	+/-
Q96J01	THOC3	THOC3	THO complex subunit 3	0.0012	0.0009	1.39	-	-	-/-
Q86V81	THOC4	ALYREF	THO complex subunit 4	0.0019	0.0036	0.51	0.0106	0.0052	2.05
Q13769	THOC5	THOC5	THO complex subunit 5 homolog	-	-	-/-	0.0021	-	+/-

Q86W42	THOC6	THOC6	THO complex subunit 6 homolog	0.0003	0.0005	0.61	-	-	-/-
Q6I9Y2	THOC7	THOC7	THO complex subunit 7 homolog	0.0011	0.0004	2.95	-	-	-/-
P52888	THOP1	THOP1	Thimet oligopeptidase	0.0001	0.0005	0.29	0.0048	0.0050	0.97
P00734	THRB	F2	Prothrombin	0.0223	0.0101	2.21	0.0383	0.0192	2.00
Q92748	THRSP	THRSP	Thyroid hormone-inducible hepatic protein	-	0.0059	-/+	-	-	-/-
P25325-2	THTM	MPST	Isoform 2 of 3-mercaptopyruvate sulfurtransferase	0.0074	0.0118	0.63	-	0.0623	-/+
P25325	THTM	MPST	3-mercaptopyruvate sulfurtransferase	0.0901	0.0687	1.31	0.0541	0.0264	2.05
Q9BU02	THTPA	THTPA	Thiamine-triphosphatase	0.0021	0.0049	0.42	-	-	-/-
Q16762	THTR	TST	Thiosulfate sulfurtransferase	0.0718	0.1179	0.61	0.0602	0.1083	0.56
Q9NXG2	THUM1	THUMPD1	THUMP domain-containing protein 1	0.0003	-	+/-	0.0058	-	+/-
Q9BV44	THUM3	THUMPD3	THUMP domain-containing protein 3	-	-	-/-	0.0030	-	+/-
P04216	THY1	THY1	Thy-1 membrane glycoprotein	0.0064	-	+/-	0.0428	0.0138	3.11
Q9P016	THYN1	THYN1	Thymocyte nuclear protein 1	0.0004	0.0003	1.50	-	-	-/-
O60830	TI17B	TIMM17B	Mitochondrial import inner membrane translocase subunit Tim17-B	0.0003	0.0006	0.54	-	-	-/-
P31483	TIA1	TIA1	Nucleolysin TIA-1 isoform p40	0.0010	0.0005	1.79	-	-	-/-
Q01085	TIAR	TIAL1	Nucleolysin TIAR	0.0030	0.0015	2.05	0.0060	-	+/-
Q7Z2Z1	TICRR	TICRR	Treslin	-	0.0000	-/+	-	-	-/-
Q13263	TIF1B	TRIM28	Transcription intermediary factor 1-beta	0.0106	0.0047	2.28	0.0141	0.0078	1.82
Q9NQ88	TIGAR	TIGAR	Fructose-2,6-bisphosphatase TIGAR	0.0004	0.0007	0.55	0.0062	-	+/-

P62072	TIM10	TIMM10	Mitochondrial import inner membrane translocase subunit Tim10	0.0020	0.0075	0.27	-	-	-/-
Q9Y5L4	TIM13	TIMM13	Mitochondrial import inner membrane translocase subunit Tim13	0.0106	0.0147	0.72	0.0169	0.0215	0.79
Q96DA6	TIM14	DNAJC19	Mitochondrial import inner membrane translocase subunit TIM14	0.0003	0.0010	0.33	-	-	-/-
Q9Y3D7	TIM16	PAM16	Mitochondrial import inner membrane translocase subunit TIM16	0.0011	0.0009	1.34	-	-	-/-
Q9BVV7	TIM21	TIMM21	Mitochondrial import inner membrane translocase subunit Tim21	0.0010	-	+/-	-	-	-/-
O14925	TIM23	TIMM23	Mitochondrial import inner membrane translocase subunit Tim23	-	0.0023	-/+	0.0133	-	+/-
O43615	TIM44	TIMM44	Mitochondrial import inner membrane translocase subunit TIM44	0.0023	0.0013	1.77	0.0100	0.0120	0.83
Q3ZCQ8	TIM50	TIMM50	Mitochondrial import inner membrane translocase subunit TIM50	0.0073	0.0053	1.38	0.0102	0.0084	1.21
O60220	TIM8A	TIMM8A	Mitochondrial import inner membrane translocase subunit Tim8 A	0.0013	0.0049	0.26	-	-	-/-
Q9Y5J9	TIM8B	TIMM8B	Mitochondrial import inner membrane translocase subunit Tim8 B	0.0013	0.0047	0.28	-	-	-/-
Q9Y5J7	TIM9	TIMM9	Mitochondrial import inner membrane translocase subunit Tim9	0.0055	0.0030	1.87	-	-	-/-
P01033	TIMP1	TIMP1	Metalloproteinase inhibitor 1	0.0110	-	+/-	0.0144	-	+/-
P35625	TIMP3	TIMP3	Metalloproteinase inhibitor 3	-	-	-/-	0.0121	0.0175	0.69

Q9UJW2	TINAG	TINAG	Tubulointerstitial nephritis antigen	0.0003	-	+/-	-	-	-/-
Q9GZM7	TINAL	TINAGL1	Tubulointerstitial nephritis antigen-like	0.0027	0.0033	0.83	0.0160	0.0152	1.05
O75663	TIPRL	TIPRL	TIP41-like protein	0.0007	0.0003	2.00	0.0062	-	+/-
Q9BRJ7	TIRR	NUDT16L1	Tudor-interacting repair regulator protein	0.0008	0.0009	0.94	-	-	-/-
P47974	TISD	ZFP36L2	mRNA decay activator protein ZFP36L2	0.0002	-	+/-	-	-	-/-
Q8WZ42-6	TITIN	TTN	Isoform 6 of Titin	0.0001	-	+/-	-	0.0003	-/+
Q8WZ42-12	TITIN	TTN	Isoform 12 of Titin	0.0000	-	+/-	-	-	-/-
Q8WZ42	TITIN	TTN	Titin	0.0002	0.0000	3.33	0.0001	0.0001	0.67
Q3LXA3	TKFC	TKFC	Triokinase/FMN cyclase	0.0477	0.1362	0.35	0.0509	0.1400	0.36
P29401	TKT	TKT	Transketolase	0.0857	0.0262	3.28	0.1266	0.0589	2.15
Q96MV1	TLCD4	TLCD4	TLC domain-containing protein 4	-	0.0004	-/+	0.0081	0.0083	0.97
Q9Y490	TLN1	TLN1	Talin-1	0.0350	0.0162	2.15	0.0413	0.0341	1.21
Q9Y4G6	TLN2	TLN2	Talin-2	-	0.0001	-/+	-	-	-/-
Q7L0Y3	TM10C	TRMT10C	tRNA methyltransferase 10 homolog C	0.0007	0.0007	1.02	-	-	-/-
Q86UB9	TM135	TMEM135	Transmembrane protein 135	-	-	-/-	0.0034	-	+/-
Q9P0S9	TM14C	TMEM14C	Transmembrane protein 14C	0.0070	0.0500	0.14	0.1452	0.0476	3.05
Q8N614	TM156	TMEM156	Transmembrane protein 156	0.0002	-	+/-	-	-	-/-
O75674	TM1L1	TOM1L1	TOM1-like protein 1	0.0004	0.0028	0.15	0.0049	-	+/-
Q6ZVM7	TM1L2	TOM1L2	TOM1-like protein 2	-	0.0006	-/+	-	-	-/-
Q6UW68	TM205	TMEM205	Transmembrane protein 205	0.0152	0.0019	8.11	0.0491	0.0595	0.83

Q6NUQ4	TM214	TMEM214	Transmembrane protein 214	-	-	-/-	0.0028	-	+/-
Q8N2U0	TM256	TMEM256	Transmembrane protein 256	0.0021	0.0045	0.46	-	-	-/-
Q8WUH6	TM263	TMEM263	Transmembrane protein 263	0.0065	0.0051	1.27	-	-	-/-
Q969M1	TM40L	TOMM40L	Mitochondrial import receptor subunit TOM40B	0.0003	-	+/-	-	-	-/-
O15321	TM9S1	TM9SF1	Transmembrane 9 superfamily member 1	-	-	-/-	0.0042	-	+/-
Q99805	TM9S2	TM9SF2	Transmembrane 9 superfamily member 2	0.0007	0.0002	3.68	0.0260	0.0055	4.71
Q9HD45	TM9S3	TM9SF3	Transmembrane 9 superfamily member 3	0.0012	-	+/-	0.0173	0.0082	2.12
Q92544	TM9S4	TM9SF4	Transmembrane 9 superfamily member 4	0.0016	-	+/-	0.0139	0.0037	3.78
Q9UM00	TMCO1	TMCO1	Calcium load-activated calcium channel	0.0010	0.0002	4.36	0.0075	0.0045	1.64
Q15363	TMED2	TMED2	Transmembrane emp24 domain-containing protein 2	0.0406	0.0176	2.31	0.0483	0.0227	2.13
Q9Y3Q3	TMED3	TMED3	Transmembrane emp24 domain-containing protein 3	0.0014	-	+/-	-	-	-/-
Q7Z7H5	TMED4	TMED4	Transmembrane emp24 domain-containing protein 4	0.0113	0.0099	1.15	0.0288	0.0154	1.87
Q9Y3A6	TMED5	TMED5	Transmembrane emp24 domain-containing protein 5	0.0100	0.0051	1.95	0.0238	0.0144	1.65
Q9Y3B3	TMED7	TMED7	Transmembrane emp24 domain-containing protein 7	0.0281	0.0137	2.06	0.0399	0.0241	1.65
Q9BVK6	TMED9	TMED9	Transmembrane emp24 domain-containing protein 9	0.0391	0.0156	2.50	0.0528	0.0291	1.81

P49755	TMEDA	TMED10	Transmembrane emp24 domain-containing protein 10	0.0552	0.0228	2.42	0.0816	0.0493	1.65
P82094	TMF1	TMF1	TATA element modulatory factor	0.0002	-	+/-	-	-	-/-
Q9NVH6	TMLH	TMLHE	Trimethyllysine dioxygenase, mitochondrial	0.0003	0.0004	0.80	0.0042	-	+/-
P17152	TMM11	TMEM11	Transmembrane protein 11, mitochondrial	-	0.0013	-/+	-	-	-/-
P57088	TMM33	TMEM33	Transmembrane protein 33	0.0046	-	+/-	0.0323	0.0096	3.37
Q9BTV4	TMM43	TMEM43	Transmembrane protein 43	0.0011	0.0002	4.61	0.0147	0.0045	3.23
Q6P2H8	TMM53	TMEM53	Transmembrane protein 53	-	-	-/-	0.0058	-	+/-
Q9BUB7	TMM70	TMEM70	Transmembrane protein 70, mitochondrial	0.0053	0.0005	10.09	0.0077	-	+/-
P28289	TMOD1	TMOD1	Tropomodulin-1	0.0018	0.0049	0.37	-	-	-/-
Q9NZR1	TMOD2	TMOD2	Tropomodulin-2	0.0001	-	+/-	-	-	-/-
Q9NYL9	TMOD3	TMOD3	Tropomodulin-3	0.0177	0.0180	0.98	0.0147	0.0123	1.19
Q9H3N1	TMX1	TMX1	Thioredoxin-related transmembrane protein 1	0.0053	0.0011	4.93	0.0071	0.0115	0.62
Q9Y320	TMX2	TMX2	Thioredoxin-related transmembrane protein 2	-	-	-/-	0.0084	-	+/-
Q96JJ7	TMX3	TMX3	Protein disulfide-isomerase TMX3	-	-	-/-	0.0084	-	+/-
Q9H1E5	TMX4	TMX4	Thioredoxin-related transmembrane protein 4	0.0019	-	+/-	0.0107	0.0111	0.97
Q6ICL3	TNG2	TANGO2	Transport and Golgi organization protein 2 homolog	0.0010	0.0036	0.27	-	-	-/-
P02585	TNNC2	TNNC2	Troponin C, skeletal muscle	0.0003	-	+/-	-	-	-/-
Q92973	TNPO1	TNPO1	Transportin-1	0.0008	0.0004	2.33	0.0103	0.0133	0.77
O14787	TNPO2	TNPO2	Transportin-2	0.0002	-	+/-	-	-	-/-

Q9Y5L0	TNPO3	TNPO3	Transportin-3	0.0001	-	+/-	0.0037	-	+/-
Q63HR2	TNS2	TNS2	Tensin-2	0.0000	0.0002	0.22	-	0.0052	-/+
Q8N6T0	TO6BL	TOP6BL	Type 2 DNA topoisomerase 6 subunit B-like	-	0.0002	-/+	-	-	-/-
Q5JTV8	TOIP1	TOR1AIP1	Torsin-1A-interacting protein 1	0.0057	0.0005	12.36	0.0080	0.0062	1.29
Q5JTV8-2	TOIP1	TOR1AIP1	Isoform 2 of Torsin-1A-interacting protein 1	0.0018	0.0006	3.13	0.0051	0.0072	0.71
Q8NFQ8	TOIP2	TOR1AIP2	Torsin-1A-interacting protein 2	0.0016	-	+/-	-	-	-/-
Q9H0E2	TOLIP	TOLLIP	Toll-interacting protein	0.0121	0.0115	1.05	0.0294	0.0184	1.60
O60784	TOM1	TOM1	Target of Myb protein 1	0.0127	0.0162	0.78	0.0134	0.0053	2.51
Q9NS69	TOM22	TOMM22	Mitochondrial import receptor subunit TOM22 homolog	0.0511	0.0236	2.17	0.0441	0.0076	5.78
Q15785	TOM34	TOMM34	Mitochondrial import receptor subunit TOM34	0.0010	0.0011	0.93	-	-	-/-
O96008	TOM40	TOMM40	Mitochondrial import receptor subunit TOM40 homolog	0.0026	0.0129	0.20	0.0152	-	+/-
O94826	TOM70	TOMM70	Mitochondrial import receptor subunit TOM70	0.0117	0.0037	3.17	0.0128	0.0120	1.06
P11387	TOP1	TOP1	DNA topoisomerase 1	0.0004	-	+/-	0.0028	0.0017	1.63
P11388-3	TOP2A	TOP2A	Isoform 3 of DNA topoisomerase 2-alpha	0.0000	-	+/-	-	-	-/-
P11388	TOP2A	TOP2A	DNA topoisomerase 2-alpha	-	-	-/-	0.0008	-	+/-
Q02880	TOP2B	TOP2B	DNA topoisomerase 2-beta	0.0004	-	+/-	0.0020	-	+/-
Q92547	TOPB1	TOPBP1	DNA topoisomerase 2-binding protein 1	0.0001	-	+/-	-	-	-/-

Q9NS56	TOPRS	TOPORS	E3 ubiquitin-protein ligase Topors	-	-	-/-	0.0011	-	+/-
O14656	TOR1A	TOR1A	Torsin-1A	0.0040	0.0021	1.89	0.0054	-	+/-
O14657	TOR1B	TOR1B	Torsin-1B	0.0002	0.0003	0.61	0.0047	-	+/-
Q9H497	TOR3A	TOR3A	Torsin-3A	0.0004	-	+/-	-	-	-/-
Q93096	TP4A1	PTP4A1	Protein tyrosine phosphatase type IVA 1	0.0016	0.0011	1.44	0.0127	-	+/-
Q12974	TP4A2	PTP4A2	Protein tyrosine phosphatase type IVA 2	0.0003	0.0005	0.61	0.0088	-	+/-
Q8WVP5	TP8L1	TNFAIP8L1	Tumor necrosis factor alpha-induced protein 8-like protein 1	-	0.0003	-/+	-	-	-/-
Q6P589	TP8L2	TNFAIP8L2	Tumor necrosis factor alpha-induced protein 8-like protein 2	0.0011	0.0004	2.67	0.0081	-	+/-
A5PLN9	TPC13	TRAPPC13	Trafficking protein particle complex subunit 13	-	0.0003	-/+	-	-	-/-
P0DI81	TPC2A	TRAPPC2	Trafficking protein particle complex subunit 2	0.0020	0.0023	0.85	-	-	-/-
P0DI82	TPC2B	TRAPPC2B	Trafficking protein particle complex subunit 2B	0.0021	-	+/-	-	-	-/-
Q9UL33	TPC2L	TRAPPC2L	Trafficking protein particle complex subunit 2-like protein	0.0084	0.0024	3.53	-	-	-/-
Q86SZ2	TPC6B	TRAPPC6B	Trafficking protein particle complex subunit 6B	0.0003	0.0024	0.13	-	-	-/-
P55327	TPD52	TPD52	Tumor protein D52	0.0280	0.0128	2.18	0.0095	-	+/-
Q16890	TPD53	TPD52L1	Tumor protein D53	-	0.0009	-/+	-	-	-/-
O43399	TPD54	TPD52L2	Tumor protein D54	0.0569	0.0273	2.09	0.0162	-	+/-

P60174	TPIS	TPI1	Triosephosphate isomerase	0.2098	0.1353	1.55	0.2213	0.2155	1.03
Q9H3S4	TPK1	TPK1	Thiamin pyrophosphokinase 1	0.0013	0.0029	0.44	-	-	-/-
P09493-5	TPM1	TPM1	Isoform 5 of Tropomyosin alpha-1 chain	0.0019	0.0026	0.74	-	0.0024	-/+
P09493-2	TPM1	TPM1	Isoform 2 of Tropomyosin alpha-1 chain	-	0.0012	-/+	-	0.0024	-/+
P09493-10	TPM1	TPM1	Isoform 10 of Tropomyosin alpha-1 chain	0.0017	-	+/-	-	-	-/-
P09493	TPM1	TPM1	Tropomyosin alpha-1 chain	0.0070	0.0013	5.22	-	-	-/-
P09493-6	TPM1	TPM1	Isoform 6 of Tropomyosin alpha-1 chain	0.0005	0.0008	0.58	-	-	-/-
P07951	TPM2	TPM2	Tropomyosin beta chain	0.0030	0.0019	1.60	0.0066	0.0027	2.41
P07951-2	TPM2	TPM2	Isoform 2 of Tropomyosin beta chain	0.0057	0.0011	5.09	0.0024	0.0026	0.92
P06753	TPM3	TPM3	Tropomyosin alpha-3 chain	0.0024	0.0010	2.51	-	-	-/-
P06753-2	TPM3	TPM3	Isoform 2 of Tropomyosin alpha-3 chain	0.0309	0.0055	5.63	0.0189	0.0112	1.69
P67936	TPM4	TPM4	Tropomyosin alpha-4 chain	0.0126	0.0039	3.19	0.0115	0.0077	1.49
P51580	TPMT	TPMT	Thiopurine S-methyltransferase	0.0048	0.0113	0.43	0.0143	0.0410	0.35
O14773	TPP1	TPP1	Tripeptidyl-peptidase 1	0.0455	0.0366	1.24	0.0296	0.0318	0.93
P29144	TPP2	TPP2	Tripeptidyl-peptidase 2	0.0024	0.0019	1.21	0.0118	0.0189	0.63
Q9Y5R8	TPPC1	TRAPPC1	Trafficking protein particle complex subunit 1	0.0026	0.0019	1.37	-	-	-/-
O43617	TPPC3	TRAPPC3	Trafficking protein particle complex subunit 3	0.0127	0.0094	1.35	-	-	-/-
Q9Y296	TPPC4	TRAPPC4	Trafficking protein particle complex subunit 4	0.0064	0.0046	1.37	0.0093	-	+/-
Q8IUR0	TPPC5	TRAPPC5	Trafficking protein particle complex subunit 5	0.0009	0.0014	0.67	-	-	-/-
O94811	TPPP	TPPP	Tubulin polymerization-promoting protein	0.0002	0.0007	0.24	-	-	-/-

P59282	TPPP2	TPPP2	Tubulin polymerization-promoting protein family member 2	-	0.0004	-/+	-	-	-/-
Q9BW30	TPPP3	TPPP3	Tubulin polymerization-promoting protein family member 3	0.0008	0.0003	2.52	-	-	-/-
P12270	TPR	TPR	Nucleoprotein TPR	0.0005	0.0001	6.23	-	-	-/-
Q5T0D9	TPRGL	TPRG1L	Tumor protein p63-regulated gene 1-like protein	0.0007	-	+/-	0.0077	-	+/-
Q9Y3C4	TPRKB	TPRKB	EKC/KEOPS complex subunit TPRKB	0.0005	0.0011	0.48	-	-	-/-
O15533	TPSN	TAPBP	Tapasin	0.0015	0.0005	2.83	0.0033	-	+/-
Q9UI30	TR112	TRMT112	Multifunctional methyltransferase subunit TRM112-like protein	0.0127	0.0027	4.62	0.0169	-	+/-
Q9Y2W1	TR150	THRAP3	Thyroid hormone receptor-associated protein 3	0.0001	-	+/-	0.0009	-	+/-
Q13595	TRA2A	TRA2A	Transformer-2 protein homolog alpha	0.0007	0.0003	2.31	0.0029	-	+/-
P62995	TRA2B	TRA2B	Transformer-2 protein homolog beta	0.0034	0.0016	2.17	0.0071	0.0028	2.54
Q9H4I3	TRABD	TRABD	TraB domain-containing protein	0.0004	-	+/-	-	-	-/-
Q15628	TRADD	TRADD	Tumor necrosis factor receptor type 1-associated DEATH domain protein	0.0028	0.0031	0.90	0.0054	-	+/-
Q9Y4K3	TRAF6	TRAF6	TNF receptor-associated factor 6	-	0.0002	-/+	-	-	-/-
Q15629	TRAM1	TRAM1	Translocating chain-associated membrane protein 1	0.0003	-	+/-	0.0103	0.0050	2.05
Q12931	TRAP1	TRAP1	Heat shock protein 75 kDa, mitochondrial	0.0153	0.0092	1.66	0.0333	0.0244	1.36

P02787	TRFE	TF	Serotransferrin	0.2467	0.1273	1.94	0.1655	0.1752	0.94
P02788	TRFL	LTF	Lactotransferrin	0.1509	0.0071	21.11	0.1765	0.0227	7.77
Q14258	TRI25	TRIM25	E3 ubiquitin/ISG15 ligase TRIM25	0.0015	0.0018	0.81	0.0183	0.0135	1.36
Q12899	TRI26	TRIM26	Tripartite motif-containing protein 26	-	-	-/-	0.0041	-	+/-
Q9BRZ2	TRI56	TRIM56	E3 ubiquitin-protein ligase TRIM56	-	-	-/-	0.0021	-	+/-
Q9C037	TRIM4	TRIM4	E3 ubiquitin-protein ligase TRIM4	-	0.0002	-/+	-	-	-/-
Q9BZR9	TRIM8	TRIM8	E3 ubiquitin-protein ligase TRIM8	-	0.0002	-/+	-	-	-/-
Q9C026	TRIM9	TRIM9	E3 ubiquitin-protein ligase TRIM9	0.0001	-	+/-	-	-	-/-
O75962	TRIO	TRIO	Triple functional domain protein	-	-	-/-	-	0.0005	-/+
Q15654	TRIP6	TRIP6	Thyroid receptor-interacting protein 6	0.0013	0.0003	5.01	-	-	-/-
Q15643	TRIPB	TRIP11	Thyroid receptor-interacting protein 11	0.0002	0.0000	6.87	-	0.0011	-/+
Q14669	TRIPC	TRIP12	E3 ubiquitin-protein ligase TRIP12	-	-	-/-	0.0020	-	+/-
Q96Q11	TRNT1	TRNT1	CCA tRNA nucleotidyltransferase 1, mitochondrial	0.0008	0.0012	0.65	0.0032	-	+/-
Q86TN4	TRPT1	TRPT1	tRNA 2'-phosphotransferase 1	0.0006	-	+/-	-	-	-/-
Q8NER1	TRPV1	TRPV1	Transient receptor potential cation channel subfamily V member 1	-	0.0001	-/+	-	-	-/-
Q9HBA0	TRPV4	TRPV4	Transient receptor potential cation channel subfamily V member 4	0.0001	-	+/-	-	-	-/-
Q9Y606	TRUA	PUS1	tRNA pseudouridine synthase A	0.0003	0.0002	1.72	0.0050	-	+/-
Q16881	TRXR1	TXNRD1	Thioredoxin reductase 1, cytoplasmic	0.0177	0.0013	13.97	0.0299	0.0052	5.71

Q9NNW7	TRXR2	TXNRD2	Thioredoxin reductase 2, mitochondrial	0.0059	0.0019	3.11	0.0145	0.0175	0.83
P07477	TRY1	PRSS1	Trypsin-1	0.1298	0.1023	1.27	-	-	-/-
P07478	TRY2	PRSS2	Trypsin-2	0.0004	-	+/-	-	-	-/-
P35030	TRY3	PRSS3	Trypsin-3	0.0044	0.0204	0.22	-	-	-/-
Q8NHM4	TRY6	PRSS3P2	Putative trypsin-6	-	0.0008	-/+	-	-	-/-
P20231	TRYB2	TPSB2	Tryptase beta-2	0.0250	0.0257	0.97	0.0291	-	+/-
Q99816	TS101	TSG101	Tumor susceptibility gene 101 protein	0.0002	0.0007	0.23	-	-	-/-
Q8TE23	TS1R2	TAS1R2	Taste receptor type 1 member 2	-	0.0063	-/+	-	-	-/-
Q8WUA8	TSK	TSKU	Tsukushin	0.0008	-	+/-	-	-	-/-
Q15631	TSN	TSN	Translin	0.0012	-	+/-	0.0149	0.0071	2.10
P19075	TSN8	TSPAN8	Tetraspanin-8	-	-	-/-	0.0133	-	+/-
Q99598	TSNAX	TSNAX	Translin-associated protein X	0.0030	0.0029	1.02	-	-	-/-
P07996	TSP1	THBS1	Thrombospondin-1	0.0049	0.0004	11.11	0.0085	0.0017	4.86
P35442	TSP2	THBS2	Thrombospondin-2	0.0012	-	+/-	0.0120	-	+/-
P30536	TSPO	TSPO	Translocator protein	-	-	-/-	0.0207	-	+/-
Q2NL82	TSR1	TSR1	Pre-rRNA-processing protein TSR1 homolog	-	-	-/-	0.0017	-	+/-
Q8NFU3-4	TSTD1	TSTD1	Isoform 4 of Thiosulfate:glutathione sulfurtransferase	0.0007	-	+/-	-	-	-/-
Q8NFU3	TSTD1	TSTD1	Thiosulfate:glutathione sulfurtransferase	0.0083	0.0038	2.20	-	-	-/-
Q5SRH9	TT39A	TTC39A	Tetratricopeptide repeat protein 39A	-	0.0002	-/+	-	-	-/-

Q99614	TTC1	TTC1	Tetratricopeptide repeat protein 1	0.0023	0.0008	2.80	-	-	-/-
Q6DKK2	TTC19	TTC19	Tetratricopeptide repeat protein 19, mitochondrial	0.0002	0.0010	0.19	-	-	-/-
Q96AY4	TTC28	TTC28	Tetratricopeptide repeat protein 28	-	-	-/-	-	0.0002	-/+
P53804	TTC3	TTC3	E3 ubiquitin-protein ligase TTC3	0.0001	-	+/-	-	-	-/-
A6NLP5	TTC36	TTC36	Tetratricopeptide repeat protein 36	0.0027	0.0263	0.10	-	0.0173	-/+
Q6PGP7	TTC37	TTC37	Tetratricopeptide repeat protein 37	0.0001	-	+/-	0.0021	0.0016	1.30
Q5R3I4	TTC38	TTC38	Tetratricopeptide repeat protein 38	0.0061	0.0220	0.28	0.0122	0.0564	0.22
Q92623	TTC9A	TTC9	Tetratricopeptide repeat protein 9A	0.0002	-	+/-	-	-	-/-
Q8N5M4	TTC9C	TTC9C	Tetratricopeptide repeat protein 9C	0.0009	0.0011	0.82	-	-	-/-
P02766	TTHY	TTR	Transthyretin	0.2562	0.1244	2.06	0.0433	0.0384	1.13
Q14166	TTL12	TTLL12	Tubulin--tyrosine ligase-like protein 12	0.0008	0.0030	0.28	0.0141	0.0167	0.84
P49638	TTPA	TTPA	Alpha-tocopherol transfer protein	0.0055	0.0093	0.59	0.0329	0.0223	1.48
Q9BTX7	TTPAL	TTPAL	Alpha-tocopherol transfer protein-like	-	0.0003	-/+	-	-	-/-
Q12792	TWF1	TWF1	Twinfilin-1	0.0018	0.0036	0.49	0.0086	0.0065	1.32
Q6IBS0	TWF2	TWF2	Twinfilin-2	0.0057	0.0068	0.84	0.0116	0.0094	1.24
O14907	TX1B3	TAX1BP3	Tax1-binding protein 3	0.0010	0.0034	0.28	-	-	-/-
Q6PKC3	TXD11	TXNDC11	Thioredoxin domain-containing protein 11	-	-	-/-	0.0021	-	+/-
O95881	TXD12	TXNDC12	Thioredoxin domain-containing protein 12	0.0330	0.0156	2.11	0.0116	-	+/-
Q9BRA2	TXD17	TXNDC17	Thioredoxin domain-containing protein 17	0.0345	0.0303	1.14	0.0371	0.0597	0.62

P83876	TXN4A	TXNL4A	Thioredoxin-like protein 4A	0.0015	0.0011	1.40	-	-	-/-
Q8NBS9	TXND5	TXNDC5	Thioredoxin domain-containing protein 5	0.0676	0.0311	2.17	0.0587	0.0440	1.33
O14530	TXND9	TXNDC9	Thioredoxin domain-containing protein 9	0.0005	-	+/-	-	-	-/-
O43396	TXNL1	TXNL1	Thioredoxin-like protein 1	0.0576	0.0298	1.93	0.0194	0.0201	0.97
P53007	TXTP	SLC25A1	Tricarboxylate transport protein, mitochondrial	0.0145	0.0182	0.80	0.0327	0.0426	0.77
P19971	TYPH	TYMP	Thymidine phosphorylase	0.0643	0.0292	2.20	0.0844	0.0671	1.26
A2RUC4	TYW5	TYW5	tRNA wybutosine-synthesizing protein 5	-	0.0004	-/+	-	-	-/-
A6NIH7	U119B	UNC119B	Protein unc-119 homolog B	-	0.0006	-/+	-	-	-/-
Q01081	U2AF1	U2AF1	Splicing factor U2AF 35 kDa subunit	0.0011	-	+/-	0.0043	-	+/-
Q01081-4	U2AF1	U2AF1	Isoform 4 of Splicing factor U2AF 35 kDa subunit	-	0.0006	-/+	-	-	-/-
P26368	U2AF2	U2AF2	Splicing factor U2AF 65 kDa subunit	0.0043	0.0004	10.46	0.0083	0.0045	1.82
Q8WU68	U2AF4	U2AF1L4	Splicing factor U2AF 26 kDa subunit	0.0013	-	+/-	-	-	-/-
P0DN76	U2AF5	U2AF1L5	Splicing factor U2AF 35 kDa subunit-like protein	0.0061	0.0032	1.89	-	-	-/-
O75643	U520	SNRNP200	U5 small nuclear ribonucleoprotein 200 kDa helicase	0.0006	0.0001	5.34	0.0090	0.0053	1.71
Q15029	U5S1	EFTUD2	116 kDa U5 small nuclear ribonucleoprotein component	0.0010	0.0005	2.05	0.0230	0.0087	2.64
Q16222	UAP1	UAP1	UDP-N-acetylhexosamine pyrophosphorylase	0.0017	0.0033	0.51	0.0114	0.0140	0.81
P51668	UB2D1	UBE2D1	Ubiquitin-conjugating enzyme E2 D1	0.0013	-	+/-	-	-	-/-

P61077	UB2D3	UBE2D3	Ubiquitin-conjugating enzyme E2 D3	0.0043	0.0038	1.13	0.0133	-	+/-
P62253	UB2G1	UBE2G1	Ubiquitin-conjugating enzyme E2 G1	0.0012	0.0056	0.22	-	-	-/-
P60604	UB2G2	UBE2G2	Ubiquitin-conjugating enzyme E2 G2	0.0021	0.0055	0.38	-	-	-/-
P68036	UB2L3	UBE2L3	Ubiquitin-conjugating enzyme E2 L3	0.0043	0.0032	1.36	0.0084	-	+/-
O14933	UB2L6	UBE2L6	Ubiquitin/ISG15-conjugating enzyme E2 L6	0.0026	0.0075	0.35	-	-	-/-
P49427	UB2R1	CDC34	Ubiquitin-conjugating enzyme E2 R1	0.0007	0.0016	0.41	-	-	-/-
Q712K3	UB2R2	UBE2R2	Ubiquitin-conjugating enzyme E2 R2	0.0003	0.0008	0.39	-	-	-/-
Q13404	UB2V1	UBE2V1	Ubiquitin-conjugating enzyme E2 variant 1	0.0090	0.0091	0.98	0.0175	0.0113	1.55
Q15819	UB2V2	UBE2V2	Ubiquitin-conjugating enzyme E2 variant 2	0.0123	0.0232	0.53	-	-	-/-
P22314	UBA1	UBA1	Ubiquitin-like modifier-activating enzyme 1	0.0364	0.0360	1.01	0.0500	0.0628	0.80
Q8TBC4	UBA3	UBA3	NEDD8-activating enzyme E1 catalytic subunit	0.0018	0.0018	0.98	0.0093	0.0070	1.32
Q9GZZ9	UBA5	UBA5	Ubiquitin-like modifier-activating enzyme 5	0.0027	0.0033	0.80	0.0051	0.0082	0.62
A0AVT1	UBA6	UBA6	Ubiquitin-like modifier-activating enzyme 6	0.0004	0.0007	0.66	0.0028	0.0033	0.85
P41226	UBA7	UBA7	Ubiquitin-like modifier-activating enzyme 7	0.0005	0.0003	1.97	0.0036	0.0038	0.97
Q9BSL1	UBAC1	UBAC1	Ubiquitin-associated domain-containing protein 1	0.0004	0.0008	0.55	-	-	-/-
P0CG47	UBB	UBB	Polyubiquitin-B	0.3259	0.1122	2.90	0.0074	0.0037	2.03
P61081	UBC12	UBE2M	NEDD8-conjugating enzyme Ubc12	0.0073	0.0074	0.99	0.0133	0.0087	1.52
P63279	UBC9	UBE2I	SUMO-conjugating enzyme UBC9	0.0041	0.0041	1.01	0.0093	-	+/-

Q8WVY7	UBCP1	UBLCP1	Ubiquitin-like domain-containing CTD phosphatase 1	0.0021	0.0007	3.08	-	-	-/-
O15205	UBD	UBD	Ubiquitin D	0.0019	-	+/-	0.0081	-	+/-
P49459	UBE2A	UBE2A	Ubiquitin-conjugating enzyme E2 A	-	0.0017	-/+	-	-	-/-
P62256	UBE2H	UBE2H	Ubiquitin-conjugating enzyme E2 H	0.0062	0.0034	1.86	-	-	-/-
P61086	UBE2K	UBE2K	Ubiquitin-conjugating enzyme E2 K	0.0238	0.0178	1.34	0.0140	0.0144	0.97
P61088	UBE2N	UBE2N	Ubiquitin-conjugating enzyme E2 N	0.0263	0.0195	1.35	0.0175	0.0139	1.26
Q9H832	UBE2Z	UBE2Z	Ubiquitin-conjugating enzyme E2 Z	-	0.0006	-/+	-	-	-/-
Q05086	UBE3A	UBE3A	Ubiquitin-protein ligase E3A	-	-	-/-	0.0017	0.0018	0.97
Q15386	UBE3C	UBE3C	Ubiquitin-protein ligase E3C	-	-	-/-	0.0025	-	+/-
Q14139	UBE4A	UBE4A	Ubiquitin conjugation factor E4 A	-	-	-/-	0.0019	0.0041	0.47
O14562	UBFD1	UBFD1	Ubiquitin domain-containing protein UBFD1	0.0005	0.0005	1.11	-	-	-/-
P11441	UBL4A	UBL4A	Ubiquitin-like protein 4A	0.0011	0.0006	1.85	-	-	-/-
Q9BZL1	UBL5	UBL5	Ubiquitin-like protein 5	-	0.0015	-/+	-	-	-/-
Q14694	UBP10	USP10	Ubiquitin carboxyl-terminal hydrolase 10	0.0002	0.0008	0.20	-	-	-/-
P54578	UBP14	USP14	Ubiquitin carboxyl-terminal hydrolase 14	0.0064	0.0067	0.96	0.0158	0.0103	1.54
Q9Y4E8	UBP15	USP15	Ubiquitin carboxyl-terminal hydrolase 15	0.0013	0.0024	0.56	0.0027	0.0027	0.97
Q9Y6I4	UBP3	USP3	Ubiquitin carboxyl-terminal hydrolase 3	0.0001	-	+/-	-	-	-/-
Q70CQ4	UBP31	USP31	Ubiquitin carboxyl-terminal hydrolase 31	-	0.0001	-/+	-	-	-/-
Q9P275	UBP36	USP36	Ubiquitin carboxyl-terminal hydrolase 36	0.0001	-	+/-	-	-	-/-

Q13107	UBP4	USP4	Ubiquitin carboxyl-terminal hydrolase 4	-	-	-/-	0.0019	-	+/-
Q9NVE5	UBP40	USP40	Ubiquitin carboxyl-terminal hydrolase 40	0.0001	-	+/-	-	-	-/-
Q96K76	UBP47	USP47	Ubiquitin carboxyl-terminal hydrolase 47	-	0.0009	-/+	-	-	-/-
P45974-2	UBP5	USP5	Isoform Short of Ubiquitin carboxyl-terminal hydrolase 5	-	-	-/-	0.0013	-	+/-
P45974	UBP5	USP5	Ubiquitin carboxyl-terminal hydrolase 5	0.0170	0.0184	0.92	0.0214	0.0160	1.34
Q93009	UBP7	USP7	Ubiquitin carboxyl-terminal hydrolase 7	0.0005	0.0008	0.63	0.0061	0.0023	2.70
Q9UMX0	UBQL1	UBQLN1	Ubiquilin-1	-	0.0054	-/+	-	-	-/-
Q9UHD9	UBQL2	UBQLN2	Ubiquilin-2	0.0006	0.0029	0.21	-	-	-/-
Q9NRR5	UBQL4	UBQLN4	Ubiquilin-4	0.0005	0.0015	0.31	-	-	-/-
Q5T4S7	UBR4	UBR4	E3 ubiquitin-protein ligase UBR4	-	-	-/-	0.0015	0.0010	1.56
Q8N806	UBR7	UBR7	Putative E3 ubiquitin-protein ligase UBR7	-	0.0003	-/+	-	-	-/-
Q04323	UBXN1	UBXN1	UBX domain-containing protein 1	0.0003	-	+/-	-	-	-/-
Q92575	UBXN4	UBXN4	UBX domain-containing protein 4	0.0004	0.0002	2.42	0.0025	0.0026	0.97
O94888	UBXN7	UBXN7	UBX domain-containing protein 7	-	0.0004	-/+	-	-	-/-
P09936	UCHL1	UCHL1	Ubiquitin carboxyl-terminal hydrolase isozyme L1	0.0042	0.0017	2.39	-	-	-/-
P15374	UCHL3	UCHL3	Ubiquitin carboxyl-terminal hydrolase isozyme L3	0.0276	0.0738	0.37	0.0155	0.0161	0.97
Q9Y5K5	UCHL5	UCHL5	Ubiquitin carboxyl-terminal hydrolase isozyme L5	0.0103	0.0124	0.83	0.0146	0.0187	0.78

Q9HA47	UCK1	UCK1	Uridine-cytidine kinase 1	-	0.0003	-/+	-	-	-/-
Q9BZX2	UCK2	UCK2	Uridine-cytidine kinase 2	0.0002	-	+/-	-	-	-/-
P47985	UCRI	UQCRFS1	Cytochrome b-c1 complex subunit Rieske, mitochondrial	0.0805	0.0366	2.20	0.0278	0.0337	0.83
P0C7P4	UCRIL	UQCRFS1P1	Putative cytochrome b-c1 complex subunit Rieske-like protein 1	0.0018	0.0050	0.36	-	-	-/-
P22309	UD11	UGT1A1	UDP-glucuronosyltransferase 1-1	0.0274	0.0074	3.71	0.0609	0.0569	1.07
P35503	UD13	UGT1A3	UDP-glucuronosyltransferase 1-3	0.0068	0.0219	0.31	0.0396	0.0728	0.54
P22310	UD14	UGT1A4	UDP-glucuronosyltransferase 1-4	0.0409	0.0505	0.81	0.0856	0.1046	0.82
P35504	UD15	UGT1A5	UDP-glucuronosyltransferase 1A5	-	-	-/-	0.0068	-	+/-
P19224	UD16	UGT1A6	UDP-glucuronosyltransferase 1-6	0.0503	0.0124	4.05	0.0755	0.0402	1.88
P19224-3	UD16	UGT1A6	Isoform 3 of UDP-glucuronosyltransferase 1-6	-	-	-/-	-	0.0461	-/+
O60656	UD19	UGT1A9	UDP-glucuronosyltransferase 1-9	0.0393	0.0285	1.38	0.0702	0.0570	1.23
Q9Y4X1	UD2A1	UGT2A1	UDP-glucuronosyltransferase 2A1	-	0.0004	-/+	-	-	-/-
P0DTE4	UD2A1	UGT2A1	UDP-glucuronosyltransferase 2A1	-	-	-/-	0.0117	0.0143	0.81
Q6UWM9	UD2A3	UGT2A3	UDP-glucuronosyltransferase 2A3	0.0035	0.0003	12.02	0.0119	0.0147	0.81
P06133-3	UD2B4	UGT2B4	Isoform 3 of UDP-glucuronosyltransferase 2B4	-	0.0004	-/+	-	-	-/-
P06133	UD2B4	UGT2B4	UDP-glucuronosyltransferase 2B4	0.0672	0.0332	2.03	0.0640	0.0474	1.35
P16662	UD2B7	UGT2B7	UDP-glucuronosyltransferase 2B7	0.0689	0.0664	1.04	0.1201	0.1081	1.11

P36537	UDB10	UGT2B10	UDP-glucuronosyltransferase 2B10	0.0148	0.0186	0.80	0.0525	0.0792	0.66
O75310	UDB11	UGT2B11	UDP-glucuronosyltransferase 2B11	0.0007	0.0004	2.00	0.0175	0.0072	2.42
P54855	UDB15	UGT2B15	UDP-glucuronosyltransferase 2B15	0.0357	0.0287	1.25	0.0603	0.0545	1.11
O75795	UDB17	UGT2B17	UDP-glucuronosyltransferase 2B17	0.0213	0.0268	0.79	0.0370	0.0641	0.58
Q8IX04	UEVLD	UEVLD	Ubiquitin-conjugating enzyme E2 variant 3	0.0001	-	+/-	-	-	-/-
Q9Y3C8	UFC1	UFC1	Ubiquitin-fold modifier-conjugating enzyme 1	0.0034	0.0050	0.68	0.0084	-	+/-
Q92890	UFD1	UFD1	Ubiquitin recognition factor in ER-associated degradation protein 1	0.0070	0.0046	1.54	0.0163	0.0087	1.87
O94874	UFL1	UFL1	E3 UFM1-protein ligase 1	0.0038	0.0034	1.12	0.0088	0.0150	0.59
P61960	UFM1	UFM1	Ubiquitin-fold modifier 1	0.0006	0.0022	0.27	-	-	-/-
Q9NUQ7	UFSP2	UFSP2	Ufm1-specific protease 2	0.0009	-	+/-	0.0143	0.0068	2.09
O60701	UGDH	UGDH	UDP-glucose 6-dehydrogenase	0.0833	0.0439	1.89	0.0882	0.0805	1.10
Q9NYU2	UGGG1	UGGT1	UDP-glucose:glycoprotein glucosyltransferase 1	0.0184	0.0049	3.78	0.0302	0.0180	1.68
Q16851	UGPA	UGP2	UTP--glucose-1-phosphate uridylyltransferase	0.0909	0.2347	0.39	0.0757	0.2536	0.30
Q13564	ULA1	NAE1	NEDD8-activating enzyme E1 regulatory subunit	0.0002	0.0002	0.94	0.0089	0.0053	1.68
P11172	UMPS	UMPS	Uridine 5'-monophosphate synthase	0.0023	0.0003	7.97	0.0142	0.0100	1.42
Q70J99	UN13D	UNC13D	Protein unc-13 homolog D	-	-	-/-	0.0025	-	+/-
Q9H3U1	UN45A	UNC45A	Protein unc-45 homolog A	0.0006	-	+/-	0.0044	-	+/-

Q8IV45	UN5CL	UNC5CL	UNC5C-like protein	0.0013	0.0003	4.89	-	-	-/-
P13051	UNG	UNG	Uracil-DNA glycosylase	-	0.0016	-/+	-	-	-/-
Q9BTM9	URM1	URM1	Ubiquitin-related modifier 1	-	0.0021	-/+	-	-	-/-
Q86UX7	URP2	FERMT3	Fermitin family homolog 3	0.0036	0.0002	14.62	0.0245	0.0050	4.87
Q9NZ43	USE1	USE1	Vesicle transport protein USE1	-	-	-/-	0.0053	-	+/-
O60763	USO1	USO1	General vesicular transport factor p115	0.0056	0.0041	1.36	0.0268	0.0212	1.26
Q93008	USP9X	USP9X	Probable ubiquitin carboxyl-terminal hydrolase FAF-X	0.0001	0.0000	1.60	0.0050	0.0039	1.27
P46939	UTRN	UTRN	Utrophin	-	-	-/-	0.0006	-	+/-
Q9P2Y5	UVRAG	UVRAG	UV radiation resistance-associated gene protein	0.0001	-	+/-	-	-	-/-
Q9UBK9	UXT	UXT	Protein UXT	0.0151	0.0005	31.26	-	-	-/-
P61421	VA0D1	ATP6V0D1	V-type proton ATPase subunit d 1	0.0087	0.0004	21.18	0.0251	0.0062	4.07
Q08AM6	VAC14	VAC14	Protein VAC14 homolog	-	-	-/-	0.0023	0.0024	0.97
P51809	VAMP7	VAMP7	Vesicle-associated membrane protein 7	0.0007	-	+/-	0.0121	-	+/-
Q9P0L0	VAPA	VAPA	Vesicle-associated membrane protein-associated protein A	0.0031	0.0022	1.44	0.0299	0.0214	1.39
O95292	VAPB	VAPB	Vesicle-associated membrane protein-associated protein B/C	0.0048	0.0027	1.75	0.0229	0.0172	1.33
P50552	VASP	VASP	Vasodilator-stimulated phosphoprotein	0.0075	0.0013	5.68	0.0151	-	+/-
Q99536	VAT1	VAT1	Synaptic vesicle membrane protein VAT-1 homolog	0.0338	0.0136	2.49	0.0850	0.0436	1.95

P38606	VATA	ATP6V1A	V-type proton ATPase catalytic subunit A	0.0168	0.0086	1.94	0.0329	0.0321	1.03
P15313	VATB1	ATP6V1B1	V-type proton ATPase subunit B, kidney isoform	0.0003	-	+/-	-	-	-/-
P21281	VATB2	ATP6V1B2	V-type proton ATPase subunit B, brain isoform	0.0144	0.0076	1.89	0.0313	0.0197	1.59
P21283	VATC1	ATP6V1C1	V-type proton ATPase subunit C 1	0.0005	-	+/-	0.0113	0.0038	2.95
Q9Y5K8	VATD	ATP6V1D	V-type proton ATPase subunit D	0.0010	0.0021	0.48	-	-	-/-
P36543	VATE1	ATP6V1E1	V-type proton ATPase subunit E 1	0.0114	0.0078	1.46	0.0079	-	+/-
Q16864	VATF	ATP6V1F	V-type proton ATPase subunit F	0.0386	0.0378	1.02	-	-	-/-
O75348	VATG1	ATP6V1G1	V-type proton ATPase subunit G 1	0.0002	0.0007	0.34	-	-	-/-
Q9UI12	VATH	ATP6V1H	V-type proton ATPase subunit H	-	0.0003	-/+	0.0070	0.0072	0.97
P27449	VATL	ATP6V0C	V-type proton ATPase 16 kDa proteolipid subunit	-	-	-/-	0.0671	-	+/-
P52735	VAV2	VAV2	Guanine nucleotide exchange factor VAV2	0.0002	-	+/-	0.0026	0.0042	0.62
P19320	VCAM1	VCAM1	Vascular cell adhesion protein 1	0.0043	0.0014	3.02	0.0008	-	+/-
P21796	VDAC1	VDAC1	Voltage-dependent anion-selective channel protein 1	0.2506	0.1602	1.56	0.1993	0.1667	1.20
P45880	VDAC2	VDAC2	Voltage-dependent anion-selective channel protein 2	0.1332	0.0793	1.68	0.0688	0.0682	1.01
Q9Y277	VDAC3	VDAC3	Voltage-dependent anion-selective channel protein 3	0.0433	0.0391	1.11	0.0615	0.0281	2.19
Q00341	VIGLN	HDLBP	Vigilin	0.0151	0.0089	1.69	0.0201	0.0185	1.09

P09327	VILI	VIL1	Villin-1	0.0095	-	+/-	0.0256	-	+/-
P08670	VIME	VIM	Vimentin	0.5661	0.1872	3.02	0.2340	0.0959	2.44
P18206	VINC	VCL	Vinculin	0.0346	0.0182	1.90	0.0330	0.0336	0.98
O60504	VINEX	SORBS3	Vinexin	0.0017	0.0030	0.57	0.0062	-	+/-
P62760	VISL1	VSNL1	Visinin-like protein 1	-	0.0053	-/+	-	0.0125	-/+
P38435	VKGC	GGCX	Vitamin K-dependent gamma-carboxylase	0.0027	0.0004	7.53	0.0180	0.0087	2.07
O00534	VMA5A	VWA5A	von Willebrand factor A domain-containing protein 5A	-	-	-/-	0.0054	0.0027	2.00
O95497	VNN1	VNN1	Pantetheinase	0.0068	0.0006	11.41	0.0207	-	+/-
O95498	VNN2	VNN2	Vascular non-inflammatory molecule 2	0.0003	-	+/-	0.0051	-	+/-
Q7Z7G8	VP13B	VPS13B	Vacuolar protein sorting-associated protein 13B	0.0000	-	+/-	-	-	-/-
Q709C8	VP13C	VPS13C	Vacuolar protein sorting-associated protein 13C	0.0001	-	+/-	0.0017	0.0007	2.43
O75436	VP26A	VPS26A	Vacuolar protein sorting-associated protein 26A	0.0012	0.0002	5.47	0.0113	0.0091	1.24
Q4G0F5	VP26B	VPS26B	Vacuolar protein sorting-associated protein 26B	0.0002	0.0006	0.38	0.0040	0.0064	0.63
O14972	VP26C	VPS26C	Vacuolar protein sorting-associated protein 26C	-	0.0004	-/+	-	-	-/-
Q7Z3J2	VP35L	VPS35L	VPS35 endosomal protein-sorting factor-like	-	-	-/-	0.0017	0.0026	0.64

Q93050	VPP1	ATP6V0A1	V-type proton ATPase 116 kDa subunit a isoform 1	0.0014	-	+/-	0.0105	-	+/-
Q13488	VPP3	TCIRG1	V-type proton ATPase 116 kDa subunit a3	0.0002	-	+/-	0.0087	-	+/-
Q9H270	VPS11	VPS11	Vacuolar protein sorting-associated protein 11 homolog	0.0001	-	+/-	-	-	-/-
Q9H269	VPS16	VPS16	Vacuolar protein sorting-associated protein 16 homolog	0.0001	-	+/-	0.0029	-	+/-
Q9P253	VPS18	VPS18	Vacuolar protein sorting-associated protein 18 homolog	-	-	-/-	0.0029	-	+/-
Q9BRG1	VPS25	VPS25	Vacuolar protein-sorting-associated protein 25	0.0131	0.0131	1.00	0.0098	0.0161	0.61
Q9UK41	VPS28	VPS28	Vacuolar protein sorting-associated protein 28 homolog	0.0020	0.0014	1.36	-	-	-/-
Q9UBQ0	VPS29	VPS29	Vacuolar protein sorting-associated protein 29	0.0280	0.0170	1.65	0.0200	0.0128	1.56
Q96QK1	VPS35	VPS35	Vacuolar protein sorting-associated protein 35	0.0060	0.0032	1.88	0.0202	0.0143	1.41
Q86VN1	VPS36	VPS36	Vacuolar protein-sorting-associated protein 36	-	0.0008	-/+	0.0074	0.0056	1.32
Q96JC1	VPS39	VPS39	Vam6/Vps39-like protein	-	-	-/-	0.0027	-	+/-
P49754	VPS41	VPS41	Vacuolar protein sorting-associated protein 41 homolog	0.0001	-	+/-	-	-	-/-
Q9NRW7	VPS45	VPS45	Vacuolar protein sorting-associated protein 45	0.0002	-	+/-	0.0041	0.0028	1.47
Q9UN37	VPS4A	VPS4A	Vacuolar protein sorting-associated protein 4A	0.0008	0.0003	2.76	0.0032	0.0033	0.97

O75351	VPS4B	VPS4B	Vacuolar protein sorting-associated protein 4B	0.0018	0.0017	1.07	0.0084	0.0068	1.23
Q96JG6	VPS50	VPS50	Syndetin	-	-	-/-	0.0016	-	+/-
Q8N1B4	VPS52	VPS52	Vacuolar protein sorting-associated protein 52 homolog	-	-	-/-	0.0023	0.0024	0.97
Q9NP79	VTA1	VTA1	Vacuolar protein sorting-associated protein VTA1 homolog	0.0024	0.0048	0.49	-	-	-/-
P02774	VTDB	GC	Vitamin D-binding protein	0.0358	0.0152	2.35	0.0417	0.0220	1.89
Q9UEU0	VTI1B	VTI1B	Vesicle transport through interaction with t-SNAREs homolog 1B	-	0.0003	-/+	-	-	-/-
P04004	VTNC	VTN	Vitronectin	0.0334	0.0320	1.05	0.0370	0.0303	1.22
Q6PCB0	VWA1	VWA1	von Willebrand factor A domain-containing protein 1	0.0133	0.0003	39.24	0.0207	-	+/-
A3KMH1	VWA8	VWA8	von Willebrand factor A domain-containing protein 8	0.0020	0.0013	1.60	0.0114	0.0113	1.01
P04275	VWF	VWF	von Willebrand factor	0.0027	0.0002	11.19	-	-	-/-
Q9BTA9	WAC	WAC	WW domain-containing adapter protein with coiled-coil	0.0001	-	+/-	-	-	-/-
Q9Y3C0	WASC3	WASHC3	WASH complex subunit 3	0.0004	0.0031	0.13	-	-	-/-
Q2M389	WASC4	WASHC4	WASH complex subunit 4	0.0006	0.0001	8.54	0.0032	0.0027	1.22
Q12768	WASC5	WASHC5	WASH complex subunit 5	-	-	-/-	0.0061	0.0030	2.00
Q9Y6W5	WASF2	WASF2	Wiskott-Aldrich syndrome protein family member 2	0.0004	0.0020	0.19	0.0054	-	+/-

A8K0Z3	WASH1	WASHC1	WASH complex subunit 1	0.0001	-	+/-	-	-	-/-
C4AMC7	WASH3	WASH3P	Putative WAS protein family homolog 3	0.0001	0.0002	0.67	-	-	-/-
Q9NQA3	WASH6	WASH6P	WAS protein family homolog 6	0.0002	-	+/-	-	-	-/-
O00401	WASL	WASL	Neural Wiskott-Aldrich syndrome protein	0.0009	0.0003	3.42	0.0049	-	+/-
P42768	WASP	WAS	Wiskott-Aldrich syndrome protein	0.0009	-	+/-	-	-	-/-
Q969T9	WBP2	WBP2	WW domain-binding protein 2	0.0079	0.0039	2.01	-	-	-/-
Q8IWB7	WDFY1	WDFY1	WD repeat and FYVE domain-containing protein 1	0.0002	0.0003	0.61	-	-	-/-
O75083	WDR1	WDR1	WD repeat-containing protein 1	0.0374	0.0225	1.66	0.0449	0.0491	0.91
Q9BZH6	WDR11	WDR11	WD repeat-containing protein 11	-	-	-/-	0.0047	0.0016	3.01
Q9GZL7	WDR12	WDR12	Ribosome biogenesis protein WDR12	0.0009	0.0004	2.55	-	-	-/-
Q9H1Z4	WDR13	WDR13	WD repeat-containing protein 13	0.0002	-	+/-	-	-	-/-
Q9H7D7	WDR26	WDR26	WD repeat-containing protein 26	0.0003	-	+/-	-	-	-/-
Q9Y2I8	WDR37	WDR37	WD repeat-containing protein 37	0.0002	-	+/-	-	-	-/-
Q5JSH3	WDR44	WDR44	WD repeat-containing protein 44	0.0004	-	+/-	0.0018	-	+/-
P61964	WDR5	WDR5	WD repeat-containing protein 5	0.0004	0.0022	0.17	0.0113	-	+/-
Q9H6Y2	WDR55	WDR55	WD repeat-containing protein 55	0.0008	0.0006	1.29	-	-	-/-
Q9GZS3	WDR61	WDR61	WD repeat-containing protein 61	0.0368	0.0184	2.00	0.0127	-	+/-
Q3MJ13	WDR72	WDR72	WD repeat-containing protein 72	0.0001	0.0002	0.33	-	-	-/-
Q8IWA0	WDR75	WDR75	WD repeat-containing protein 75	-	-	-/-	0.0022	-	+/-

Q9H967	WDR76	WDR76	WD repeat-containing protein 76	-	0.0002	-/+	-	-	-/-
Q6UXN9	WDR82	WDR82	WD repeat-containing protein 82	0.0010	0.0009	1.19	0.0056	-	+/-
A4D1P6	WDR91	WDR91	WD repeat-containing protein 91	-	-	-/-	0.0036	0.0024	1.47
Q9Y5W5	WIF1	WIF1	Wnt inhibitory factor 1	0.0024	-	+/-	-	-	-/-
Q9Y484	WIPI4	WDR45	WD repeat domain phosphoinositide-interacting protein 4	0.0002	-	+/-	-	-	-/-
O95785	WIZ	WIZ	Protein Wiz	0.0001	-	+/-	-	-	-/-
Q14191	WRN	WRN	Bifunctional 3'-5' exonuclease/ATP-dependent helicase WRN	-	0.0001	-/+	-	-	-/-
P47989	XDH	XDH	Xanthine dehydrogenase/oxidase	0.0023	0.0070	0.33	0.0026	0.0238	0.11
P98170	XIAP	XIAP	E3 ubiquitin-protein ligase XIAP	-	0.0003	-/+	-	-	-/-
A4UGR9-4	XIRP2	XIRP2	Isoform 4 of Xin actin-binding repeat-containing protein 2	0.0013	-	+/-	-	-	-/-
Q5GH77	XKR3	XKR3	XK-related protein 3	-	0.0002	-/+	-	-	-/-
O14980	XPO1	XPO1	Exportin-1	0.0003	0.0004	0.77	0.0202	0.0122	1.66
P55060	XPO2	CSE1L	Exportin-2	0.0006	0.0004	1.40	0.0223	0.0174	1.28
Q9UIA9	XPO7	XPO7	Exportin-7	0.0001	-	+/-	0.0029	-	+/-
O43592	XPOT	XPOT	Exportin-T	0.0001	-	+/-	0.0042	-	+/-
Q9NQW7	XPP1	XPNPEP1	Xaa-Pro aminopeptidase 1	0.0003	0.0006	0.56	0.0079	0.0064	1.23
O43895	XPP2	XPNPEP2	Xaa-Pro aminopeptidase 2	0.0003	-	+/-	-	-	-/-
Q9NQH7	XPP3	XPNPEP3	Xaa-Pro aminopeptidase 3	0.0003	0.0010	0.26	0.0076	0.0078	0.97

Q13426	XRCC4	XRCC4	DNA repair protein XRCC4	0.0002	-	+/-	-	-	-/-
P13010	XRCC5	XRCC5	X-ray repair cross-complementing protein 5	0.0145	0.0074	1.95	0.0337	0.0177	1.90
P12956	XRCC6	XRCC6	X-ray repair cross-complementing protein 6	0.0173	0.0098	1.75	0.0392	0.0330	1.19
Q9H0D6	XRN2	XRN2	5'-3' exoribonuclease 2	-	-	-/-	0.0034	0.0017	1.97
O75695	XRP2	RP2	Protein XRP2	0.0042	0.0011	3.73	-	-	-/-
O75191	XYLB	XYLB	Xylulose kinase	0.0032	0.0083	0.38	0.0098	0.0352	0.28
O75063	XYLK	FAM20B	Glycosaminoglycan xylosylkinase	-	-	-/-	0.0041	-	+/-
P67809	YBOX1	YBX1	Nuclease-sensitive element-binding protein 1	0.0051	0.0023	2.19	0.0117	0.0062	1.90
P16989	YBOX3	YBX3	Y-box-binding protein 3	0.0012	-	+/-	-	-	-/-
P07947	YES	YES1	Tyrosine-protein kinase Yes	-	0.0006	-/+	-	0.0033	-/+
O95070	YIF1A	YIF1A	Protein YIF1A	-	-	-/-	-	0.0101	-/+
Q9GZM5	YIPF3	YIPF3	Protein YIPF3	-	-	-/-	0.0084	-	+/-
Q9BSR8	YIPF4	YIPF4	Protein YIPF4	0.0010	-	+/-	-	-	-/-
Q96EC8	YIPF6	YIPF6	Protein YIPF6	-	-	-/-	0.0109	-	+/-
O15498	YKT6	YKT6	Synaptobrevin homolog YKT6	0.0007	-	+/-	-	-	-/-
P49750	YLPM1	YLPM1	YLP motif-containing protein 1	-	-	-/-	0.0007	-	+/-
Q96TA2	YMEL1	YME1L1	ATP-dependent zinc metalloprotease YME1L1	-	-	-/-	0.0020	-	+/-
P62699	YPEL5	YPEL5	Protein yippee-like 5	0.0004	0.0006	0.67	-	-	-/-
Q9Y5A9	YTHD2	YTHDF2	YTH domain-containing family protein 2	0.0004	-	+/-	-	-	-/-

Q7Z739	YTHD3	YTHDF3	YTH domain-containing family protein 3	0.0013	0.0005	2.33	0.0039	-	+/-
P59817	Z280A	ZNF280A	Zinc finger protein 280A	0.0025	-	+/-	-	-	-/-
Q96KM6	Z512B	ZNF512B	Zinc finger protein 512B	0.0001	0.0006	0.21	-	-	-/-
P25311	ZA2G	AZGP1	Zinc-alpha-2-glycoprotein	0.0139	0.0198	0.70	0.0161	0.0128	1.25
O60293	ZC3H1	ZFC3H1	Zinc finger C3H1 domain-containing protein	0.0000	-	+/-	-	-	-/-
Q8WU90	ZC3HF	ZC3H15	Zinc finger CCCH domain-containing protein 15	0.0001	0.0002	0.41	-	-	-/-
Q7Z2W4	ZCCHV	ZC3HAV1	Zinc finger CCCH-type antiviral protein 1	0.0005	0.0001	5.27	0.0040	0.0018	2.20
Q6NZY4	ZCHC8	ZCCHC8	Zinc finger CCHC domain-containing protein 8	0.0008	0.0005	1.53	-	-	-/-
Q8TCF1	ZFAN1	ZFAND1	AN1-type zinc finger protein 1	-	0.0003	-/+	-	-	-/-
Q96DA0	ZG16B	ZG16B	Zymogen granule protein 16 homolog B	0.0028	0.0008	3.77	-	-	-/-
Q8N5A5	ZGPAT	ZGPAT	Zinc finger CCCH-type with G patch domain-containing protein	0.0004	-	+/-	-	-	-/-
Q96NC0	ZMAT2	ZMAT2	Zinc finger matrin-type protein 2	-	0.0002	-/+	-	-	-/-
O43670	ZN207	ZNF207	BUB3-interacting and GLEBS motif-containing protein ZNF207	0.0002	-	+/-	-	-	-/-
O60281	ZN292	ZNF292	Zinc finger protein 292	-	-	-/-	-	0.0009	-/+
Q5VUA4	ZN318	ZNF318	Zinc finger protein 318	0.0000	-	+/-	-	-	-/-
Q86UD4	ZN329	ZNF329	Zinc finger protein 329	-	-	-/-	-	0.0028	-/+
Q06730	ZN33A	ZNF33A	Zinc finger protein 33A	0.0001	-	+/-	-	-	-/-

P17032	ZN37A	ZNF37A	Zinc finger protein 37A	0.0013	0.0002	8.54	-	-	-/-
Q6P9A3	ZN549	ZNF549	Zinc finger protein 549	0.0004	-	+/-	-	-	-/-
Q8NEP9	ZN555	ZNF555	Zinc finger protein 555	-	0.0001	-/+	-	-	-/-
Q5T7W0	ZN618	ZNF618	Zinc finger protein 618	0.0001	-	+/-	-	-	-/-
Q14966	ZN638	ZNF638	Zinc finger protein 638	0.0001	-	+/-	-	-	-/-
O60232	ZNRD2	ZNRD2	Protein ZNRD2	0.0017	0.0055	0.32	-	-	-/-
Q8NHG8	ZNRF2	ZNRF2	E3 ubiquitin-protein ligase ZNRF2	0.0014	0.0007	2.06	-	-	-/-
Q8NEW0	ZNT7	SLC30A7	Zinc transporter 7	-	-	-/-	0.0147	-	+/-
Q07157	ZO1	TJP1	Tight junction protein ZO-1	0.0010	0.0002	5.60	0.0012	0.0010	1.19
Q9UDY2	ZO2	TJP2	Tight junction protein ZO-2	0.0008	0.0001	12.52	-	0.0011	-/+
O95049	ZO3	TJP3	Tight junction protein ZO-3	0.0002	-	+/-	-	-	-/-
Q9UK55	ZPI	SERPINA10	Protein Z-dependent protease inhibitor	-	-	-/-	0.0055	-	+/-
O75312	ZPR1	ZPR1	Zinc finger protein ZPR1	0.0002	0.0020	0.08	-	-	-/-
O43264	ZW10	ZW10	Centromere/kinetochore protein zw10 homolog	0.0002	-	+/-	0.0032	-	+/-
Q15942	ZYX	ZYX	Zyxin	0.0016	-	+/-	0.0047	-	+/-
O43149	ZZEF1	ZZEF1	Zinc finger ZZ-type and EF-hand domain-containing protein 1	0.0001	-	+/-	-	-	-/-
